# High-Resolution Tuning of Iridium(III) 4′-Aryl-terpy Chromophores: A Hammett Parameter-Guided General Methodology for Systematic Property Control through Orbital Decoupling

**DOI:** 10.1021/acs.inorgchem.5c04650

**Published:** 2026-02-10

**Authors:** Erica S. Knorr, Jordan C. Kelly, Ryan B. Gaynor, Thomas N. Rohrabaugh, Caleb A. Brown, Daniel P. Harrison

**Affiliations:** † 1024 U.S. Army Combat Capabilities Development Command Army Research Laboratory, 2800 Powder Mill Rd, Adelphi, Maryland 20783, United States; ‡ Department of Chemical and Biological Science and Engineering, 6613 United States Military Academy, West Point, New York 10996, United States; § Department of Chemistry, Virginia Military Institute, 401 Maury-Brooke Hall, Lexington, Virginia 24450, United States

## Abstract

A series of six phenyl-substituted 4′-phenyl-2,2′:6′,2″-terpyridine, **RPhTerpy**, iridium­(III) complexes of the form [Ir­(**RPhTerpy**)­(ppy)­Cl]­(PF_6_), where ppy is C^N cyclometalated 2-(phenyl)­pyridine, and with Hammett parameters spanning 1.69 units, were synthesized using microwave-assisted reaction procedures and characterized via physical (cyclic voltammetry, NMR, crystallography) and photophysical methods (absorption, emission, time-correlated single photon counting, Franck–Condon line shape analysis). The iridium complexes’ redox potentials, electrochemically determined ground state HOMO–LUMO gap (eHLG), photophysically determined energy gap between ground- and excited-state (*E*_00_, 77 K), estimated excited-state reduction potential (*E*(Ir*^/–^)), and time-dependent density functional theory predicted HOMO–LUMO gap and lowest energy transition (LET) correlate strongly to the ligands’ Hammett parameters, suggesting that the Hammett parameter can be used as a convenient method to model and fine-tune the physical and photophysical characteristics for this series of Ir­(III) complexes. These analyses reveal similar correlations when applied to data for emissive complexes previously reported. Experimental and computational modeling data indicate that the HOMO and LUMO are effectively decoupled, suggesting independent control over both is possible and offering a general methodology for high-resolution design of desired characteristics.

## Graphical abstract

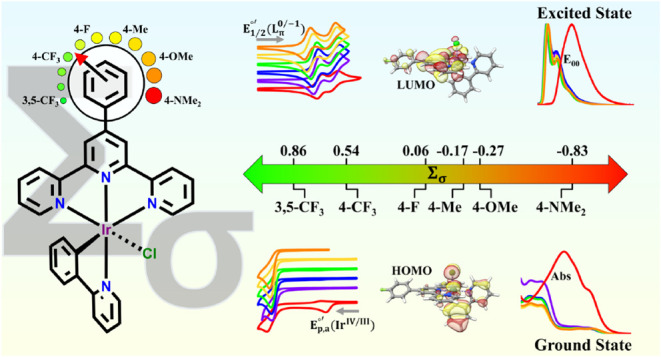

## Introduction

Fine-tuning physical and photophysical properties of transition metal complexes is a central premise in physical inorganic chemistry and is dictated by the electronic properties of ligands chelated to metals. Among the mainstay ligands that have been widely utilized as platforms for transition metal complexes are those derived from 2,2′:6′,2″-terpyridine (**Terpy**), due to their versatility in controlling electronic and steric properties. **Terpy** complexes have found numerous applications in fields such as catalysis^1–9^ and photochemistry.^10–14^ Slattery and Sjödin evaluated electrochemical (and other) properties of homoleptic bis-**Terpy** complexes chelated to cobalt,^6,15^ iron,^15^ and manganese,^16^ of the form [M­(**4**′**-R-Terpy**)_2_]^2+^ and correlated the substituent Hammett parameters with *M* ^III/II^ and ligand-centered reduction potentials. A similar analysis with ruthenium and osmium also revealed an appreciable correlation between reduction potentials and substituent π-acidities (Figure 1).^17,18^

**Figure 1:**
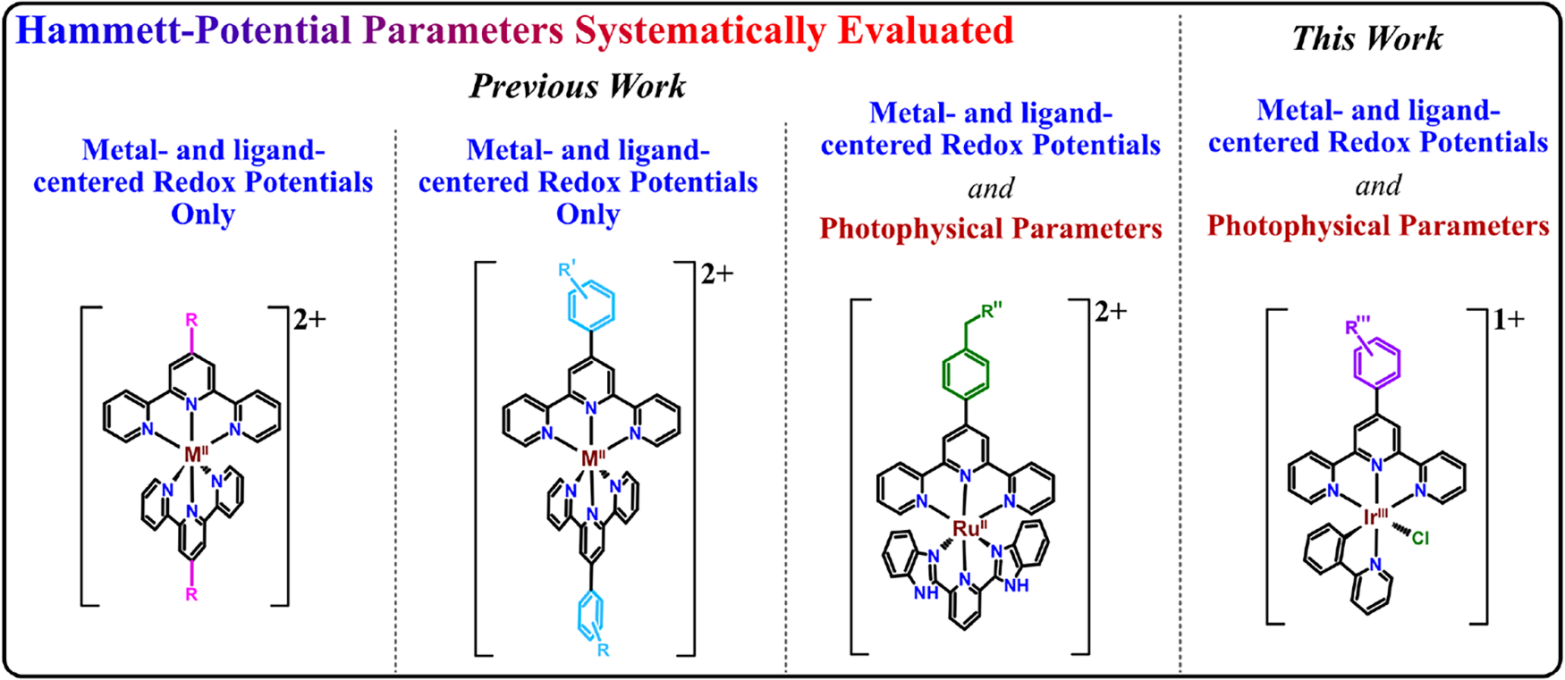
Previous work: Hammett-Potential analyses of homoleptic **Terpy**-derived metal complexes containing a variety of functional groups and metals have been performed with metal- and ligand-centered redox potentials. Hammett-Potential evaluation of both metal- and ligand-centered redox potentials and photophysical parameters have been evaluated with [(H_2_pbbzim)­Ru­(4′-R′PhTerpy)]­(PF_6_)_2_ ^20^ and, in this work, a [Ir­(**RPhTerpy**)­(ppy)­Cl]­(PF_6_) series.

These works were expanded upon to evaluate the impact of separating the functional group from the **Terpy** by including a phenyl spacer.^19^ Hammett-Redox potential analysis revealed that phenyl-spacers result in 60–80% attenuation of electronic coupling as compared to the **4**′**-R-Terpy** analog, depending upon the metal and redox couple analyzed.^19^

Reports correlating metal complexes derived from 4′-substituted **Terpy**, such as 77 K emission wavelengths/energies (*E*_00_), the estimated excited-state reduction potential, etc., are rare. Baitalik, however, performed a Hammett analysis evaluating photophysical properties of [(H_2_pbbzim)­Ru­(4′-R′PhTerpy)]­(PF_6_)_2_, where H_2_pbbzim = 2,6-bis­(benzimidazole-2-yl)­pyridine and **4**′**-R**′**PhTerpy** are 4′-tolyl-substituted terpyridine ligands, which revealed a strong correlation between the Hammett parameter and various properties.^20^

Given the utility of iridium chromophores as organic light-emitting diode (OLED) materials,^21–23^ cellular staining agents,^24–26^ photoredox catalysts for chemical synthesis,^27–29^ and solar fuels production,^2,9,30–33^ in addition to the importance of controlling their physical and photophysical properties for these and other applications, it is desirable to have a general and robust handle by which to model such properties of emissive molecules. Thus, we sought to determine (1) if the redox potential fine-tuning imparted by systematically adjusting the complexes’ π-acidity (as quantified by Hammett parameters) extended to an iridium series derived from **Terpy**, (2) if, and to what magnitude, the π-acidity of the ligands correlated to trends in the complexes’ photophysical properties, and (3) if the complexes’ properties could be selectively tuned by virtue of decoupling the HOMO and LUMO with alternative architectures to [M­(**Terpy**)_2_]^2+^.

Herein, we report a new microwave synthetic procedure for a series of [Ir­(**RPhTerpy**)­(ppy)­Cl]­(PF_6_) complexes, where **RPhTerpy** is substituted 4′-phenyl-2,2′:6′,2″-terpyridine and where ppy is C^N cyclometalated 2-(phenyl)­pyridine, as well as delineating their electrochemical details, crystallographic features, and photophysical properties. Electrochemical redox potentials, time-dependent density functional theory (TD-DFT)-predicted HOMO–LUMO transitions, and the photophysically determined energy gap between ground and excited states (*E*_00_) are evaluated and correlated well to the Hammett parameter of the 4′-aryl functional group, which spans 1.69 units, from +0.86 to −0.83, allowing for fine-tuning the behavior of the iridium complexes. Furthermore, processing data from reported emissive complexes in a manner analogous to this study suggests that the capacity to fine-tune physical and photophysical properties is broadly applicable across diverse metal–ligand systems when employing the Hammett analysis as a guide.

## Results

### Synthesis and Characterization

A series of six 4′-aryl-substituted iridium­(III) complexes of the general form [Ir­(**RPhTerpy**)­(ppy)­Cl]­(PF_6_) were synthesized with a range of π-acidities (e.g., electron-withdrawing groups: fluoro-, trifluoromethyl-, and electron-donating methyl-, methoxy-, dimethylamino-) to investigate the effect the substituent has on the complexes’ properties (Figure 2). The groups were systematically incorporated on the aryl ring at the 4′-position of the phenyl-terpyridines via Krönke pyridine procedures,^19,35–37^ and the resulting meridional κ^3^-N^N^N, **RPhTerpy** ligands were then chelated to IrCl_3_·3H_2_O through a newly developed, rapid microwave-assisted reaction sequence that was derived from previously reported atmospheric pressure reactions (Figure 2). The final complexes were isolated as their hexafluorophosphate salts, PF_6_^–^, with final two-step yields of 35–64%.

**Figure 2:**
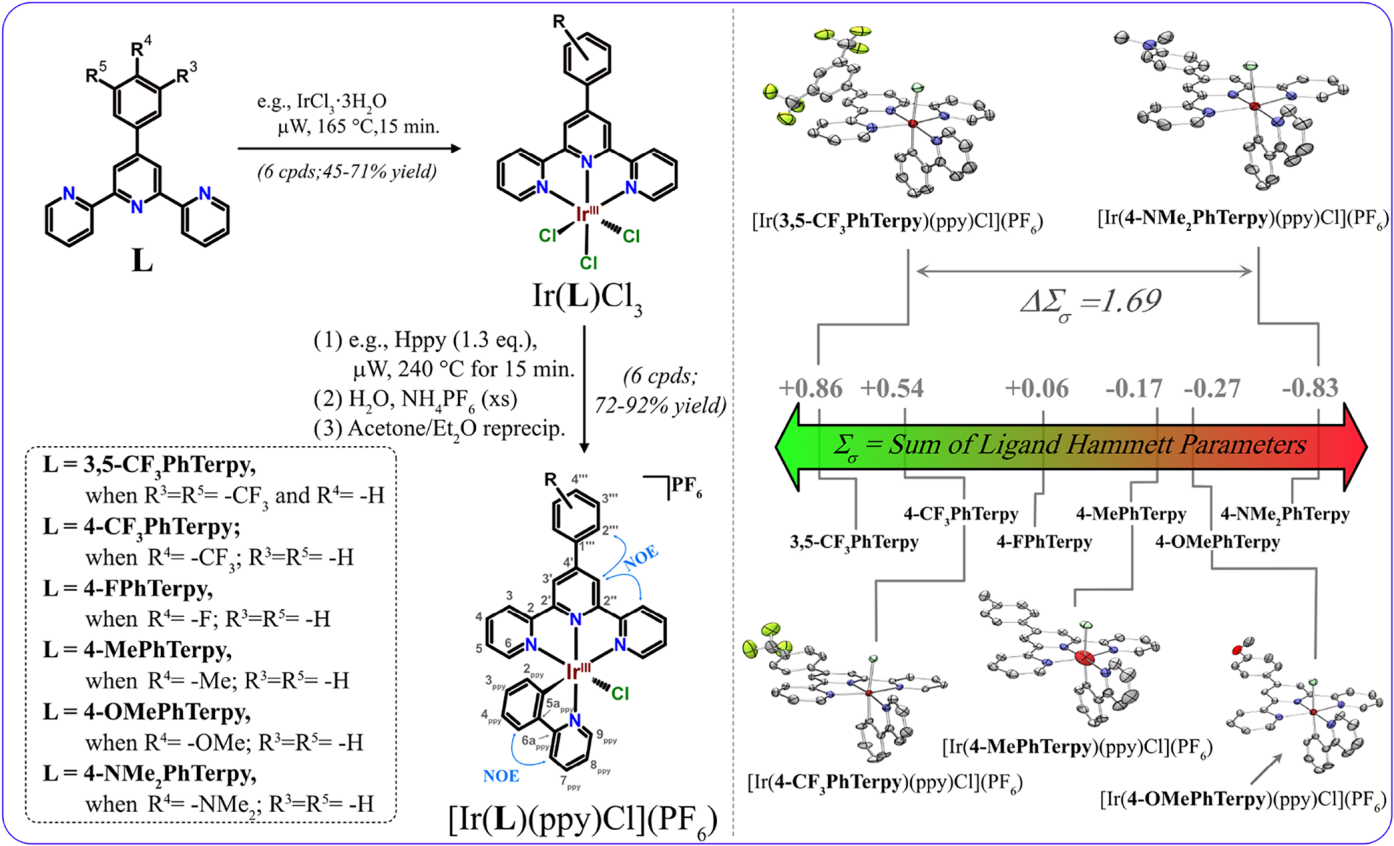
Synthetic scheme for [Ir­(**RPhTerpy**)­(ppy)­Cl]­(PF_6_) complexes and the sum of Hammett parameters (Σ_σ_ calculated according to eq 2 ^19,34^ ) of the **RPhTerpy** ligand functional groups (inlaid). Krönke pyridine synthesis provided a convenient route to **RPhTerpy**,^19,35–37^ which were used to synthesize Ir­(**RPhTerpy**)­Cl_3_ by microwave heating in ethylene glycol (e.g.). Target complexes, [Ir­(**RPhTerpy**)­(ppy)­Cl]­(PF_6_), were rapidly synthesized by heating a slight excess of 2-phenylpyridine (Hppy) with the appropriate Ir­(**RPhTerpy**)­Cl_3_. Atom numbering for NMR assignment is included in gray, with key NOEs indicated in blue double-arrows. Thermoellipsoids are depicted at 50% level for single-crystal structures of [Ir­(**RPhTerpy**)­(ppy)­Cl]­(PF_6_) complexes. Hydrogen atoms, PF_6_ counterions, and solvent molecules are omitted for clarity. Color scheme: iridium (firebrick red), carbon (gray), nitrogen (blue), oxygen (red), fluoride (green), chloride (pale green).

While the ppy ligand may form isomers with the cyclometallating phenyl carbon either *cis*- or *trans*-to-chloride, NMR analysis indicates that a single isomer of the target [Ir­(**RPhTerpy**)­(ppy)­Cl]­(PF_6_) molecules is isolated in a >20:1 ratio after crystallization.^9,38–40^ The expected number of resonances, coupling patterns, and spin systems are present via ^1^H NMR, ^1^H–^1^H DQF-COSY, ^1^H–^1^H NOESY, ^1^H–^1^H TOCSY, ^1^H–^13^C HSQC-TOCSY spectra, while the expected number of carbon NMR resonances are observed, alongside ^1^ *J*_CF_, ^2^ *J*_CF_, and ^3^ *J*_CF_ coupling patterns and constants for fluorine containing complexes ([Ir­(**3,5-CF**_**3**_ **PhTerpy**)­(ppy)­Cl]­(PF_6_), [Ir­(**4-CF**_**3**_ **PhTerpy**)­(ppy)­Cl]­(PF_6_), and [Ir­(**4-FPhTerpy**)­(ppy)­Cl]­(PF_6_)). Unambiguous proton and carbon resonance assignment was achieved with three key intraligand nuclear Overhauser effect (NOE) enhancements observed in ^1^H–^1^H NOESY spectra, where the sharp singlet associated with the central pyridine ring of terpy demonstrates a through-space interaction with (1) the adjacent (terminal) pyridine ring, (2) the ortho-protons of the 4′-aryl group, and (3) the interaction between the H5_*ppy*_ and H6_*ppy*_ protons in the ppy ligand– and long-range coupling indicated by ^1^H–^13^C HMBC, in combination with ^1^H–^1^H DQF-COSY, ^1^H–^1^H TOCSY, and ^1^H–^13^C HSQC-TOCSY experiments.

Single-crystal X-ray crystallography of five complexes confirmed the isomeric form of the κ^2^-C^N cyclometalated ppy ligand in which the carbon orients *trans*-to-chloride, as indicated by NMR analysis, and literature precedence of related compounds. For example, the ppy bite angles [N–Ir–C: 80.2(2)–80.7(2)°] and bond lengths [Ir–N: 2.073(4)–2.088(3) Å and Ir–C: 2.010(5)–2.029(3) Å] are in agreement to lengths reported for ppy in [Ir­(ppy)_3_] [N–Ir–C: 78.6(4)–79.6(4)°, Ir–N: 2.07(1)–2.095(9) Å, and Ir–C: 2.032(9)–2.06(1) Å][Bibr ref41] and other compounds (Table S10). The Ir­(III) centers in the title complexes adopt a distorted octahedral geometry due to the rigidity of the planar **RPhTerpy** ligand. As expected, the **RPhTerpy** is coordinated to the iridium through its three nitrogen atoms in a meridional fashion. The Ir–N bond lengths of the two terminal pyridine rings of the **RPhTerpy**, ranging from 2.042(4)–2.067(4) Å, are longer than the iridium–nitrogen bond of the central ring Ir–N: 1.947(3)–1.972(4) Å bond. The structural parameters are in excellent agreement with structures of a similar architecture, especially [Ir­(**PhTerpy**)­(ppy)­Cl]­(PF_6_) (see Table S10 for additional structure metrics).^9,38,39,42,43^ Similar to [Ir­(**PhTerpy**)­(ppy)­Cl]­(PF_6_), all crystal structures indicate that the aryl groups are not coplanar with the terpyridine moiety, to varying degrees in the solid state.

Matrix-assisted laser desorption ionization mass spectrometry attempts yielded unsatisfactory results even though precise results are achieved with neutral iridium complexes (Figures S70–S75).^44,45^ However, positive-mode high-resolution electrospray ionization provided additional confirmation of structure (Figures S76–S81).

### Electrochemical Analysis and DFT Modeling

Cyclic voltammetry is a convenient electroanalytical method for determining select physical properties of a species, such as redox potentials and the relative energy separation of high-lying occupied (HOMO) and low-lying unoccupied (LUMO) orbitals (i.e., the electrochemical HOMO–LUMO gap, eHLG).

Figure 3 overlays the cyclic voltammograms for the [Ir­(**RPhTerpy**)­(ppy)­Cl]­(PF_6_) series. All Ir­(IV/III) couples (*E*_*p*,*a*_^°′^(*Ir* ^IV/III^)) are irreversible at 0.100 V/s scan rate, which indicates that the complexes are decomposing to unknown species after oxidation. In all cases, the first ligand-centered reductions are electrochemically and chemically reversible. While the second reductions are similarly reversible, their wave shapes and peak-to-peak separations (Table S1) indicate that the electron-transfer rate constant is slower than that of the first reduction.

**Figure 3:**
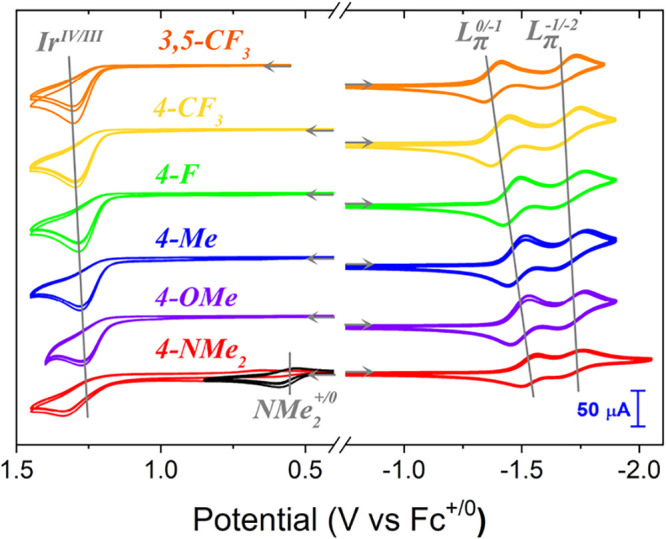
Overlay of CVs for the [Ir­(**RPhTerpy**)­(ppy)­Cl]­(PF_6_) series. All CVs were collected in MeCN at a 100 mV/s scan rate with 0.1 M TBAPF_6_ and are referenced vs Fc^+/0^ using a 3 mm diameter glassy carbon electrode at 22 ± 2 °C. Three CV cycles are plotted for each complexes anodic and cathodic voltammagram. The anodic CV scans start between 0 and +0.55 V, sweep anodically to a switching potential near +1.45 V and return, while the cathodic CV scans initialize at −0.75 V sweeping cathodically to switching potentials between −1.85 V and −2.05 V. The orange traces are 2.0 mM [Ir­(**3,5-CF**_**3**_ **PhTerpy**)­(ppy)­Cl]­(PF_6_), the yellow-orange traces are 2.2 mM [Ir­(**4-CF**_**3**_ **PhTerpy**)­(ppy)­Cl]­(PF_6_), the green traces are 1.9 mM [Ir­(**4-FPhTerpy**)­(ppy)­Cl]­(PF_6_), the blue traces are 1.9 mM [Ir­(**4-MePhTerpy**)­(ppy)­Cl]­(PF_6_), the purple traces are 2.0 mM [Ir­(**4-OMePhTerpy**)­(ppy)­Cl]­(PF_6_), and the red and black traces are 1.8 mM [Ir­(**4-NMe**_**2**_ **PhTerpy**)­(ppy)­Cl]­(PF_6_). The switching potential for the black CV is +0.85 V. Initial scan direction is indicated with gray arrows.

Qualitatively, the redox events vary systematically as a function of aryl group’s π-acidity. The metal oxidation is least impacted, with the reduction potential range spanning only about 20 mV for the series. The first reduction, however, is strongly coupled to the aryl functionality, with a reduction potential window of 120 mV (Table S1). The described electrochemical behavior is consistent with a set of previously studied [Ir­(**Terpy**)­(Rppy)­Cl]­(PF_6_) and [Ir­(**PhTerpy**)­(Rppy)­Cl]­(PF_6_) complexes, where Rppy is substituted 2-(phenyl)­pyridine.^9^

It is worth noting that [Ir­(**4-NMe**_**2**_ **PhTerpy**)­(ppy)­Cl]­(PF_6_) displays an Ir­(IV/III) redox couple more anodically shifted than that of the other members of the series, which was initially unexpected given that **4-NMe**_**2**_ **PhTerpy** is the most electron-rich ligand. However, the **4-NMe**_**2**_ **PhTerpy** complex has a fourth couple (Figure 3, in black) that is not observed in the rest of the series and is initially irreversible but which becomes reversible when the switching potential is set below the Ir­(IV/III) couple. Thus, we attribute this couple at +0.60 V vs Fc^+/0^ to the redox behavior of the high-energy lone pair of electrons in the dimethylamino-functional group. While the oxidation is likely localized on the amine functionality, its oxidation occurs first. By virtue of the complex now having a larger overall charge (i.e., 2+), the metal-centered Ir­(IV/III) becomes harder to oxidize and anodically shifts relative to the remainder of the series. The ligand-centered reductions are unaffected by the additional couple.

Density Functional Theory (DFT) calculations were performed using the Gaussian 16 software suite^46^ to further support electrochemical observations. The hybrid exchange–correlation functional B3PW91 ^47–51^ was used given its good performance in modeling optical properties of iridium complexes in particular,^50,52^ and the Kohn–Sham orbital model was utilized, given its improved description of molecular excitations.^53^ The def2-SVP basis set was used on all atoms except iridium, which used the def2-tzvp alongside the GD3 London dispersion correction. Acetonitrile solvent effects were accounted for through the conductor-like polarizable continuum model (CPCM)^54^ and frequency calculations performed at the same level of theory to ensure optimized structures were minima. Optimized structure metrics are in good agreement with crystallographic data (Table S17).

Figure 4a depicts the HOMO and LUMO of [Ir­(**4-OMePhTerpy**)­(ppy)­Cl]^+^, along with their orbital contributions. The HOMO resides primarily on ppy (33%) and Ir (29%) in preference to 4-OMePh– (<1%) or the terpyridine moiety (6%), while the LUMO resides primarily on the 4-OMePhTerpy (92%, with 5% contribution from 4-OMePh– and 87% contribution from terpy), rather than the ppy (<1%). The HOMO energies remain largely the same across the series (−6.28 to −6.23 eV), which is not surprising considering there is very little contribution from the **RPhTerpy** and is suggestive of why *E*_*p*,*a*_^°′^(*Ir* ^IV/III^) exhibits only small changes with substituent π-acidity: the HOMO is largely unaltered by variations in the ligand substituents. Second, the LUMO energies vary significantly over the series, with the LUMO decreasing in energy with increasing π-acidity. The terpy coefficient is the dominant contributor in the LUMO, and this observation is true for the rest of the complexes in the series as well, which exhibit values between 84 and 87%. Likewise, these data are consistent with significant orbital contribution on the **RPhTerpy** ligand and the large changes in *E*_1/2_^°′^(*L*_π_^(0/–1)^). These observations relating MO densities (see Tables S20–S25) and orbital energies are true for each of the complexes in the series, with the exception of [Ir­(**4-NMe**_**2**_ **PhTerpy**)­(ppy)­Cl]­(PF_6_), where the HOMO–1 resembles the HOMOs of the other members in the series (Figure 4B). Its HOMO, however, is distinct from the other complexes in the series, and is dominated by the 4-NMe_2_Ph– contribution (84%; 93% when summed with the terpy moiety), which is consistent with electrochemical data previously discussed.

**Figure 4:**
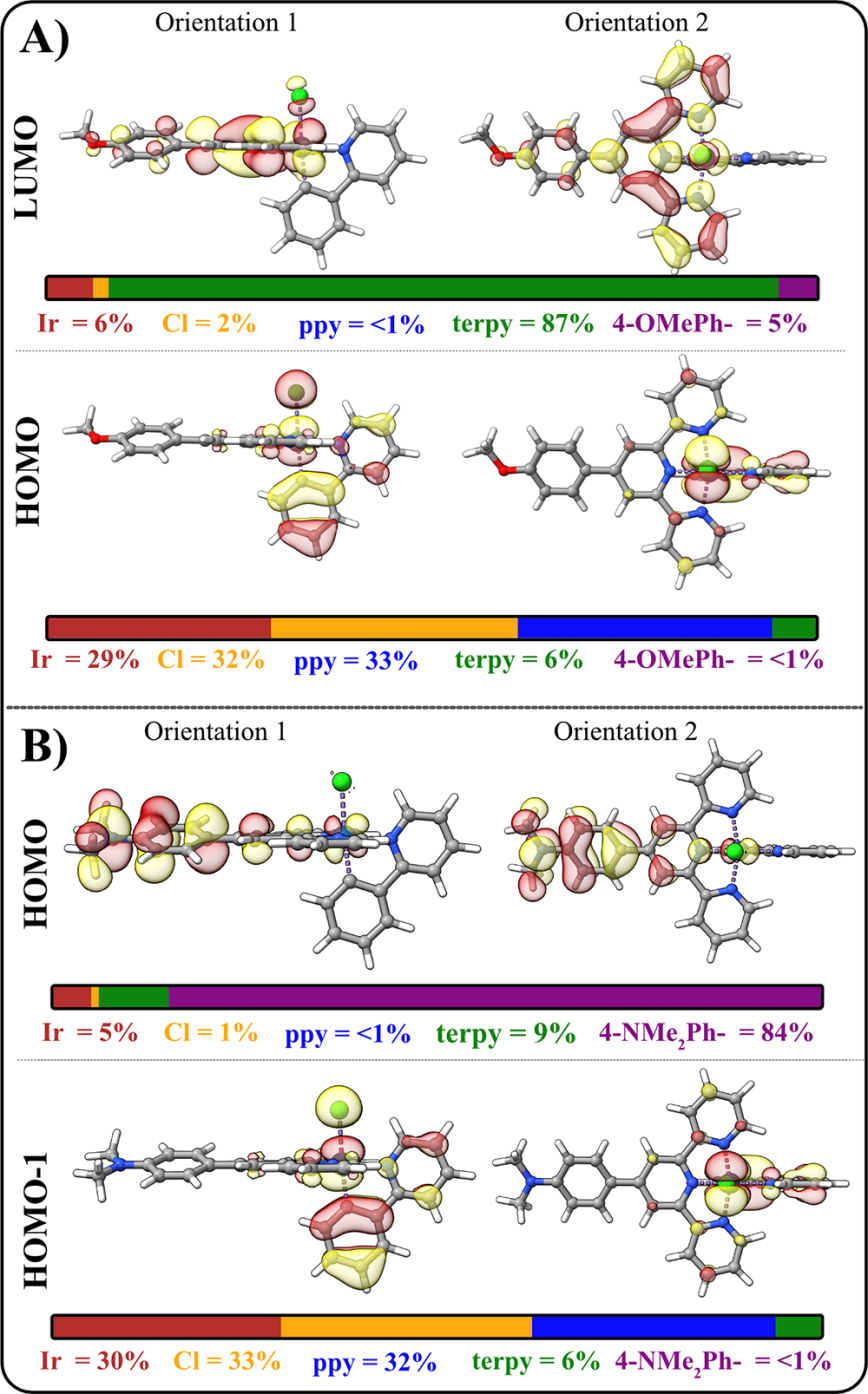
Kohn–Sham orbital density of (A) the HOMO (bottom) and LUMO (top) of [Ir­(**4-OMePhTerpy**)­(ppy)­Cl]^+^ presented in two orientations. (B) HOMO (top) and HOMO–1 (bottom) of [Ir­(**4-NMe**_**2**_ **PhTerpy**)­(ppy)­Cl]­(PF_6_). The horizontal bars are orbital composition bars indicating contributions for the five components of the complexes: Ir = red, Cl = orange, ppy moiety = blue, terpy moiety = green, RPh– moiety = purple.

Similar RPh– -dominated orbitals are identifiable for the other complexes in the series (e.g., the RPh– -dominated orbital of 3,5-CF_3_Ph– is HOMO–8; 4-CF_3_Ph– = HOMO–6; 4-FPh– = HOMO–3; 4-MePh– = HOMO–3; 4-OMePh– = HOMO–1). The energy of these orbitals increases from −8.06 eV to −6.36 eV (Figures S47 and S48 and Tables S20–S25) as the RPh– group becomes more π-basic, while at the same time, the energy of the ppy-dominated MOs (i.e., the HOMOs for these 5 complexes) again remains approximately the same (−6.25 ± 0.02 eV). Thus, the π-basicity is sufficiently extreme in [Ir­(**4-NMe**_**2**_ **PhTerpy**)­(ppy)­Cl]­(PF_6_) that the 4-NMe_2_Ph– dominated orbital increases in energy (to −5.65 eV) above its ppy-dominated orbital (at −6.19 eV) to become the HOMO and alters the nature of the lowest-energy excitation from metal-to-ligand charge transfer (MLCT) to ligand-to-ligand charge transfer (LLCT). This reversal of orbitals results in altered electrochemical behavior and later manifests itself in fundamentally different photophysical properties.

Time-Dependent Density Functional Theory (TD-DFT) with the Tamm-Dancoff Approximation was used to model the transitions of the title complexes and aid in our evaluation (Figure 5).^55^ The lowest-energy transitions (LET) are solely composed of transitions from the HOMO to the LUMO, decrease in energy (ranging from 2.41 to 2.31 eV; vertical values in Figure 5) with increasing π-acidity (excluding [Ir­(**4-NMe**_**2**_ **PhTerpy**)­(ppy)­Cl]­(PF_6_)), and follow a similar red-shift trend observed in experimental absorption spectra (Figure 6a). Computed UV–vis spectra are in reasonable agreement with experimental observations (Figures S49–S54).

**Figure 5:**
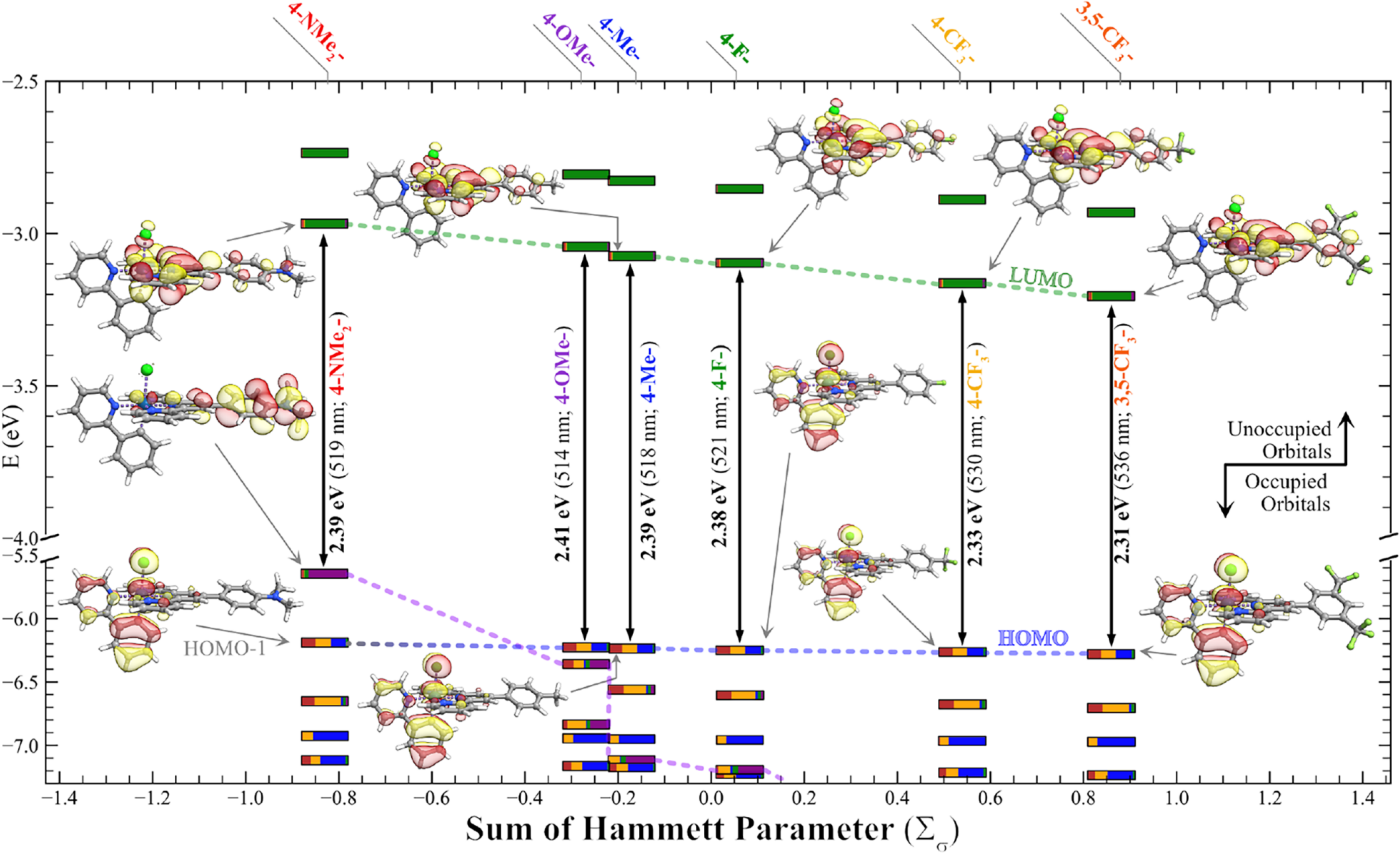
Energy diagram showing the selected Kohn–Sham MO surfaces obtained by DFT calculations for the [Ir­(**RPhTerpy**)­(ppy)­Cl]­(PF_6_) series (see the Experimental Section for the detailed functional and basis set used) plotted as a function of Σ_σ_. The MOs for [Ir­(**4-OMePhTerpy**)­(ppy)­Cl]­(PF_6_) are omitted for clarity (see Figure 4). The HOMO–1 is also depicted for [Ir­(**4-NMe**_**2**_ **PhTerpy**)­(ppy)­Cl]­(PF_6_) as it resembles the HOMOs of other complexes, while its HOMO is localized on the amine moiety. The LET calculated with TD-DFT is labeled adjacent to the black vertical arrows, with LET transition wavelength in parentheses, for each compound. The horizontal bars are orbital composition bars indicating contributions for the five defined fragments of the complexes: Ir = red, Cl = orange, ppy moiety = blue, terpy moiety = green, RPh– moiety = purple. The green dashed lines connect the terpy-dominated LUMOs of the complexes, the blue dashed lines connect the ppy-dominated HOMOs, and the purple dashed lines connect RPh– -dominated occupied orbitals.

**Figure 6:**
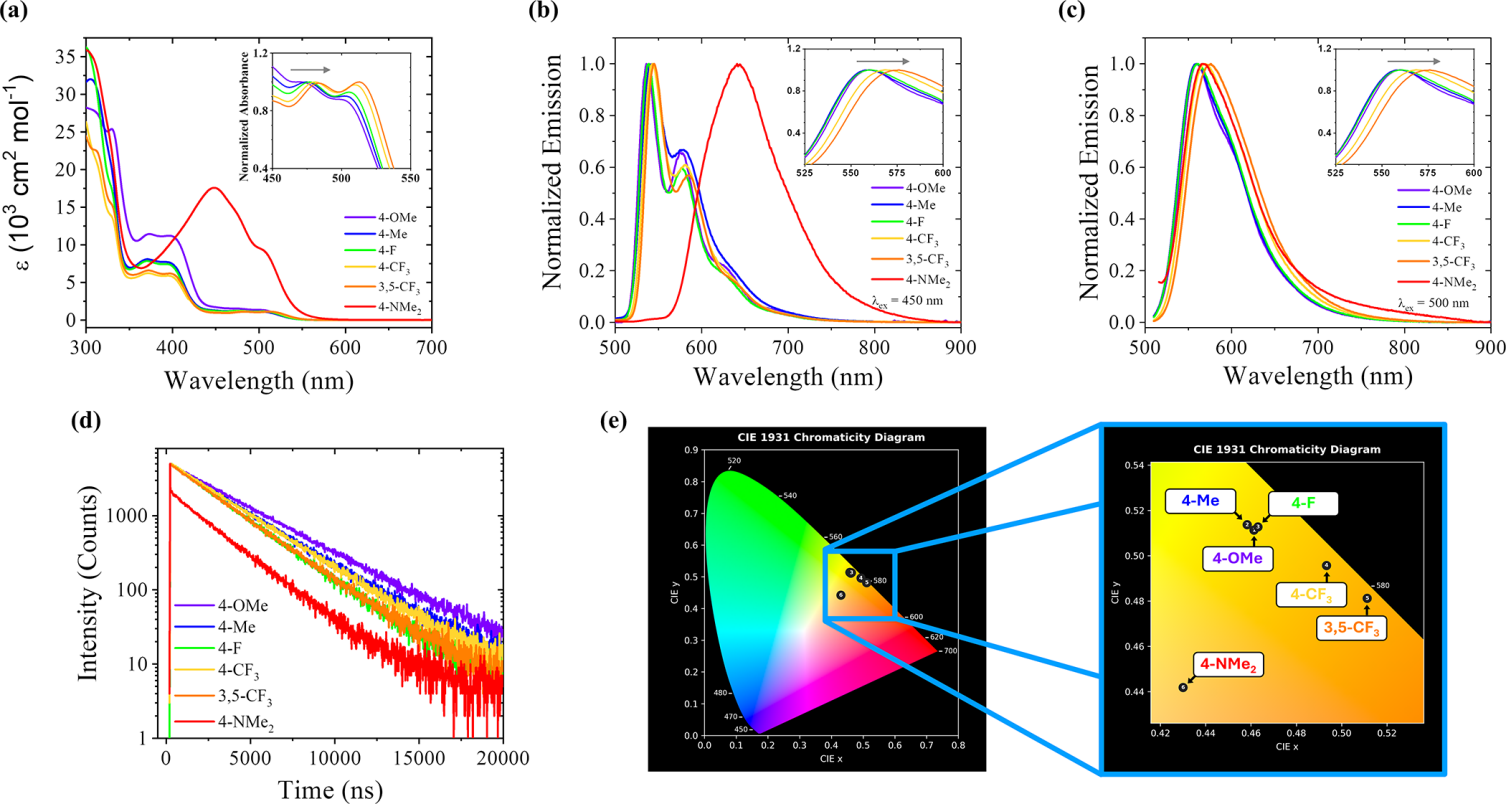
(a) Molar absorptivity of complexes in acetonitrile; (b) normalized steady-state emission spectra at 77 K (4:5 propionitrile:butyronitrile, λ_ex_ = 450 nm); (c) normalized steady-state emission spectra at room temperature (acetonitrile, λ_*ex*_ = 500 nm); (d) emission decay traces in deaerated acetonitrile (λ_ex_ = 510 nm); (e) CIE chromaticity diagram showing the color coordinates of complexes at room temperature and a zoomed-in portion focused on the 580 nm region. The insets of graphs (a–c) show the peak shifts with the gray arrows indicating decreasing electron-withdrawing character from left to right.

### Photophysical Analysis

Absorbance, photoluminescence, and photoluminescent decay were evaluated for the series. All complexes exhibit ligand-centered (LC) transitions at wavelengths less than 350 nm and MLCT transitions from approximately 350 to 550 nm, with the exception of [Ir­(**4-NMe**_**2**_ **PhTerpy**)­(ppy)­Cl]­(PF_6_) (Figure 6a), which instead exhibits a LLCT transition from 350 to 575 nm. [Ir­(**4-NMe**_**2**_ **PhTerpy**)­(ppy)­Cl]­(PF_6_) will be excluded from further discussion of photophysical trends because the ground state photophysics for [Ir­(**4-NMe**_**2**_ **PhTerpy**)­(ppy)­Cl]­(PF_6_) are based on LLCT rather than the MLCT transitions of interest. For all other complexes, the lowest-energy MLCT peak increases in energy as the substituents become more electron-withdrawing.

The Ir­(III) chromophores emit between 525 and 800 nm both at 77 K and at room temperature. At 77 K (Figure 6b), the emission is less broad and more structured than at room temperature (Figure 6c), due to the rigidochromic effect.^56,57^ The 77 K emission was further studied via Franck–Condon line shape analysis (FCLSA) to extract the energy gap between the lowest vibrational level of the ground state and the lowest vibrational level of the excited state, *E*_00_ (Table 1 and Figures S61–S66 and Tables S26–S27). As the absorbance peak of the lowest-energy MLCT increases, *E*_00_ decreases. Likewise, as *E*_00_ decreases, the emission peaks for the chromophores at room temperature decrease in energy and increase in wavelength. Emission for these complexes is relatively long-lived, with decay times ranging between 2.5 and 3.5 μs (Table 1 and Figure 6d). The emission decays are monoexponential, indicating that only one pathway is contributing to the decay rate within the laser pulse width. Decay from the triplet state is suggested by the long monoexponential decay in the absence of oxygen as well as the significant Stokes shift between absorption and emission peaks. These observations are consistent with the decay pathways of other Ir­(III) chromophores, wherein the molecules are excited to the singlet state and undergo rapid intersystem crossing before decay from the triplet state.^58–64^

**Table 1:** Summary of the Photophysical Properties of [Ir­(**RPhTerpy**)­(ppy)­Cl]­(PF_6_) Complexes^42^

complex	λ_MLCT_/nm (ε/cm^2^ mol^–1^)	λ_em_ (77 K)^*a*^/nm	λ_em_ (RT)^*b*^/nm	ϕ_*em*_^*b*^	τ_*em*_^*b*,*c*^/ μs	*k*_*nr*_^*b*^/10^5^ s^–1^	*E*_00_^*a*^/eV
[Ir(**4-OMePhTerpy**)(ppy)Cl](PF_6_)	471 (1600)	536	550	0.73(1)	3.54	0.76	2.31
[Ir(**4-MePhTerpy**)(ppy)Cl](PF_6_)	475 (1200)	543	560	0.68(1)	3.11	1.05	2.31
[Ir(**4-FPhTerpy**)(ppy)Cl](PF_6_)	477 (1300)	539	561	0.59(1)	2.67	1.52	2.30
[Ir(**4-CF**_**3**_ **PhTerpy**)(ppy)Cl](PF_6_)	481 (1200)	543	569	0.66(1)	3.00	1.13	2.28
[Ir(**3,5-CF**_**3**_ **PhTerpy**)(ppy)Cl](PF_6_)	483 (1100)	545	576	0.60(1)	2.71	1.48	2.27
[Ir(**4-NMe**_**2**_ **PhTerpy**)(ppy)Cl](PF_6_)	448 (18,000)^*d*^	641	567	<0.01	2.43^*e*^	4.09	1.92
[Ir(**4-** *N* **-(morpholino)PhTerpy**)(ppy)Cl](PF_6_)^42^		616^*f*^	575^*g*^	0.02^*g*^	4.78^*g*^	2.04^*g*^	2.01^*h*^
[Ir(**PhTerpy**)(ppy)Cl](PF_6_)^42^		540^*f*^	560^*g*^	0.63^*g*^	5.98^*g*^	0.62^*g*^	2.30^*h*^

*^a^* —In a 4:5 prop/but glass at 77 K (λ_em_ = 450 nm). *^b^* —In freeze-pump-thaw degassed acetonitrile at room temperature (λ_em_ = 500 nm). *^c^* —λ_em_ = 510 nm. ^*d*^—Absorbance and extinction coefficient at LLCT band. ^*e*^—Biexponential fit. ^*f*^—Data obtained in glassy 4:1 ethanol/methanol matrix. ^*g*^—Data obtained in deaerated acetonitrile at room temp. ^*h*^—Estimated from λ_em_ (77 K).^42^

Both the lifetime and quantum yield of the chromophores tend to decrease as *E*_00_ decreases, while the rate constant for nonradiative decay (*k_nr_*) tends to increase (Table 1 and Figure S67). It is unclear why the energy gap law is only loosely followed for this series (Figure S67a; *r* ^2^ = 0.35), but it may be a consequence of differing reorganizational energy associated with varying amounts of intraligand delocalization of the π* electron density in the MLCT excited-state that accompanies phenyl-ring rotation, which was induced by the excited state and is a phenomenon observed and evaluated by McCusker using ruthenium tris-4,4′-diaryl-2,2′-bipyridines.^65^ Triplet state optimized structures of the [Ir­(**RPhTerpy**)­(ppy)­Cl]­(PF_6_) series display varying degrees of phenyl-ring rotation as compared to their singlet state optimized geometries (Table S17), an effect also noted by Yoshikawa in dicationic complexes of **4-MePhTerpy**.^66^ Other studies have similarly attributed deviations from energy gap law to the distortion of molecular coordinates.^67–70^ FCLSA of the emission at room temperature further supports this notion. The Huang–Rhys factor, *S*, describes vibrational coupling within a complex and is known to be proportional to the square of distortion.^67^ For example, [Ir­(**4-FPhTerpy**)­(ppy)­Cl]­(PF_6_) has the highest calculated *S* value (Table S27). When this compound is removed from the energy gap law plot, *r* ^2^ increases to 0.71 (Figure S67b), indicating that the deviation may indeed be due to differences in molecular distortion and reorganizational energy. The photophysical properties of the title series are generally consistent with those of analogous complexes.^9^

Unlike the other compounds in this series, [Ir­(**4-NMe**_**2**_ **PhTerpy**)­(ppy)­Cl]­(PF_6_) exhibits high intensity absorbance between 350 and 550 nm, which we attribute to LLCT.^71–74^ Electrochemical data and computational modeling support this conclusion: the HOMO displays significant density on the 4-NMe_2_Ph– moiety rather than on the metal center and ppy ligand, where it has an outsized orbital contribution of over 80% of the HOMO (Figure 4b). In addition to differences in absorption, the emission of [Ir­(**4-NMe**_**2**_ **PhTerpy**)­(ppy)­Cl]­(PF_6_) shifts significantly between room temperature and 77 K measurements (2036 cm^–1^) compared to the other [Ir­(**RPhTerpy**)­(ppy)­Cl]­(PF_6_) complexes (475–988 cm^–1^). Unusually, [Ir­(**4-NMe**_**2**_ **PhTerpy**)­(ppy)­Cl]­(PF_6_) emission hypsochromically shifts at higher temperatures. Excitation scans performed on the room-temperature sample resemble the MLCT absorbance features observed for the other complexes rather than the expected LLCT features for [Ir­(**4-NMe**_**2**_ **PhTerpy**)­(ppy)­Cl]­(PF_6_) (Figure S68f), indicating that room-temperature emission may primarily arise from MLCT transitions. The room-temperature emission decays biexponentially, which also suggests that emission is the result of two decay pathways. A study on a [Ir­(**RPhTerpy**)­(ppy)­Cl]­(PF_6_) complex with a morpholinyl group at the 4′-position similarly observes large temperature-dependent emission shifts.^42^ In this study, the authors determine that emission arises from two energetically close excitation states. Similar to [Ir(**4-NMe**_**2**_ **PhTerpy**)(ppy)Cl](PF_6_), excitation scans on the morpholine-bearing complex at room temperature resemble MLCT features under certain conditions, further supporting that [Ir(**4-NMe**_**2**_ **PhTerpy**)(ppy)Cl](PF_6_) likely emits from a combination of two energetically close excitation states. Again, computational work supports this conclusion as the HOMO–1 of [Ir­(**4-NMe**_**2**_ **PhTerpy**)­(ppy)­Cl]­(PF_6_) is analogous to the HOMO observed for the other complexes in this series, with the LUMO primarily residing on the terpy moiety.

Organometallic chromophores with amine-bearing ligands are known to exhibit lower emission quantum yields and shorter lifetimes than their unsubstituted counterparts. This phenomenon has been observed in Pt­(II),^71,72^ Fe­(II),^75^ Ru­(II),^76^ and Ir­(III)^73,74,77^ complexes. Based on both experimental and theoretical work on these chromophores, the absorbance and emission of amine-bearing chromophores are likely influenced by intraligand or LLCT events with C^N cyclometalating ligands.^71–77^ The low quantum yields of these compounds are likely due to destabilization of the MLCT state, which makes deactivation through a nonemissive metal-centered state more favorable.^75,78^ Similar effects on the quantum yield, lifetime, and other properties of [Ir­(**4-NMe**_**2**_ **PhTerpy**)­(ppy)­Cl]­(PF_6_) are observed in this study. Given the similarity to the aforementioned work and our computational modeling, the dimethylamine is responsible for the observed deviations from the remaining members of the series.

### Hammett Analysis

Quantitative assessment of the electronic control that the aryl-substituents impart on the numerous physical and photophysical properties [Ir­(**RPhTerpy**)­(ppy)­Cl]­(PF_6_) complexes can be accomplished by performing a Hammett free energy relationship (**HFER**) analysis according to the general eqs 1 and 2.^19,34^

1 $$A\left(L\right)=\rho \Sigma_{\sigma }+A\left(H\right)$$

2 $$\Sigma_{\sigma }=\sigma_{m,3}+\sigma_{p}+\sigma_{m,5}$$

In eq 1, *A*(*L*) is a physical or photophysical parameter (e.g. $A\left(L\right)=E_{1/2}^{{}^{\circ}\prime }\left(L\right)$ when evaluating formal reduction potentials, etc.) associated with a metal complex coordinated to a particular ligand, *L*, whereas *A*(*H*) represents the same property associated with a metal complex coordinated to the nonfunctionalized ligand, in this case [Ir(**PhTerpy**)(ppy)Cl](PF_6_). The sum of Hammett parameters, Σ_σ_, is the sum of *meta* Hammett parameters at the 3-position, σ_*m*,3_, and the 5-position, σ_*m*,5_, and the *para* Hammett parameter, σ_*p*_ (when the functional groups are not hydrogen). The extent to which a given process or parameter is sensitive to substituent changes can be determined by measuring the slope, ρ, in Hammett-Potential plots, with greater magnitude slopes indicating stronger correlation between π-acidity and the property of interest.^79,80^ Table S4 in the SI lists the ρ and *A*(*H*) values with uncertainty for all Hammett-Potential analyses presented here.

Beginning the **HFER** analysis with redox events, *A*(*L*), for example, is the formal reduction potential associated with a metal complex coordinated ligand (when the diffusion coefficients for the oxidized and reduced forms are assumed to be equal), and *A*(*H*) represents the reduction potential of the unsubstituted ligand. Figure 7a plots the reduction potentials of the complexes as a function of their Σ_σ_. The *E*_*p*,*a*_^°′^(*Ir* ^IV/III^) is plotted for the irreversible couple representing the compound’s HOMO, while the *E*_1/2_^°′^(*L*_π_^(0/–1)^) and *E*_1/2_^°′^(*L*_π_^(−1/–2)^) were used to plot the LUMO energy and second reduction, respectively. [Ir(**4-NMe**_**2**_ **PhTerpy**)(ppy)Cl](PF_6_) is excluded from the analysis because the low-lying amine-based redox couple distorts the energy of the metal-centered HOMO by virtue of increased charge after amine-oxidation. In each case, a strong correlation between the redox event and the ligand’s Σ_σ_ (*r* ^2^ = 0.96–0.99) exists (Table S4). Of these redox events, the redox couple associated with the LUMO (i.e., *E*_1/2_^°′^(*L*_π_^(0/–1)^)) is most strongly coupled to the π-acidity of the substituent (ρ = 0.103 V/Σ_σ_), which is nearly four times that of the redox couple corresponding to the HOMO (i.e., *E*_*p*,*a*_^°′^(*Ir* ^IV/III^); ρ = 0.023 V/Σ_σ_), and about twice as strongly coupled relative to the second ligand-centered reduction (ρ = 0.042 V/Σ_σ_). Additionally, a strong correlation holds when evaluating a plot of the difference between the HOMO–LUMO redox potentials (i.e., the electrochemically determined HOMO–LUMO gap, eHLG) as a function of **RPhTerpy** Σ_σ_ (*r* ^2^ = 0.99; with ρ = −0.080 V/Σ_σ_; Figure 7a).

First row transition metal-centered oxidations of homoleptic [M­(**RPhTerpy**)_2_]^2+^ complexes of Ni­(II), Mn­(II), Fe­(II), and Co­(II) display ρ ranging 0.037–0.057 V/Σ_σ_, with ρ ranging 0.06–0.1 V/Σ_σ_ for the first ligand-centered reductions.^19^ Comprehensive second and third-row transition metal studies have not been performed. However, *E* ^°′^(*Os* ^(III/II)^) = 0.11 V/Σ_σ_ (*r* ^2^ = 0.84) for the oxidation of [Os­(**RPhTerpy**)_2_]^2+^, when R in **RPhTerpy** = 4-OMe–, 4-Me–, 4-H–, 4-Br–, and 4-Cl–, and indicates a similar magnitude of electronic coupling to the current iridium architecture, while no correlation (*r* ^2^ = 0.05) is present for *E*_1/2_^°′^(*L*_π_^(0/–1)^) (Figure S1).^18^

Taken together, the data indicates that the aryl functional group imparts a (1) degree of electronic control over each of the metal- and ligand-centered redox couples evaluated, (2) that a great deal of control over the LUMO energy is afforded by the **RPhTerpy** substituents, and (3) that the *E*_*p*,*a*_^°′^(*Ir* ^IV/III^) may be purely inductive in nature, given the small ρ-value of 0.023 V/Σ_σ_, likely indicating that the ligand is nearly completely decoupled from the HOMO. Computational modeling generally supports these conclusions. Notably, the eHLG correlates well with both computational modeling of the lowest-energy transition (Δ*E*(LET); *r* ^2^ = 0.991) and *E*_00_ values (*r* ^2^ = 0.985; Figure S2).

Considering the strong electronic coupling between the substituent of the **RPhTerpy** ligand and the metal- and ligand-centered redox potentials, it is therefore reasonable to posit that the **HFER** analysis can provide quantitative characterization of other thermodynamic properties—such as photophysically measured and computationally calculated properties—to ultimately provide a comprehensive, robust, and inherently predictive analysis of emissive systems.

Performing a **HFER** analysis next with photophysical parameters, and again excluding [Ir­(**4-NMe**_**2**_ **PhTerpy**)­(ppy)­Cl]­(PF_6_), reveals a strong correlation exists for the absorption maxima of the MLCT bands and for emission at room temperature (*r* ^2^ = 0.93 and 0.91, respectively; Figure 7b). Unlike the absorption and emission wavelengths, *k_nr_* and τ*_em_* vs Σ_σ_ demonstrate a poor correlation (*r* ^2^ = 0.39 and 0.42, respectively; Figure S3), which is unsurprising considering that the complexes deviate from energy gap law. However, an excellent correlation exists between the *E*_00_ and Σ_σ_ (*r* ^2^ = 0.99; Figure 7b), indicating the potential utility in using the Hammett parameter to predictably design compounds with desired *E*_00_ values even when plagued by noncorrelating kinetic processes.

**Figure 7:**
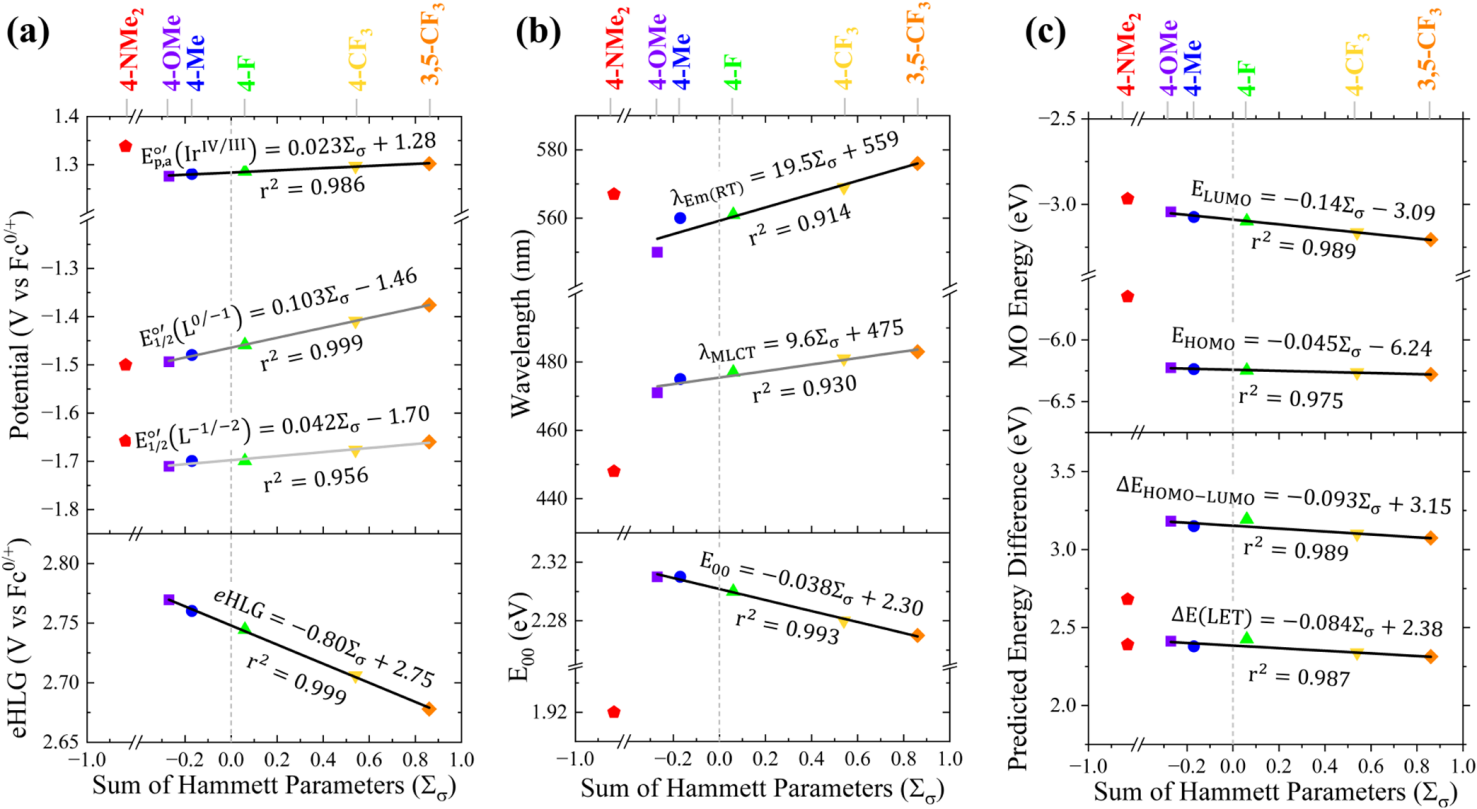
Hammett-Potential plots as a function of ligand Σ_σ_. The Σ_σ_ = 0 is depicted with a gray dashed line. The linear regressions and regression data are overlaid on the plots. (a) *E*_*p*,*a*_^°′^(*Ir* ^IV/III^), *E*_1/2_^°′^(*L*_π_^(0/–1)^), and *E*_1/2_^°′^(*L*_π_^(−1/–2)^) (top) and eHLG (*E*_*p*,*a*_^°′^(*Ir* ^IV/III^) – *E*_1/2_^°′^(*L*_π_^(0/–1)^); bottom); (b) absorbance maxima of the MLCT band and emission maxima at room temperature (top) and *E*_00_ (bottom); (c) computationally calculated HOMO and LUMO MO energies (top), computationally determined energy differences, Δ*E*_HOMO–LUMO_ (bottom), and Δ*E*(LET) (from TD-DFT lowest-energy transition; bottom).

Given the importance of controlling excited-state redox power in photocalytic processes, excited-state redox potential estimates for the series were generated using the observed electrochemical data and 77 K emission data according to eqs 3 and 4. While these ignore differences in *E*_00_ and emission maxima, as well as differences in spectroscopic and electrochemical analysis and the electrostatic work term, ω_*r*_, describing charge generation and separation within the electron-transfer complex, it is assumed that the difference between electrochemical and optical gaps is fairly constant and can be used to calculate potentials for relative trends within a series (Table S3).^29,81^

3 $$E\left({\mathrm{Ir}}^{*/-}\right)=E^{{}^{\circ}\prime }\left(L_{\pi }^{\left(0/-1\right)}\right)+E_{00}+\omega_{r}$$

4 $$E\left({\mathrm{Ir}}^{+/*}\right)=E_{p,a}^{{}^{\circ}\prime }\left({\mathrm{Ir}}^{\mathrm{IV}/\mathrm{III}}\right)-E_{00}+\omega_{r}$$

Hammett-Potential analysis of the estimated potentials indicates a strong correlation (*r* ^2^ = 0.98) between the estimated excited-state reduction potentials, *E*(Ir*^/–^), and Σ_σ_ but no correlation (*r* ^2^ = 0.20) with the estimated excited-state oxidation potential, *E*(*Ir* ^+/^*). It is not surprising that the values for *E*(*Ir* ^+/^*) change very little (*E*(*Ir* ^+/^*) = −1.01 ± 0.01 V), given the common ground state HOMO that the complexes possess. A large ρ value (ρ = 0.13 V/Σ_σ_) for *E*(Ir*^/–^) indicates that the strong functional group coupling observed with *E* ^°′^(*L*_π_^(0/–1)^) translates to the excited state as a result of significant MO density extending to these functional groups (see Figure 5), which is unsurprising as these estimates couple two thermodynamic measurements that also correlate strongly.

Continuing the **HFER** analysis with DFT modeling reveals that HOMO and LUMO energies strongly correlate (*r* ^2^ = 0.98 and 0.99, respectively; Figure 7c) to Σ_σ_, as do the energy differences in these orbitals (*r* ^2^ = 0.99; Figure 7c). However, these values overestimate the magnitude of the coupling as compared to experimental values (ρ­(Δ*E*_HOMO–LUMO_) = −0.093 eV/Σ_σ_ vs ρ­(*E*_00_) = −0.038 eV/Σ_σ_). Similar to the plots of *E*_*p*,*a*_^°′^(*Ir* ^IV/III^) and *E*_1/2_^°′^(*L*_π_^(0/–1)^), the LUMO (ρ = −0.14 eV/Σ_σ_) is more strongly coupled to substituent π-acidity than the HOMO (ρ = −0.045 eV/Σ_σ_). Likewise, the high-energy RPh– -dominated MOs for the series (identified by the purple dashed lines in Figures 5 and S47) demonstrate a very strong electronic coupling (ρ = −1.39 eV/Σ_σ_) and Hammett correlation (*r* ^2^ = 0.91; Figure S48).

Next, the TD-DFT calculated lowest-energy transition, Δ*E*(LET), correlates linearly with Σ_σ_ (ρ­(Δ*E*(LET)) = −0.084 eV/Σ_σ_; *r* ^2^ = 0.987) as the variation of Δ*E*(LET) (and Δ*E*_HOMO–LUMO_) across the series is solely a consequence of terpy ligand electron density altering the position of the LUMO, especially considering that HOMO energies remain largely intact across the series (see Figure 4). Again, these observations are true, with the exception of [Ir­(**4-NMe**_**2**_ **PhTerpy**)­(ppy)­Cl]­(PF_6_), where the HOMO is significantly higher energy (−5.65 eV) as compared to other complexes (−6.28 to −6.23 eV), and where its HOMO–1 more closely resembles the HOMO of the rest of the series, in both composition and energy.

Notably, [Ir­(**4-NMe**_**2**_ **PhTerpy**)­(ppy)­Cl]­(PF_6_) was excluded from the various **HFER** analyses due to its significant deviations from the trends observed with the remaining members of the series, and it serves as an important example of where Hammett analyses can fall short. Thus, the Hammett parameters reliably model physical and photophysical relationships, though this observation holds only when the key molecular orbital character is similar in nature.

## Discussion

Systematic studies evaluating the relationship between substituent Hammett values relative to the physical (i.e., redox potentials; eHLG) and photoluminescent (i.e., *E*_00_; *k*_*nr*_, excited-state redox potentials; DFT modeled HOMO–LUMO gap and lowest-energy transitions) properties of inorganic complexes are sparse, especially for iridium. Thus, direct comparison of the [Ir­(**RPhTerpy**)­(ppy)­Cl]­(PF_6_) series to others is complicated. However, some insight can be gleaned by applying the **HFER** analysis presented in this work to available data found in our literature survey of second- and third-row photoluminescent transition metal complexes. For example, Eisenberg reported that excited-state emission properties of platinum­(II) dithiolate complexes correlate strongly with the Hammett parameters of substituents at the 4- and 4′-position of 4,4′-R-2,2′-bipyridine (Table 2).^81^

**Table 2:** Table of Hammett-Property Analysis for Select Series Computed from Reported Values^a^

compound	*E*_ox_	*E*_red_	eHLG	*E*_00_	*E* ^(^*^/−)^	E*^/+^	Δ*E*_HOMO–LUMO_
Pt(4,4′-R-2,2′-bipyridine)(dithiolate)	0.01 V/Σ_σ_ + 0.39 V (0.02)	0.36 V/Σ_σ_ – 1.26 V (0.92)	–0.35 V/Σ_σ_ + 1.65 V (0.91)	–0.26 V/Σ_σ_ + 1.82 V (0.95)	0.10 V/Σ_σ_ + 0.55 V (0.63)	0.28 V/Σ_σ_ – 1.43 V (0.92)	not reported
[Ir(2,4-F-ppy)2(4,4′-R‴-bpy)](PF_6_)^82^	–0.35 V/Σ_σ_ + 1.53 V (0.99)	–0.07 V/Σ_σ_ – 1.07 V (0.63)	–0.28 V/Σ_σ_ + 2.60 V (0.98)	–0.24 V/Σ_σ_ + 2.36 V (0.96)	–0.31 V/Σ_σ_ + 1.29 V (0.91)	–0.11 V/Σ_σ_ – 0.83 V (0.89)	–0.38 V/Σ_σ_ + 3.29 V (0.99)
[Ir(2,4-F-ppy)_2_](RPhcarbene)^83^	0.18 V/Σ_σ_ + 0.55 V (0.97)	0.09 V/Σ_σ_ – 2.62 V (0.92)	0.09 V/Σ_σ_ + 3.17 V (0.90)	0.16 V/Σ_σ_ + 2.44 V (0.98)	0.24 V/Σ_σ_ – 0.18 V (0.98)	0.02 V/Σ_σ_ – 1.89 V (0.68)	0.09 V/Σ_σ_ + 3.85 V (0.88)
[(H_2_pbbzim)Ru(4′-R′PhTerpy)](PF_6_)_2_ ^20^	0.24 V/Σ_σ_ + 1.03 V (0.98)	0.02 V/Σ_σ_ – 1.48 V (0.48)	0.22 V/Σ_σ_ + 2.52 V (0.99)	–0.12 V/Σ_σ_ + 1.89 V (0.99)	0.35 V/Σ_σ_ – 0.84 V (0.98)	–0.10 V/Σ_σ_ + 0.41 V (0.99)	not reported
[Ir(**RPhTerpy**)(ppy)Cl](PF_6_)^γ^	0.023 V/Σ_σ_ + 1.284 V (0.98)	0.103 V/Σ_σ_ – 1.464 V (0.99)	–0.080 V/Σ_σ_ + 2.748 V (0.99)	–0.038 V/Σ_σ_ + 2.302 V (0.99)	0.07 V/Σ_σ_ + 0.84 V (0.99)	0.06 V/Σ_σ_ – 1.02 V (0.99)	–0.093 eV/Σ_σ_ + 3.153 eV (0.99)

a Each potential was treated analogous to the [Ir­(**RPhTerpy**)­(ppy)­Cl]­(PF_6_) according to eq 1. When two functional groups were present in a given complex, their σ_*p*_ or σ_*m*_ values were multiplied by 2 to give a per-functional group ρ-value. ^γ^—[Ir­(**4-NMe**_**2**_ **PhTerpy**)­(ppy)­Cl]­(PF_6_) is excluded from the analysis.

Similar to the observed correlation between *E*_00_ and Hammett parameters herein, Meyer previously demonstrated that the 77 K emission properties of bis­(2,2′-bipyridine)­ruthenium­(II) complexes correlate with Hammett parameters. These complexes take the form *cis*-[Ru­(bpy)_2_(κ^1^-L)­(X)]^*n*+^, where κ^1^-L are 4-substituted pyridines and X^–^ is the strong-field ligand, nitrite (*E*_00_ = 0.09 eV/Σ_σ_ + 1.91 eV; *r* ^2^ = 0.99), the weak-field ligand chloride (*E*_00_ = 0.10 eV/Σ_σ_ + 2.14 eV; *r* ^2^ = 0.99), or triphenylphosphine (*E*_00_ = 0.10 eV/Σ_σ_ + 2.28 eV; *r* ^2^ = 0.99) and allows for fine-tuning of emission energy with the X-substituent serving as handle for coarse tuning, while leaving the electronic coupling (i.e., ρ) largely intact.^84^

Processing Baitalik’s electrochemical and photophysical data evaluating [(H_2_pbbzim)­Ru­(4′-R′PhTerpy)]­(PF_6_)_2_ demonstrates that *E*_1/2_^°′^(*Ru* ^III/II^), *E*_00_, and estimated excited-state redox potentials correlate strongly with the ligand’s Hammett parameter (*r* ^2^ > 0.98; Table 2), while the ligand-centered reduction potentials remain unaltered throughout the series.^20^

Grätzel characterized a series of homologous biscyclometalated Ir­(III) complexes Ir­(2,4-di-X-phenyl-pyridine)_2_(pic),^85^ where X = H–, F–, Cl–, and Br–, and pic = picolinate. While the published data did not lend itself to treatment identical to our evaluation, orbital energies (experimental and computational) and emission maxima correlate well with ligand Hammett parameters. Baranoff later extended this study to develop a more robust Hammett-Potential model for [Ir­(R″-ppy)_2_(acac)], [Ir­(R″-ppy)_2_(pic)], and [Pt­(R″-ppy)­(acac)] complexes, with acac = acetyl acetonate and R″-ppy = substituted 2-(phenyl)­pyridine.^86^ Presumably, Baranoff’s multivariable model can be extended to photoluminescent parameters as well. Evaluation of Skórka’s electrochemical and photophysical data with iridium cycylometalated complexes appended with bipyridines of the form [Ir­(ppy)_2_(4,4′-R‴-bpy)]­(PF_6_) and [Ir­(2,4-F-ppy)_2_(4,4-R‴-bpy)]­(PF_6_), where 2,4-F-ppy is 2-(2,4-difluorophenyl)­pyridine, indeed demonstrate strong correlations between the Hammett parameter and redox potentials, eHLG, *E*_00_, DFT predicted HOMO–LUMO gap, and estimated excited-state redox potentials (Table 2).^82^ It is noteworthy that the electronic communication, as indicated by ρ, is largely preserved when either ppy or 2,4-F-ppy complexes are evaluated. This observation suggests that the ppy ligands can be viewed as scaling factors akin to the X^–^ substituents in Meyer’s work. Similar behavior is observed with phenyl, bis-meta-substituted imidazolylidene complexes of the form [Ir­(2,4-F-ppy)_2_]­(RPhcarbene) (Table 2).^83^

Strategies for tuning iridium photophysical properties usually involve varying ligands without regard to systematic functional group analysis,^87^ again limiting direct comparison to the [Ir­(**RPhTerpy**)­(ppy)­Cl]­(PF_6_) series. However, the magnitude of electronic coupling (i.e., ρ) is significantly less for the [Ir­(**RPhTerpy**)­(ppy)­Cl]­(PF_6_) in this report than the above examined cases. For example, ρ­(*E*_00_) and ρ­(eHLG) are 23–36% and 15–33% less than the examined series, while ρ­(*E* ^(^*^/−)^) and ρ­(*E* ^+/^*) are attenuated by 20–70% and 21–60%. This observed attenuation relative to the bpy derivatives is not surprising because substituents are directly connected to the coordinated bipyridine, while electronic communication between the substituents and coordinated terpyridine ligands reported herein is disrupted by a phenyl spacer. Likewise, the majority of the ρ-values are larger for the ruthenium system with 4′R′PhTerpy than for the [Ir­(**RPhTerpy**)­(ppy)­Cl]­(PF_6_) complexes. However, the π-acidity of the [Os­(**RPhTerpy**)_2_]^2+^ complex’s ligand is coupled significantly stronger to its oxidation potential than to its reduction, which is opposite of that observed here; stronger coupling to osmium’s oxidation potential is indicative that the terpyridine ligand’s substituents are modulating the energy of the HOMO, rather than the LUMO as demonstrated here. The small ρ­(*E*_00_) for Ru and [Ir­(**RPhTerpy**)­(ppy)­Cl]­(PF_6_) indicates that the range with which the *E*_00_ can be tuned by **RPhTerpy** is narrow, but this offers fine-tuning capabilities.

## Conclusions

A comprehensive set of **HFER** analyses were performed to ascertain the utility of using Hammett parameters as a means to characterize and quantify the electronic coupling between functional groups and an array of ground- and excited-state thermodynamic parameters in emissive compounds. A strong correlation between the ligands’ Hammett parameters and several experimental and theoretical properties is revealed when using the [Ir­(**RPhTerpy**)­(ppy)­Cl]­(PF_6_) series reported here as a test case. In summary, a strong correlation is present between Σ_σ_ and the:

- metal-centered oxidation potentials (*E*_*p*,*a*_^°′^(*Ir* ^IV/III^); e.g. the ground state HOMO).
- ligand-centered reduction potentials (*E*_1/2_^°^′^^(*L*_π_^(0/–1)^); e.g. the ground state LUMO).
- MLCT absorption maxima, room-temperature emission maxima, and *E*_00_.
- estimated excited-state reduction potentials (*E*(Ir*^/–^)).
- DFT calculated HOMO–LUMO orbital energy differences (Δ*E*_HOMO–LUMO_)
- TD-DFT calculated lowest-energy transitions (Δ*E*(LET)).

While ω_*r*_, *k*_*nr*_, and emission decay do not correlate well to Σ_σ_, the strong correlation between Hammett and various thermodynamic parameters found in the [Ir­(**RPhTerpy**)­(ppy)­Cl]­(PF_6_) series largely extends to physical and photophysical features observed in other cases of functionalized bipyridine, phenanthroline, and cyclometalated series of platinum, ruthenium, and iridium complexes. The correlation is particularly apparent when we apply the comprehensive **HFER** analysis approach presented in this work to the available data of second- and third-row photoluminescent transition metal complexes.

Furthermore, physical, photophysical, and computational analyses suggest that using the [Ir­(N^N^N)­(C^N)­(X)]^+^ architecture effectively decouples the HOMO and LUMO, localizing them on individual moieties (HOMO on ppy; LUMO on **RPhTerpy**), and suggests that each may be independently adjusted to offer a strategy for high-resolution tuning capability in future systems, which is contrary to homoleptic bis-terpyridine metal complexes.

Taken together, this work implies that Hammett parameter analysis is generally applicable to chromophores and offers a means to predict and systematically tune ground- and excited-state photoluminescent properties beyond those evaluated here. This work demonstrates how powerful Hammett analysis can be when designing chromophores for multiple applications, from OLED (due to correlation with emissive maxima) to photocatalysis (due to strong correlation with photoredox potentials). Thus, Hammett analysis is a quantitative tool that may be employed in every synthetic organic and inorganic photochemist’s toolbox and is superior to alternative qualitative approaches.

## Experimental Section

### Instrumentation

Absorption spectra were collected with a Shimadzu 2700 UV–visible spectrometer. Solution samples were prepared by dissolving complexes in acetonitrile in a 1 cm × 1 cm quartz cuvette. NMR spectra were collected on a JEOL JNM-ECZ400S-CON 400 MHz, 9.4 T Spectrometer. All ^1^H and ^13^C­{^1^H} chemical shifts were referenced to residual solvent signals: 2.50 and 39.52 ppm for DMSO-*d*_6_.^88^ ^19^F resonances were determined by using PF_6_^–^ as an internal standard (−68.3 ppm, *J*_PF_ = 711 Hz), which was determined by inserting a sealed melting point capillary tube filled with trifluoroacetic acid (−76.55 ppm) into an NMR tube. A CEM Discover 2.0 Microwave Reactor was used for microwave synthesis. Infrared spectra were collected in absorbance mode on a Nicolet iS50 FTIR spectrometer using the built-in ATR attachment (64 scans, 0.482 cm^–1^ steps, background subtracted).

### Electrochemical Analysis

Cyclic voltammetry experiments were performed in a 3-compartment glass cell separated by medium- or fine-porosity frits. A glassy carbon electrode was suspended in the center compartment with a septum containing 2 predrilled holes: one for the GC electrode and one for the degassing tube. In one outer compartment, a reference electrode was suspended in a septum containing 2 predrilled holes: one for the reference electrode and one for a degassing tube. In the remaining outer compartment, platinum wire or gauze was threaded through a septum adjacent to a degassing tube and served as the counter electrode. Prior to use, the cells were rinsed with MeCN, and the appropriate solutions were placed in their respective compartments. The analysis solutions were freed of oxygen by vigorously degassing them with N_2_, for a minimum of 5 min that had flowed through gas washers containing MeCN. The 3 mm diameter GC electrodes, purchased from CH Instruments, were polished for ∼10 s with 0.3 μm alumina polish, on a polishing pad, and rinsed with water followed by MeCN. Prior to performing electrochemical experiments, the Ag wire reference electrode solution was replaced with a stock solution of 10 mM AgNO_3_ in 0.1 M TBAPF_6_ MeCN. All iridium complexes are free of impurities via CV analysis. Electrochemical measurements using the 10 mM AgNO_3_ were conducted a CH Instruments 760E potentiostat and referenced internally vs Fc^+/0^.

### X-ray Diffraction

Single crystals for X-ray structure determination of [Ir­(**3,5-CF**_**3**_ **PhTerpy**)­(ppy)­Cl]­(PF_6_), [Ir­(**4-CF**_**3**_ **PhTerpy**)­(ppy)­Cl]­(PF_6_), [Ir­(**4-MePhTerpy**)­(ppy)­Cl]­(PF_6_), [Ir­(**4-OMePhTerpy**)­(ppy)­Cl]­(PF_6_), and [Ir­(**4-NMe**_**2**_ **PhTerpy**)­(ppy)­Cl]­(PF_6_) were grown via slow diffusion of diethyl ether into test tubes containing a solution of the complexes in acetonitrile or acetone. Care was taken to ensure that the samples were not exposed to light by placing them in an amber bottle and in a cabinet. X-ray intensity data were collected at 170 K using graphite-monochromated Mo Kα radiation (λ = 0.71073 Å) on a Rigaku XtaLAB Mini II diffractometer with an Oxford Cryosystems Cryostream 800 and accompanying Oxford AD61 Dry Air Unit. Data collection and reduction were done using Rigaku’s CrysAlisPro, including absorption corrections by the analytical numeric method.^89^ Olex2 1.5 ^90^ was used for structure solutions through intrinsic phasing with ShelXT^91^ and refined with ShelXL,^92^ while full-matrix least-squares minimization was conducted with ShelX.^92^ Heavily disordered solvent molecules were removed by the SQUEEZE/PLATON technique.^93^ The H atoms were positioned geometrically and treated as riding atoms during subsequent refinement. The methyl groups were allowed to rotate about their local 3-fold axes. Crystal data and detailed experimental conditions are given in the Supporting Information. Comparison of select bond lengths and angles is shown in Table S10. ORTEP-3 was used to write POV-Ray files for figure production.^94^

### Steady-State Emission

Steady-state emission spectra were collected on a fluorescence spectrometer (Edinburgh FS5). The samples were excited at 500 nm using the light from a 150 W Xe lamp, and the emission was detected by a visible Hamamatsu photomultiplier tube. Spectra were processed with emission correction files on the Edinburgh Fluoracle software package (v. 2.9.0.4). Room-temperature solution samples were prepared in freeze–pump–thaw degassed acetonitrile in a 1 cm × 1 cm quartz cuvette. Emission spectra were collected in triplicate with a 1 nm step size and a dwell time of 0.5 s. Samples at 77 K were prepared in 4:5 propionitrile/butyronitrile (4:5 prop/but) in an NMR tube and placed into a liquid nitrogen dewar. Twenty emission spectra were collected and averaged for each sample with a step size of 1 nm and a dwell time of 0.1 s.

### Emission Quantum Yield

Emission quantum yields of complexes in freeze–pump–thaw degassed acetonitrile were measured relative to a standard of Rhodamine 6G in ethanol (Φ = 0.95).^95^ Absorbance values at 500 nm were prepared to 0.05–0.08 O.D. Samples were excited at 500 nm with a slit width of 2.25 nm, and emission was collected with a slit width of 1 nm. Three separate photoluminescence spectra were collected from 510 to 900 nm (step size 1 nm, dwell time 0.5 s) for each sample. The integrated emission intensity was calculated using the Edinburgh Fluoracle software package (v. 2.9.0.4). Relative quantum yields were calculated using eq 5, where S = sample; R = reference; Φ = quantum yield; *I* = integrated emission intensity (510–900 nm); *A* = absorbance at 500 nm; and η = refractive index of the solvent.^96,97^

5 $$\Phi_{S}=\Phi_{R}\left(\frac{I_{S}}{I_{R}}\right)\left(\frac{1-10^{-A_{R}}}{1-10^{-A_{S}}}\right){\left(\frac{\eta_{S}}{\eta_{R}}\right)}^{2}$$

### Time-Resolved Emission

Time-resolved emission measurements were recorded at room temperature on the same fluorescence spectrometer. The samples were excited by a pulsed diode laser (operating at 510 ± 10 nm, having a pulse width 85 ps) with a repetition rate of 50 kHz (Edinburgh EPL-510 nm). Emission decay traces were acquired using time-correlated single photon counting (TCSPC; 1024 channels; 20 μs window) with data collection for 5000 counts. The decay traces were fit with a monoexponential tail fit using the Edinburgh Fluoracle software package (v. 2.9.0.4). Weighted biexponential fits were calculated using eq 6 to determine the weighting factors and eq 7 to calculate the weighted lifetime (τ_*w*_), where Φ = weighting factor, *A* = initial value, τ = emission decay time.

6 $$\Phi_{1}=\frac{A_{1}\tau_{1}}{A_{1}\tau_{1}+A_{2}\tau_{2}}$$

7 $$\tau_{w}=\frac{\Phi_{1}\tau_{1}^{2}+\Phi_{2}\tau_{2}^{2}}{\Phi_{1}\tau_{1}+\Phi_{2}\tau_{2}}$$

### Mass Spectrometry

#### LDI-TOF and MALDI-TOF

Mass spectra were acquired using a Shimadzu Biotech Axima Confidence instrument equipped with a 337 nm N_2_ laser for matrix-assisted laser desorption ionization (MALDI-TOF) and laser desorption/ionization-time of flight (LDI-TOF), in the method of Lou.^44^ The instrument was calibrated using the ProteoMass Peptide MALDI-MS Calibration Kit 2 purchased from Sigma-Aldrich following the external standard procedures provided. LDI samples were prepared by dissolving 2 mg of sample with 1 mL of THF in an Eppendorf tube. Roughly 0.5–0.8 μL of the samples was pipetted onto the MALDI target plate and allowed to evaporate. Samples were prepared for MALDI-TOF by dissolving 20 mg of the 2,5-dihydroxybenzoic acid (DHB) matrix to a fresh Eppendorf tube with 1 mL of THF. The samples were then mixed with matrix solution in a 1:1 ratio and pipetted onto the MALDI target plate and allowed to evaporate. Mass spectra were acquired in linear-mode (positive) by averaging spectra from 500 profiles, where each profile was a sum of 5 shots using a range of power level of 38–40, depending on the sample analyzed, while MALDI utilized a power level of 55.

#### ESI-MS

Approximately 0.5 mg of iridium complex was dissolved in approximately 0.5 mL of acetonitrile and filtered with a Thompson vial and analyzed with a JEOL AccuTOF-CS instrument in ESI+ mode.

### Materials

Anhydrous acetonitrile was obtained from ACROS Organics (extradry, Acroseal) or Millipore Sigma. Tetra-*n*-butylammonium hexafluorophosphate was recrystallized twice from ethanol (∼3 mL of ethanol was used per gram of electrolyte), and the needles were collected on a fritted funnel and air-dried by pulling a vacuum through the collection for several hours. Iridium­(III) chloride trihydrate was obtained from Pressure Chemical and used as received. Other solvents and chemicals were used as received from Fisher Scientific.

### Synthetic Details

#### Additional Synthetic Detail Narrative

Synthesis of complexes of the general form [Ir­(**L**)­(ppy)­Cl]­(PF_6_), where **L** is a meridional κ^3^-N^N^N ligand, has been accomplished by refluxing a solution of Ir­(**L**)­Cl_3_ and 2-phenylpyridine (ppy) in ethylene glycol (e.g.) overnight in good yield,^2,9,24–26,42^ while the Ir­(**L**)­Cl_3_ precursors are obtained rapidly by stirring the ligand and IrCl_3_·3H_2_O in a small volume of e.g. in a test tube submerged in a 170–180 °C oil bath for 15–30 min and isolated by filtration.^2,9,26,42,43^ Modifying these reaction conditions for a more rapid synthesis of the target [Ir­(**RPhTerpy**)­(ppy)­Cl]­(PF_6_) complexes is desirable, especially considering the adoption of high-throughput capability of pressurized reactions and shorter reaction times afforded by microwave-assisted reaction technology. Therefore, a solution of Ir­(**RPhTerpy**)­Cl_3_ and a slight excess of ppy in small volumes (∼3 mL) of e.g. were heated to 240 °C and held for 15 min, over which time, the Ir­(**RPhTerpy**)­Cl_3_ dissolved and orange solutions formed. These solutions are strongly emissive upon irradiation with a 365 nm flashlight to qualitatively indicate the formation of a new emissive species. The solutions were transferred to an Erlenmeyer flask and diluted with water (25 mL), and then precipitated by the addition of several equivalents of ammonium hexafluorophosphate (NH_4_PF_6_). These flocculent orange solids were collected, redissolved in acetone, and reprecipitated with diethyl ether in yields ranging from 72 to 92% (Figure 2). For convenience, the syntheses of Ir­(**RPhTerpy**)­Cl_3_ were performed in a microwave vessel similar to published test tube procedures in yields ranging from 45 to 71%.

#### Ligand Synthesis

All **RPhTerpy** ligands were prepared according to previously reported Krönke pyridine synthetic procedures: 6-(pyridin-2-yl)-4-[4-(trifluoromethyl)­phenyl]-2,2′-bipyridine, **4-CF**_**3**_ **PhTerpy**;^19^ 4-[3,5-bis­(trifluoromethyl)­phenyl]-6-(pyridin-2-yl)-2,2′-bipyridine, **3,5-CF**_**3**_ **PhTerpy**;^19^ (4-(4-methoxyphenyl)-6-(pyridin-2-yl)-2,2′-bipyridine), **4-OMePhTerpy**;^19^ (4-[4-methylphenyl]-6-(pyridin-2-yl)-2,2′-bipyridine), **4-MePhTerpy**;^35^ 4-[4-fluorophenyl]-6-(pyridin-2-yl)-2,2′-bipyridine, **4-FPhTerpy**;^36^ 4-[4-(*N,N*-dimethylamino)­phenyl]-6-(pyridin-2-yl)-2,2′-bipyridine, **4-NMe**_**2**_ **PhTerpy**.^37^

#### General Ir­(**RPhTerpy**)­Cl_3_ Synthesis

Ir­(**L**)­Cl_3_ complexes have been previously synthesized by oil bath heating, and these procedures were modified for microwave-assisted heating.^43^ A volume of ethylene glycol (e.g.) was added to a 30 mL microwave reaction vessel containing a stirbar. Taking care to prevent reagents from sticking to the upper portion of the reaction vessel, the **RPhTerpy** was then suspended in the center reaction vial, followed by IrCl_3_·3H_2_O. Next, a second portion of e.g. was added, and the solution was carefully prestirred and then placed in the microwave queue. The mixture was then heated to 75 °C and held for 90 s, then to 125 °C and held for 90 s, and finally to 165 °C and held for 15 min. The reaction was allowed to cool and settle for at least a half an hour, but higher yields were cooled overnight. The dark reaction solution was then decanted through a 15–30 mL medium porosity fritted funnel. The remaining solid was transferred with absolute ethanol to the frit and rinsed with 2 × 15 mL portions of ethanol, followed by 2 × 15 mL portions of diethyl ether. Red to dark red, microcrystalline solids of Ir­(**RPhTerpy**)­Cl_3_ were isolated following vacuum air drying on the funnel and used without further purification or characterization.

Ir­(**3,5-CF**_**3**_ **PhTerpy**)­Cl_3_. Ethylene glycol (5 mL); **3,5-CF**_**3**_ **PhTerpy** (0.445 g, 1.00 mmol); IrCl_3_·3H_2_O (0.352 g, 1.00 mmol); ethylene glycol (5 mL). Red microcrystalline solid (0.339 g, 1.00 mmol, 44.9% yield, C_23_H_13_Cl_3_F_6_IrN_3_).

Ir­(**4-CF**_**3**_ **PhTerpy**)­Cl_3_. Ethylene glycol (5 mL); **4-CF**_**3**_ **PhTerpy** (0.377 g, 1.00 mmol); IrCl_3_·3H_2_O (0.352 g, 1.00 mmol); ethylene glycol (5 mL). Red microcrystalline solid (0.444 g, 0.657 mmol, 65.9% yield, C_22_H_14_Cl_3_F_3_IrN_3_).

Ir­(**4-FPhTerpy**)­Cl_3_.^26^ Ethylene glycol (5 mL); **4-FPhTerpy** (0.327 g, 1.00 mmol); IrCl_3_·3H_2_O (0.352 g, 1.00 mmol); ethylene glycol (5 mL). Red microcrystalline solid (0.445 g, 0.711 mmol, 71.1% yield, C_21_H_14_Cl_3_FIrN_3_).

Ir­(**4-MePhTerpy**)­Cl_3_.^26^ Ethylene glycol (5 mL); **4-MePhTerpy** (0.323 g, 1.00 mmol); IrCl_3_·3H_2_O (0.352 g, 1.00 mmol); ethylene glycol (5 mL). Red microcrystalline solid (0.435 g, 0.699 mmol, 69.9% yield, C_22_H_17_Cl_3_IrN_3_).

Ir­(**4-OMePhTerpy**)­Cl_3_. Ethylene glycol (10 mL); **4-OMePhTerpy** (0.679 g, 2.00 mol); IrCl_3_·3H_2_O (0.705 g, 2.00 mmol); ethylene glycol (10 mL). The reaction was performed 4 times, and the red microcrystalline solids were combined (3.500 g, 5.49 mmol, 68.6% yield, C_22_H_17_Cl_3_IrN_3_ *O*).

Ir­(**4-NMe**_**2**_ **PhTerpy**)­Cl_3_. Ethylene glycol (5 mL); **4-NMe**_**2**_ **PhTerpy** (0.352 g, 1.00 mmol); IrCl_3_·3H_2_O (0.352 g, 1.00 mmol); ethylene glycol (5 mL). Red microcrystalline solid (0.451 g, 0.693 mmol, 69.3% yield, C_23_H_20_Cl_3_IrN_4_).

#### General [Ir­(**RPhTerpy**)­(ppy)­Cl]­(PF_6_) Synthesis

An atmospheric pressure synthesis of a related compound, [Ir­(**RPhTerpy**)­(ppy)­Cl]­(PF_6_), served as a model procedure and was modified to decrease the reaction under microwave conditions.^2,9,42^ A slight excess of ppy was added to a tared 10 mL microwave reaction vessel containing a stirbar, followed by the addition of Ir­(**RPhTerpy**)­Cl_3_ and e.g. The solutions were prestirred and then placed in the microwave queue. The mixture was then heated to 240 °C and held for 15 min. These often generated homogeneous solutions, except for the **4-FPhTerpy** derivative. Upon cooling, the reaction solution was diluted with 25 mL of water. In some cases, a murky solution formed but dissolved upon the addition of 5–10 mL of acetone. If any particulates were noted, the solution was filtered through Celite. Next, 5–10 equiv of ammonium hexafluorophosphate were dissolved in water and slowly added to the reaction solution to precipitate orange solids. This solid was collected on a 15 mL medium porosity fritted funnel. The material was then redissolved in a minimal amount of acetone (5–20 mL) and then slowly diluted with diethyl ether to precipitate flocculent solids that were again collected on a 15 mL medium porosity fritted funnel, washed with 2 × 15 mL of diethyl ether, and air-dried. The reported yield represents that these powders were of good purity via NMR. All materials were recrystallized by dissolving them in acetonitrile, or acetone, and performing vapor–vapor diffusion in an amber bottle containing diethyl ether. All characterization data was collected using recrystallized material. ^1^H spectra of [Ir­(**4-MePhTerpy**)­(ppy)­Cl]­(PF_6_) and [Ir­(**4-FPhTerpy**)­(ppy)­Cl]­(PF_6_) are consistent with the previously published spectra,^26^ and ^13^C data is included here. A suite of 2D NMR (^1^H–^1^H DQF-COSY, ^1^H–^1^H NOESY, ^1^H–^1^H TOCSY, ^1^H–^13^C HSQC-TOCSY, ^1^H–^13^C HSQC, ^1^H–^13^C HSQC-TOCSY, and ^1^H–^13^C HMBC) data was collected on each compound and allowed for unambiguous assignment of ^1^H and ^13^C. See manuscript Figure 2 for atom assignments.

[Ir­(**3,5-CF**_**3**_ **PhTerpy**)­(ppy)­Cl]­(PF_6_). Hppy (0.047 g, 0.31 mmol, 1.3 equiv); Ir­(**3,5-CF**_**3**_ **PhTerpy**)­Cl_3_ (0.175 g, 0.235 mmol); e.g. (3.53 g). Deep red-purple solid (0.176 g, 0.181 mmol, 77% yield). ^1^H NMR (400 MHz, DMSO-*d*_6_) δ 9.92 (d, *J* = 5.9 Hz, 1H, H9_*ppy*_), 9.40 (s, 2H, H3′), 9.01–8.98 (m, 4H, H2‴+H3), 8.53 (d, *J* = 8.3 Hz, 1H, H6_*ppy*_), 8.44 (s, 1H, H4‴), 8.29 (m, 3H, H4+H7_*ppy*_), 7.97 (d, *J* = 7.9 Hz, 1H, H5_*ppy*_), 7.84 (ddd, *J* = 7.3, 5.9, 1.5 Hz, 1H, H8_*ppy*_), 7.74 (d, *J* = 5.7 Hz, 2H, H6), 7.59 (ddd, *J* = 7.1, 5.6, 1.4 Hz, 2H, H5), 6.95 (t, *J* = 7.9 Hz, 1H, H4_*ppy*_), 6.76 (t, *J* = 7.9 Hz, 1H, H3_*ppy*_), 6.10 d, *J* = 7.9 Hz, 1H, H2_*ppy*_. ^13^C­{^1^H} NMR (101 MHz, DMSO-*d*_6_) δ 165.38 (C6a_*ppy*_), 157.69 (C2), 155.39 (C2′), 151.84 (C6), 150.42 (C9_*ppy*_), 148.11 (C4′), 143.76 (C5a_*ppy*_), 141.18 (C1_*ppy*_), 140.34 + 140.19 (C4+C7_*ppy*_), 138.22 (C1‴), 131.29 (q, ^2^ *J*_CF_ = 33.2 Hz, C3‴), 130.32 + 130.19 (C2_*ppy*_+C3_*ppy*_), 129.36 (br, C2‴), 129.27 (C5), 126.25 (C3), 124.58 (C8_*ppy*_), 124.24 (br, C4‴), 123.96 (C4_*ppy*_), 123.40 (q, ^1^ *J*_CF_ = 273.0 Hz, −CF_3_), 122.76 (C3′), 120.84 (C6_*ppy*_). ^19^F (376 MHz, DMSO-*d*_6_) δ −59.1 (s, 6F, −CF_3_). ESI-MS obs’d (rel. % intensity), calc’d (rel. % intensity), ppm diff for [C_34_H_21_ClF_12_IrN_4_P – PF_6_]^+^: 825.0973 (49), 825.0978 (49), 0.6; 826.0995 (18), 826.0995 (19), 0; 827.0964 (100), 827.0979 (100), 1.8; 828.1022 (36), 828.0997 (37), 3.0; 829.0973 (28), 829.0981 (32), 1.0; 830.1004 (10), 830.0999 (11), 0.6.

[Ir­(**4-CF**_**3**_ **PhTerpy**)­(ppy)­Cl]­(PF_6_). Hppy (0.045 g, 0.29 mmol, 1.3 equiv); Ir­(**4-CF**_**3**_ **PhTerpy**)­Cl_3_ (0.151 g, 0.223 mmol); e.g. (3.50 g). Red solid (0.170 g, 0.188 mmol, 85% yield). ^1^H NMR (400 MHz, DMSO-*d*_6_) δ 9.90 (d, *J* = 5.8 Hz, 1H, H9_*ppy*_), 9.36 (s, 2H, H3′), 8.99 (d, *J* = 8.1 Hz, 2H, H3), 8.54 (m, *J* = 8.5 Hz, 3H, H6_*ppy*_+H2‴), 8.31 (*J* = 8.0 Hz, 1H, H7_*ppy*_), 8.27 (dd, *J* = 8.1, 1.5 Hz, 2H, H4), 8.15 (d, *J* = 8.2 Hz, 2H, H3‴), 7.97 (d, *J* = 8.0 Hz, 1H, H5_*ppy*_), 7.84 (ddd, *J* = 7.3, 5.8, 1.4 Hz, 1H, H8_*ppy*_), 7.73 (d, *J* = 5.8 Hz, 2H, H6) 7.58 (dt, *J* = 8.1, 1.4 Hz, 2H, H5), 6.94 (t, *J* = 7.7 Hz, 1H, H4_*ppy*_), 6.77 (td, *J* = 7.5, 1.3 Hz, 1H, H3_*ppy*_), 6.10 (d, *J* = 7.6 Hz, 1H, H2_*ppy*_). ^13^C­{^1^H} NMR (101 MHz, DMSO-*d*_6_) δ 165.38 (C6a_*ppy*_), 157.76 (C2), 155.42 (C2′), 151.80 (C6), 150.44 (C9_*ppy*_), 149.71 (C4′), 143.75 (C5a_*ppy*_), 141.18 (C1_*ppy*_), 140.31 + 140.25 (C7_*ppy*_+C4), 139.41 (C1‴), 130.88 (q, ^2^ *J*_CF_ = 31.0 Hz, C4‴), 130.37 + 130.22 (C2_*ppy*_+C3_*ppy*_), 129.21 + 126.19 (C3+C3‴), 125.26 (C5_*ppy*_), 124.55­(C8_*ppy*_), 123.92 (C4_*ppy*_), 123.60 (q, ^1^ *J*_CF_ = 273.0 Hz, −CF_3_), 122.37 (C3^′^), 120.82 (C6_*ppy*_). ^19^F (376 MHz, DMSO-*d*_6_) δ −59.3 (s, 3F, −CF_3_). ESI-MS obs’d (rel. % intensity), calc’d (rel. % intensity), ppm diff for [C_33_H_22_ClF_9_IrN_4_P – PF_6_]^+^: 757.1089 (49), 757.1094 (49), 0.7; 758.1144 (18), 758.1131 (18), 1.7; 759.1103 (100), 759.1108 (100), 0.7; 760.1134 (35), 760.1146 (36), 1.6; 761.1101 (29), 761.1093 (32), 1.1; 762.1132 (10), 762.113 (10), 0.3.

[Ir­(**4-FPhTerpy**)­(ppy)­Cl]­(PF_6_). Hppy (0.047 g, 0.30 mmol, 1.3 equiv); Ir­(**4-FPhTerpy**)­Cl_3_ (0.149 g, 0.238 mmol); e.g. (3.50 g). Red solid 0.148 g, 0.173 mmol, 72% yield.^26^ ^1^H NMR (400 MHz, DMSO-*d*_6_) δ 9.90 (dd, *J* = 6.2, 1.5 Hz, 1H, H9_*ppy*_), 9.28 (s, 2H, H3′), 8.97 (d, *J* = 8.3 Hz, 2H, H3), 8.52 (d, *J* = 8.4 Hz, 1H, H6), 8.41 (dd, *J* = 8.8 Hz, ^3^ *J*_HF_ = 5.4 Hz, 2H, H2‴), 8.30 (td, *J* = 7.8, 1.6 Hz, 1H, H7_*ppy*_), 8.25 (td, *J* = 7.8, 1.5 Hz, 2H, H4), 7.96 (dd, *J* = 8.0, 1.4 Hz, 1H, H5_*ppy*_), 7.83 (ddd, *J* = 7.3, 5.8, 1.4 Hz, 1H, H8_*ppy*_), 7.71 (dd, *J* = 5.7, 1.4 Hz, 2H, H6), 7.63 (t, ^2^ *J*_HF_, *J* = 8.8 Hz, 2H, H3‴), 7.56 (ddd, *J* = 7.3, 5.6, 1.4 Hz, 2H, H5), 6.94 (td, *J* = 7.5, 1.2 Hz, 1H, H4_*ppy*_), 6.76 (td, *J* = 7.5, 1.4 Hz, 1H, H3_*ppy*_), 6.08 (dd, *J* = 7.7, 1.1 Hz, 1H, H2_*ppy*_). ^13^C­{^1^H} NMR (101 MHz, DMSO-*d*_6_) δ 165.42 (C6a_*ppy*_), 164.14 (d, ^1^ *J*_CF_ = 249.5 Hz, C4‴), 157.89 (C2), 155.24 (C2^′^), 151.76 (C6), 150.45 (C4^′^), 150.30 (C9_*ppy*_), 143.77 (C5a_*ppy*_), 141.23 (C1_*ppy*_), 140.26 + 140.19 (C4+C7_*ppy*_), 131.83 (d, ^4^ *J*_CF_ = 3.0 Hz, C1‴), 130.78 (d, ^3^ *J*_CF_ = 8.9 Hz, C2‴), 130.37 + 130.18 (C2_*ppy*_+C3_*ppy*_), 129.11 (C5), 126.06 (C3), 125.24 (C5_*ppy*_), 124.53 (C8_*ppy*_), 123.87 (C4_*ppy*_), 121.64 (C3′), 120.79 (C6_*ppy*_), 116.42 (d, ^2^ *J*_CF_ = 21.9 Hz, C3‴). ^19^F (376 MHz, DMSO-*d*_6_) δ −108.1 (tt, ^2^ *J*_HF_ = 8.8 Hz, ^3^ *J*_HF_ = 5.4 Hz, 1F). ESI-MS obs’d (rel. % intensity), calc’d (rel. % intensity), ppm diff for [C_32_H_22_ClF_7_IrN_4_P – PF_6_]^+^: 707.1123 (50), 707.1113 (49), 1.4; 708.1153 (17), 708.1165 (18), 1.7; 709.1142 (100), 709.1132 (100), 1.4; 710.1194 (32), 710.1183 (35), 1.5; 711.114 (28), 711.1121 (32), 2.7; 712.115 (10), 712.1145 (10), 0.7.

[Ir­(**4-MePhTerpy**)­(ppy)­Cl]­(PF_6_). Hppy (0.049 g, 0.32 mmol, 1.3 equiv); Ir­(**4-MePhTerpy**)­Cl_3_ (0.151 g, 0.242 mmol); e.g. (3.50 g). Red solid (0.183 g, 0.215 mmol, 89% yield).^26^ ^1^H NMR (400 MHz, DMSO-*d*_6_) δ 9.90 (d, *J* = 5.2 Hz, 1H, H9_*ppy*_), 9.28 (s, 2H, H3′), 9.00 (d, *J* = 7.6 Hz, 2H, H3), 8.53 (d, *J* = 8.2 Hz, 1H, H6_*ppy*_), 8.38–8.15 (m, 5H, H7_*ppy*_+H4+H2‴), 7.97 (dd, *J* = 7.9, 1.4 Hz, 1H, H5_*ppy*_), 7.83 (ddd, *J* = 7.3, 5.8, 1.4 Hz, 1H, H8_*ppy*_), 7.71 (dd, *J* = 5.8, 1.5 Hz, 2H, H6), 7.56 (ddd, *J* = 6.9, 5.7, 1.3 Hz, 4H, H5+H3‴), 6.94 (td, *J* = 7.5, 1.2 Hz, 1H, H4_*ppy*_), 6.77 (td, *J* = 7.5, 1.3 Hz, 1H, H3_*ppy*_), 6.09 (dd, *J* = 7.8, 1.2 Hz, 1H, H2_*ppy*_), 2.50 (s, 3H, −CH_3_). ^13^C­{^1^H} NMR (101 MHz, DMSO-*d*_6_) δ 165.45 (C6a_*ppy*_), 157.97 (C2), 155.23 (C2′), 151.76 (C6), 151.30 (C4′), 150.48 (C9_*ppy*_), 143.81 (C5a_*ppy*_), 141.42 + 141.29 (C4‴+C1_*ppy*_), 140.26 + 140.18 (C4+C7_*ppy*_), 132.31 (C1‴), 130.40 + 130.21 (C2_*ppy*_+C3_*ppy*_), 130.01 + 129.08 (C5+C3‴), 128.13 (C2‴), 126.07 (C3), 125.25 (C5_*ppy*_), 124.55 (C8_*ppy*_), 123.87 (C4_*ppy*_), 121.21 (C3^′^), 120.80 (C6_*ppy*_), 21.02 (−CH_3_). ESI-MS obs’d (rel. % intensity), calc’d (rel. % intensity), ppm diff for [C_33_H_25_ClF_6_IrN_4_P – PF_6_]^+^: 703.1393 (50), 703.1364 (49), 4.1; 704.1424 (19), 704.1415 (18), 1.3; 705.1393 (100), 705.1382 (100), 1.6; 706.1424 (37), 706.1433 (36), 1.3; 707.1368 (29), 707.1371 (32), 0.4; 708.1424 (10), 708.1394 (10), 4.2.

[Ir­(**4-OMePhTerpy**)­(ppy)­Cl]­(PF_6_). Hppy (0.047 g, 0.31 mmol, 1.3 equiv); Ir­(**4-OMePhTerpy**)­Cl_3_ (0.150 g, 0.225 mmol); e.g. (3.53 g). Dark red-purple solid (0.182 g, 0.210 mmol, 89% yield). ^1^H NMR (400 MHz, DMSO-*d*_6_) δ 9.90 (d, *J* = 4.7 Hz, 1H, H9_*ppy*_), 9.24 (s, 2H, H3′), 8.98 (dd, *J* = 8.2, 1.3 Hz, 2H, H3), 8.51 (d, *J* = 8.2 Hz, 1H, H6_*ppy*_), 8.34 (d, *J* = 8.9 Hz, 2H, H2‴), 8.29 (td, *J* = 7.8, 1.6 Hz, 1H, H7_*ppy*_), 8.24 (td, *J* = 7.9, 1.5 Hz, 2H, H4), 7.96 (dd, *J* = 7.9, 1.5 Hz, 1H, H5_*ppy*_), 7.82 (ddd, *J* = 7.3, 5.8, 1.4 Hz, 1H, H8_*ppy*_), 7.70 (dd, *J* = 5.7, 1.4 Hz, 2H, H6), 7.55 (ddd, *J* = 7.3, 5.6, 1.4 Hz, 2H, H5), 7.29 (d, *J* = 8.9 Hz, 2H, H3‴), 6.93 (td, *J* = 7.6, 1.2 Hz, 1H H4_*ppy*_), 6.77 (td, *J* = 7.4, 1.4 Hz, 1H, H3_*ppy*_), 6.09 (dd, *J* = 7.7, 1.2 Hz, 1H, H2), 3.94 (s, 3H, –OCH_3_). ^13^C­{^1^H} NMR (101 MHz, DMSO-*d*_6_) δ 165.48 (C6a_*ppy*_), 161.98 (C4‴), 158.04 (C2), 155.10 (C2′), 151.72 (C6), 151.05 (C4′), 150.48 (C9_*ppy*_), 143.81 (C5a_*ppy*_), 141.31 (C1_*ppy*_), 140.23 + 140.13 (C7_*ppy*_+C4), 130.38 + 130.18 (C2_*ppy*_+C3_*ppy*_), 129.89 (C2‴), 129.01 (C5), 127.32 (C1‴), 125.99 (C3), 125.23 (C5_*ppy*_), 124.52 (C8_*ppy*_), 123.85 (C4_*ppy*_), 120.77 (C6_*ppy*_), 120.69 (C3^′^), 114.84 (C3‴), 55.66 (−OCH_3_). ESI-MS obs’d (rel. % intensity), calc’d (rel. % intensity), ppm diff for [C_33_H_25_ClF_6_IrN_4_OP – PF_6_]^+^: 719.1309 (49), 719.1311 (49), 0.3; 720.1349 (17), 720.1361 (18), 1.7; 721.1327 (100), 721.1325 (100), 0.3; 722.1411 (34), 722.1376 (37), 4.8; 723.1345 (28), 723.134 (32), 0.7; 724.1358 (9), 724.1361 (11), 0.4.

[Ir­(**4-NMe**_**2**_ **PhTerpy**)­(ppy)­Cl]­(PF_6_). Hppy (0.043 g, 0.28 mmol, 1.2 equiv); Ir­(**4-NMe**_**2**_ **PhTerpy**)­Cl_3_ (0.151 g, 0.232 mmol); e.g. (3.50 g). Deep red solid (0.187 g, 0.213 mmol, 92% yield). ^1^H NMR (400 MHz, DMSO-*d*_6_) δ 9.90 (d, *J* = 6.0 Hz, 1H, H9_*ppy*_), 9.15 (s, 2H, H3′), 8.96 (d, *J* = 7.6 Hz, 2H, H3), 8.50 (dd, *J* = 8.4, 1.3 Hz, 1H, H6_*ppy*_), 8.32–8.16 (m, 5H, H7+H2‴+H4), 7.95 (dd, *J* = 7.9, 1.5 Hz, 1H, H5_*ppy*_), 7.82 (ddd, *J* = 7.4, 5.8, 1.4 Hz, 1H, H8_*ppy*_), 7.68 (d, *J* = 5.0 Hz, 2H, H6), 7.52 (ddd, *J* = 7.3, 5.6, 1.4 Hz, 2H, H5), 6.96 (d, *J* = 9.0 Hz, 2H, H3‴), 6.93 (td, *J* = 7.4, 1.3 Hz, 1H, H4_*ppy*_) 6.77 (td, *J* = 7.4, 1.3 Hz, 1H, H3_*ppy*_), 6.10 (dd, *J* = 7.6, 1.1 Hz, 1H, H2_*ppy*_), 3.12 (s, 6H, –N­(CH_3_)_2_). ^13^C­{^1^H} NMR (101 MHz, DMSO-*d*_6_) δ 165.54 (C6a_*ppy*_), 158.27 (C2), 154.74 (C2′), 152.36 (C4‴), 151.66 (C6), 151.34 (C4′), 150.50 (C9_*ppy*_), 143.86 (C5a_*ppy*_), 141.43 (C1_*ppy*_), 140.14 + 139.99 (C7_*ppy*_+C4), 130.37 + 130.17 (C2_*ppy*_+C3_*ppy*_), 129.25 (C2‴), 128.81 (C5), 125.77 (C3), 125.20 (C5_*ppy*_), 124.48 (C8_*ppy*_), 123.77 (C4_*ppy*_), 121.19 (C1‴), 120.73 (C6_*ppy*_), 119.01 (C3′), 112.06 (C3‴), 39.77 (–N­(CH_3_)_2_). ESI-MS obs’d (rel. % intensity), calc’d (rel. % intensity), ppm diff for [C_34_H_28_ClF_6_IrN_5_P – PF_6_]^+^: 732.1619 (52), 732.1633 (49), 1.9; 733.1676 (19), 733.1661 (19), 2.0; 734.1663 (100), 734.166 (100), 0.4; 735.1667 (36), 735.1688 (38), 2.9; 736.1636 (30), 736.1657 (32), 2.9; 737.1659 (11), 737.1656 (11), 0.4.

## Supplementary Material





## Notes

CV experimental reduction potential tables; ^1^H, ^13^C, ^19^F NMR data; 2D NMR data (DQF-COSY, TOCSY, NOESY, edited-HSQC, HSQC-TOCSY, HMBC); photophysical data (UV–vis, TCSPC, FCLSA); MS data; IR data; computational data tables; TD-DFT-predicted UV–vis spectra; DFT *xyz* structures; data tables used in preparing Table 1; ORTEP diagrams; and crystallographic data (PDF)
G16 computational *XYZ* coordinates (ZIP)
