# Supplementary material for: High-Resolution Tuning of Iridium(III) 4′-Aryl-terpy Chromophores: A Hammett Parameter-Guided General Methodology for Systematic Property Control through Orbital Decoupling
Source: Inorg Chem. 2026 Feb 10;65(7):3829–45. doi: 10.1021/acs.inorgchem.5c04650 (PMC12933883; doi:10.1021/acs.inorgchem.5c04650)
Supplement: Supplementary file 1 [file ic5c04650_si_001.pdf]

High-Resolution Tuning of Iridium(III) 4'-Aryl-Terpy Chromophores: A Hammett Parameter-Guided General Methodology for Systematic Property Control through Orbital Decoupling

Erica S. Knorr,[†] Jordan C. Kelly,[†] Ryan B. Gaynor,[†,‡] Thomas N. Rohrabaugh, Jr.,[†] Caleb A. Brown,[¶] and Daniel P. Harrison[*,¶]

†*U.S. Army Combat Capabilities Development Command Army Research Laboratory, 2800 Powder Mill Rd, Adelphi, MD 20783, United States*

‡*Department of Chemical and Biological Science and Engineering, United States Military Academy, West Point, New York 10996, United States*

¶*Department of Chemistry, Virginia Military Institute, 401 Maury-Brooke Hall, Lexington, VA 24450, United States*

E-mail: harrisondp@vmi.edu

Contents

1 Electrochemistry **S8**

1.1 Reduction Potential Table S8

1.2 [Os(**RPhTerpy**)$_2$]$^{2+}$ Electrochemical Data S9

2 Estimated Excited State Redox Potentials **S10**

3 Hammett-Potential Analysis **S11**

3.1 Hammett-Potential Analysis of the [Ir(**RPhTerpy**)(ppy)Cl](PF_6) Series . . S11

3.2 Hammett-Potential Analysis of Referenced Work S13

4 NMR Spectra **S15**

4.1 Overlay of ^{1}H NMR Spectra S16

4.2 NMR Data for [Ir(**3,5-CF$_3$PhTerpy**)(ppy)Cl](PF_6) S17

4.3 NMR Data for [Ir(**4-CF$_3$PhTerpy**)(ppy)Cl](PF_6) S23

4.4 NMR Data for [Ir(**4-FPhTerpy**)(ppy)Cl](PF_6) S29

4.5 NMR Data for [Ir(**4-MePhTerpy**)(ppy)Cl](PF_6) S35

4.6 NMR Data for [Ir(**4-OMePhTerpy**)(ppy)Cl](PF_6) S41

4.7 NMR Data for [Ir(**4-NMe$_2$PhTerpy**)(ppy)Cl](PF_6) S47

5 Crystallographic Data **S53**

5.1 Additional Crystallography Narrative S53

5.2 [Ir(**3,5-CF$_3$PhTerpy**)(ppy)Cl](PF_6) S54

5.3 [Ir(**4-CF$_3$PhTerpy**)(ppy)Cl](PF_6) S56

5.4 [Ir(**4-MePhTerpy**)(ppy)Cl](PF_6) S58

5.5 [Ir(**4-OMePhTerpy**)(ppy)Cl](PF_6) S60

5.6 [Ir(**4-NMe$_2$PhTerpy**)(ppy)Cl](PF_6) S62

6 Infrared Spectra **S64**

7 Computational Details **S65**

7.1 Computational Nomenclature Key S65

7.2 Example Input Title Card for Optimization and Frequency Check Calculation S66

7.3 Example Input Title Card for TD DFT Calculation S67

7.4 def2-svp text and values S69

7.5 IR-ECP text and values . . . S70

7.6 Computational Data Table . . . S71

7.7 Computational Structure Metric Comparison to Crystallographic Data . . . S72

7.8 MO Composition . . . S73

7.8.1 RPhTerpy Energy-Composition Diagram . . . S73

7.8.2 Percent Composition Tables for Select MOs . . . S75

7.9 TD DFT Predicted and Experimental UV-Vis Spectra Overlays . . . S77

7.10 XYZ coordinates of Optimized Structures . . . S84

8 Photophysical Data **S120**

8.1 Extinction Coefficients . . . S120

8.2 Franck-Condon Line Shape Analysis . . . S124

8.3 Miscellaneous Photophysical Data . . . S128

9 Mass Spectra **S130**

References **S143**

List of Supplemental Figures

Figure S1 Hammett-Potential plot of [Os(**RPhTerpy**)$_2$]$^{2+}$ series . . . S9

Figure S2 HOMO-LUMO comparison . . . S11

Figure S3 Hammett Photophysics . . . S11

Figure S4 Stacked ^{1}H NMR of [Ir(**RPhTerpy**)(ppy)Cl](PF$_6$) . . . S16

Figure S5 NMRs of [Ir(**3,5-CF$_3$PhTerpy**)(ppy)Cl](PF$_6$) . . . S17

Figure S6 NMRs of [Ir(**3,5-CF$_3$PhTerpy**)(ppy)Cl](PF$_6$) (continued) . . . S18

Figure S7 NMRs of [Ir(**3,5-CF$_3$PhTerpy**)(ppy)Cl](PF$_6$) (continued) . . . S19

Figure S8 NMRs of [Ir(**3,5-CF$_3$PhTerpy**)(ppy)Cl](PF$_6$) (continued) . . . S20

Figure S9 NMRs of [Ir(**3,5-CF$_3$PhTerpy**)(ppy)Cl](PF$_6$) (continued) . . . S21

Figure S10 NMRs of [Ir(**3,5-CF$_3$PhTerpy**)(ppy)Cl](PF$_6$) (continued) S22

Figure S11 NMRs of [Ir(**4-CF$_3$PhTerpy**)(ppy)Cl](PF$_6$) S23

Figure S12 NMRs of [Ir(**4-CF$_3$PhTerpy**)(ppy)Cl](PF$_6$) (continued) S24

Figure S13 NMRs of [Ir(**4-CF$_3$PhTerpy**)(ppy)Cl](PF$_6$) (continued) S25

Figure S14 NMRs of [Ir(**4-CF$_3$PhTerpy**)(ppy)Cl](PF$_6$) (continued) S26

Figure S15 NMRs of [Ir(**4-CF$_3$PhTerpy**)(ppy)Cl](PF$_6$) (continued) S27

Figure S16 NMRs of [Ir(**4-CF$_3$PhTerpy**)(ppy)Cl](PF$_6$) (continued) S28

Figure S17 NMRs of [Ir(**4-FPhTerpy**)(ppy)Cl](PF$_6$) S29

Figure S18 NMRs of [Ir(**4-FPhTerpy**)(ppy)Cl](PF$_6$) (continued) S30

Figure S19 NMRs of [Ir(**4-FPhTerpy**)(ppy)Cl](PF$_6$) (continued) S31

Figure S20 NMRs of [Ir(**4-FPhTerpy**)(ppy)Cl](PF$_6$) (continued) S32

Figure S21 NMRs of [Ir(**4-FPhTerpy**)(ppy)Cl](PF$_6$) (continued) S33

Figure S22 NMRs of [Ir(**4-FPhTerpy**)(ppy)Cl](PF$_6$) (continued) S34

Figure S23 NMRs of [Ir(**4-MePhTerpy**)(ppy)Cl](PF$_6$) S35

Figure S24 NMRs of [Ir(**4-MePhTerpy**)(ppy)Cl](PF$_6$) (continued) S36

Figure S25 NMRs of [Ir(**4-MePhTerpy**)(ppy)Cl](PF$_6$) (continued) S37

Figure S26 NMRs of [Ir(**4-MePhTerpy**)(ppy)Cl](PF$_6$) (continued) S38

Figure S27 NMRs of [Ir(**4-MePhTerpy**)(ppy)Cl](PF$_6$) (continued) S39

Figure S28 NMRs of [Ir(**4-MePhTerpy**)(ppy)Cl](PF$_6$) (continued) S40

Figure S29 NMRs of [Ir(**4-OMePhTerpy**)(ppy)Cl](PF$_6$) S41

Figure S30 NMRs of [Ir(**4-OMePhTerpy**)(ppy)Cl](PF$_6$) (continued) S42

Figure S31 NMRs of [Ir(**4-OMePhTerpy**)(ppy)Cl](PF$_6$) (continued) S43

Figure S32 NMRs of [Ir(**4-OMePhTerpy**)(ppy)Cl](PF$_6$) (continued) S44

Figure S33 NMRs of [Ir(**4-OMePhTerpy**)(ppy)Cl](PF$_6$) (continued) S45

Figure S34 NMRs of [Ir(**4-OMePhTerpy**)(ppy)Cl](PF$_6$) (continued) S46

Figure S35 NMRs of [Ir(**4-NMe$_2$PhTerpy**)(ppy)Cl](PF$_6$) S47

Figure S36 NMRs of [Ir(**4-NMe$_2$PhTerpy**)(ppy)Cl](PF$_6$) (continued) S48

Figure S37 NMRs of [Ir(**4-NMe$_2$PhTerpy**)(ppy)Cl](PF$_6$) (continued) S49

Figure S38 NMRs of [Ir(**4-NMe$_2$PhTerpy**)(ppy)Cl](PF$_6$) (continued) S50

Figure S39 NMRs of [Ir(**4-NMe$_2$PhTerpy**)(ppy)Cl](PF$_6$) (continued) S51

Figure S40 NMRs of [Ir(**4-NMe$_2$PhTerpy**)(ppy)Cl](PF$_6$) (continued) S52

Figure S41 ORTEP projection of [Ir(**3,5-CF$_3$PhTerpy**)(ppy)Cl](PF$_6$) S55

Figure S42 ORTEP projection of [Ir(**4-CF$_3$PhTerpy**)(ppy)Cl](PF$_6$) S57

Figure S43 ORTEP projection of [Ir(**4-MePhTerpy**)(ppy)Cl](PF$_6$) S59

Figure S44 ORTEP projection of [Ir(**4-OMePhTerpy**)(ppy)Cl](PF$_6$) S61

Figure S45 ORTEP projection of [Ir(**4-NMe$_2$PhTerpy**)(ppy)Cl](PF$_6$) S63

Figure S46 IR Spectra of [Ir(**RPhTerpy**)(ppy)Cl](PF$_6$) S64

Figure S47 RPh-Dominated MO Energy-Composition Diagram S73

Figure S48 RPh-Dominated MO Energy Plot S74

Figure S49 Experimental and TD-DFT UV-Vis of [Ir(**3,5-CF$_3$PhTerpy**)(ppy)Cl](PF$_6$)S78

Figure S50 Experimental and TD-DFT UV-Vis of [Ir(**4-CF$_3$PhTerpy**)(ppy)Cl](PF$_6$)S79

Figure S51 Experimental and TD-DFT UV-Vis of [Ir(**4-FPhTerpy**)(ppy)Cl](PF$_6$)S80

Figure S52 Experimental and TD-DFT UV-Vis of [Ir(**4-MePhTerpy**)(ppy)Cl](PF$_6$)S81

Figure S53 Experimental and TD-DFT UV-Vis of [Ir(**4-OMePhTerpy**)(ppy)Cl](PF$_6$)S82

Figure S54 Experimental and TD-DFT UV-Vis of [Ir(**4-NMe$_2$PhTerpy**)(ppy)Cl](PF$_6$)S83

Figure S55 UV-Vis Spectrum of [Ir(**4-OMePhTerpy**)(ppy)Cl](PF$_6$) S120

Figure S56 UV-Vis Spectrum of [Ir(**4-MePhTerpy**)(ppy)Cl](PF$_6$) S121

Figure S57 UV-Vis Spectrum of [Ir(**4-FPhTerpy**)(ppy)Cl](PF$_6$) S121

Figure S58 UV-Vis Spectrum of [Ir(**4-CF$_3$PhTerpy**)(ppy)Cl](PF$_6$) S122

Figure S59 UV-Vis Spectrum of [Ir(**3,5-CF$_3$PhTerpy**)(ppy)Cl](PF$_6$) S122

Figure S60 UV-Vis Spectrum of [Ir(**4-NMe$_2$PhTerpy**)(ppy)Cl](PF$_6$) S123

Figure S61 FCLSA of [Ir(**4-OMePhTerpy**)(ppy)Cl](PF$_6$) S124

Figure S62 FCLSA of [Ir(**4-MePhTerpy**)(ppy)Cl](PF$_6$) S124

Figure S63 FCLSA of [Ir(**4-FPhTerpy**)(ppy)Cl](PF$_6$) S125

Figure S64 FCLSA of [Ir(**4-CF_3PhTerpy**)(ppy)Cl](PF_6) S125

Figure S65 FCLSA of [Ir(**3,5-CF_3PhTerpy**)(ppy)Cl](PF_6) S126

Figure S66 FCLSA of [Ir(**4-NMe_2PhTerpy**)(ppy)Cl](PF_6) S126

Figure S67 Energy Gap Law S128

Figure S68 Absorbance and Emission (77 K) spectra S129

Figure S69 Absorbance and emission spectra from quantum yield. S129

Figure S70 MALDI-MS of [Ir(**4-OMePhTerpy**)(ppy)Cl](PF_6) S131

Figure S71 MALDI-MS of [Ir(**4-MePhTerpy**)(ppy)Cl](PF_6) S132

Figure S72 MALDI-MS of [Ir(**4-FPhTerpy**)(ppy)Cl](PF_6) S133

Figure S73 MALDI-MS of [Ir(**4-CF_3PhTerpy**)(ppy)Cl](PF_6) S134

Figure S74 MALDI-MS of [Ir(**3,5-CF_3PhTerpy**)(ppy)Cl](PF_6) S135

Figure S75 MALDI-MS of [Ir(**4-NMe_2PhTerpy**)(ppy)Cl](PF_6) S136

Figure S76 ESI-MS of [Ir(**4-OMePhTerpy**)(ppy)Cl](PF_6) S137

Figure S77 ESI-MS of [Ir(**4-MePhTerpy**)(ppy)Cl](PF_6) S138

Figure S78 ESI-MS of [Ir(**4-FPhTerpy**)(ppy)Cl](PF_6) S139

Figure S79 ESI-MS of [Ir(**4-CF_3PhTerpy**)(ppy)Cl](PF_6) S140

Figure S80 ESI-MS of [Ir(**3,5-CF_3PhTerpy**)(ppy)Cl](PF_6) S141

Figure S81 ESI-MS of [Ir(**4-NMe_2PhTerpy**)(ppy)Cl](PF_6) S142

List of Supplemental Tables

Table S1 Reduction potential Table S8

Table S2 Data Table for [Os(**RPhTerpy**)$_2$]$^{2+}$. S9

Table S3 Estimated Excited State Potentials for [Ir(**RPhTerpy**)(ppy)Cl](PF_6) S10

Table S4 Hammett Potential Analyses Data Table S12

Table S5 Data Table for platinum(II) dithiolate complexes. S13

Table S6 Data Table for [(H_2pbbzim)Ru(4′-R′PhTerpy)](PF_6)$_2$. S13

Table S7 Data Table for [Ir(ppy)$_2$(4,4′-R‴-bpy)](PF_6) S13

Table S8 Data Table for $[Ir(ppy)_2(4,4'\text{-}R'''\text{-}bpy)](PF_6)$ S14

Table S9 Data Table for $[Ir(2,4\text{-}F\text{-}ppy)_2(RPhcarbene)]$ S14

Table S10 XRD Metric Literature Comparison S53

Table S11 XRD Data for [Ir(**3,5-CF$_3$PhTerpy**)(ppy)Cl](PF_6) S54

Table S12 XRD Data for [Ir(**4-CF$_3$PhTerpy**)(ppy)Cl](PF_6) S56

Table S13 XRD Data for [Ir(**4-MePhTerpy**)(ppy)Cl](PF_6) S58

Table S14 XRD Data for [Ir(**4-OMePhTerpy**)(ppy)Cl](PF_6) S60

Table S15 XRD Data for [Ir(**4-NMe$_2$PhTerpy**)(ppy)Cl](PF_6) S62

Table S16 Computational Energy Data Table S71

Table S17 SC-XRD Bond Length Metrics for Terpyridine Moiety S72

Table S18 SC-XRD Bond Length Metrics for Ppy Moiety S72

Table S19 Table of RPh-Dominated MO's and Energy S74

Table S20 MO Composition table for [Ir(**3,5-CF$_3$PhTerpy**)(ppy)Cl]$^+$ S75

Table S21 MO Composition table for [Ir(**4-CF$_3$PhTerpy**)(ppy)Cl]$^+$ S75

Table S22 MO Composition table for [Ir(**4-FPhTerpy**)(ppy)Cl]$^+$ S75

Table S23 MO Composition table for [Ir(**4-MePhTerpy**)(ppy)Cl]$^+$ S76

Table S24 MO Composition table for [Ir(**4-OMePhTerpy**)(ppy)Cl]$^+$ S76

Table S25 MO Composition table for [Ir(**4-NMe$_2$PhTerpy**)(ppy)Cl]$^+$ S76

Table S26 FCLSA Parameters in Glass S127

Table S27 FCLSA Parameters in Acetonitrile S127

1 Electrochemistry

1.1 Reduction Potential Table

Table S1: Table of reduction potentials collected at 100 mv/s scan rate in 0.1 M $TBAPF_6$ in acetontrile under a nitrogen atmosphere. All values are in volts referenced to $Fc^{+/0}$as an internal standard. The values in parentheses indicate the peak-to-peak, ΔE_p, separation between anodic and cathodic half-waves. ΔE(HOMO-LUMO) is the voltage difference between the $E^{\circ\prime}_{p,a}(Ir^{IV/III})$ and $E^{\circ\prime}_{1/2}(L_\pi^{0/-1})$ couple.

**Complex**	Σ_σ	$E^{\circ\prime}_{p,a}(Ir^{IV/III})$	$E^{\circ\prime}_{1/2}(L_\pi^{0/-1})$	$E^{\circ\prime}_{1/2}(L_\pi^{-1/-2})$	$E^{\circ\prime}_{1/2}(NMe_2^{+/0})$	**eHLG**
[Ir(**3,5-CF$_3$PhTerpy**)(ppy)Cl](PF_6)	0.86	1.302	-1.376 (75)	-1.660 (147)	n/a	2.770
[Ir(**4-CF$_3$PhTerpy**)(ppy)Cl](PF_6)	0.54	1.297	-1.409 (83)	-1.676 (147)	n/a	2.761
[Ir(**4-FPhTerpy**)(ppy)Cl](PF_6)	0.06	1.286	-1.459 (79)	-1.699 (145)	n/a	2.745
[Ir(**4-MePhTerpy**)(ppy)Cl](PF_6)	-0.17	1.281	-1.480 (77)	-1.699 (142)	n/a	2.706
[Ir(**4-OMePhTerpy**)(ppy)Cl](PF_6)	-0.27	1.276	-1.494 (79)	-1.710 (112)	n/a	2.678
[Ir(**4-NMe$_2$PhTerpy**)(ppy)Cl](PF_6)	-0.83	1.338[a]	-1.500 (68)	-1.658 (101)	0.597 (67)	n/a

1.2 $[Os(RPhTerpy)_2]^{2+}$ Electrochemical Data

Table S2: Electrochemical data used to generate the Hammett-Potential plot for the described $[Os(\mathbf{RPhTerpy})_2]^{2+}$ series.[1] [a] - σ_p is multiplied by two here to account for the presence of two substituted-terpyridines per metal center. Hence, the plot provides a per functional group slope (ρ value) shown in Figure S1.

Complex	$\sigma_p \times 2$	Σ_σ [a]	$E^{\circ\prime}_{p,a}(Os^{III/II})$	$E^{\circ\prime}_{1/2}(L_\pi^{(0/-1)})$
$[Os(\mathbf{4\text{-}OMePhTerpy})_2]^{2+}$	-0.27	-0.54	0.47	-1.66
$[Os(\mathbf{4\text{-}MePhTerpy})_2]^{2+}$	-0.16	-0.32	0.47	-1.59
$[Os(\mathbf{4\text{-}HPhTerpy})_2]^{2+}$	0	0	0.52	-1.583
$[Os(\mathbf{4\text{-}ClPhTerpy})_2]^{2+}$	0.23	0.46	0.545	-1.566
$[Os(\mathbf{4\text{-}BrPhTerpy})_2]^{2+}$	0.23	0.46	0.59	-1.62

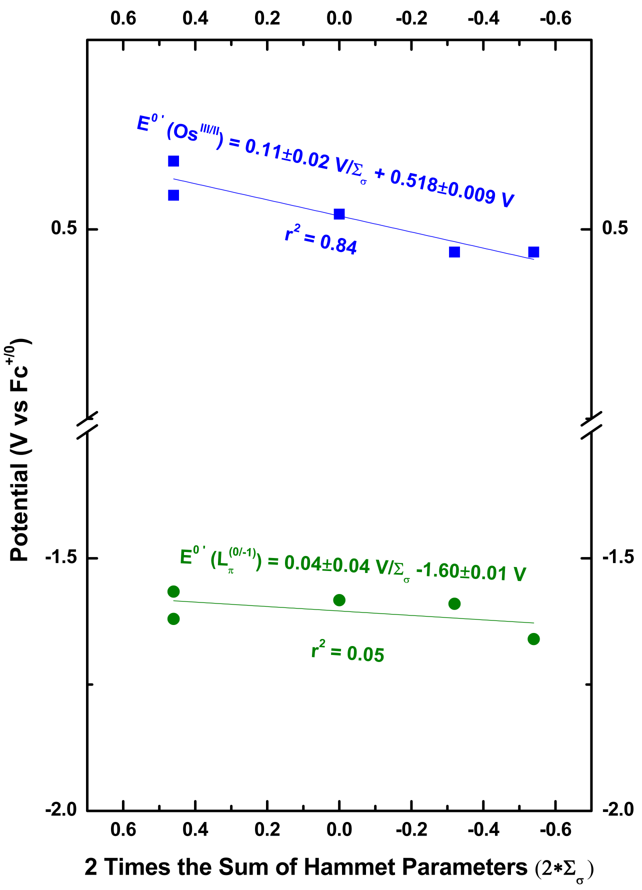

Figure S1: Hammett-Potential plot of $[Os(\mathbf{RPhTerpy})_2]^{2+}$ series generated from literature data.[1] Σ_σ is multiplied by two here to account for the presence of two Terpy's per metal center. Hence, the plot provides a per functional group slope (ρ value). While there is a good correlation between metal-centered redox potential and $2^*\Sigma_\sigma$, there is no correlation between it and the ligand-centered redox potential.

2 Estimated Excited State Redox Potentials

Table S 3: Estimated excited state oxidation and reduction potentials for the [Ir(**RPhTerpy**)(ppy)Cl](PF_6) series.

**Complex**	$E(Ir^{*/-})$	$E(Ir^{+/*})$
[Ir(**4-OMePhTerpy**)(ppy)Cl](PF_6)	0.78	-0.99
[Ir(**4-MePhTerpy**)(ppy)Cl](PF_6)	0.80	-1.00
[Ir(**4-FPhTerpy**)(ppy)Cl](PF_6)	0.84	-1.01
[Ir(**4-CF$_3$PhTerpy**)(ppy)Cl](PF_6)	0.90	-1.01
[Ir(**3,5-CF$_3$PhTerpy**)(ppy)Cl](PF_6)	0.93	-1.01

3 Hammett-Potential Analysis

3.1 Hammett-Potential Analysis of the [Ir(RPhTerpy)(ppy)Cl](PF_6) Series

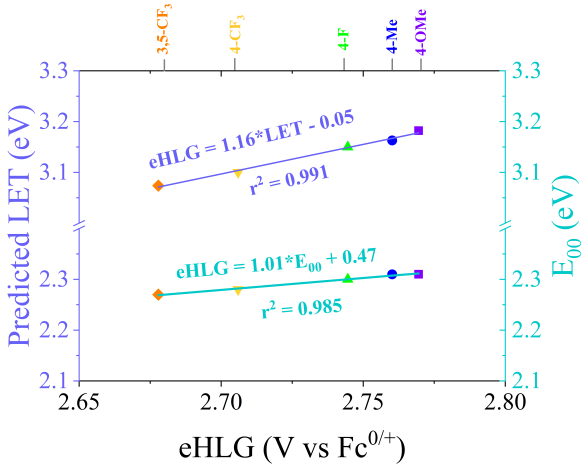

Figure S2: TD DFT predicted lowest energy transition (LET) and E_{00} correlations with the electrochemically-calculated HOMO-LUMO gap (eHLG). Linear regression fits are overlaid.

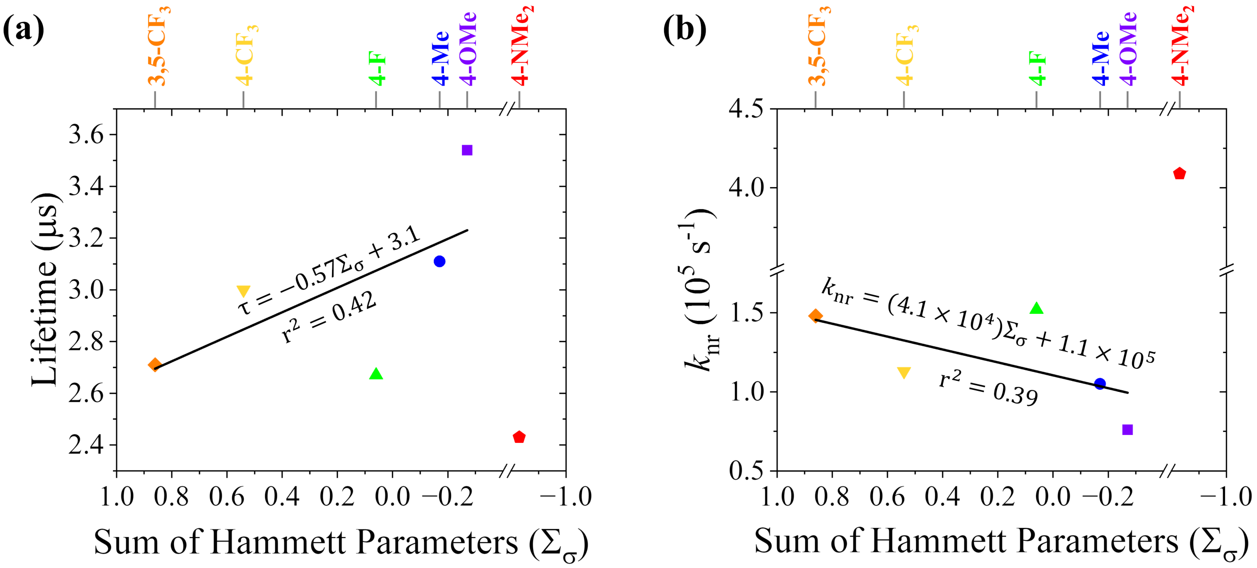

Figure S3: Hammett analysis plots for (a) emission decay times and (b) rate constants of non-radiative decay. Linear regression fits are overlaid.

Table S 4: Table of ρ and $A(H)$ values with uncertainty and linear regression fit. eHLG = electrochemically determined HOMO-LUMO gap ($E^{\circ\prime}_{p,a}(Ir^{IV/III})$-$E^{\circ\prime}(L_{\pi}^{(0/-1)})$); E_{HOMO} = computationally generated HOMO energy; E_{LUMO} = computationally generated LUMO energy; $\Delta E_{HOMO-LUMO}$ = difference in DFT generated HOMO and LUMO energies; ΔE_{LET} = TD DFT calculated lowest energy transition.

**Parameter**	ρ	**error in** ρ	**A(H)**	**error in A(H)**	r^2
$E^{\circ\prime}_{p,a}(Ir^{IV/III})$ (V vs $Fc^{+/0}$)	0.023	0.002	1.284	0.001	0.986
$E^{\circ\prime}_{1/2}(L_{\pi}^{(0/-1)})$ (V vs $Fc^{+/0}$)	0.103	0.002	-1.464	0.001	0.999
$E^{\circ\prime}_{1/2}(L_{\pi}^{(-1/-2)})$ (V vs $Fc^{+/0}$)	0.042	0.004	-1.698	0.002	0.956
eHLG (eV)	-0.080	0.002	2.748	0.0007	0.999
λ_{Abs} (nm)	10	2	475.5	0.7	0.930
λ_{Em} (RT; nm)	20	3	559	2	0.914
E_{00} (77K; eV)	-0.038	0.002	2.302	0.001	0.993
E_{HOMO} (eV)	-0.045	0.004	-6.242	0.002	0.975
E_{LUMO} (eV)	-0.138	0.007	-3.089	0.003	0.989
Δ $E_{HOMO-LUMO}$ (eV)	-0.093	0.005	3.153	0.002	0.990
ΔE_{LET} (eV)	-0.084	0.005	2.384	0.002	0.987

3.2 Hammett-Potential Analysis of Referenced Work

Table S 5: Table of values used to perform the Hammett-Property analysis for platinum(II) dithiolate complexes of 4,4′-R-2,2′-bipyridine.[2] Energy values are in V or eV. [a] - σ_m is multiplied by two to provide a per-substituent analysis.

**Functional Group**	σ_m	$\sigma_m \times 2^a$	E_{red}	E_{ox}	**eHLG**	E_{em}	$E^{(*/-)}$	$E^{+/*}$
tert-butyl	-0.2	-0.4	-1.4	0.39	1.79	1.93	0.54	-1.55
CH3	-0.17	-0.34	-1.37	0.39	1.76	1.87	0.5	-1.48
H	0	0	-1.34	0.38	1.72	1.86	0.52	-1.49
Cl	0.23	0.46	-1.04	0.38	1.42	1.68	0.64	-1.30
CO2Et	0.45	0.9	-0.96	0.41	1.37	1.58	0.62	-1.17

Table S6: Table of values used to perform the Hammett-Property analysis for [(H_2pbbzim)Ru(4′-R′PhTerpy)]$(PF_6)_2$, where H_2pbbzim = 2,6-bis(benzimidazole-2-yl)pyridine and 4′-R′PhTerpy are 4′-tolyl-substituted terpyridine.[3] Energy values are in V or eV.

**Functional Group**	σ_p	λ_{em}	E_{em}	E_{ox}	E_{red}	**eHLG**	$E^{(+/*)}$	$E^{*/-}$
$PhCH_3$	-0.17	650	1.91	1.01	-1.48	2.49	-0.90	0.43
$PhCH_2Br$	0.14	661	1.88	1.07	-1.48	2.55	-0.81	0.4
$PhCH_2CN$	0.18	664	1.87	1.08	-1.48	2.56	-0.79	0.39
$PhCHBr_2$	0.32	671	1.85	1.12	-1.47	2.59	-0.73	0.38
PhCHO	0.42	675	1.84	1.15	-1.47	2.62	-0.69	0.37

Table S7: Table of values used to perform the Hammett-Property analysis for [Ir(ppy)$_2$(4,4′-R‴-bpy)](PF_6).[4] Energy values are in V or eV. [a] - σ_p is multiplied by two to provide a per-substituent analysis. [b] - Difference between DFT calculated HOMO and LUMO energies.

**Functional Group**	σ_p	$\sigma_p \times 2^a$	λ_{em}	E_{em}	E_{ox}	E_{red}	**eHLG**	$E^{(*/-)}$	$E^{+/*}$	$\Delta E_{HOMO-LUMO}$ [b]
NH_2	-0.66	-1.32	518	2.39	2.07	-0.65	2.72	1.74	-0.32	3.52
F	0.06	0.12	606	2.05	1.47	-0.76	2.23	1.29	-0.58	3.03
CN	0.66	1.32	651	1.90	1.10	-0.84	1.94	1.06	-0.8	2.52

Table S8: Table of values used to perform the Hammett-Property analysis for [Ir(ppy)$_2$(4,4′-R‴-bpy)](PF$_6$).[4] Energy values are in V or eV. ^{a} - σ_p is multiplied by two to provide a per-substituent analysis. ^{b} - Difference between DFT calculated HOMO and LUMO energies.

**Functional Group**	σ_p	$\sigma_p \times 2^a$	λ_{em}	E_{em}	E_{ox}	E_{red}	**eHLG**	$E^{(*/-)}$	$E^{+/*}$	$\Delta E_{HOMO-LUMO}$ ^{b}
NH_2	-0.66	-1.32	456	2.72	2	-0.94	2.94	1.78	-0.72	3.78
OMe	-0.27	-0.54	507	2.45	1.72	-1.06	2.78	1.39	-0.73	3.50
F	0.06	0.12	538	2.3	1.47	-1.13	2.6	1.17	-0.83	3.28
CN	0.66	1.32	598	2.07	1.08	-1.13	2.21	0.94	-0.99	2.77

Table S 9: Table of values used to perform the Hammett-Property analysis for [Ir(2,4-F-ppy)$_2$(RPhcarbene)].[5] Energy values are in V or eV. ^{a} - σ_m is multiplied by two to provide a per-substituent analysis. ^{b} - Difference between DFT calculated HOMO and LUMO energies.

**Functional Group**	σ_m	σ_m***2**^{a}	λ_{em}	E_{em}	E_{ox}	E_{red}	**eHLG**	$E^{(*/-)}$	$E^{+/*}$	$\Delta E_{HOMO-LUMO}$ ^{b}
H	0	0	507	2.45	0.56	-2.61	3.17	-0.16	-1.89	3.87
Me	-0.07	-0.14	515	2.41	0.51	-2.64	3.15	-0.23	-1.9	3.83
Cl	0.37	0.74	487	2.55	0.67	-2.55	3.22	0	-1.88	3.92
F	0.34	0.68	486	2.55	0.67	-2.57	3.24	-0.02	-1.88	3.91

4 NMR Spectra

[Page intentionally left black due to formatting requirements.]

4.1 Overlay of ^{1}H NMR Spectra

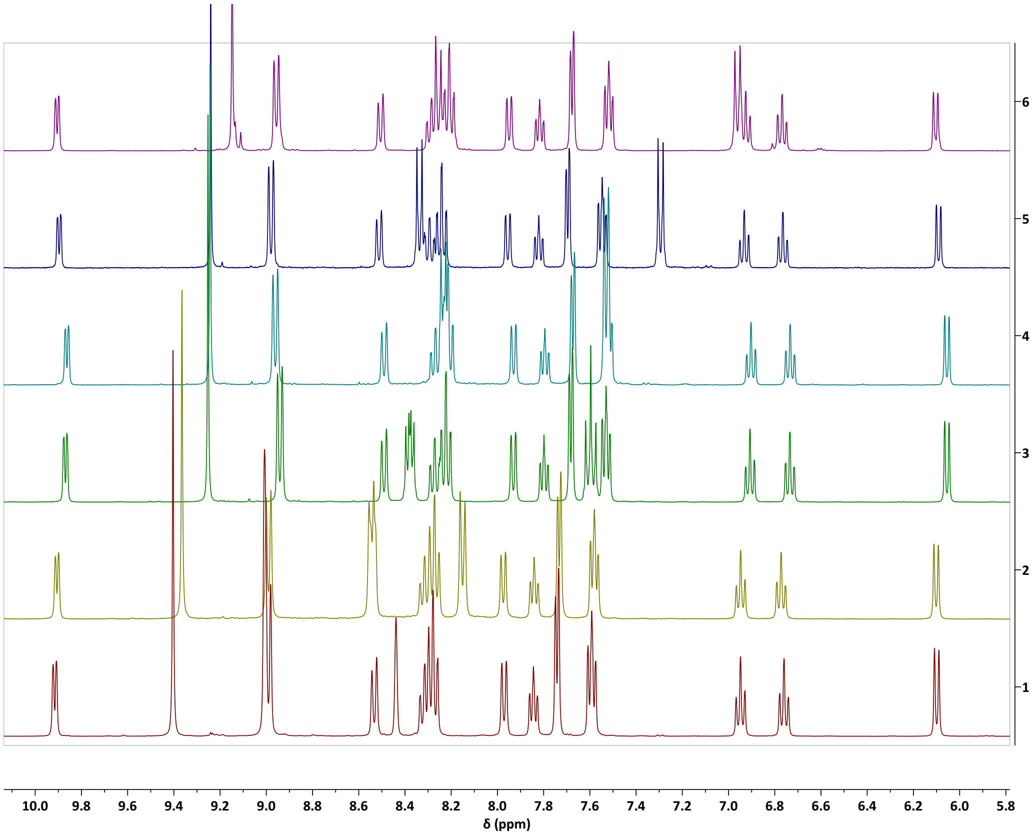

Figure S4: Stacked ^{1}H NMR spectra of [Ir(**RPhTerpy**)(ppy)Cl](PF$_6$). The spectrum labeled 1 is [Ir(**3,5-CF$_3$PhTerpy**)(ppy)Cl](PF$_6$), 2 = [Ir(**4-CF$_3$PhTerpy**)(ppy)Cl](PF$_6$), 3 = [Ir(**4-FPhTerpy**)(ppy)Cl](PF$_6$), 4 = [Ir(**4-MePhTerpy**)(ppy)Cl](PF$_6$), 5 = [Ir(**4-OMePhTerpy**)(ppy)Cl](PF$_6$), 6 = [Ir(**4-NMe$_2$PhTerpy**)(ppy)Cl](PF$_6$).

4.2 NMR Data for [Ir(3,5-CF_3PhTerpy)(ppy)Cl](PF_6)

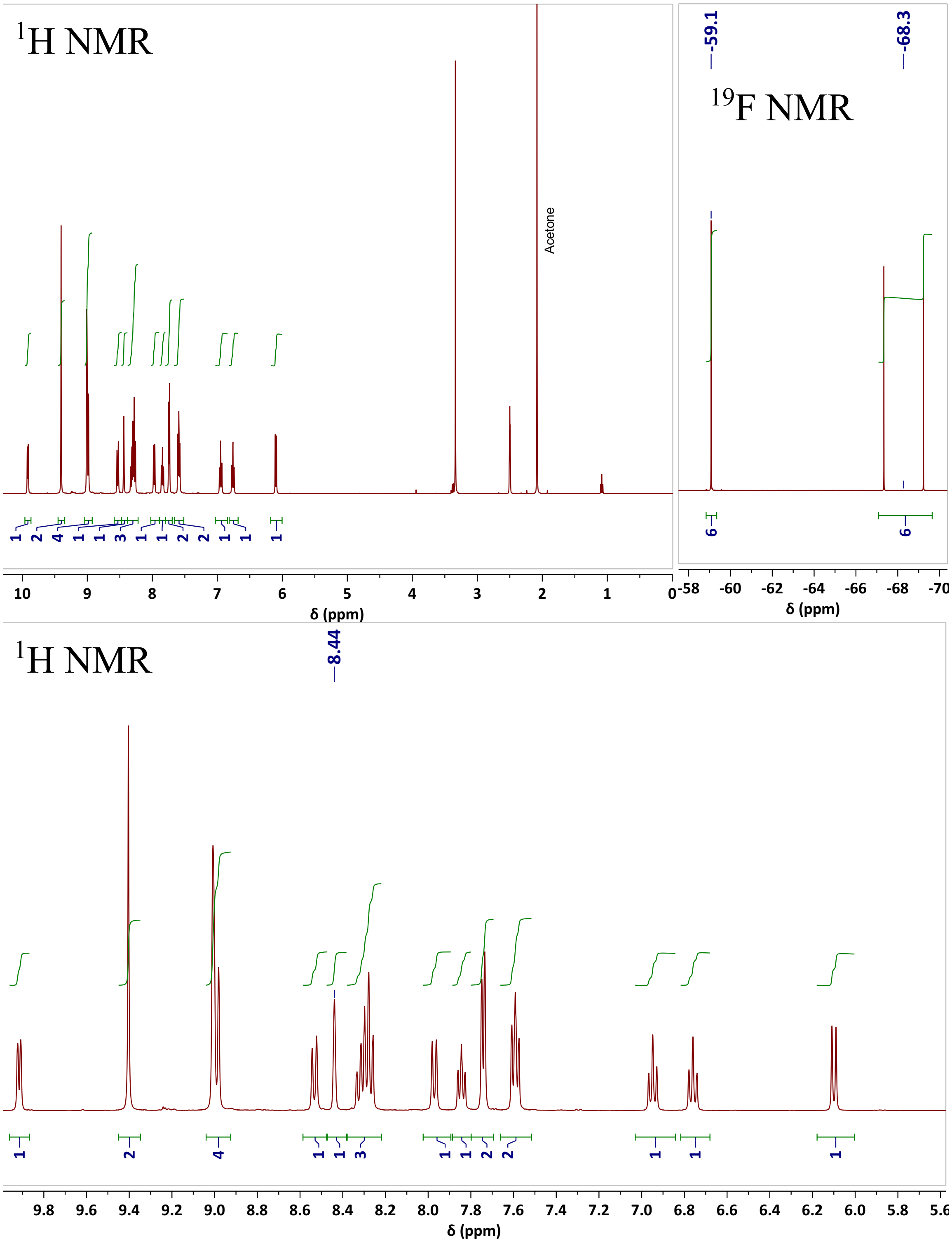

Figure S5: ^{1}H NMR Spectrum (top left) and expanded ^{1}H NMR Spectrum (bottom) and ^{19}F NMR spectrum (top right) of 3,5-CF_3PhTerpy collected in DMSO-d_6.

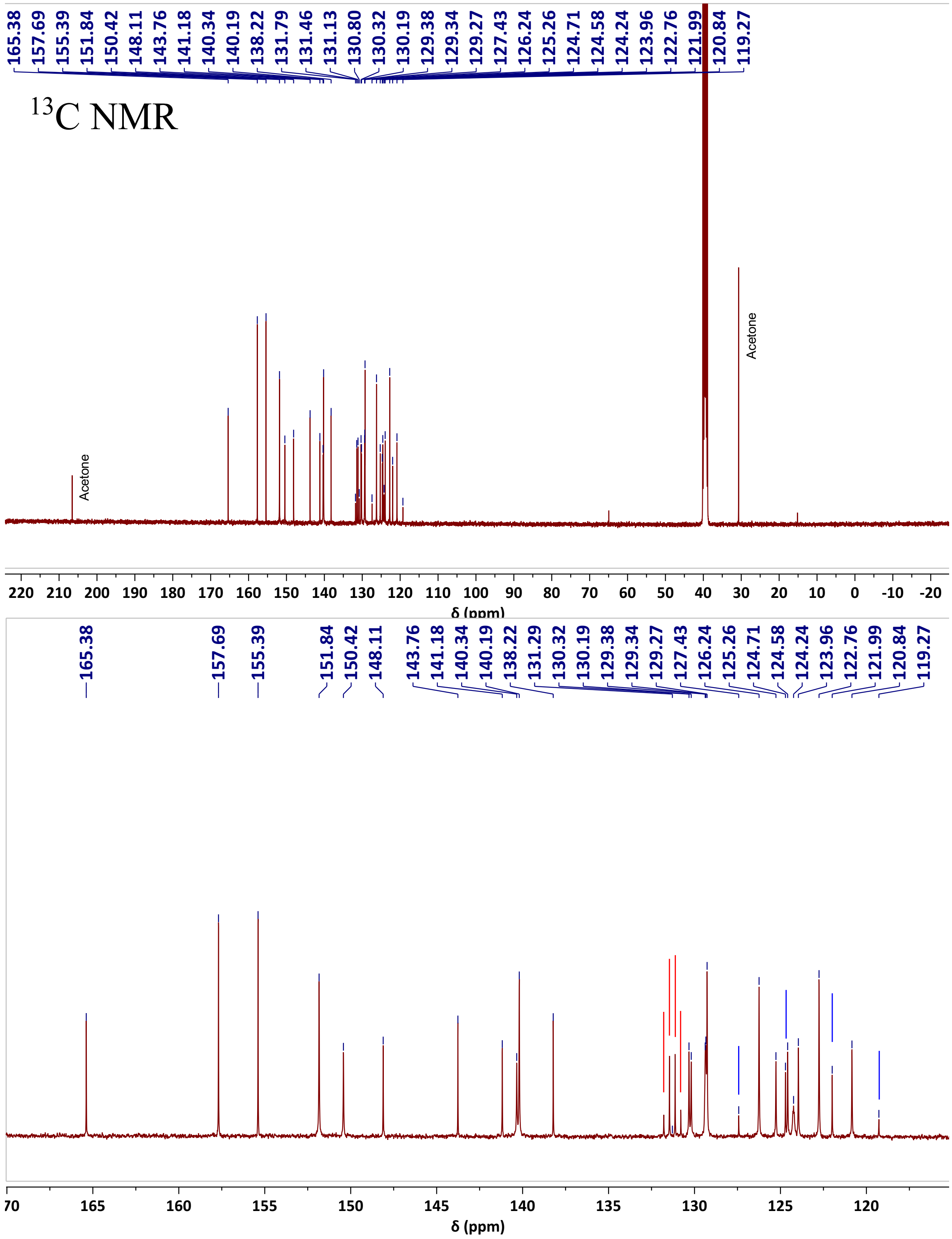

Figure S6: ^{13}C NMR Spectrum (top) and expanded ^{13}C NMR Spectrum (bottom) of [Ir(**3,5-CF$_3$PhTerpy**)(ppy)Cl](PF$_6$) collected in DMSO-d_6. Note: the red lines indicate the quartet of the trifluoromethyl carbon.

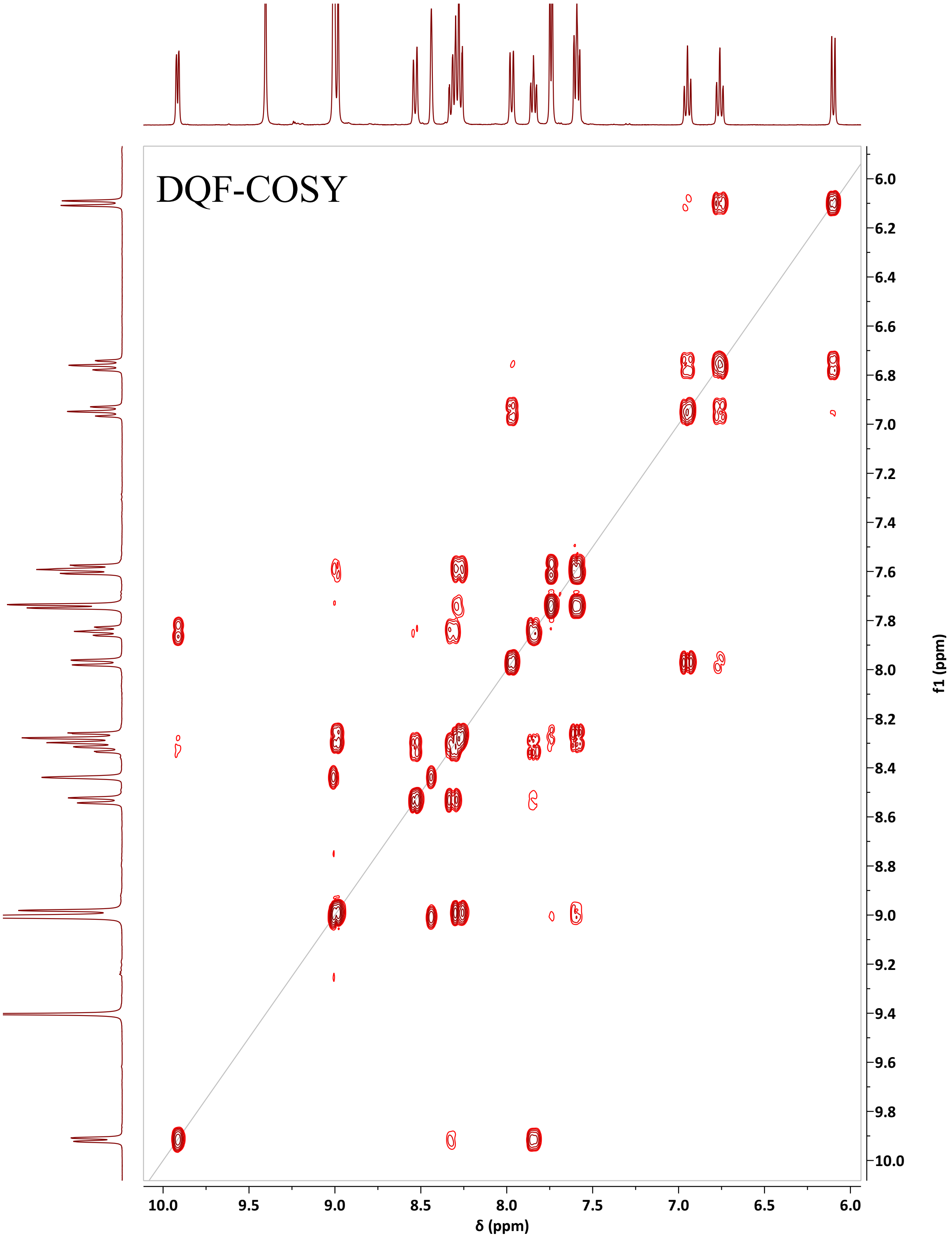

Figure S7: ^{1}H-^{1}H DQF-COSY spectrum of [Ir(**3,5-CF$_3$PhTerpy**)(ppy)Cl](PF$_6$) collected in DMSO-d_6.

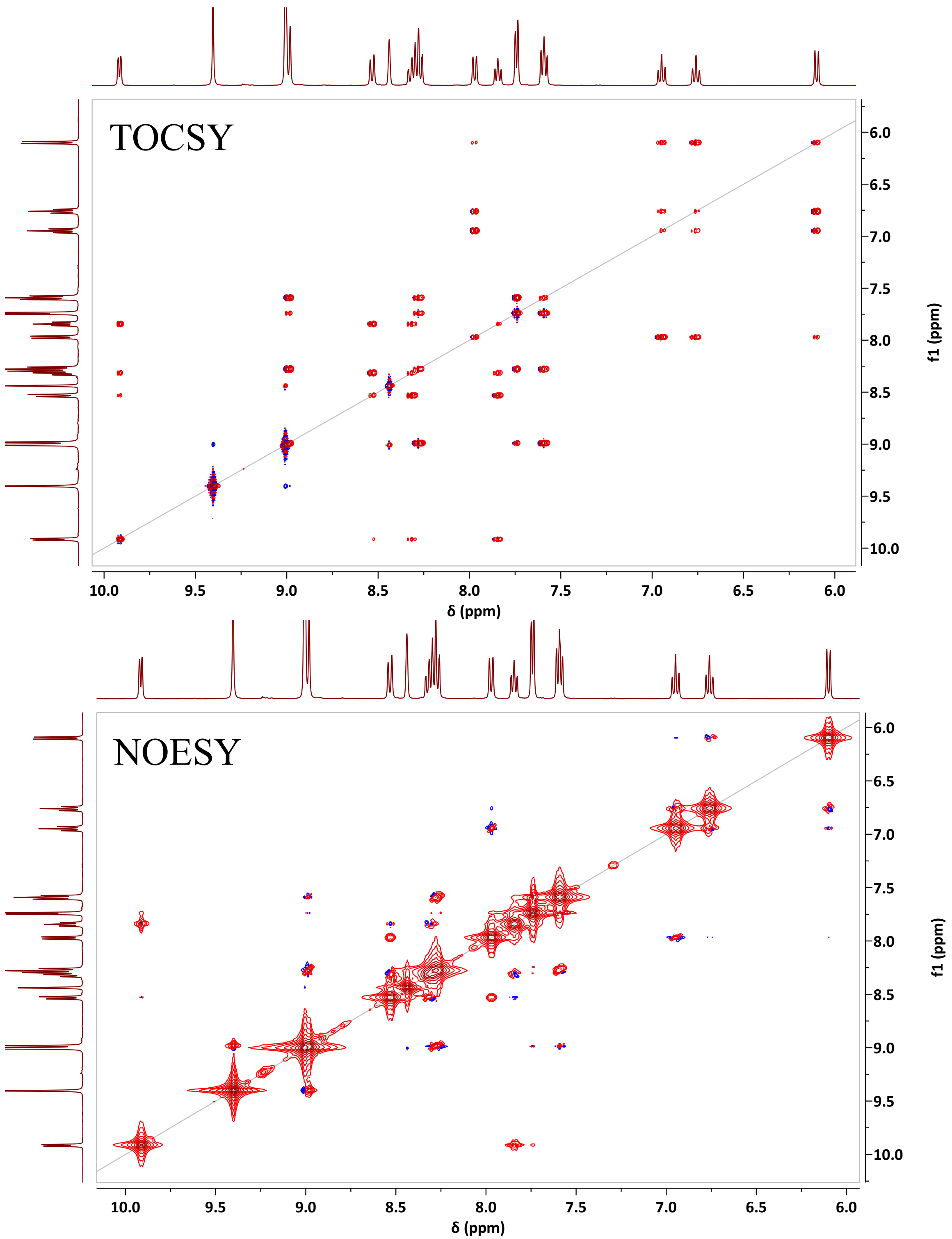

Figure S8: ^{1}H-^{1}H TOCSY spectrum (top) of [Ir(**3,5-CF$_3$PhTerpy**)(ppy)Cl](PF$_6$) collected in DMSO-d_6 and the ^{1}H-^{1}H NOESY spectrum (bottom).

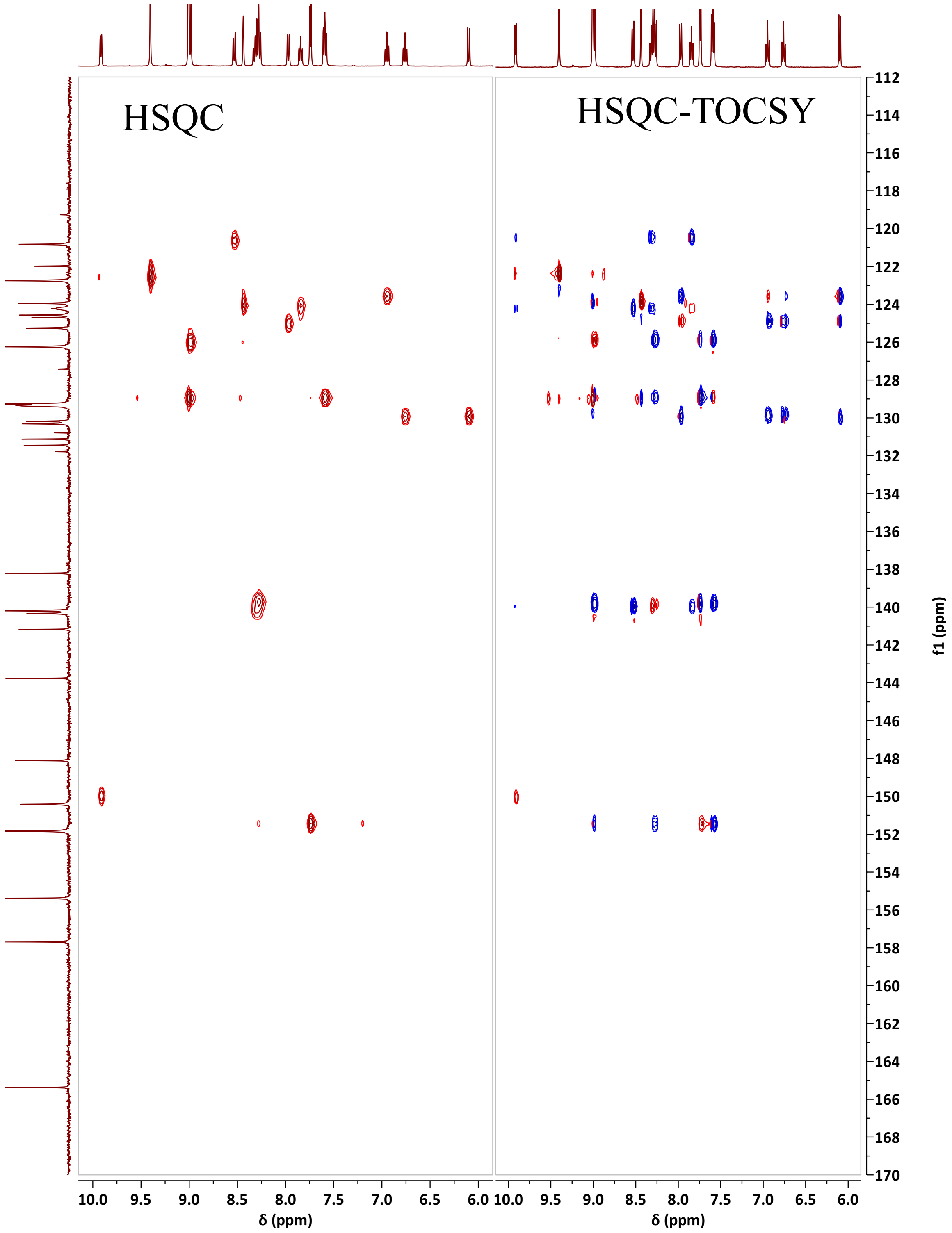

Figure S9: ^{1}H-^{13}C Edited-HSQC spectrum (left) and ^{1}H-^{13}C HSQC-TOCSY spectrum (right) of [Ir(**3,5-CF$_3$PhTerpy**)(ppy)Cl](PF_6) collected in DMSO-d_6.

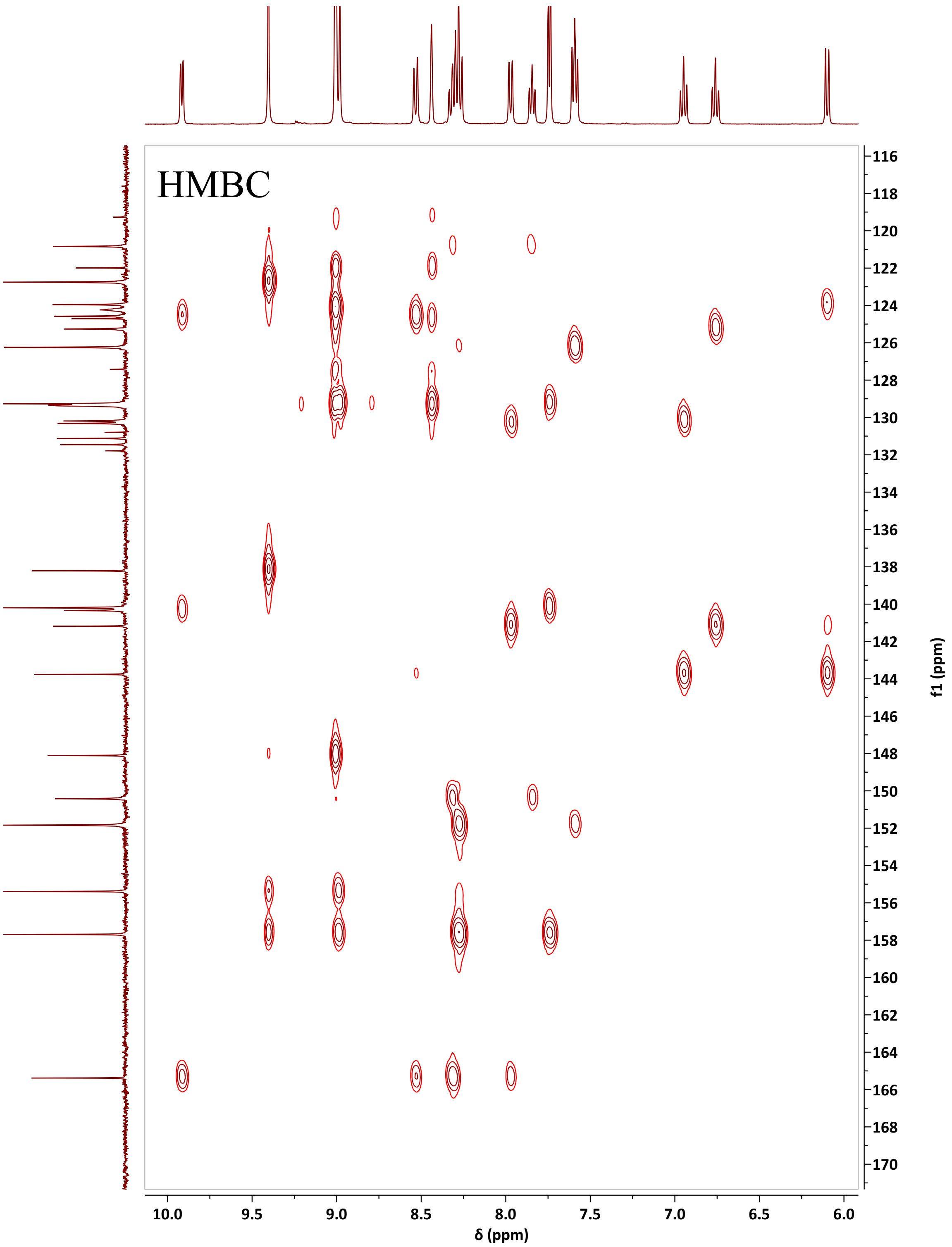

Figure S10: ^{1}H-^{13}C HMBC spectrum of [Ir(**3,5-CF$_3$PhTerpy**)(ppy)Cl](PF$_6$) collected in DMSO-d_6.

4.3 NMR Data for [Ir(4-CF$_3$PhTerpy)(ppy)Cl](PF$_6$)

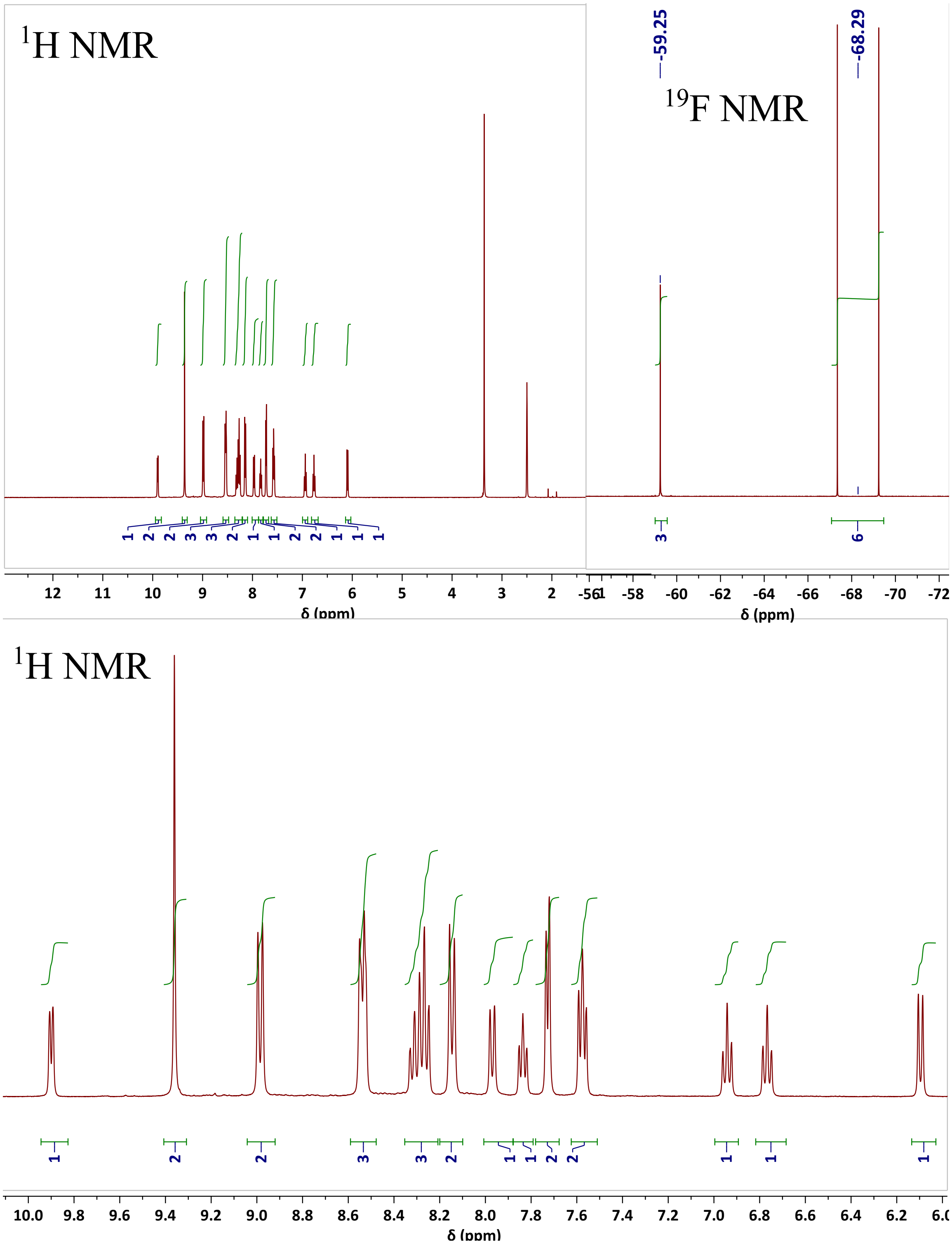

Figure S11: ^{1}H NMR Spectrum (top left) and expanded ^{1}H NMR Spectrum (bottom) and ^{19}F NMR spectrum (top right) of 4-CF$_3$PhTerpy collected in DMSO-d_6.

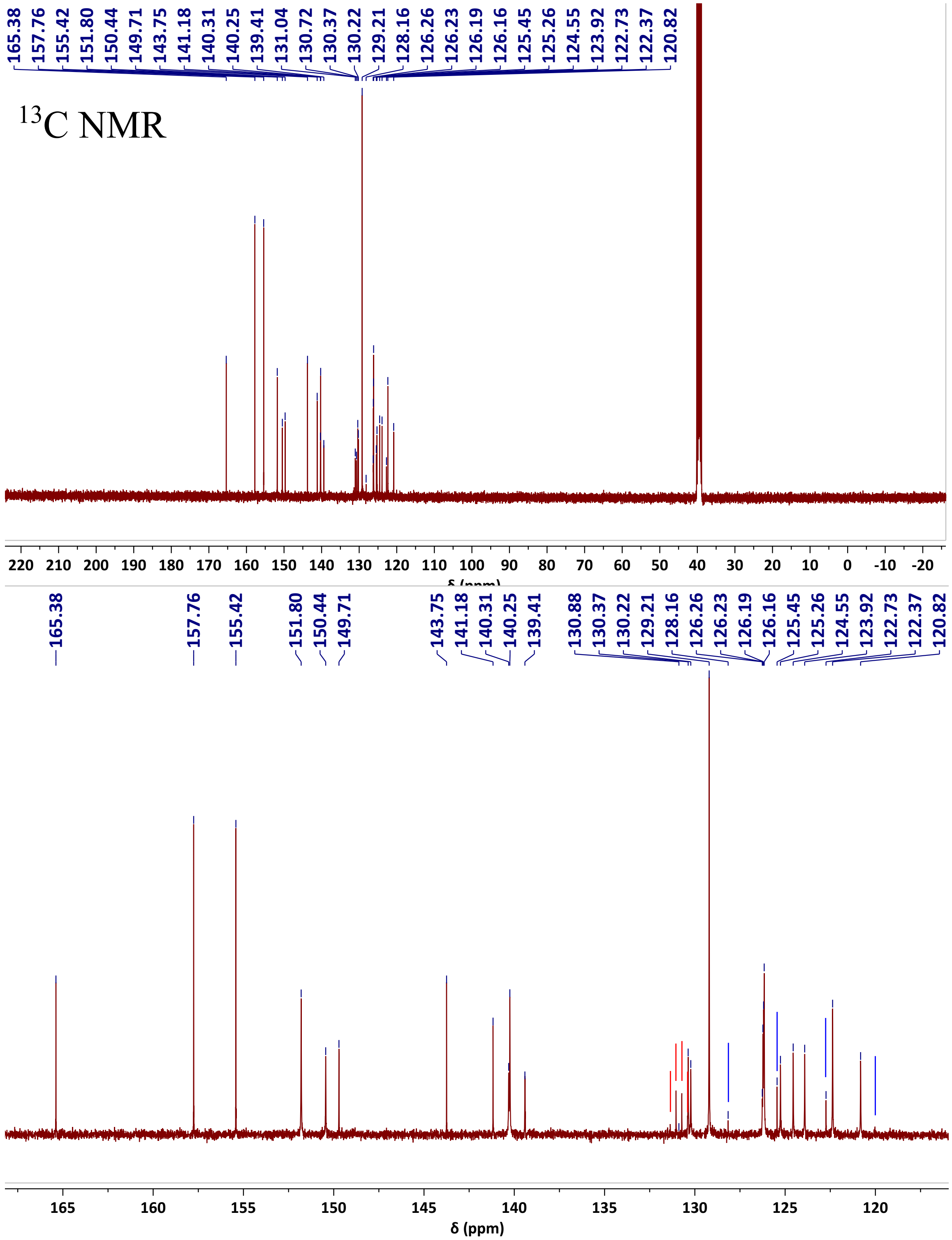

Figure S12: ^{13}C NMR Spectrum (top) and expanded ^{13}C NMR Spectrum (bottom) of [Ir(**4-CF$_3$PhTerpy**)(ppy)Cl](PF$_6$) collected in DMSO-d_6. Note: the red lines indicate the quartet of the trifluoromethyl carbon.

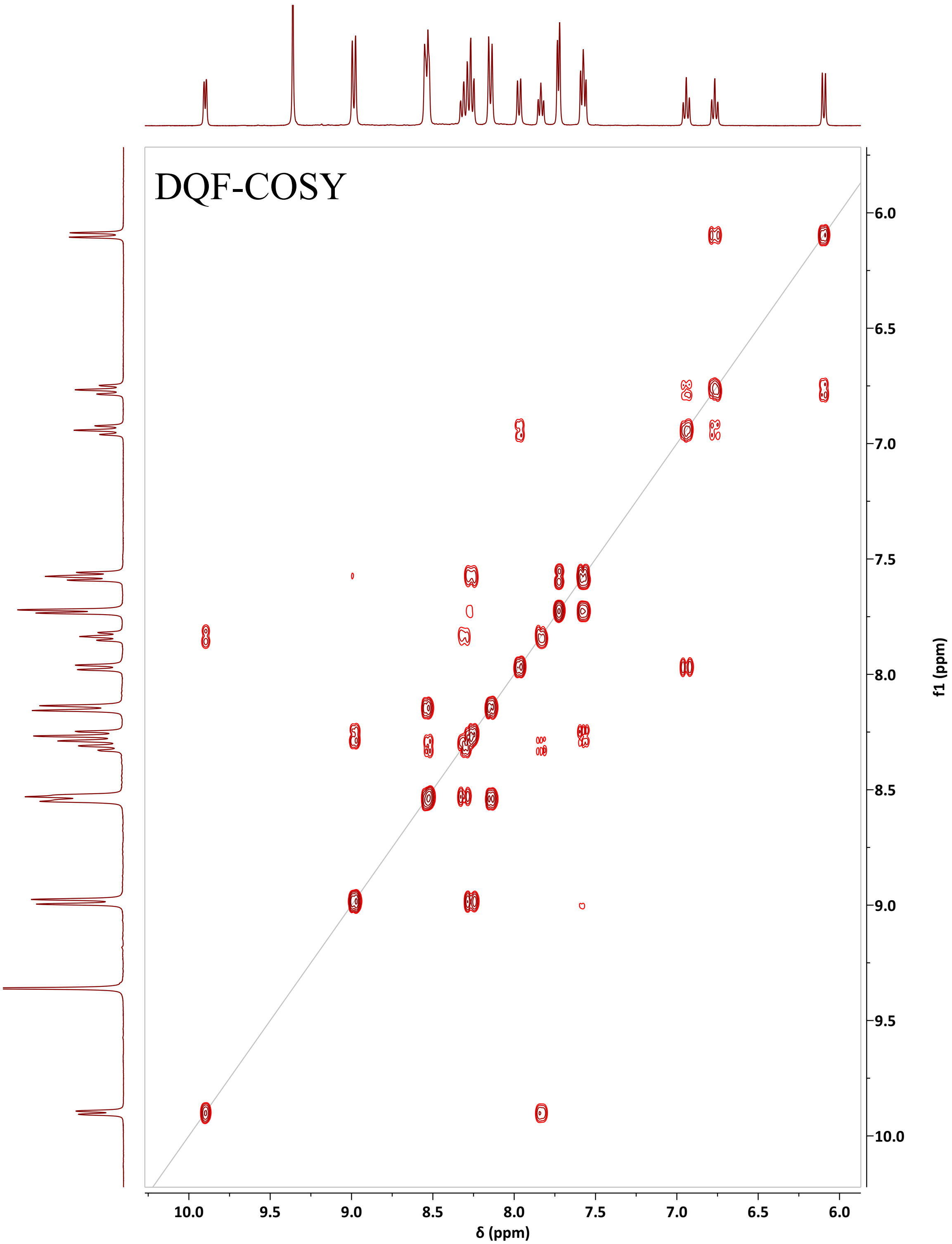

Figure S13: ^{1}H-^{1}H DQF-COSY spectrum of [Ir(**4-CF$_3$PhTerpy**)(ppy)Cl](PF$_6$) collected in DMSO-d_6.

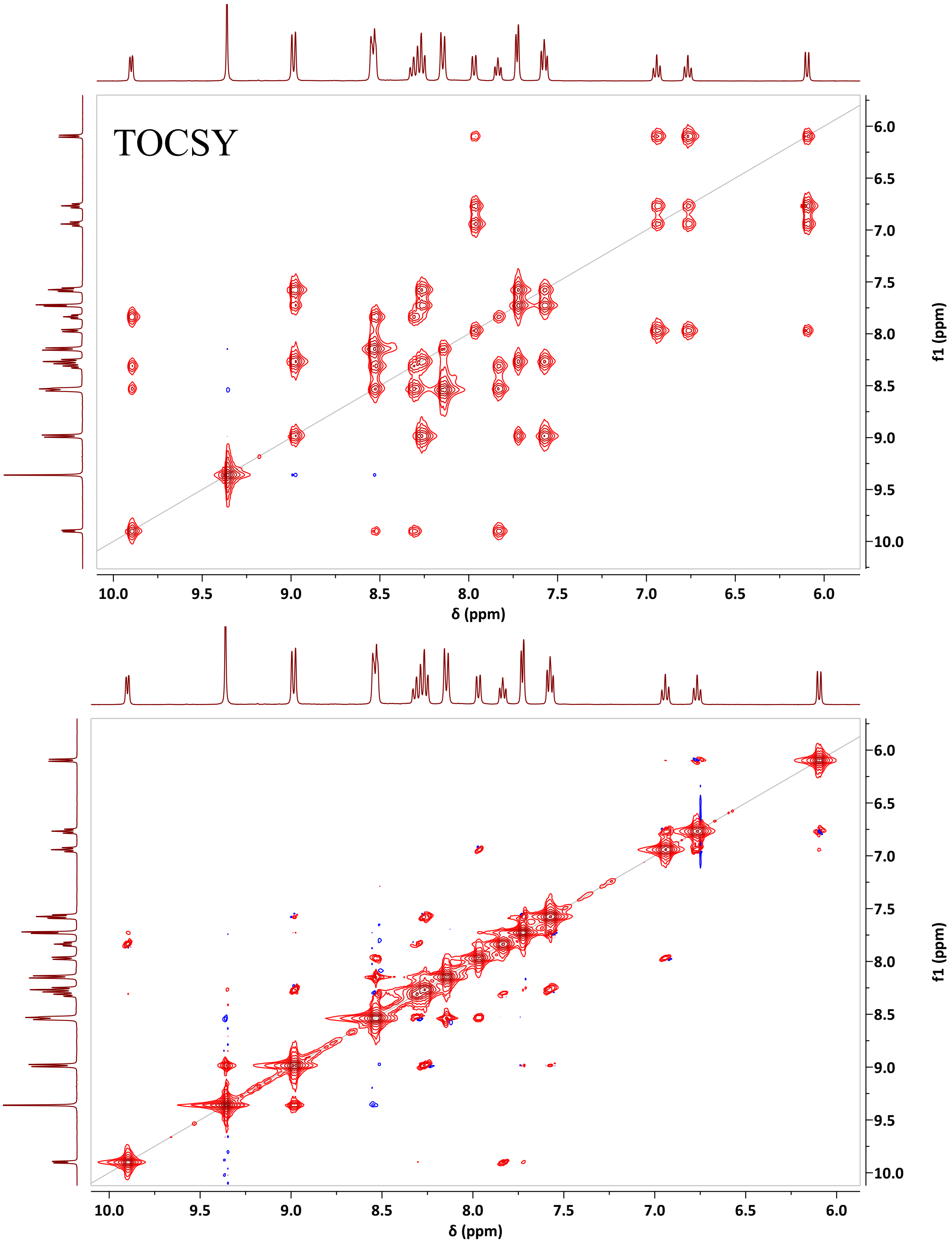

Figure S14: ^{1}H-^{1}H TOCSY spectrum (top) of [Ir(**4-CF$_3$PhTerpy**)(ppy)Cl](PF_6) collected in DMSO-d_6 and the ^{1}H-^{1}H NOESY spectrum (bottom).

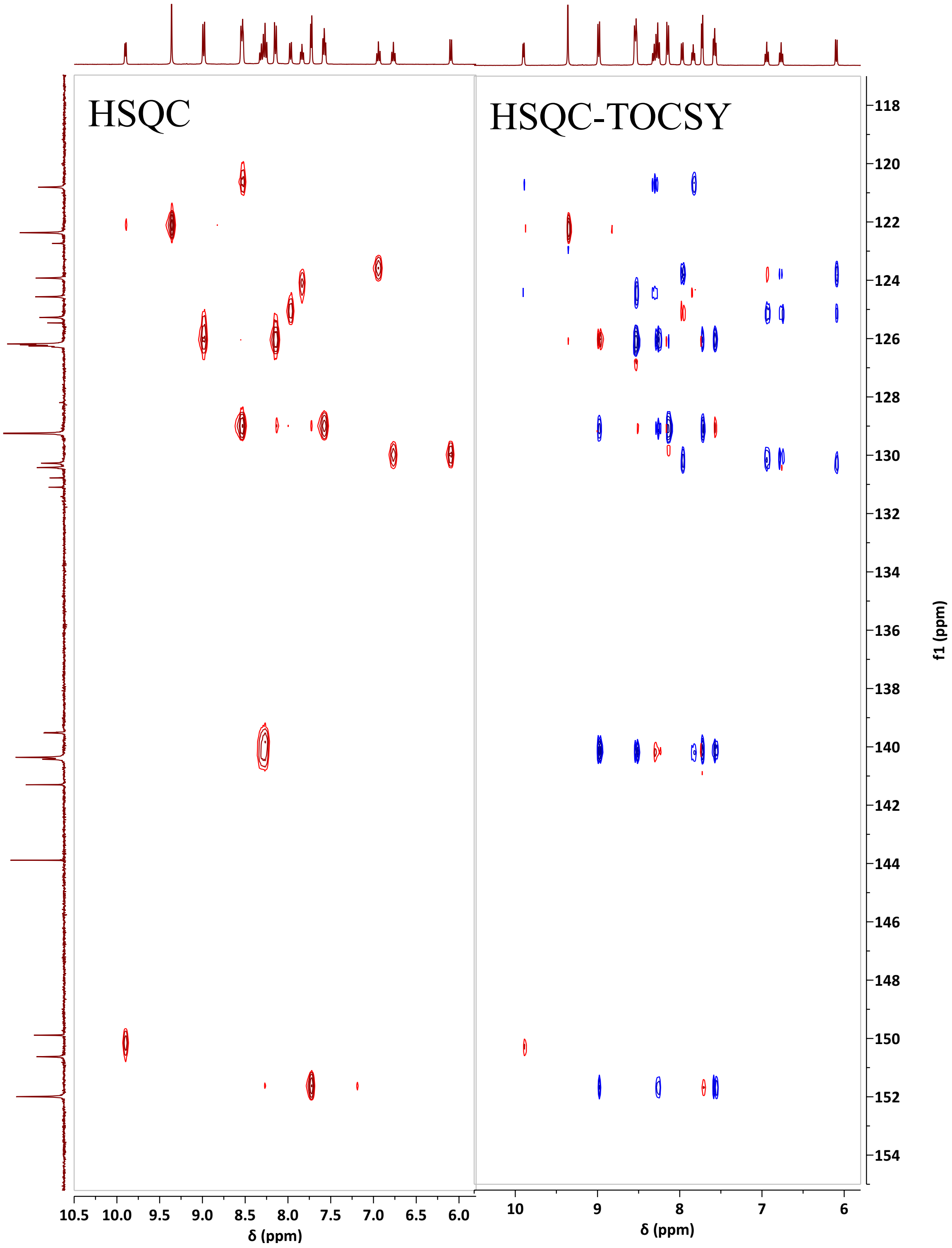

Figure S15: ^{1}H-^{13}C Edited-HSQC spectrum (left) and ^{1}H-^{13}C HSQC-TOCSY spectrum (right) of [Ir(**4-CF$_3$PhTerpy**)(ppy)Cl](PF_6) collected in DMSO-d_6.

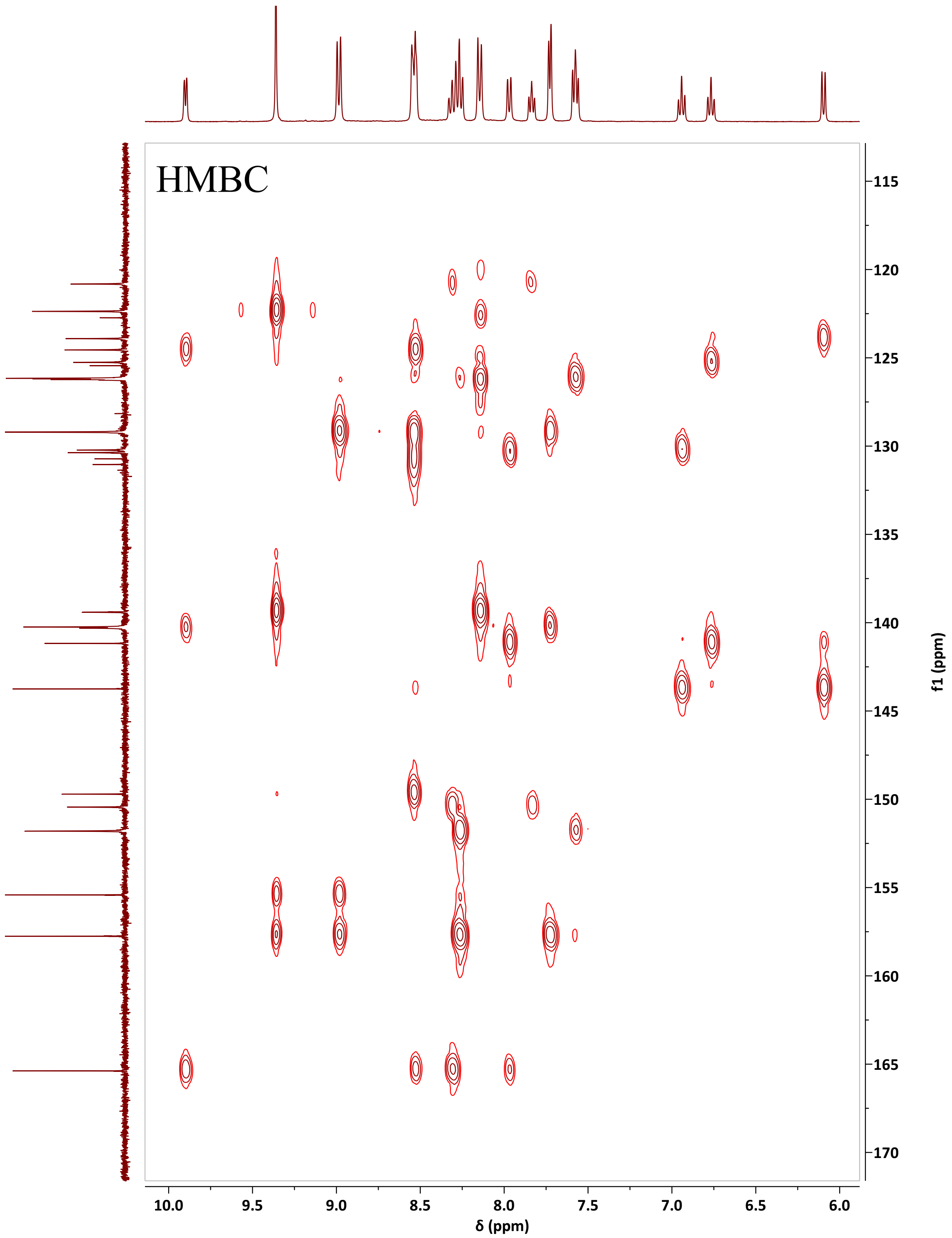

Figure S16: ^{1}H-^{13}C HMBC spectrum of [Ir(**4-CF$_3$PhTerpy**)(ppy)Cl](PF$_6$) collected in DMSO-d_6.

4.4 NMR Data for [Ir(4-FPhTerpy)(ppy)Cl](PF_6)

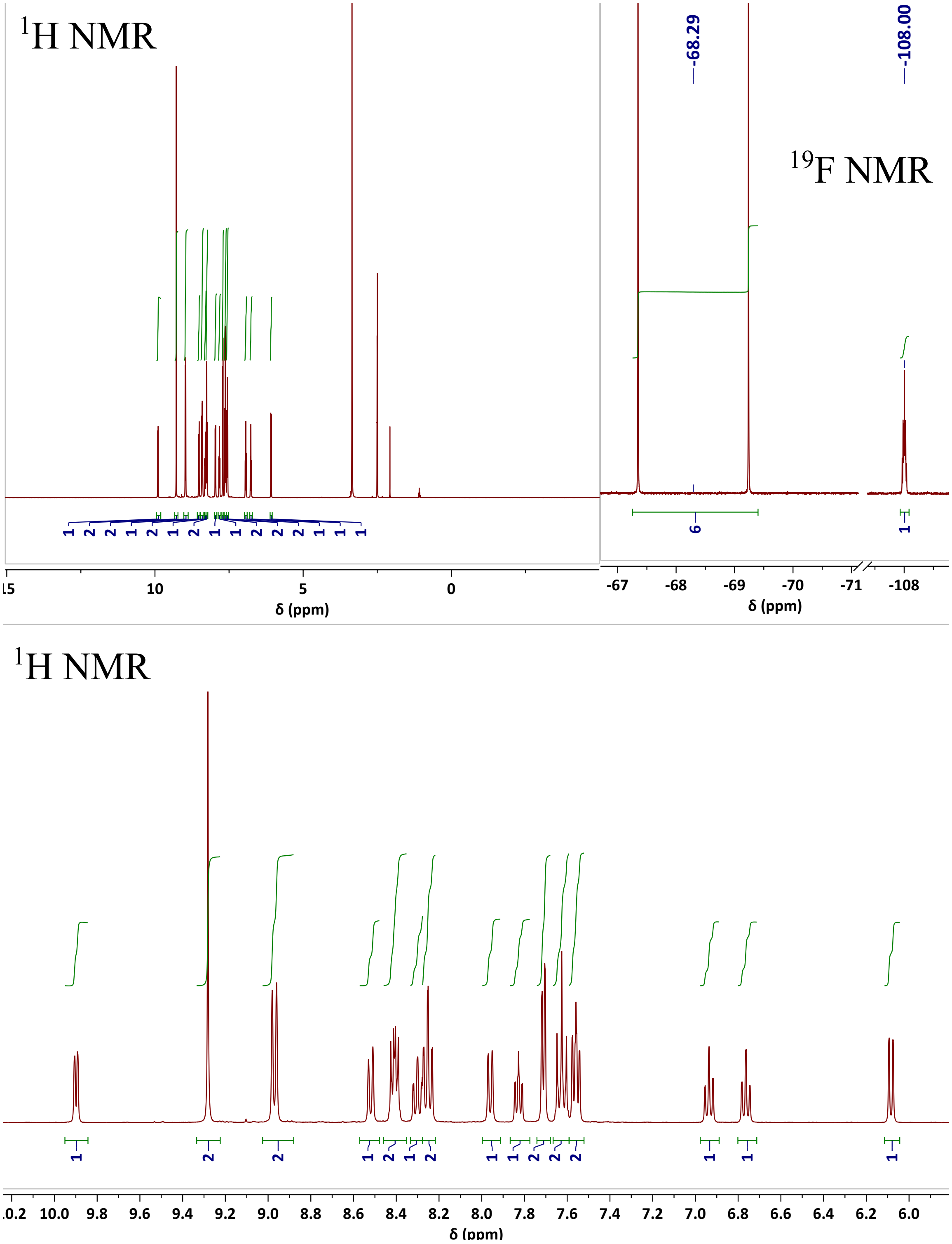

Figure S17: ^{1}H NMR Spectrum (top left) and expanded ^{1}H NMR Spectrum (bottom) and ^{19}F NMR spectrum (top right) of 4-FPhTerpy collected in DMSO-d_6.

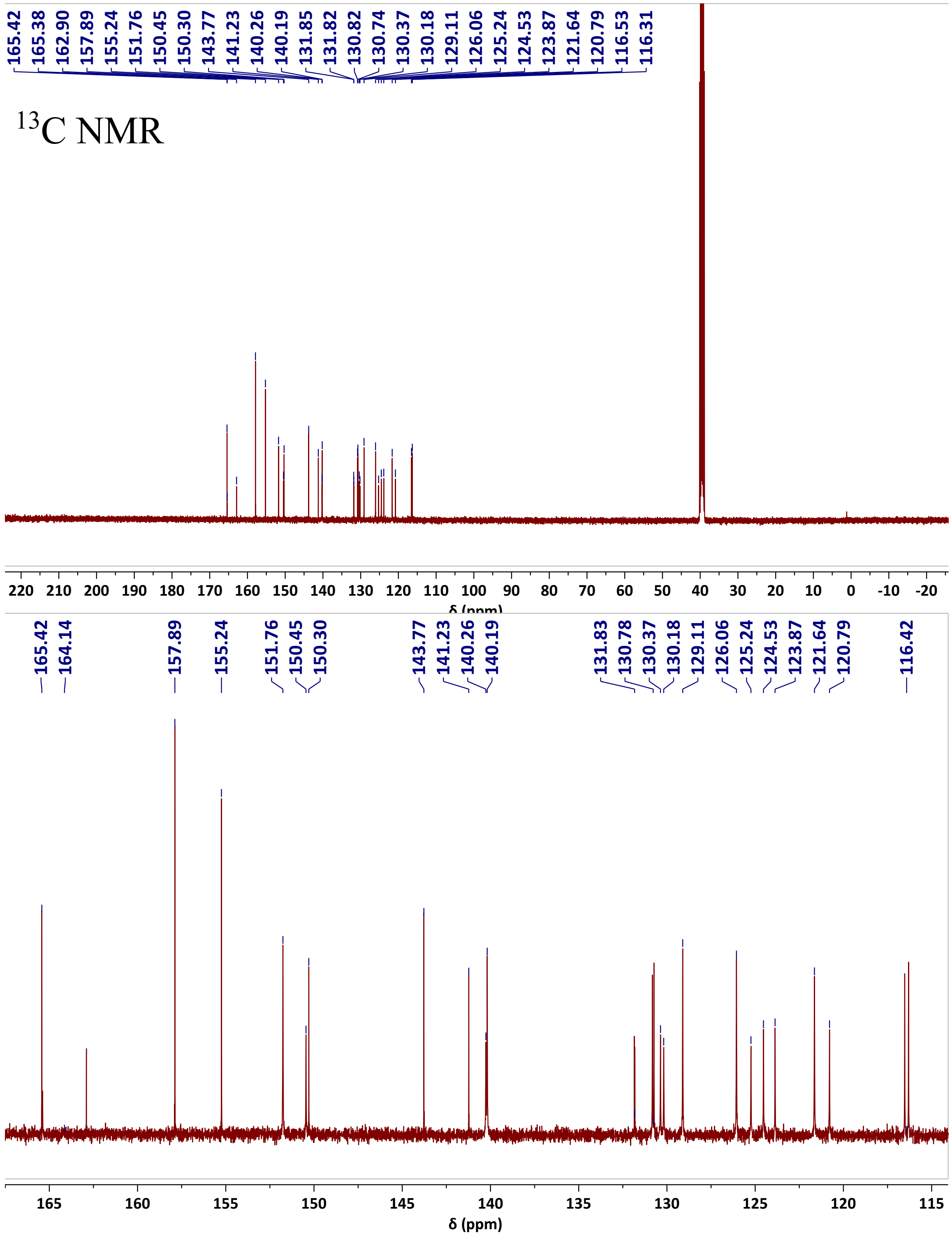

Figure S18: ^{13}C NMR Spectrum (top) and expanded ^{13}C NMR Spectrum (bottom) of [Ir(**4-FPhTerpy**)(ppy)Cl](PF$_6$) collected in DMSO-d_6.

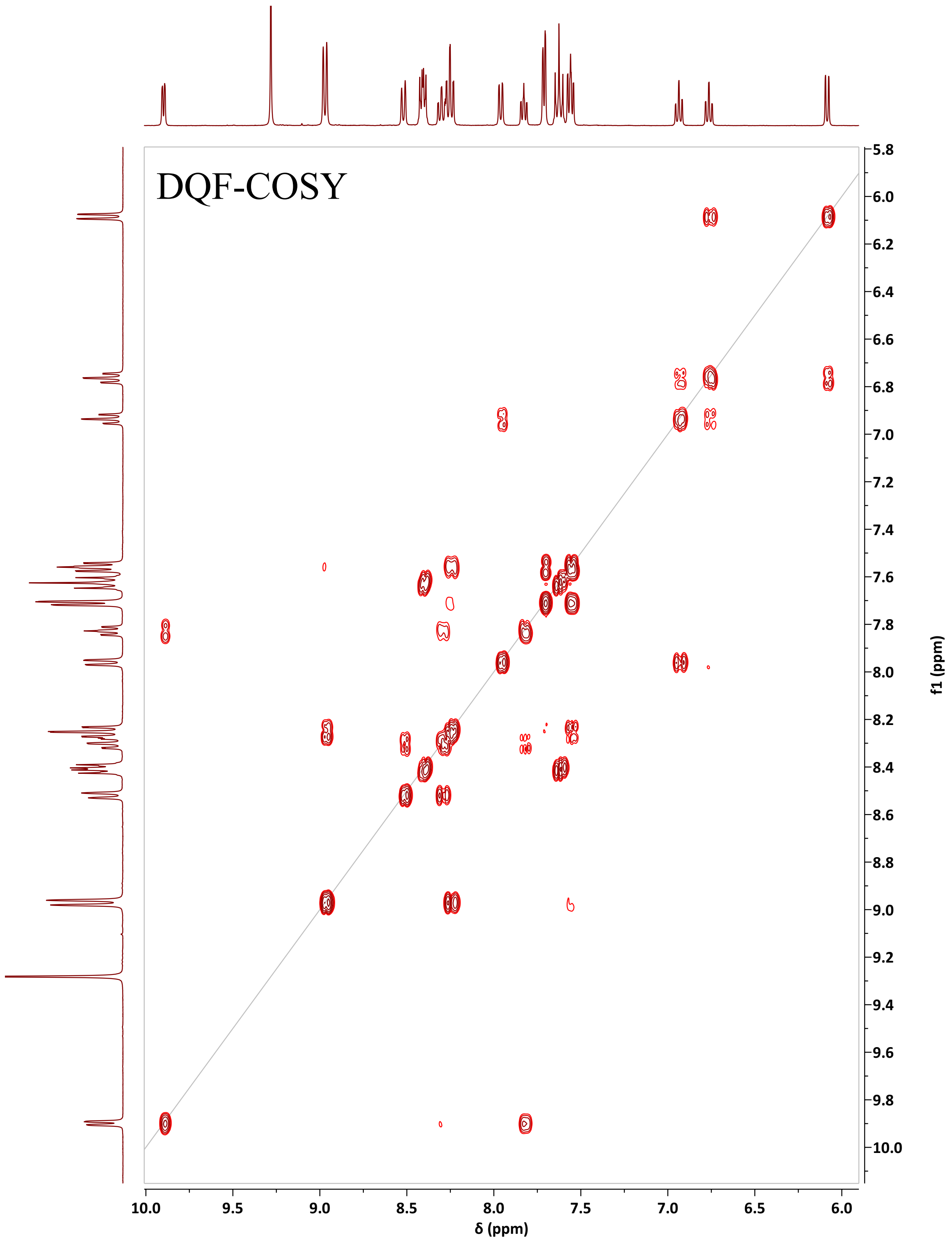

Figure S19: ^{1}H-^{1}H DQF-COSY spectrum of [Ir(**4-FPhTerpy**)(ppy)Cl](PF_6) collected in DMSO-d_6.

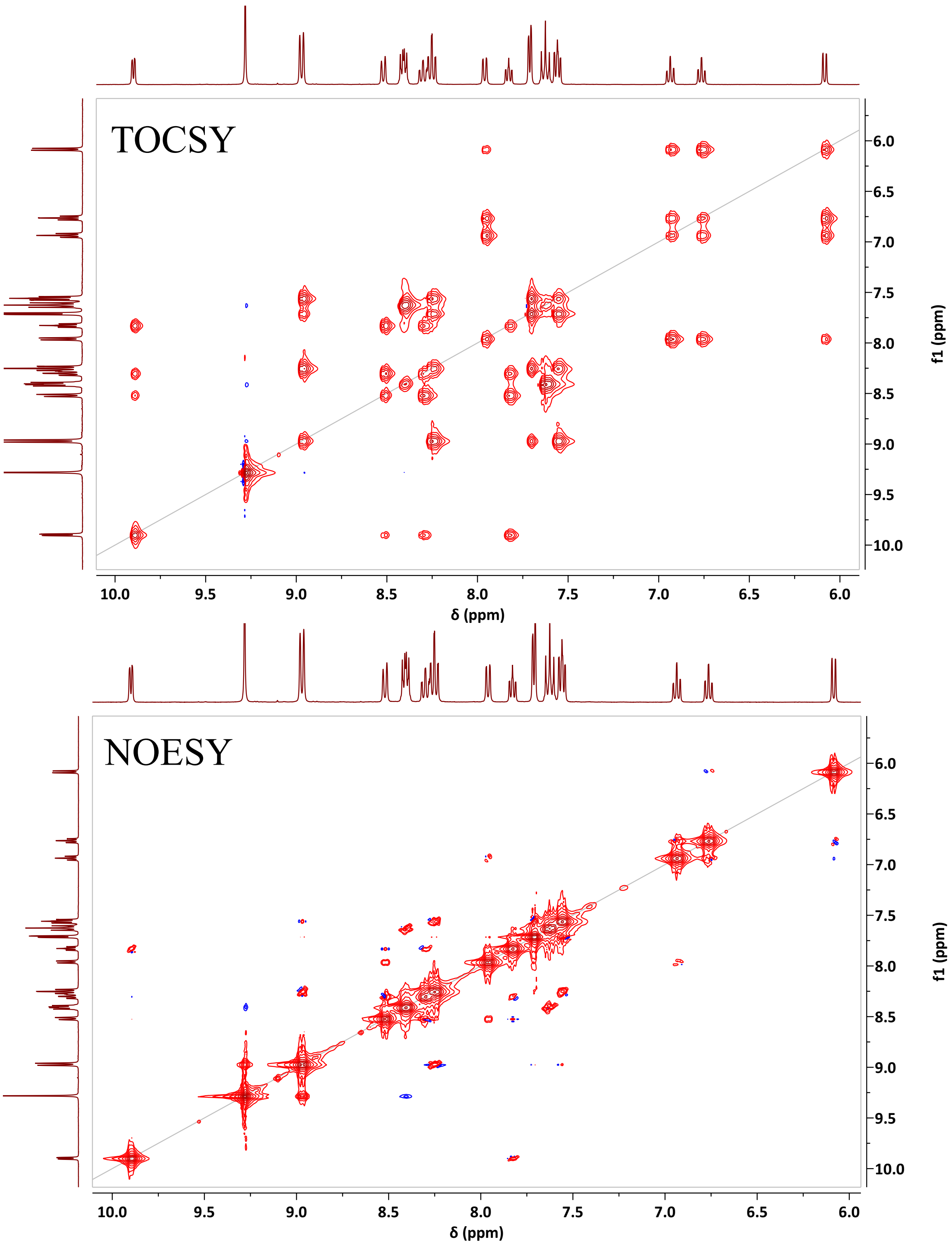

Figure S20: ^{1}H-^{1}H TOCSY spectrum (top) of [Ir(**4-FPhTerpy**)(ppy)Cl](PF_6) collected in DMSO-d_6 and the ^{1}H-^{1}H NOESY spectrum (bottom).

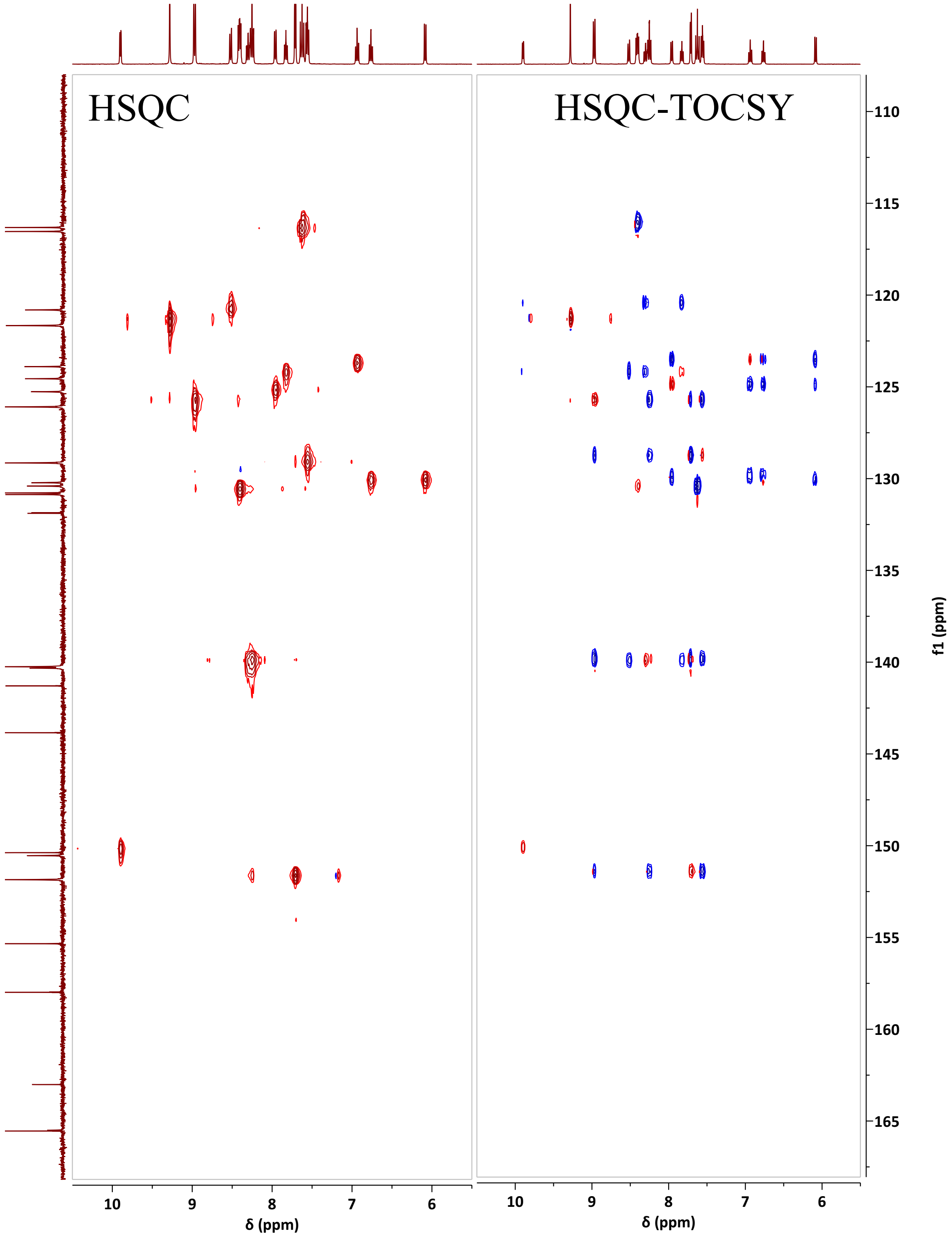

Figure S21: ^{1}H-^{13}C Edited-HSQC spectrum (left) and ^{1}H-^{13}C HSQC-TOCSY spectrum (right) of [Ir(**4-FPhTerpy**)(ppy)Cl](PF_6) collected in DMSO-d_6.

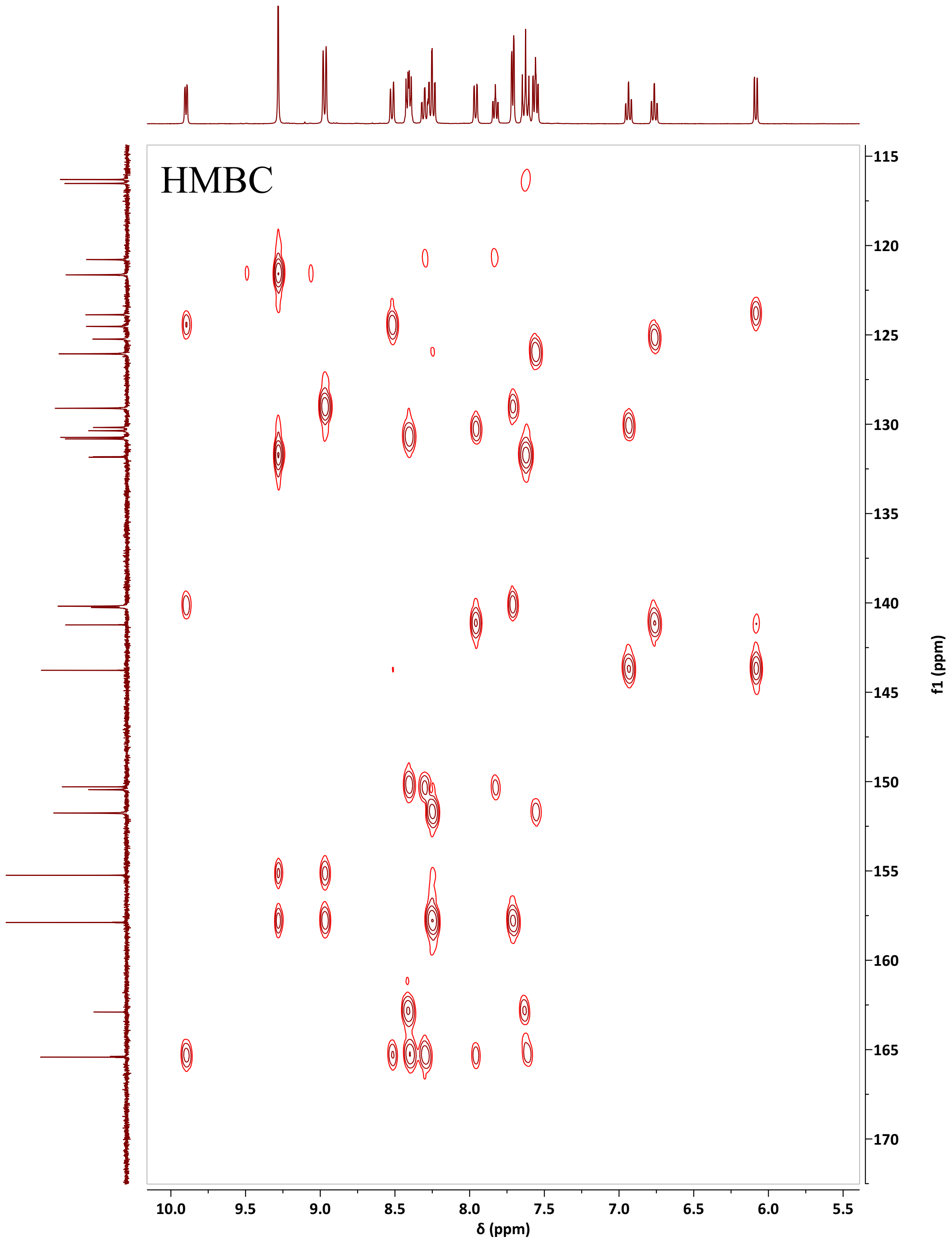

Figure S22: ^{1}H-^{13}C HMBC spectrum of [Ir(**4-FPhTerpy**)(ppy)Cl](PF_6) collected in DMSO-d_6.

4.5 NMR Data for [Ir(4-MePhTerpy)(ppy)Cl](PF_6)

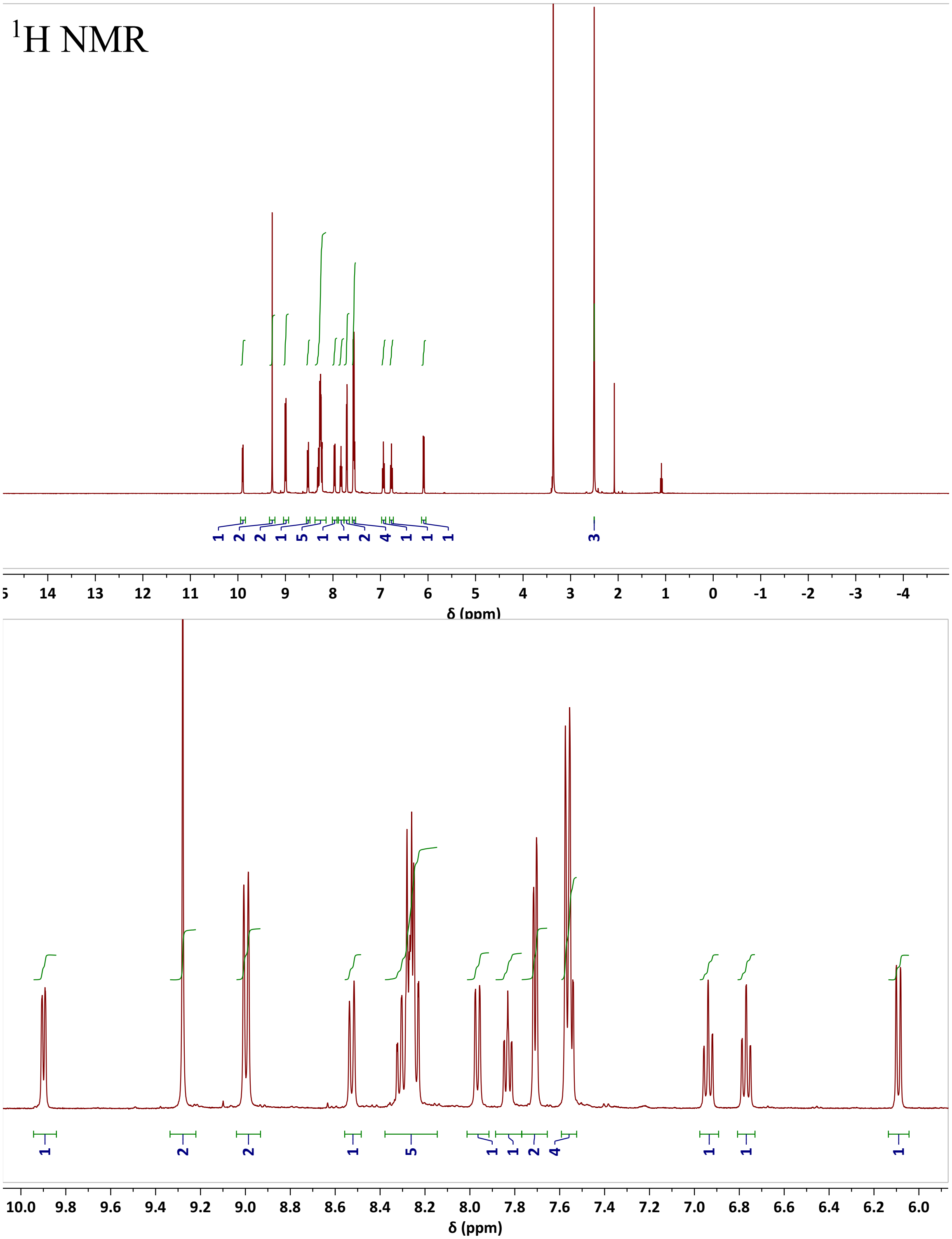

Figure S23: ^{1}H NMR Spectrum (top left) and expanded ^{1}H NMR Spectrum (bottom) and ^{19}F NMR spectrum (top right) of 4-MePhTerpy collected in DMSO-d_6.

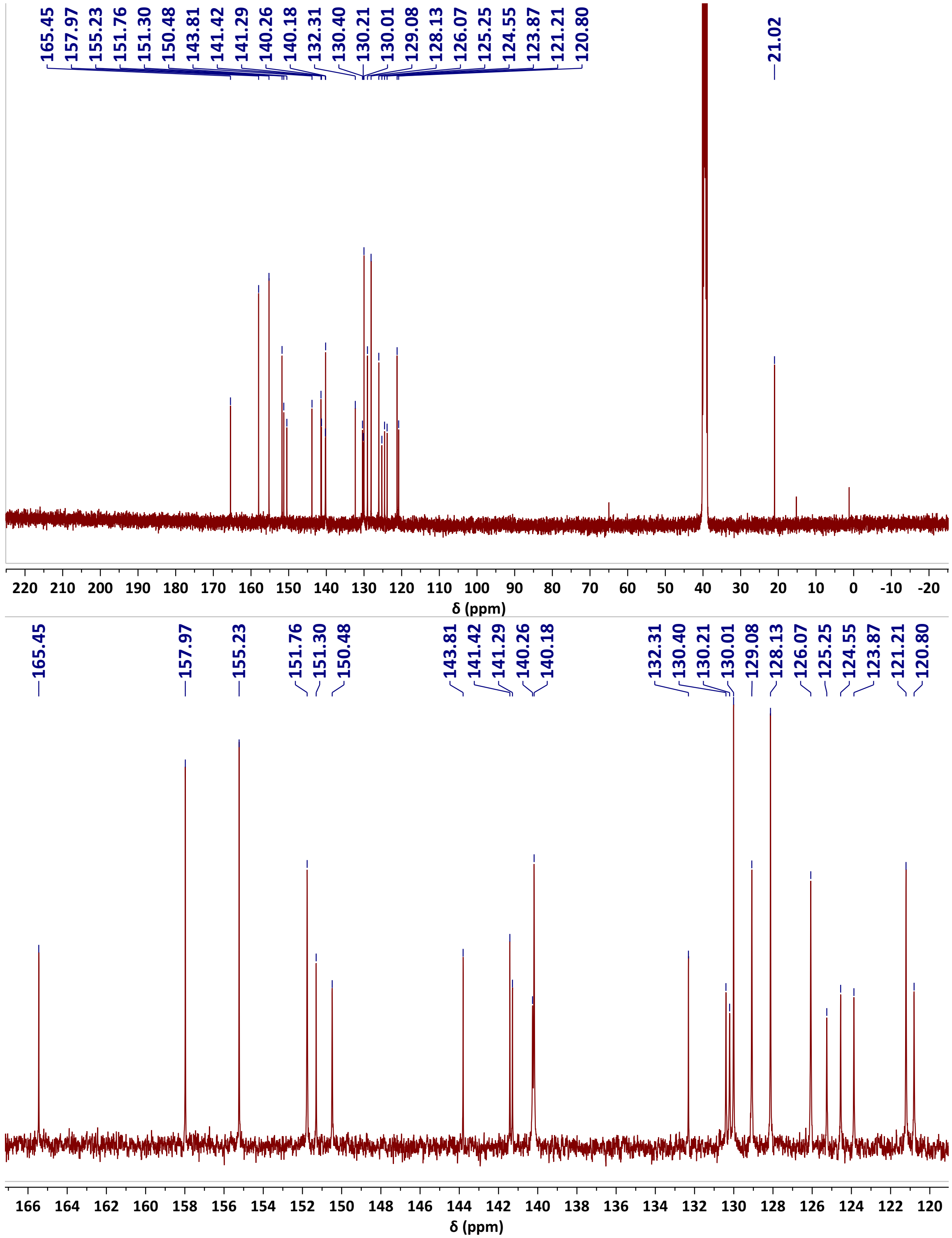

Figure S24: ^{13}C NMR Spectrum (top) and expanded ^{13}C NMR Spectrum (bottom) of [Ir(**4-MePhTerpy**)(ppy)Cl](PF_6) collected in DMSO-d_6.

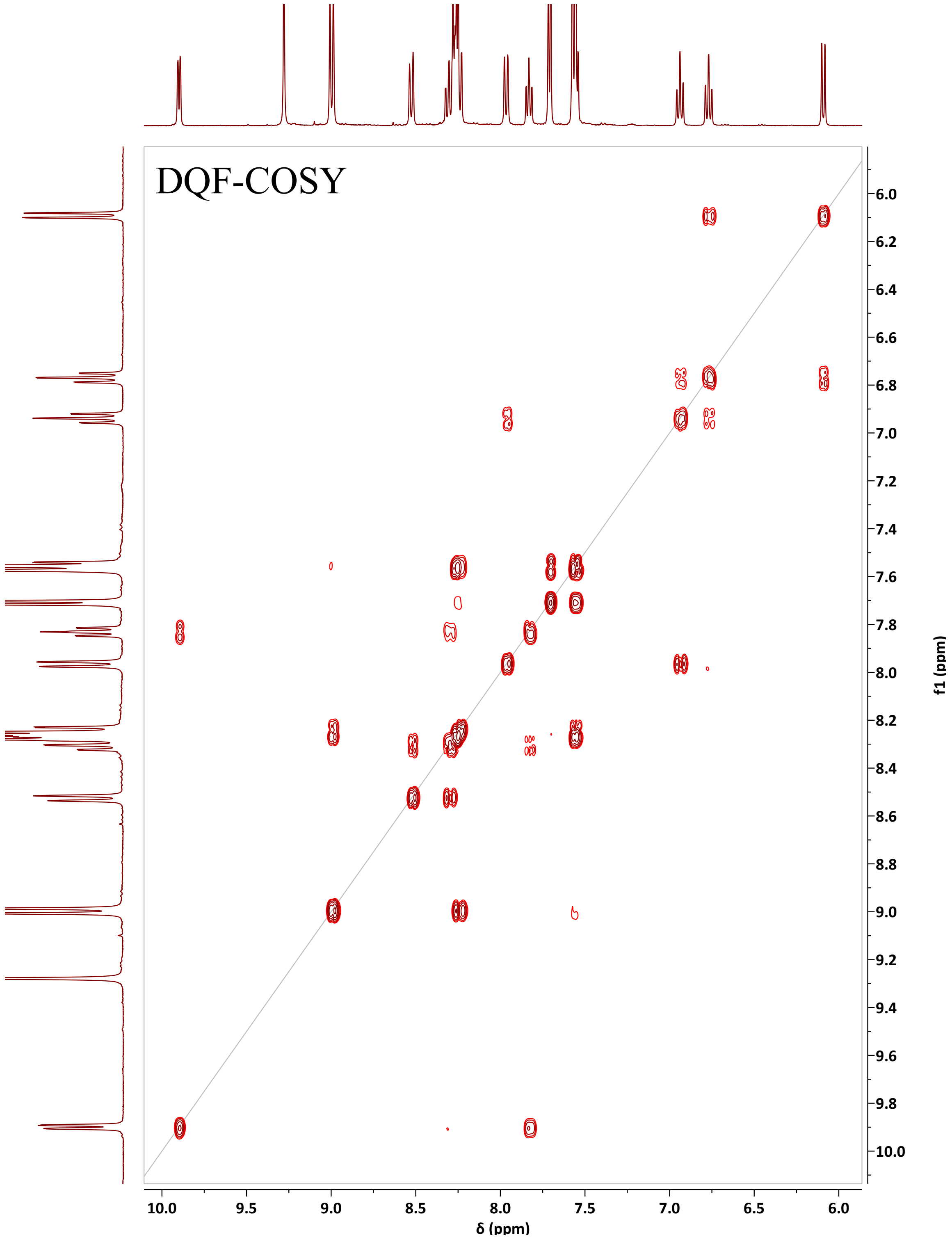

Figure S25: ^{1}H-^{1}H DQF-COSY spectrum of [Ir(**4-MePhTerpy**)(ppy)Cl](PF_6) collected in DMSO-d_6.

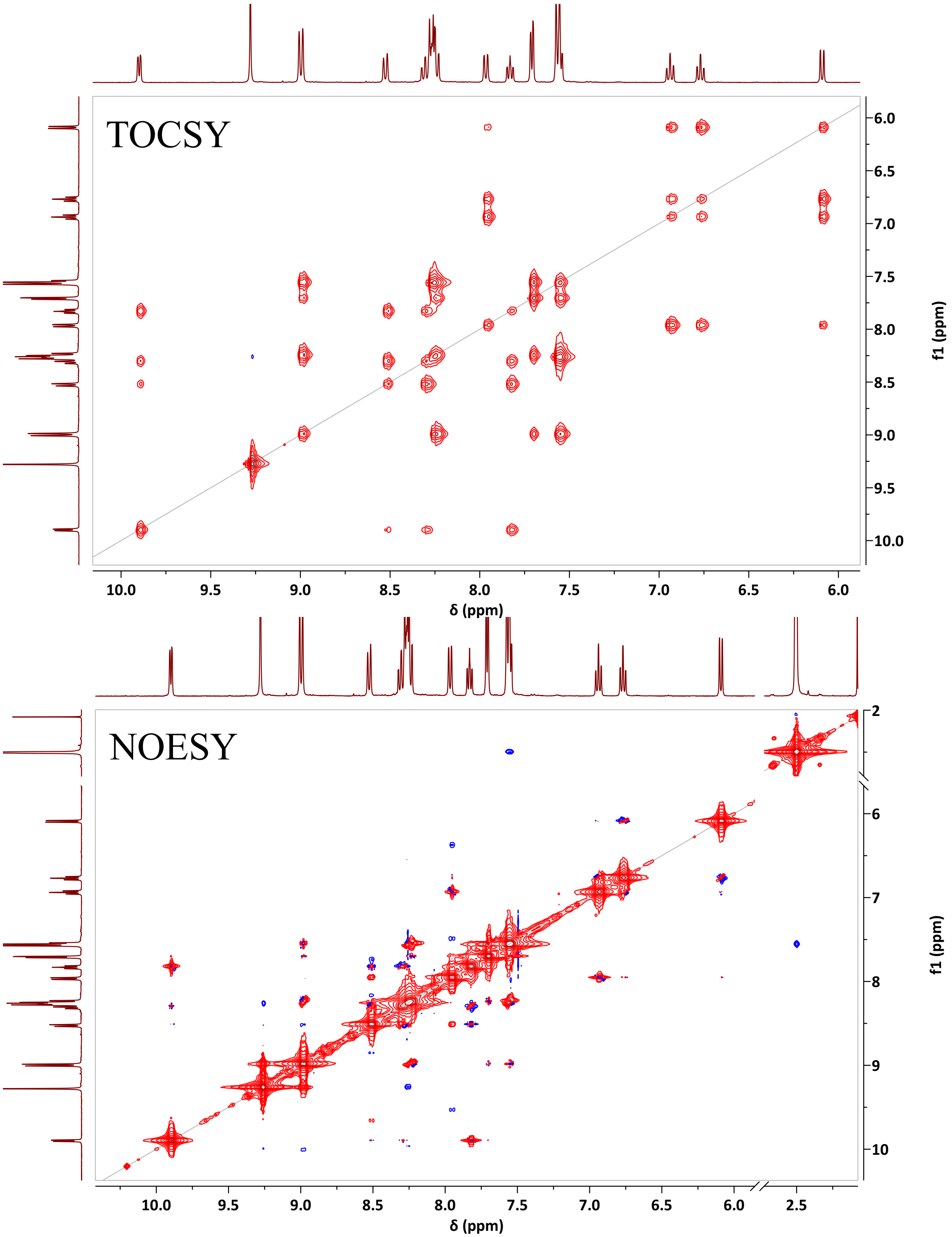

Figure S26: ^1H-^1H TOCSY spectrum (top) of [Ir(**4-MePhTerpy**)(ppy)Cl](PF_6) collected in DMSO-d_6 and the ^1H-^1H NOESY spectrum (bottom).

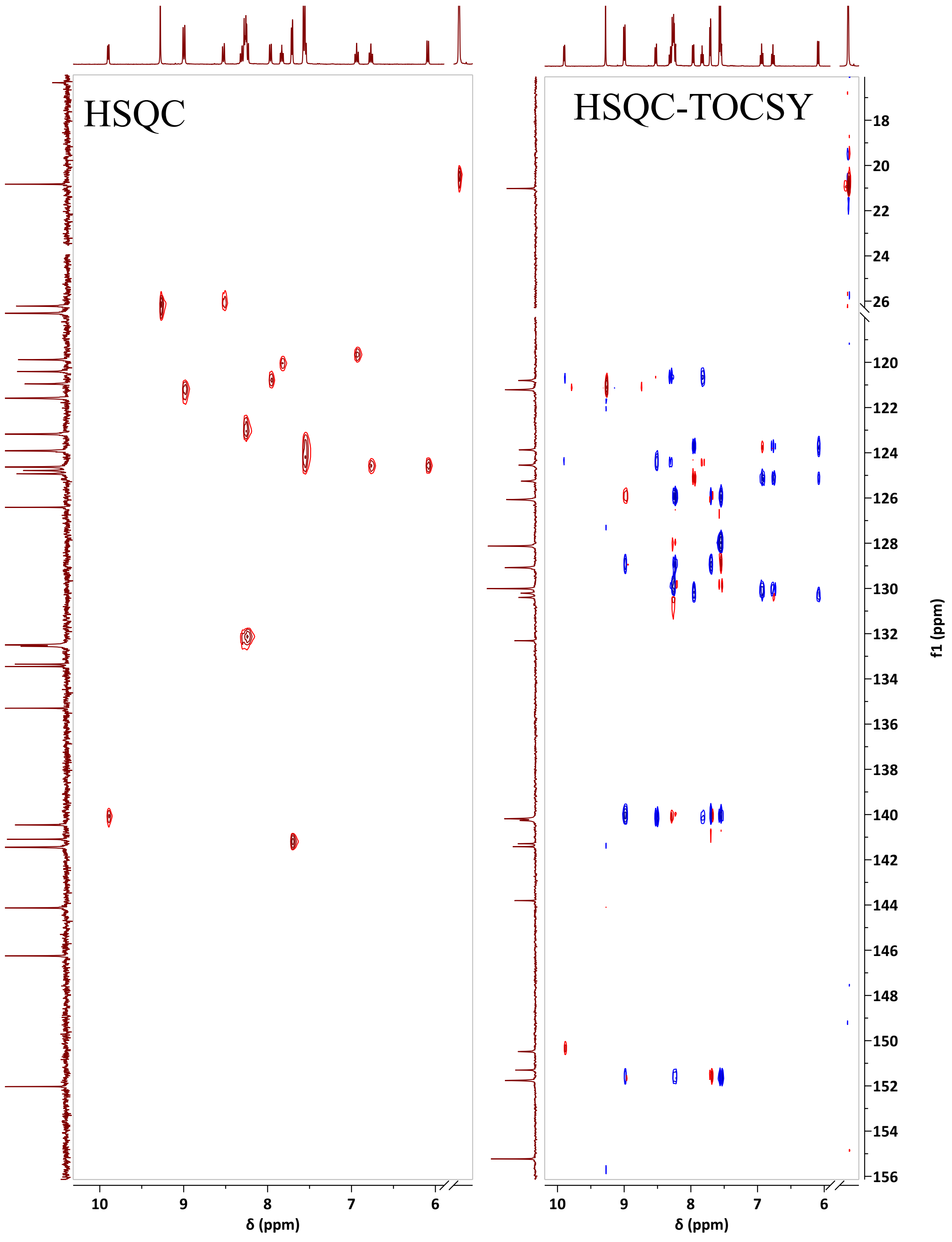

Figure S27: ^{1}H-^{13}C Edited-HSQC spectrum (left) and ^{1}H-^{13}C HSQC-TOCSY spectrum (right) of [Ir(**4-MePhTerpy**)(ppy)Cl](PF_6) collected in DMSO-d_6.

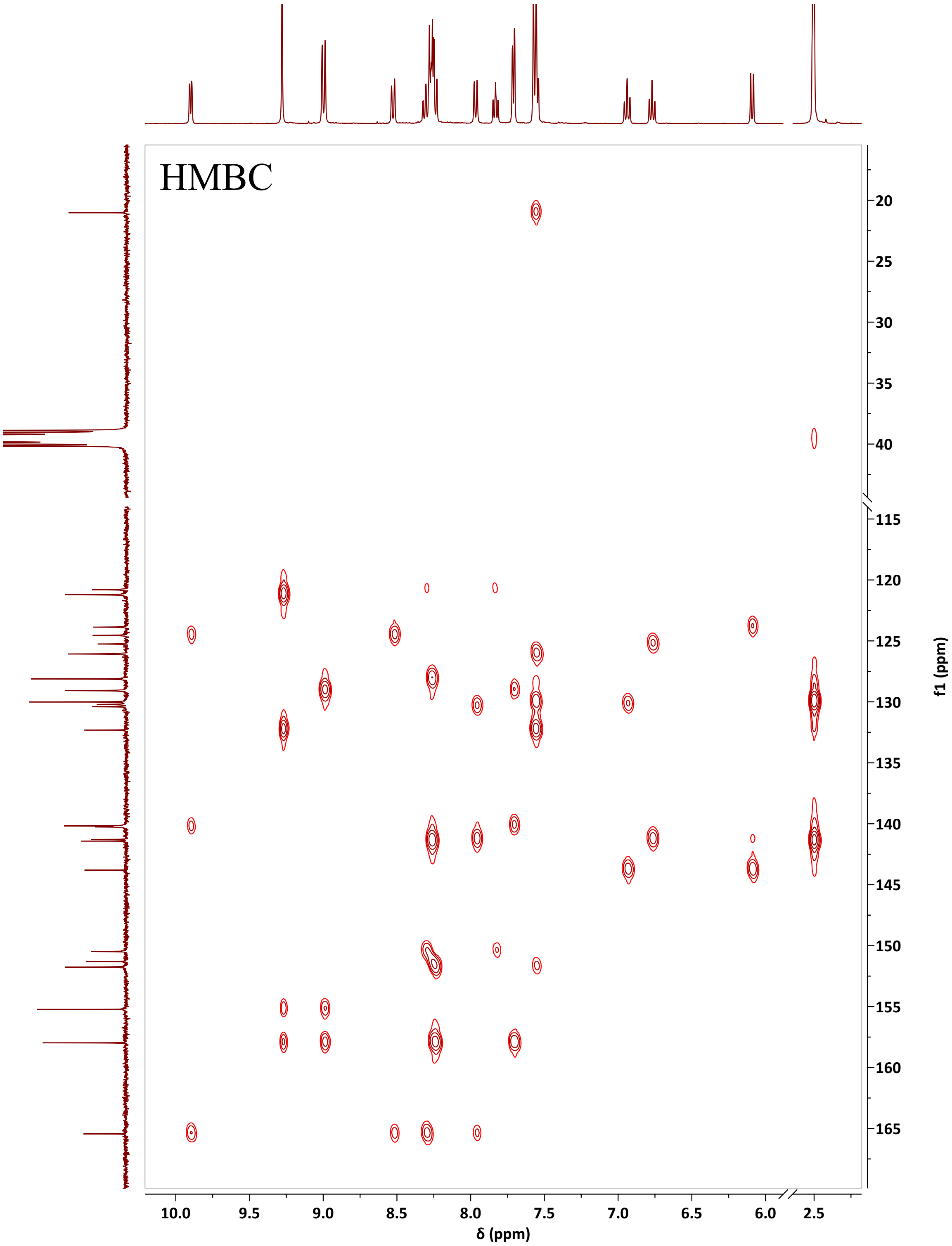

Figure S28: ^{1}H-^{13}C HMBC spectrum of [Ir(**4-MePhTerpy**)(ppy)Cl](PF_6) collected in DMSO-d_6.

4.6 NMR Data for [Ir(4-OMePhTerpy)(ppy)Cl](PF_6)

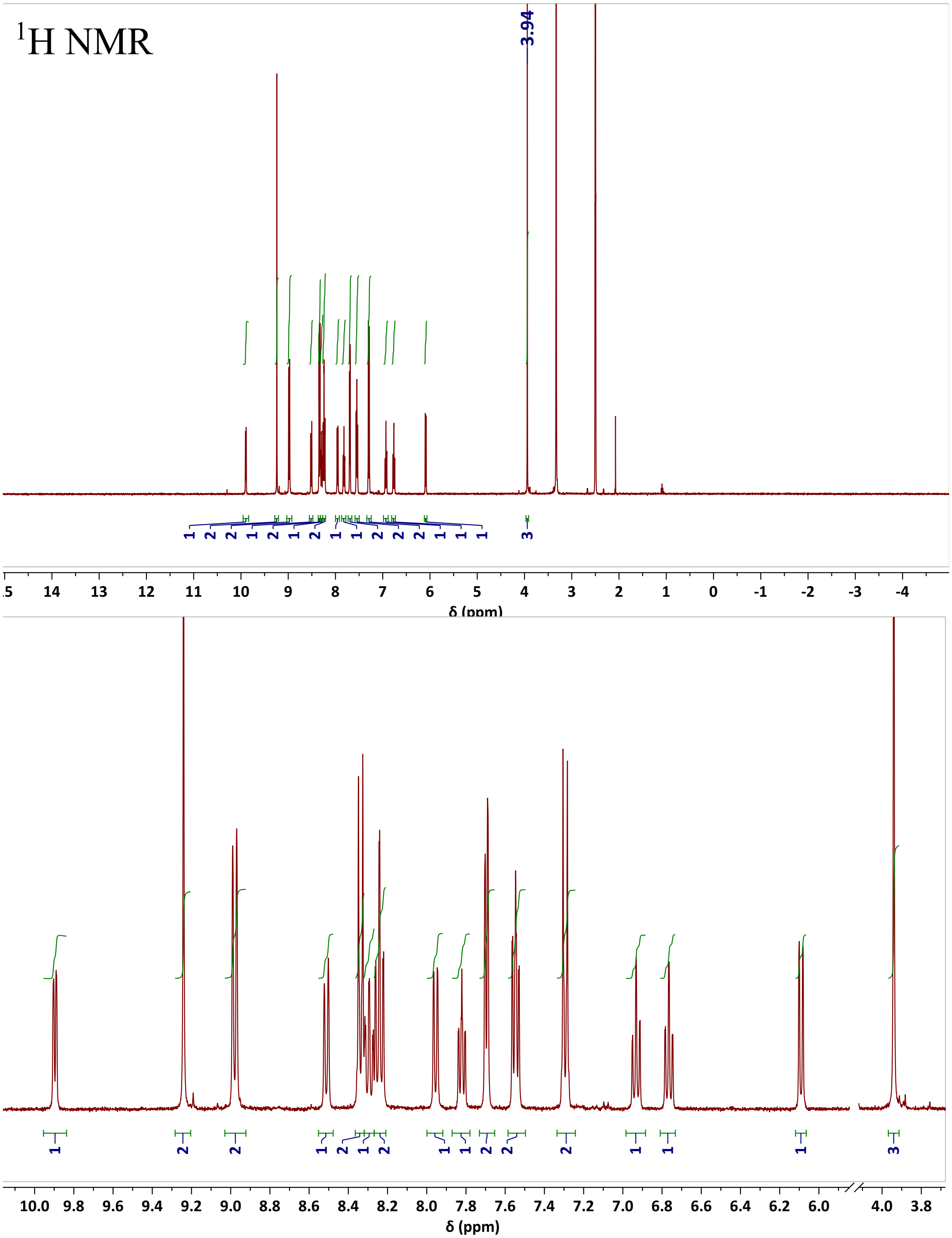

Figure S29: ^{1}H NMR Spectrum (top left) and expanded ^{1}H NMR Spectrum (bottom) and ^{19}F NMR spectrum (top right) of 4-OMePhTerpy collected in DMSO-d_6.

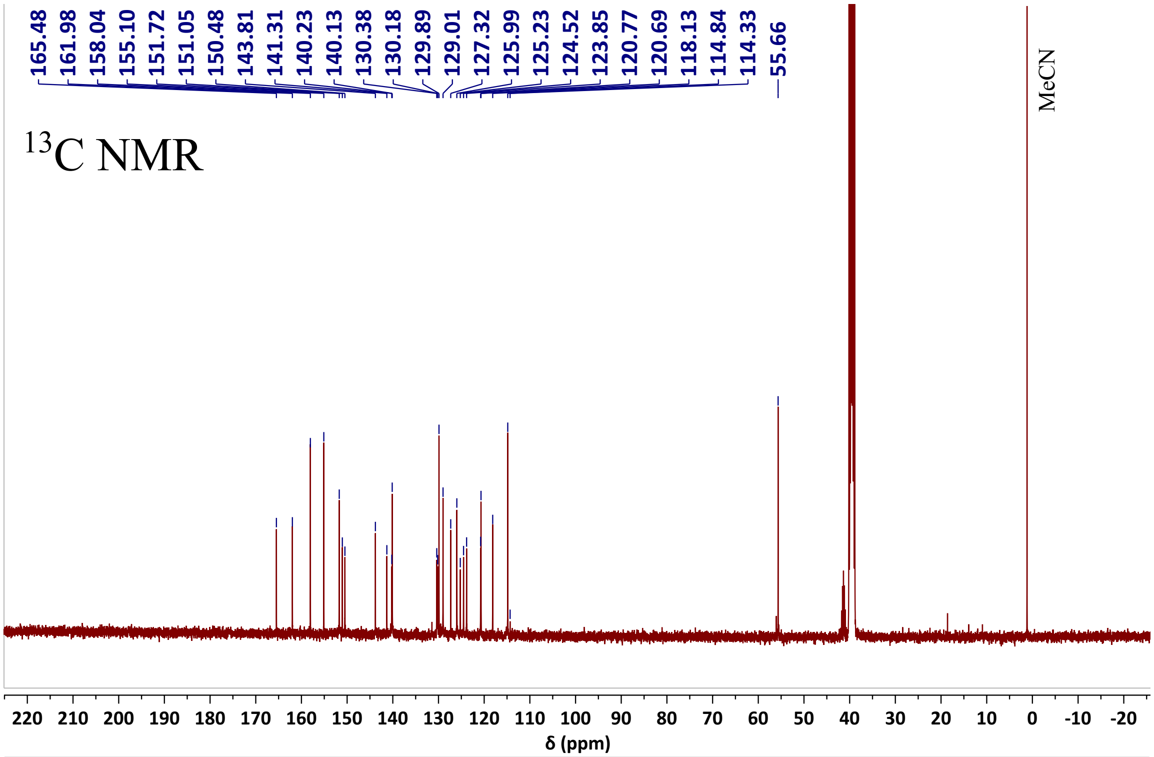

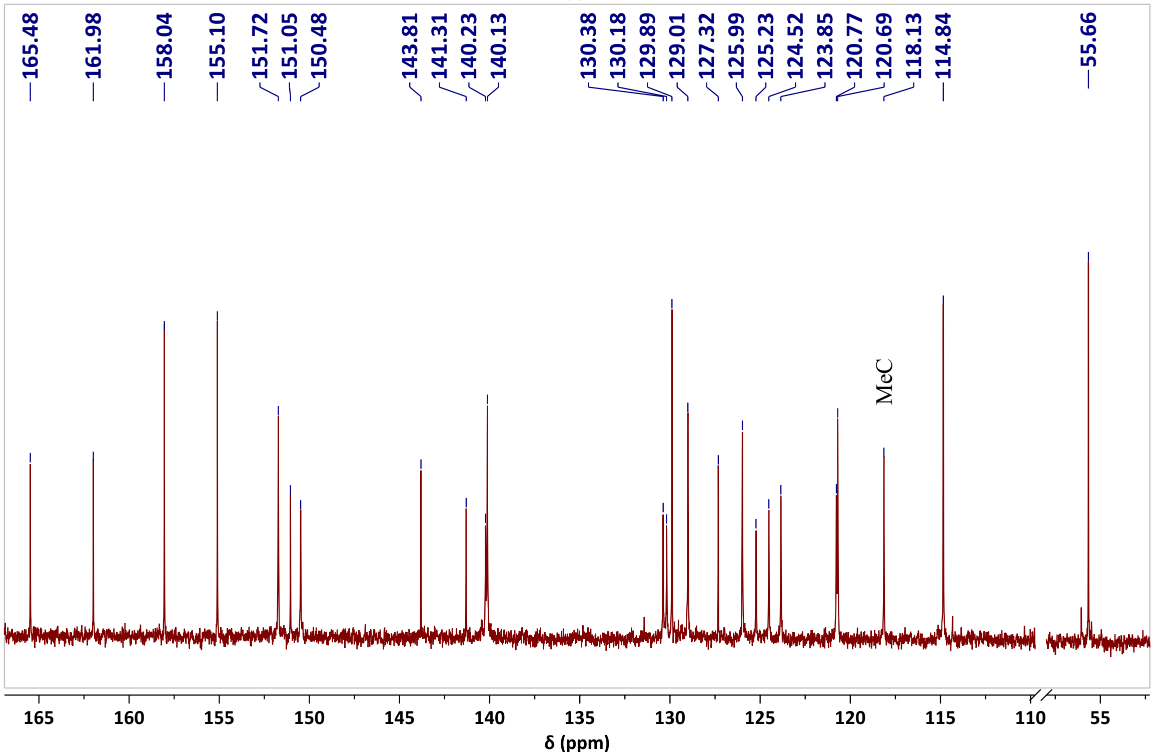

Figure S30: ^{13}C NMR Spectrum (top) and expanded ^{13}C NMR Spectrum (bottom) of [Ir(**4-OMePhTerpy**)(ppy)Cl](PF_6) collected in DMSO-d_6.

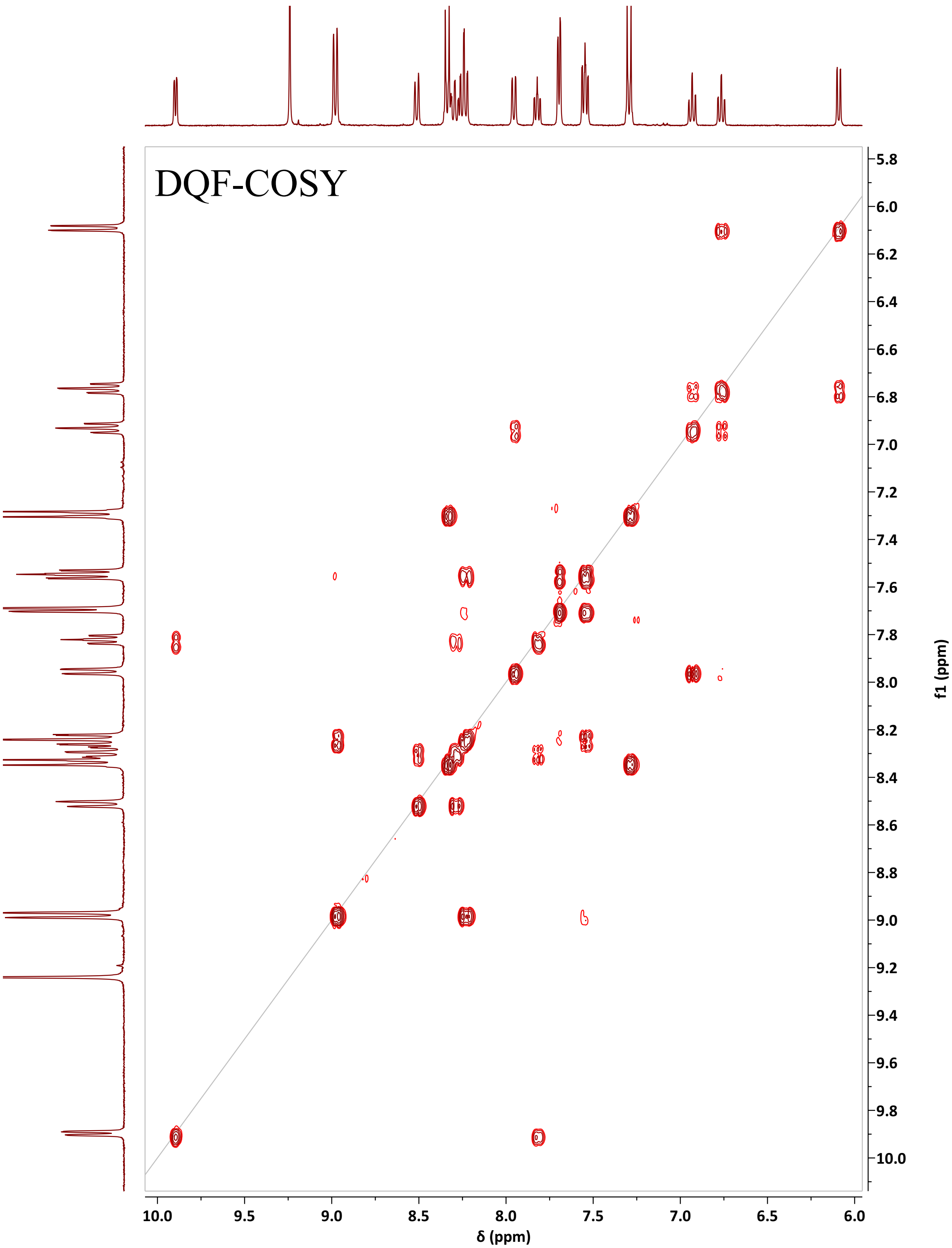

Figure S31: ¹H-¹H DQF-COSY spectrum of [Ir(**4-OMePhTerpy**)(ppy)Cl](PF_6) collected in DMSO-d_6.

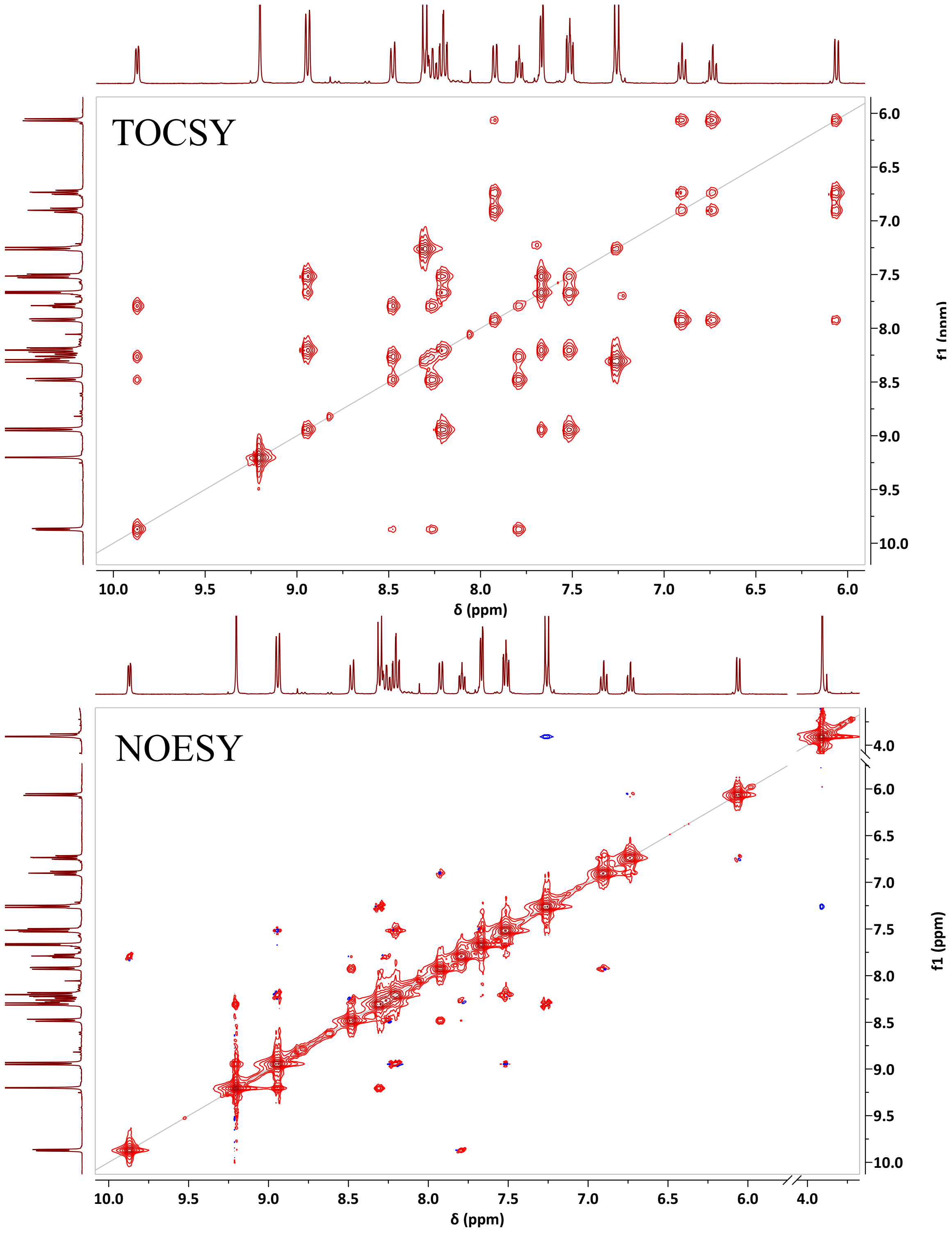

Figure S32: ^1H-^1H TOCSY spectrum (top) of [Ir(**4-OMePhTerpy**)(ppy)Cl](PF_6) collected in DMSO-d_6 and the ^1H-^1H NOESY spectrum (bottom).

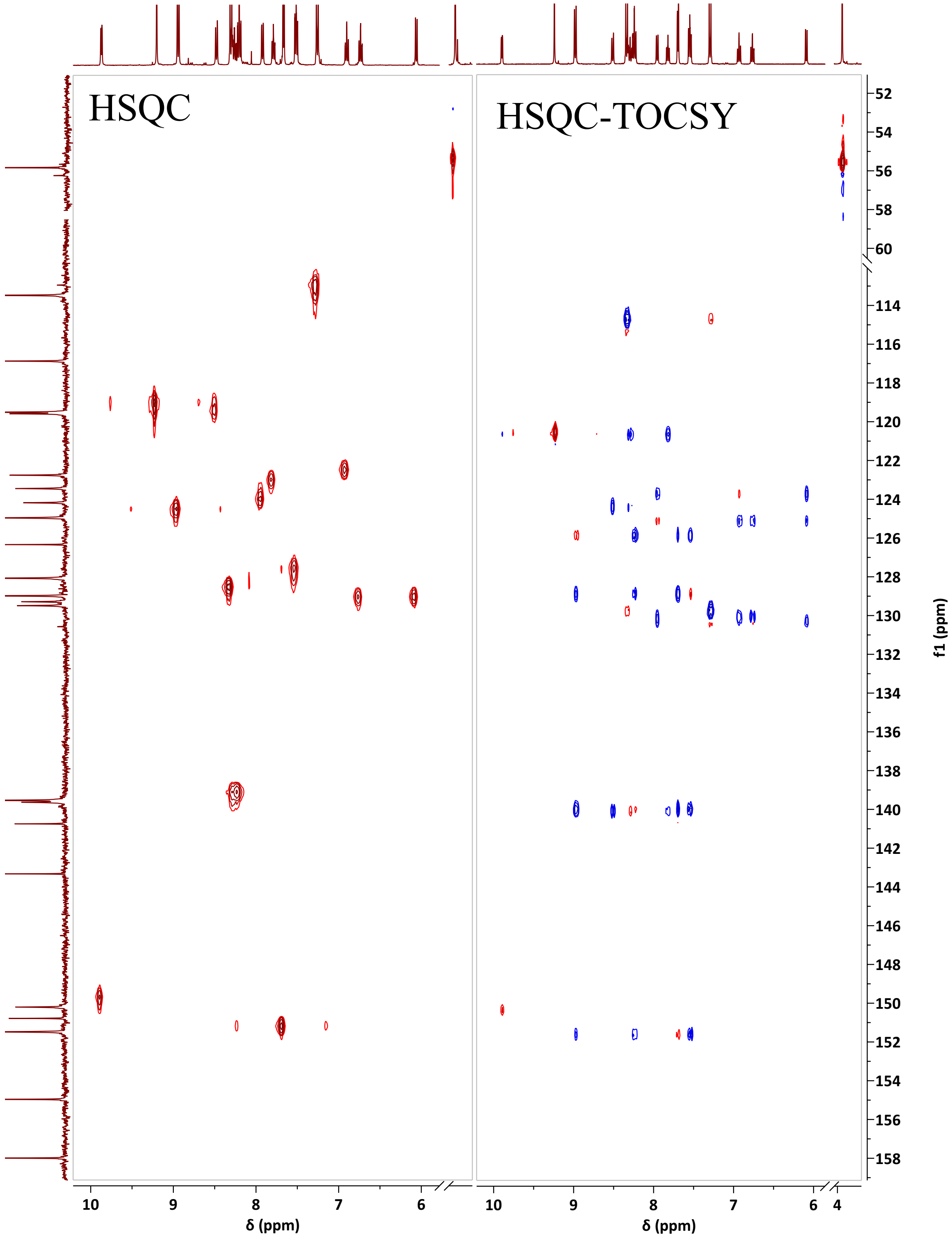

Figure S33: ^{1}H-^{13}C Edited-HSQC spectrum (left) and ^{1}H-^{13}C HSQC-TOCSY spectrum (right) of [Ir(**4-OMePhTerpy**)(ppy)Cl](PF$_6$) collected in DMSO-d_6.

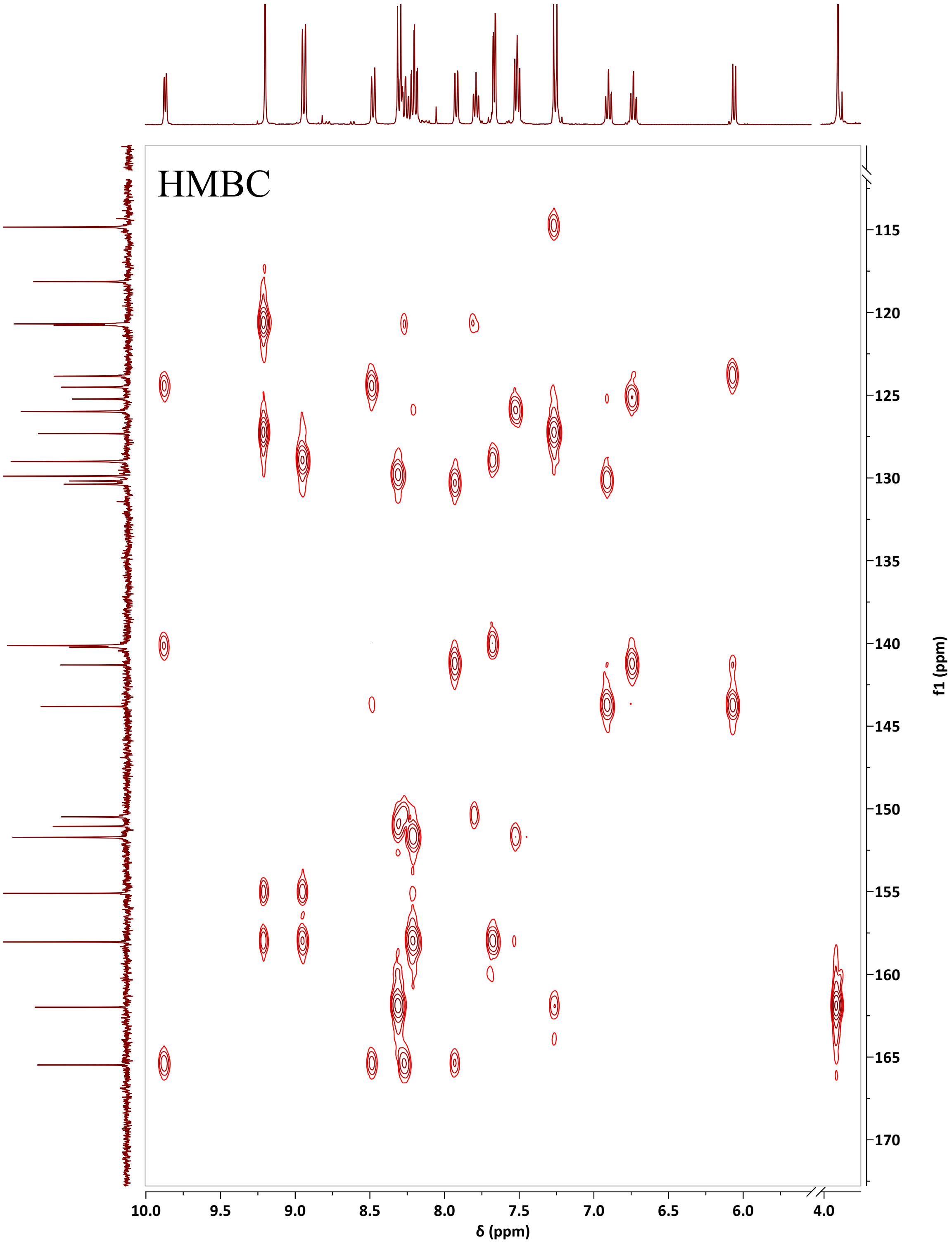

Figure S34: ^{1}H-^{13}C HMBC spectrum of [Ir(**4-OMePhTerpy**)(ppy)Cl](PF_6) collected in DMSO-d_6.

4.7 NMR Data for [Ir(4-NMe$_2$PhTerpy)(ppy)Cl](PF$_6$)

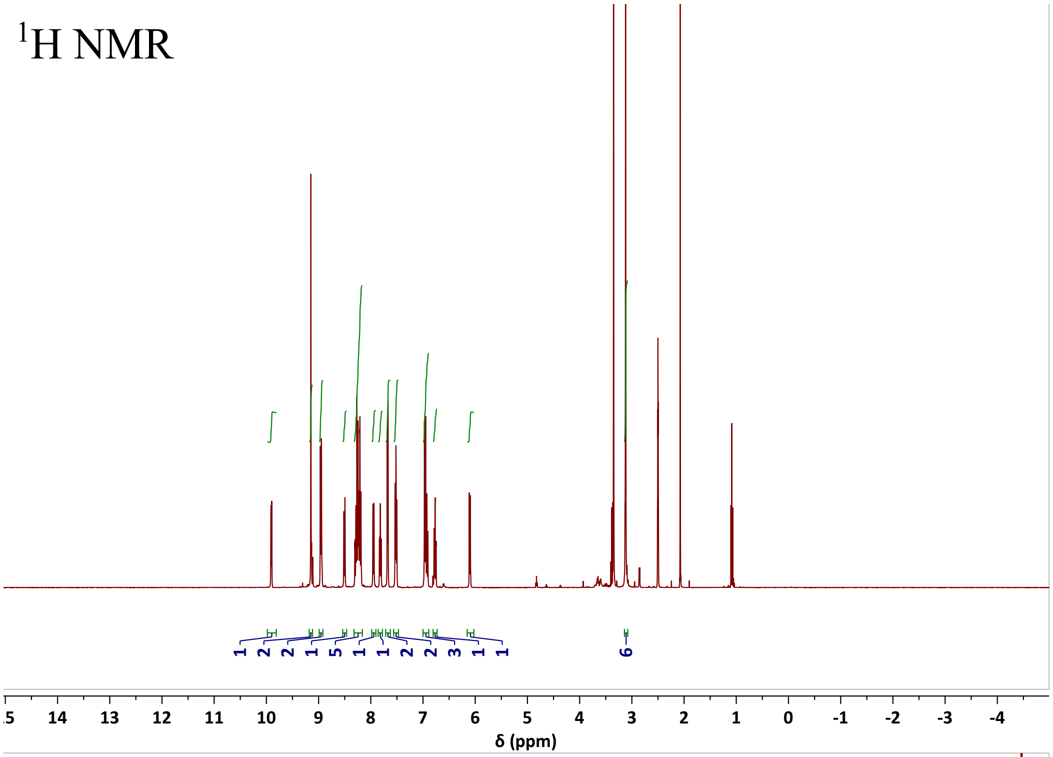

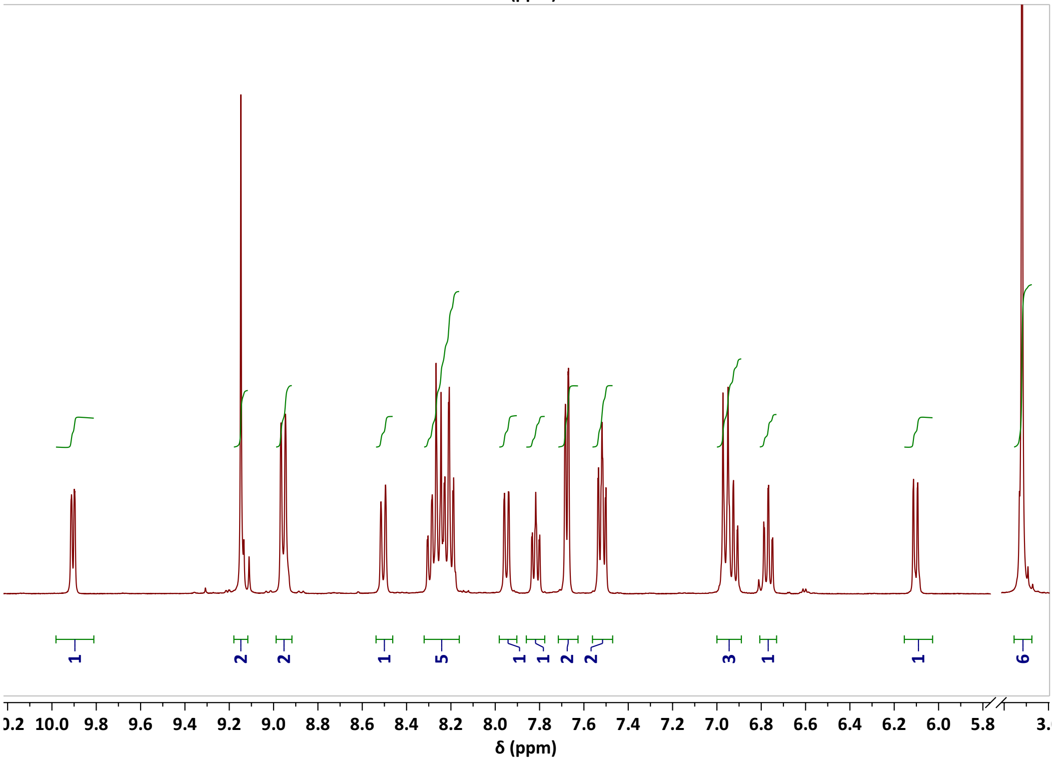

Figure S35: ^{1}H NMR Spectrum (top left) and expanded ^{1}H NMR Spectrum (bottom) and ^{19}F NMR spectrum (top right) of 4-NMe$_2$PhTerpy collected in DMSO-d_6.

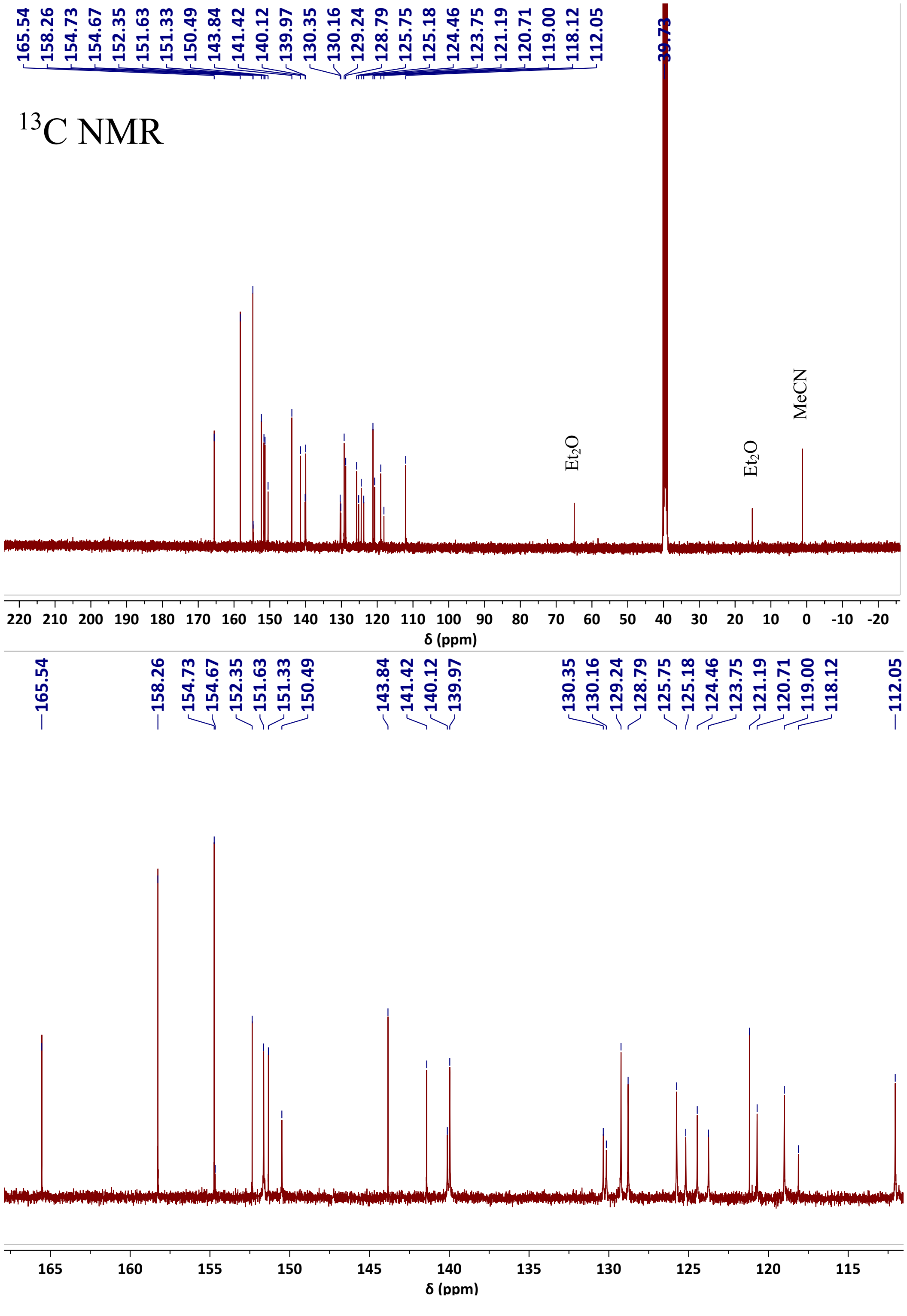

Figure S36: ^{13}C NMR Spectrum (top) and expanded ^{13}C NMR Spectrum (bottom) of [Ir(**4-NMe$_2$PhTerpy**)(ppy)Cl](PF$_6$) collected in DMSO-d_6.

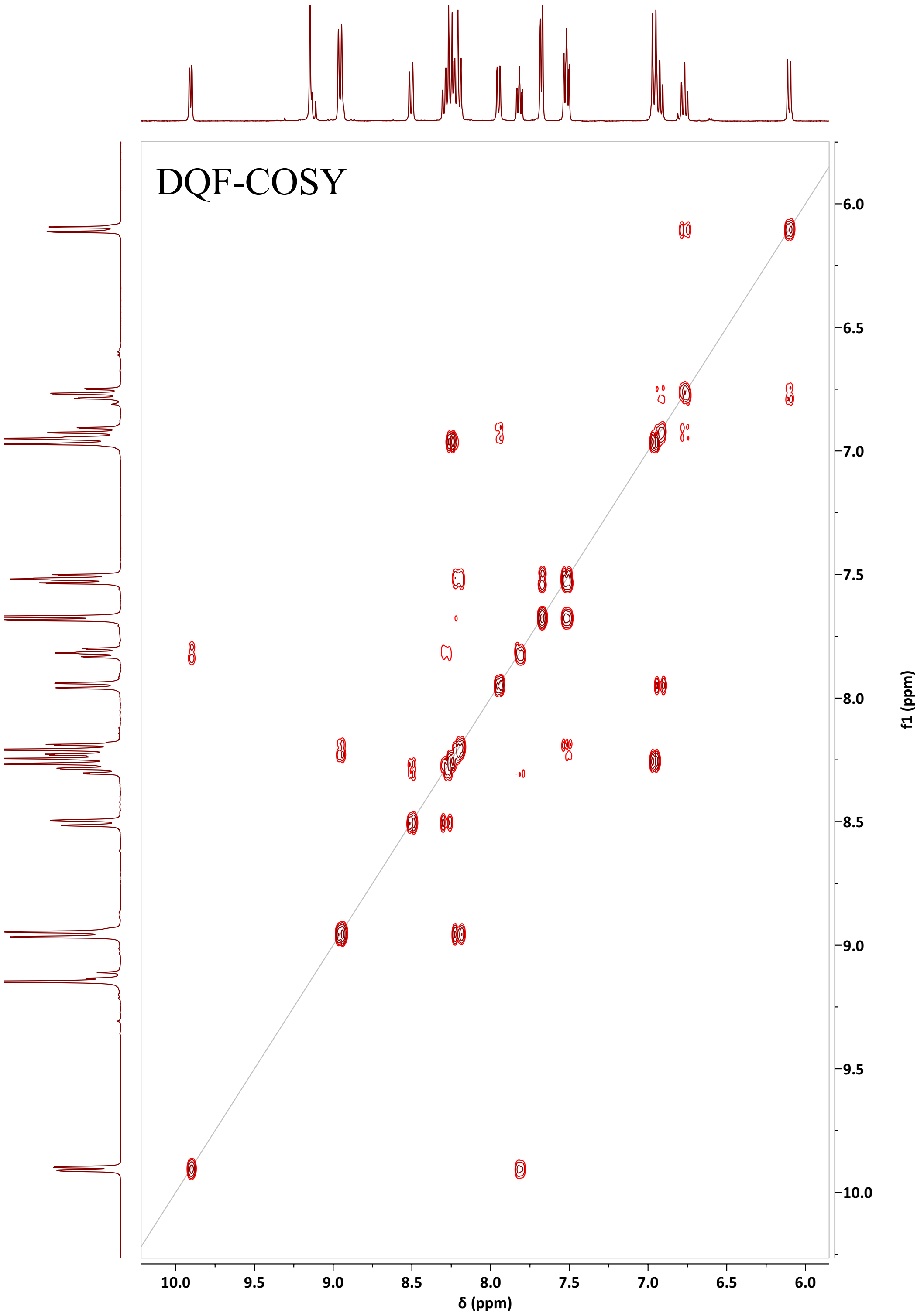

Figure S37: ^{1}H-^{1}H DQF-COSY spectrum of [Ir(**4-NMe$_2$PhTerpy**)(ppy)Cl](PF_6) collected in DMSO-d_6.

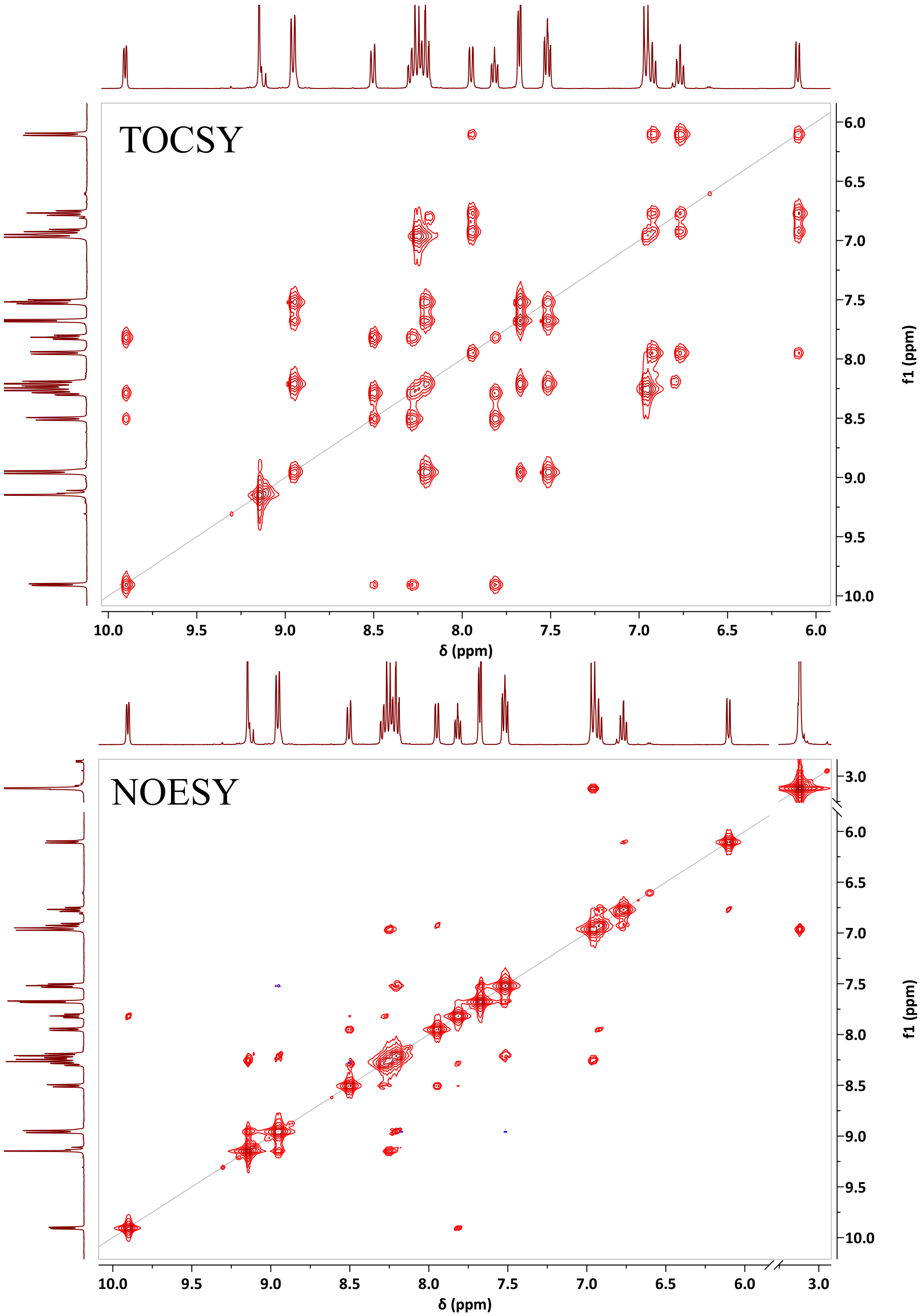

Figure S38: ^{1}H-^{1}H TOCSY spectrum (top) of [Ir(**4-NMe$_2$PhTerpy**)(ppy)Cl](PF_6) collected in DMSO-d_6 and the ^{1}H-^{1}H NOESY spectrum (bottom).

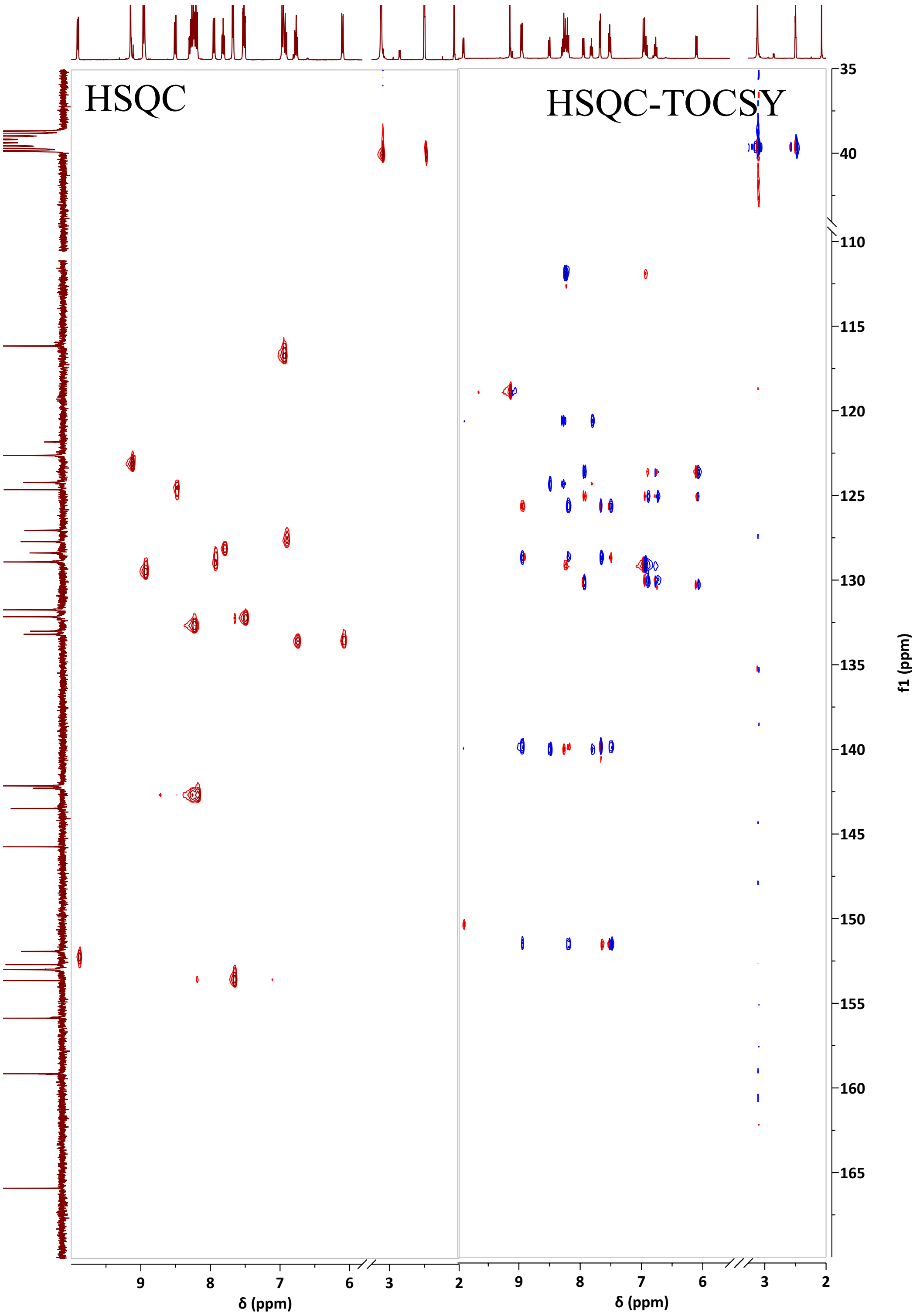

Figure S39: ^{1}H-^{13}C Edited-HSQC spectrum (left) and ^{1}H-^{13}C HSQC-TOCSY spectrum (right) of [Ir(**4-NMe$_2$PhTerpy**)(ppy)Cl](PF_6) collected in DMSO-d_6.

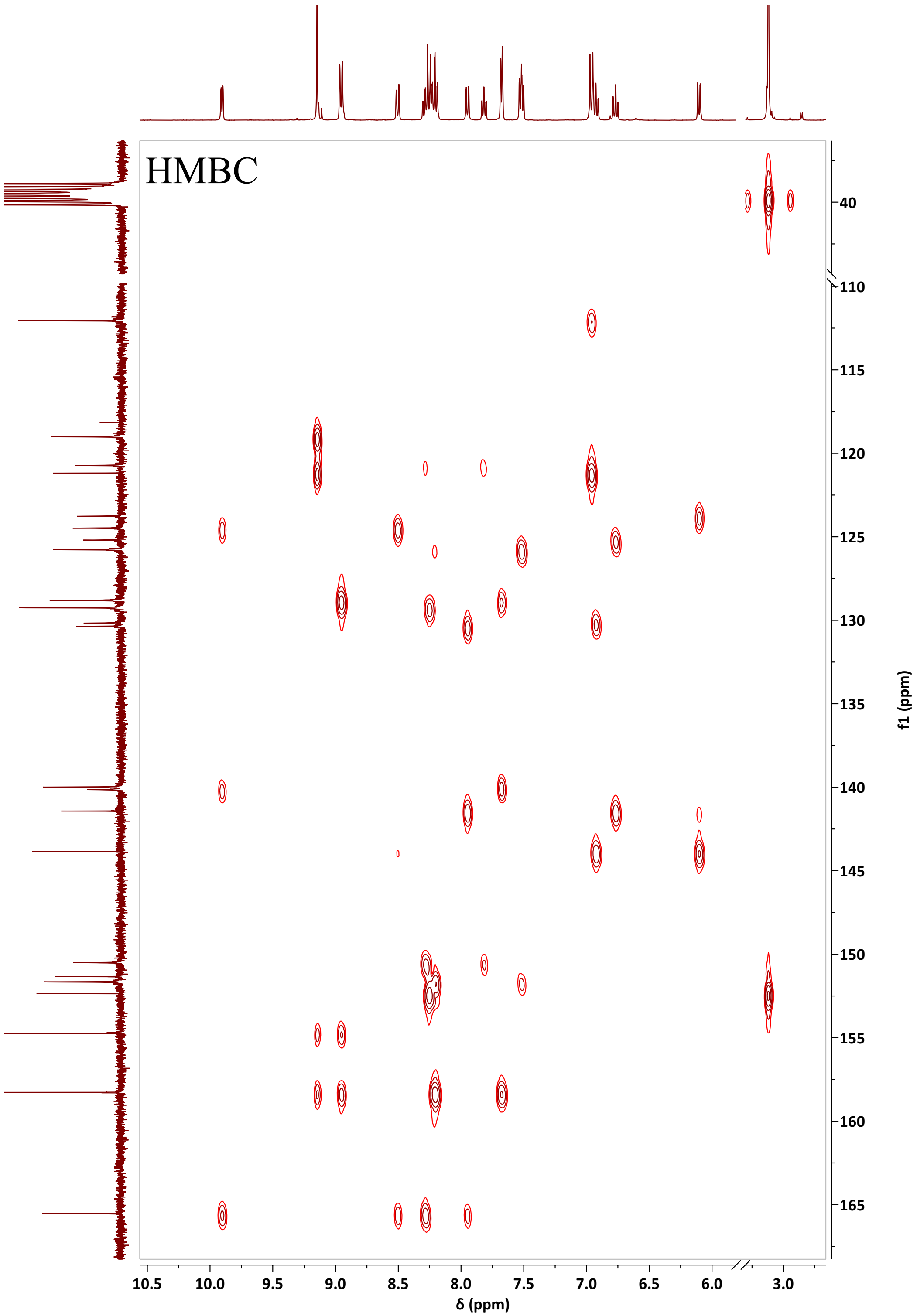

Figure S40: ^{1}H-^{13}C HMBC spectrum of [Ir(**4-NMe$_2$PhTerpy**)(ppy)Cl](PF_6) collected in DMSO-d_6.

5 Crystallographic Data

5.1 Additional Crystallography Narrative

Table S10: Comparison of select bond lengths (Å) and angles (°) of [Ir(**RPhTerpy**)(ppy)Cl](PF_6) complexes and related literature structures.[6–9] btp = 2-(2-pyridyl)benzo[b]thiophene; MeO-mppy = 2-(4-methoxyphenyl)-5-methylpyridine. [a] – the value is the average of the terminal Ir-N bonds. Standard deviations are in parentheses. [b] – The angle between the **RPhTerpy** terminal nitrogen atoms. [c] – Bond distance from iridium to the nitrogen atom of pyridine in ppy. [d] – Torsional angle between the aryl-ring and the central pyridine ring. [e] – Bond distance from iridium to the cyclometallated carbon atom of the phenyl ring in ppy.

**Compound**	**RPhTerpy**			**Phenyl pyridine**	
	Ir—N[a]	Ir—N[c]	Torsional Angle[d]	Ir—C	N[e]
[Ir(**3,5-CF$_3$PhTerpy**)(ppy)Cl](PF_6)	2.052(6)	1.963(4)	-45.3	2.073(4)	2.010(5)
[Ir(**4-CF$_3$PhTerpy**)(ppy)Cl](PF_6)	2.047(4)	1.947(3)	6.4	2.076(3)	2.023(4)
[Ir(**4-MePhTerpy**)(ppy)Cl](PF_6)	2.054(3)	1.960(2)	-7.0	2.088(3)	2.029(3)
[Ir(**4-OMePhTerpy**)(ppy)Cl](PF_6)	2.054(9)	1.972(4)	-9.9	2.081(4)	2.016(5)
[Ir(**4-NMe$_2$PhTerpy**)(ppy)Cl](PF_6)	2.062(4)	1.961(4)	14.4	2.083(4)	2.016(4)
[Ir(**PhTerpy**)(ppy)Cl](PF_6)[6]	2.048(14)	1.936(7)	-26.1	2.058(8)	2.014(9)
[Ir(**terpy**)(MeO-mppy)Cl](PF_6)[7]	2.038(3)	1.944(2)	–	2.068(2)	2.021(2)
[Ir(**terpy**)(ppy)H](PF_6)[8]	2.025(13)	2.002(8)	–	2.12(1)	2.02(1)
[Ir(**terpy**)(ppy)(OH)](PF_6)[8]	2.046(13)	2.011(8)	–	2.08(1)	2.04(1)
[Ir(**terpy**)(btp)Cl](PF_6)[9]	2.046(6)	1.951(4)	–	2.087(4)	2.031(6)

5.2 [Ir(3,5-CF_3PhTerpy)(ppy)Cl](PF_6)

Crystal Data for $IrC_{34}H_{21}N_4F_6ClPF_6$ (M =972.17 g/mol): monoclinic, space group $P2_1/n$ (no. 14), a = 8.9095(2) Å, b = 18.6633(5) Å, c = 22.9429(6) Å, β = 93.878(2)°, V = 3806.23(17) Å^3, Z = 4, T = 170.0(1) K, μ(Mo K$_\alpha$) = 3.709 mm^{-1}, D_{calc} = 1.697 g/cm^3, 39360 reflections measured (4.174° ≤ 2θ ≤ 54.958°), 8734 unique (R_{int} = 0.0479, R_{sigma} = 0.0578) which were used in all calculations. The final R_1 was 0.0364 (I > 2σ(I)) and wR_2 was 0.0896 (all data).

Table S11: Crystal data and structure refinement for [Ir(**3,5-CF_3PhTerpy**)(ppy)Cl](PF_6).

Empirical formula	$IrC_{34}H_{21}N_4F_6ClPF_6$
Formula weight	972.17
Temperature/K	170.0(1)
Crystal system	monoclinic
Space group	$P2_1/n$
a/Å	8.9095(2)
b/Å	18.6633(5)
c/Å	22.9429(6)
α/°	90
β/°	93.878(2)
γ/°	90
Volume/Å^3	3806.23(17)
Z	4
ρ_{calc}g/cm^3	1.697
μ/mm^{-1}	3.709
F(000)	1880.0
Crystal size/mm^3	0.37 × 0.11 × 0.07
Radiation	Mo K$_\alpha$ (λ = 0.71073)
2θ range for data collection/°	4.174 to 54.958
Index ranges	-11 ≤ h ≤ 11, -24 ≤ k ≤ 23, -26 ≤ l ≤ 29
Reflections collected	39360
Independent reflections	8734 [R_{int} = 0.0479, R_{sigma} = 0.0578]
Data/restraints/parameters	8734/1239/588
Goodness-of-fit on F^2	0.996
Final R indexes [I>=2σ (I)]	R_1 = 0.0364, wR_2 = 0.0830
Final R indexes [all data]	R_1 = 0.0634, wR_2 = 0.0896
Largest diff. peak/hole / e Å^{-3}	1.34/-0.74

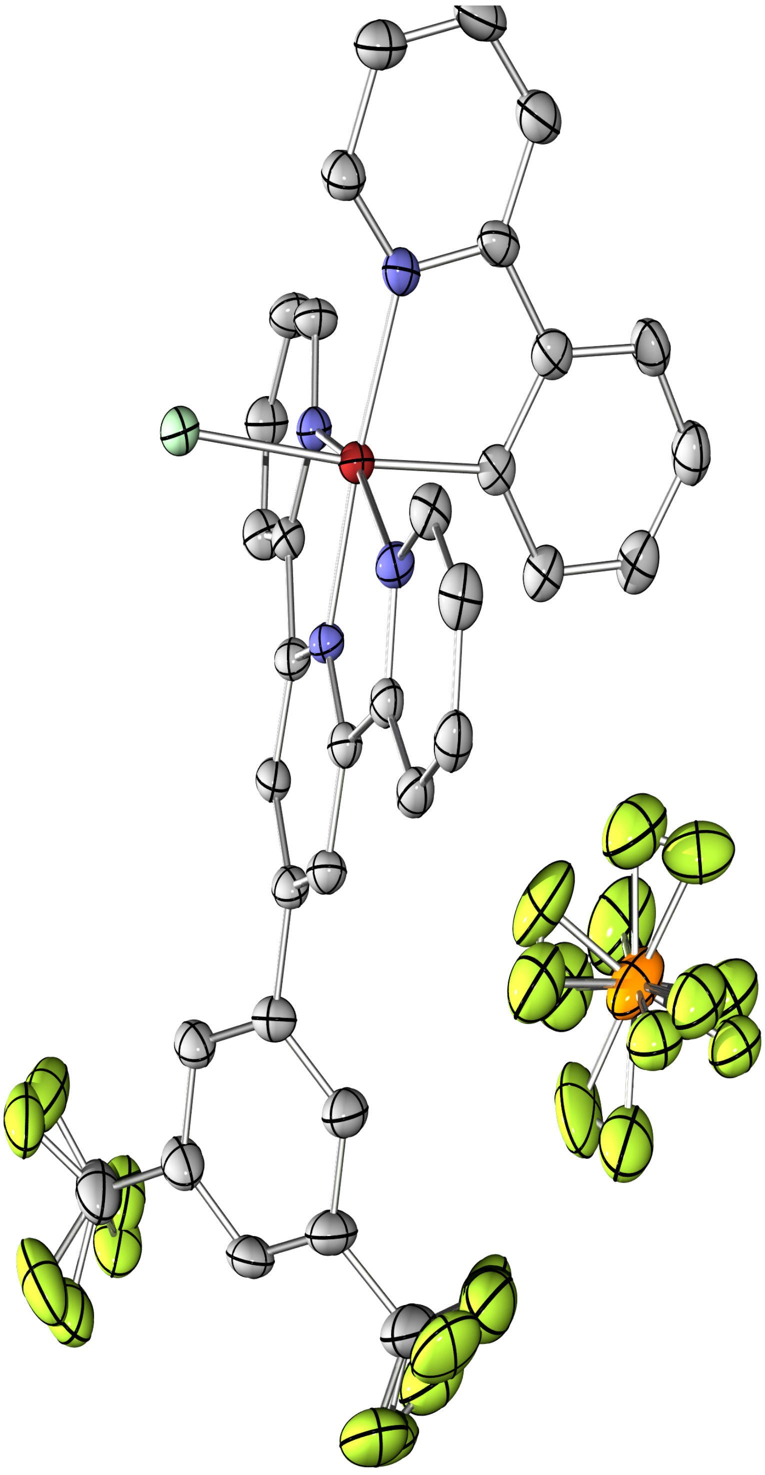

Figure S41: ORTEP projection of [Ir(**3,5-CF$_3$PhTerpy**)(ppy)Cl](PF$_6$) rendered with POV-RAY at the at the 40% thermal ellipsoid level. Carbon atoms are grey, nitrogen atoms are light blue, iridium atoms are dark red, chlorine atoms are pale green, phosphorus atoms are orange.

5.3 [Ir(4-CF_3PhTerpy)(ppy)Cl](PF_6)

Crystal Data for $IrN_4C_{33}H_{22}F_3ClPF_6$ (M =904.16 g/mol): monoclinic, space group P2$_1$/c (no. 14), a = 13.1170(2) Å, b = 19.6888(3) Å, c = 12.4057(2) Å, β = 100.6640(10)°, V = 3148.54(9) Å^3, Z = 4, T = 170.0(1) K, μ(Mo K$_\alpha$) = 4.462 mm^{-1}, D_{calc} = 1.907 g/cm^3, 60152 reflections measured (3.16° ≤ 2θ ≤ 54.968°), 7221 unique (R_{int} = 0.0387, R$_{\text{sigma}}$ = 0.0292) which were used in all calculations. The final R_1 was 0.0300 (I > 2σ(I)) and wR_2 was 0.0568 (all data).

Table S12: Crystal data and structure refinement for [Ir(**4-CF_3PhTerpy**)(ppy)Cl](PF_6).

Empirical formula	$C_{33}H_{22}N_4F_9PClIr$
Formula weight	904.16
Temperature/K	170.0(1)
Crystal system	monoclinic
Space group	P2$_1$/c
a/Å	13.1170(2)
b/Å	19.6888(3)
c/Å	12.4057(2)
α/°	90
β/°	100.6640(10)
γ/°	90
Volume/Å^3	3148.54(9)
Z	4
ρ_{calc}g/cm^3	1.907
μ/mm^{-1}	4.462
F(000)	1752.0
Crystal size/mm^3	0.126 × 0.073 × 0.035
Radiation	Mo K$_\alpha$ (λ = 0.71073)
2θ range for data collection/°	3.16 to 54.968
Index ranges	-17 ≤ h ≤ 17, -25 ≤ k ≤ 25, -16 ≤ l ≤ 15
Reflections collected	60152
Independent reflections	7221 [R$_{\text{int}}$ = 0.0387, R$_{\text{sigma}}$ = 0.0292]
Data/restraints/parameters	7221/594/533
Goodness-of-fit on F^2	1.079
Final R indexes [I>=2σ (I)]	R$_1$ = 0.0300, wR$_2$ = 0.0542
Final R indexes [all data]	R$_1$ = 0.0418, wR$_2$ = 0.0568
Largest diff. peak/hole / e Å^{-3}	0.81/-0.99

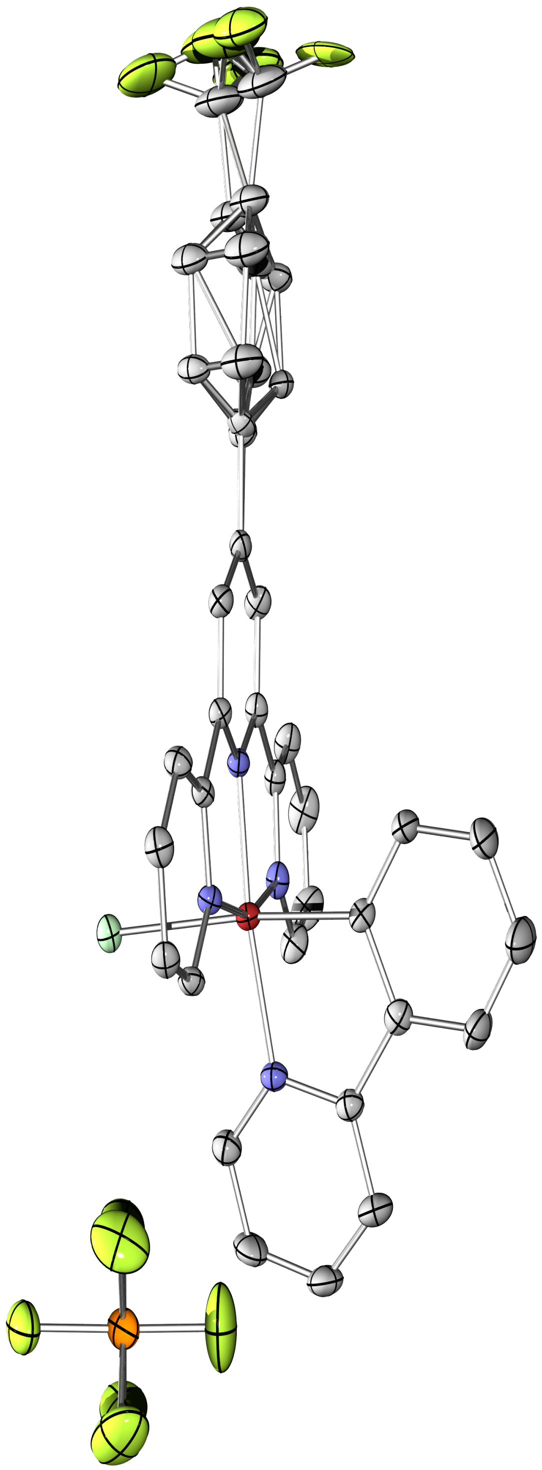

Figure S42: ORTEP projection of [Ir(**4-CF$_3$PhTerpy**)(ppy)Cl](PF_6) rendered with POV-RAY at the at the 40% thermal ellipsoid level. Carbon atoms are grey, nitrogen atoms are light blue, iridium atoms are dark red, chlorine atoms are pale green, phosphorus atoms are orange.

5.4 [Ir(4-MePhTerpy)(ppy)Cl](PF_6)

Crystal Data for $IrC_{33}H_{25}N_4ClPF_6$ (M =850.19 g/mol): triclinic, space group P-1 (no. 2), a = 11.8508(3) Å, b = 11.9997(3) Å, c = 13.9656(3) Å, α = 110.159(2)°, β = 91.464(2)°, γ = 102.966(2)°, V = 1805.27(8) Å^3, Z = 2, T = 170.0(1) K, μ(Mo K_α) = 3.874 mm^{-1}, D_{calc} = 1.564 g/cm^3, 32354 reflections measured (3.126° $\leq 2\theta \leq$ 54.968°), 8195 unique (R_{int} = 0.0472, R_{sigma} = 0.0438) which were used in all calculations. The final R_1 was 0.0269 (I > 2σ(I)) and wR_2 was 0.0611 (all data).

Table S13: Crystal data and structure refinement for [Ir(**4-MePhTerpy**)(ppy)Cl](PF_6).

Empirical formula	$IrC_{33}H_{25}N_4ClPF_6$
Formula weight	850.19
Temperature/K	170.0(1)
Crystal system	triclinic
Space group	P-1
a/Å	11.8508(3)
b/Å	11.9997(3)
c/Å	13.9656(3)
α/°	110.159(2)
β/°	91.464(2)
γ/°	102.966(2)
Volume/Å^3	1805.27(8)
Z	2
ρ_{calc}g/cm^3	1.564
μ/mm^{-1}	3.874
F(000)	828.0
Crystal size/mm^3	0.43 × 0.19 × 0.1
Radiation	Mo K_α (λ = 0.71073)
2θ range for data collection/°	3.126 to 54.968
Index ranges	$-15 \leq h \leq 15$, $-15 \leq k \leq 15$, $-18 \leq l \leq 18$
Reflections collected	32354
Independent reflections	8195 [R_{int} = 0.0472, R_{sigma} = 0.0438]
Data/restraints/parameters	8195/6/426
Goodness-of-fit on F^2	1.021
Final R indexes [I>=2σ (I)]	R_1 = 0.0269, wR_2 = 0.0595
Final R indexes [all data]	R_1 = 0.0334, wR_2 = 0.0611
Largest diff. peak/hole / e Å^{-3}	0.87/-1.21

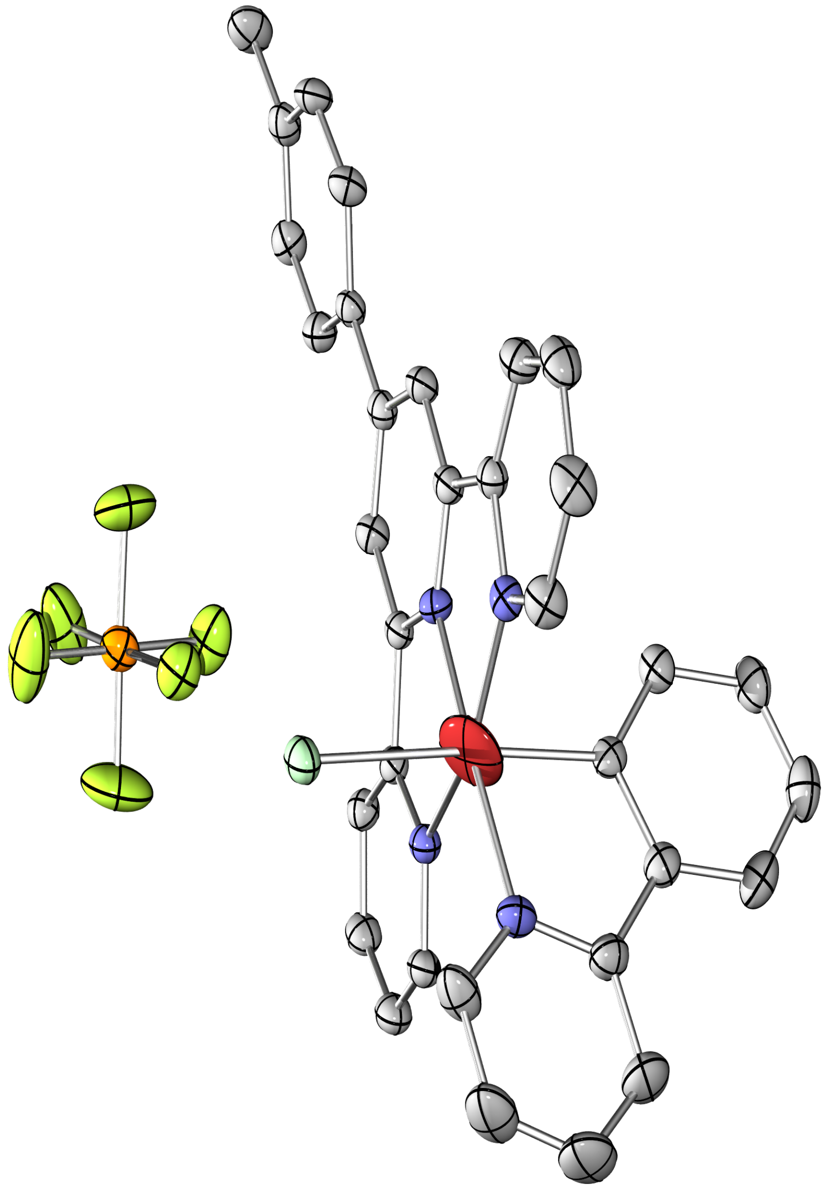

Figure S43: ORTEP projection of [Ir(**4-MePhTerpy**)(ppy)Cl](PF_6) rendered with POV-RAY at the at the 40% thermal ellipsoid level. Carbon atoms are grey, nitrogen atoms are light blue, iridium atoms are dark red, chlorine atoms are pale green, phosphorus atoms are orange.

5.5 [Ir(4-OMePhTerpy)(ppy)Cl](PF_6)

Crystal Data for $IrC_{33}H_{25}N_4OClPF_6$ (M =866.19 g/mol): triclinic, space group P-1 (no. 2), a = 9.0621(5) Å, b = 18.7091(7) Å, c = 21.7395(4) Å, α = 110.662(3)°, β = 95.463(4)°, γ = 99.377(4)°, V = 3355.8(2) Å^3, Z = 4, T = 170.0(1) K, μ(Mo Kα) = 4.172 mm^{-1}, D_{calc} = 1.714 g/cm^3, 61086 reflections measured (3.86° ≤ 2θ ≤ 54.968°), 15219 unique (R_{int} = 0.0569, R_{sigma} = 0.0629) which were used in all calculations. The final R_1 was 0.0427 (I > 2σ(I)) and wR_2 was 0.1076 (all data).

Table S14: Crystal data and structure refinement for [Ir(**4-OMePhTerpy**)(ppy)Cl](PF_6).

Empirical formula	$IrC_{33}H_{25}N_4OClPF_6$
Formula weight	866.19
Temperature/K	170.0(1)
Crystal system	triclinic
Space group	P-1
a/Å	9.0621(5)
b/Å	18.7091(7)
c/Å	21.7395(4)
α/°	110.662(3)
β/°	95.463(4)
γ/°	99.377(4)
Volume/Å^3	3355.8(2)
Z	4
ρ_{calc}g/cm^3	1.714
μ/mm^{-1}	4.172
F(000)	1688.0
Crystal size/mm^3	0.412 × 0.103 × 0.046
Radiation	Mo Kα (λ = 0.71073)
2θ range for data collection/°	3.86 to 54.968
Index ranges	-11 ≤ h ≤ 11, -24 ≤ k ≤ 23, -28 ≤ l ≤ 28
Reflections collected	61086
Independent reflections	15219 [R_{int} = 0.0569, R_{sigma} = 0.0629]
Data/restraints/parameters	15219/1847/1026
Goodness-of-fit on F^2	1.056
Final R indexes [I>=2σ (I)]	R_1 = 0.0427, wR_2 = 0.1008
Final R indexes [all data]	R_1 = 0.0655, wR_2 = 0.1076
Largest diff. peak/hole / e Å^{-3}	1.71/-1.39

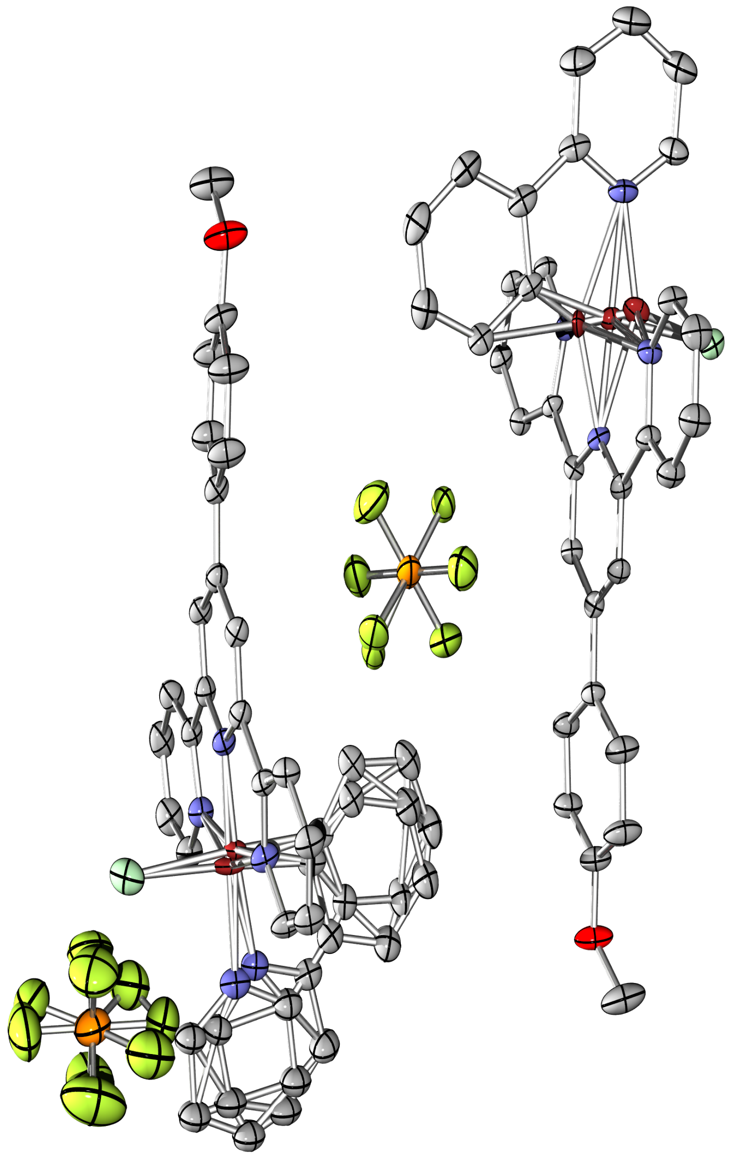

Figure S44: ORTEP projection of [Ir(**4-OMePhTerpy**)(ppy)Cl](PF_6) rendered with POV-RAY at the at the 40% thermal ellipsoid level. Carbon atoms are grey, nitrogen atoms are light blue, oxygen atoms are red, iridium atoms are dark red, chlorine atoms are pale green, phosphorus atoms are orange.

5.6 [Ir(4-NMe$_2$PhTerpy)(ppy)Cl](PF$_6$)

Crystal Data for IrN$_6$C$_{36}$H$_{31}$ClPF$_6$ (M =920.29 g/mol): triclinic, space group P-1 (no. 2), a = 11.4748(4) Å, b = 13.8788(5) Å, c = 14.0508(3) Å, α = 63.991(3)°, β = 70.671(3)°, γ = 74.745(3)°, V = 1879.25(12) Å^3, Z = 2, T = 169.99(10) K, μ(Mo Kα) = 3.730 mm^{-1}, D_{calc} = 1.626 g/cm^3, 31581 reflections measured (3.298° ≤ 2θ ≤ 54.968°), 8533 unique (R_{int} = 0.0622, R$_{\text{sigma}}$ = 0.0724) which were used in all calculations. The final R_1 was 0.0349 (I > 2σ(I)) and wR_2 was 0.0819 (all data).

Table S15: Crystal data and structure refinement for [Ir(**4-NMe$_2$PhTerpy**)(ppy)Cl](PF$_6$).

Empirical formula	C$_{36}$H$_{31}$ClF$_6$IrN$_6$P
Formula weight	920.29
Temperature/K	170.0(1)
Crystal system	triclinic
Space group	P-1
a/Å	11.4748(4)
b/Å	13.8788(5)
c/Å	14.0508(3)
α/°	63.991(3)
β/°	70.671(3)
γ/°	74.745(3)
Volume/Å^3	1879.25(12)
Z	2
ρ_{calc}g/cm^3	1.626
μ/mm^{-1}	3.730
F(000)	904.0
Crystal size/mm^3	0.288 × 0.158 × 0.078
Radiation	Mo Kα (λ = 0.71073)
2θ range for data collection/°	3.298 to 54.968
Index ranges	-14 ≤ h ≤ 14, -17 ≤ k ≤ 18, -18 ≤ l ≤ 18
Reflections collected	31581
Independent reflections	8533 [R$_{\text{int}}$ = 0.0622, R$_{\text{sigma}}$ = 0.0724]
Data/restraints/parameters	8533/0/463
Goodness-of-fit on F^2	0.997
Final R indexes [I>=2σ (I)]	R$_1$ = 0.0349, wR$_2$ = 0.0767
Final R indexes [all data]	R$_1$ = 0.0502, wR$_2$ = 0.0819
Largest diff. peak/hole / e Å^{-3}	2.26/-2.13

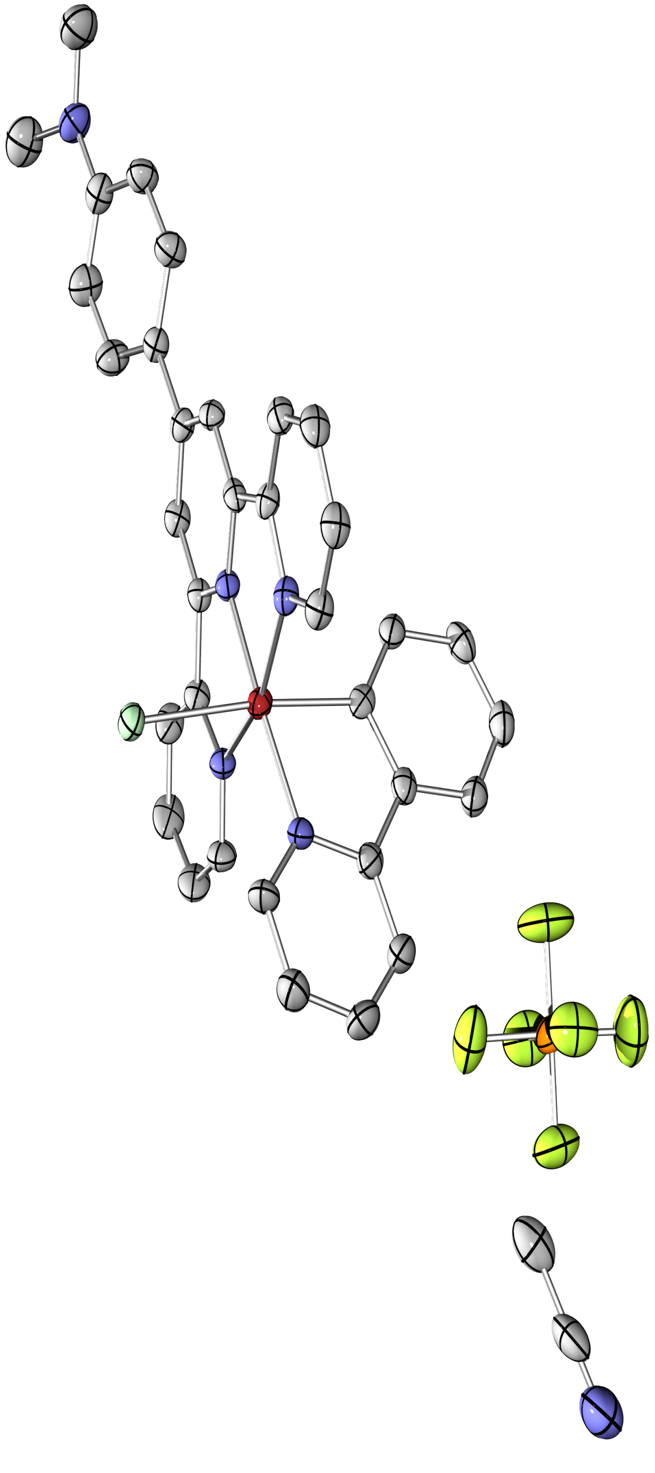

Figure S45: ORTEP projection of [Ir(**4-NMe$_2$PhTerpy**)(ppy)Cl](PF_6) rendered with POV-RAY at the at the 40% thermal ellipsoid level. Carbon atoms are grey, nitrogen atoms are light blue, iridium atoms are dark red, chlorine atoms are pale green, phosphorus atoms are orange.

6 Infrared Spectra

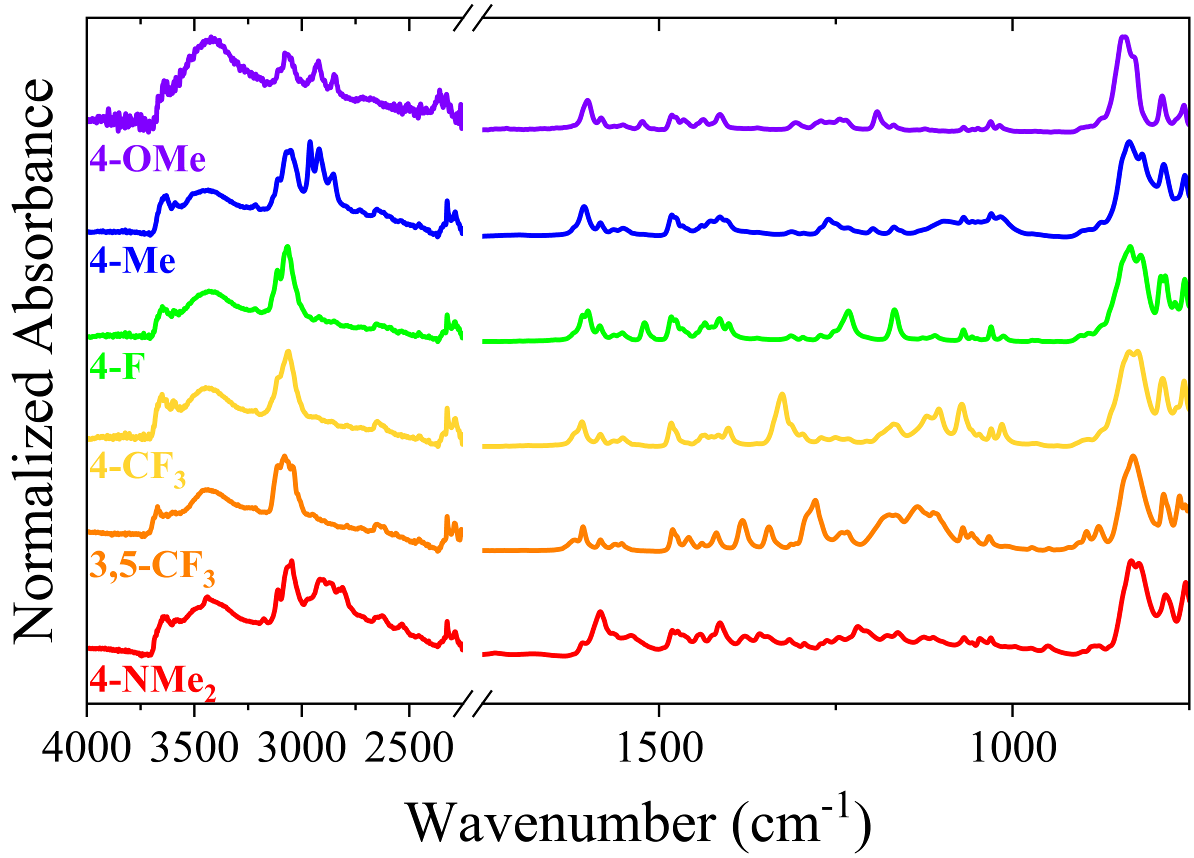

Figure S46: IR spectra of [Ir(**RPhTerpy**)(ppy)Cl](PF_6). A drop of [Ir(**4-OMePhTerpy**)(ppy)Cl](PF_6) dissolved in a minimal amount of acetonitrile was placed on the ATR crystal and allowed to evaporate. Spectra were collected for the dried solid (1000 scans). Spectra were processed in Origin 2025b. To better show features between 4000-2250 cm^{-1}, this portion of the spectra was baseline corrected and normalized separately from the 1750-750 cm^{-1} range.

7 Computational Details

A detailed description of the nomenclature and title cards are provided below in Section 7. UCSF ChimeraX was used to visualize molecular orbitals of computationally generated structures.[10]

7.1 Computational Nomenclature Key

The xyz coordinate files for each optimized and frequency checked structures are included in a supplemental zip file. The nomenclature key for the xyz coordinate files and Computational Data Tables is as follows, using singlet **[Ir(4-OMePhTerpy)(Ppy)(Cl)]**$^+$ as a representative example. Note: The triplet state is represented with "t" and the ppy isomer, in which the cyclometallated carbon in the phenyl-ring is *cis* to the chlorine, is represented with "iso."

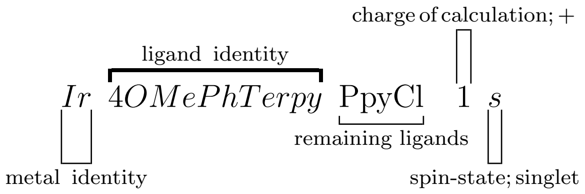

7.2 Example Input Title Card for Optimization and Frequency Check Calculation

An example input title card is below. Note: c = charge and m = multiplicy. Ir4OMePhTerpyPpyCl1s ([Ir(**4OMePhTerpy**)(ppy)Cl](PF_6)) is used as an example here.

```
%chk=Ir4OMePhTerpyPpyCl1s.chk
%nprocshared=16 %mem=32GB #p opt(calcfc,tight) int=ultrafine freq
b3pw91/gen maxdisk=8GB 6d empiricaldispersion=GD3 gfinput gfprint pseu-
do=read scrf=(cpcm,solvent=acetonitrile) pop=full


recl_pyo


c m
[insert atoms and coordinates here]


C H N F Cl 0
def2svp
****
[insert def2svp text and values here]
****


IR 0
[insert Ir-ECP text and values here]
```

7.3 Example Input Title Card for TD DFT Calculation

Here, Ir4OMePhTerpyCl1s is used as an example.

30 vertical excitations were used for singlet structures, while 90 for triplets.

```
%OldChk=Ir4OMePhTerpyPpyCl1s.chk %chk=Ir4OMePhTerpyPpyCl1sTD.chk
%nprocshared=16 %mem=32GB #p int=ultrafine TDA(singlets, NStates=30)
geom=AllCheck guess=read b3pw91/gen maxdisk=8GB 6d empiricaldisper-
sion=GD3 scrf=(cpcm,solvent=acetonitrile) gfinput gfprint pseudo=read
c m
[insert atoms and coordinates here]


C H N F Cl 0
def2svp
****
[insert def2svp text and values here]
****


IR 0
[insert Ir-ECP text and values here]
```

7.4 def2-svp text and values

```
Ir 0
S 3 1.00
    30.000000000 0.30797903228
    27.000000000 -0.46726361781
    13.961973911 0.47161003146
S 1 1.00
    5.3956977802 1.0000000
S 1 1.00
    1.2149128721 1.0000000
S 1 1.00
    0.55885743756 1.0000000
S 1 1.00
    0.14097974313 1.0000000
S 1 1.00
    0.50021924989D-01 1.0000000
P 4 1.00
    15.902664143 -0.16290720099
    14.415830698 0.23483212987
    5.7597608991 -0.30305337176
    1.5008913108 0.55512982069
P 1 1.00
    0.72348035957 1.0000000
P 1 1.00
    0.32785980328 1.0000000
P 1 1.00
    0.56000000000D-01 1.0000000
D 4 1.00
    8.6321692504 0.75000099949D-01
    6.5898192302 -0.17326965173
    1.5808379663 0.55065196913
    0.71827834905 0.85273641436
D 1 1.00
    0.30571851998 1.0000000
D 1 1.00
    0.11827071879 1.0000000
F 1 1.00
    0.6100800 1.0000000
```

7.5 IR-ECP text and values

```
IR-ECP 3 60
f potential
    1
    2 3.03407192 21.53103107
s-f potential
    3
    2 13.65220260 732.26919978
    2 6.82610130 26.48472087
    2 3.03407192 -21.53103107
p-f potential
    3
    2 10.27986840 299.48947357
    2 5.13993410 26.46623354
    2 3.03407192 -21.53103107
d-f potential
    3
    2 7.34985897 124.45759451
    2 3.67492949 14.03599518
     2 3.03407192 -21.53103107
```

7.6 Computational Data Table

Table S16: Table of Gibbs Free Energy values (in Hartrees) for the optimized structures and their corresponding imaginary frequencies. [a] - isomer of [Ir(**4-OMePhTerpy**)(ppy)Cl](PF_6) where the cyclometallated carbon is *cis* to the chlorine and pyridine nitrogen *trans* to it; it is 8.5 kcal less stable in the singlet spin state. s = singlet and t = triplet.

Compound	Spin State	Gibbs Free Energy	Number of Imaginary Frequencies
[Ir(**3,5-CF_3PhTerpy**)(ppy)Cl](PF_6)	s	-2688.330618	0
[Ir(**4-CF_3PhTerpy**)(ppy)Cl](PF_6)	s	-2351.651588	0
[Ir(**4-FPhTerpy**)(ppy)Cl](PF_6)	s	-2114.106122	0
[Ir(**4-MePhTerpy**)(ppy)Cl](PF_6)	s	-2054.229535	0
[Ir(**4-OMePhTerpy**)(ppy)Cl](PF_6)	s	-2129.349566	0
[Ir(**4-NMe_2PhTerpy**)(ppy)Cl](PF_6)	s	-2148.751760	0
Iso-[Ir(**4-OMePhTerpy**)(ppy)Cl](PF_6)[a]	s	-2129.336868	0
[Ir(**3,5-CF_3PhTerpy**)(ppy)Cl](PF_6)	t	-2688.259133	0
[Ir(**4-CF_3PhTerpy**)(ppy)Cl](PF_6)	t	-2351.580208	0
[Ir(**4-FPhTerpy**)(ppy)Cl](PF_6)	t	-2114.033930	0
[Ir(**4-MePhTerpy**)(ppy)Cl](PF_6)	t	-2054.156597	0
[Ir(**4-OMePhTerpy**)(ppy)Cl](PF_6)	t	-2129.275215	0
[Ir(**4-NMe_2PhTerpy**)(ppy)Cl](PF_6)	t	-2148.689057	0
Iso-[Ir(**4-OMePhTerpy**)(ppy)Cl](PF_6)[a]	t	-2129.261183	0

7.7 Computational Structure Metric Comparison to Crystallographic Data

Table S17: Comparison of select bond lengths (Å) Comparison of select bond lengths (Å) and angles (°) of the terpyridine moiety in [Ir(**RPhTerpy**)(ppy)Cl](PF_6) complexes for single crystal X-ray diffraction structures and computational derived structures in the singlet and triplet state. [a] - crystallographic data not acquired

	**Distal Ir—N (Avg)**			**Central Ir—N**			**RPh Torsion Angle**		
**Compound**	XRD	Singlet	Triplet	XRD	Singlet	Triplet	XRD	Singlet	Triplet
[Ir(**3,5-CF$_3$PhTerpy**)(ppy)Cl](PF_6)	2.052(6)	2.040	2.045	1.963(4)	1.953	1.950	-45.3	-38.1	-30.0
[Ir(**4-CF$_3$PhTerpy**)(ppy)Cl](PF_6)	2.047(4)	2.040	2.045	1.947(3)	1.953	1.951	6.4	-37.3	-31.7
[Ir(**4-FPhTerpy**)(ppy)Cl](PF_6)	[a]	2.039	2.043	1.963(4)	1.947	1.954	[a]	-35.5	-33.6
[Ir(**4-MePhTerpy**)(ppy)Cl](PF_6)	2.054(3)	2.041	2.043	1.960(2)	1.949	1.954	-7.0	-34.3	-32.7
[Ir(**4-OMePhTerpy**)(ppy)Cl](PF_6)	2.054(9)	2.041	2.042	1.972(4)	1.949	1.955	-9.9	-31.9	-32.1
[Ir(**4-NMe$_2$PhTerpy**)(ppy)Cl](PF_6)	2.062(4)	2.041	2.042	1.961(4)	1.950	1.930	14.4	-26.2	-12.1

Table S 18: Comparison of select bond lengths (Å) of the ppy ligand in [Ir(**RPhTerpy**)(ppy)Cl](PF_6) complexes for single crystal X-ray diffraction structures and computational derived structures in the singlet and triplet state.

	**Ir-N Bond Lengths**			**Ir-C Bond Lengths**		
**Compound**	XRD	Singlet	Triplet	XRD	Singlet	Triplet
[Ir(**3,5-CF$_3$PhTerpy**)(ppy)Cl](PF_6)	2.073(4)	2.074	2.085	2.010(5)	2.011	1.970
[Ir(**4-CF$_3$PhTerpy**)(ppy)Cl](PF_6)	2.076(3)	2.074	2.086	2.023(4)	2.010	1.971
[Ir(**4-FPhTerpy**)(ppy)Cl](PF_6)	[a]	2.074	2.086	[a]	2.010	1.971
[Ir(**4-MePhTerpy**)(ppy)Cl](PF_6)	2.088(3)	2.074	2.086	2.029(3)	2.010	1.971
[Ir(**4-OMePhTerpy**)(ppy)Cl](PF_6)	2.081(4)	2.074	2.086	2.016(5)	2.010	1.973
[Ir(**4-NMe$_2$PhTerpy**)(ppy)Cl](PF_6)	2.083(4)	2.075	2.088	2.016(4)	2.010	2.003

7.8 MO Composition

7.8.1 RPhTerpy Energy-Composition Diagram

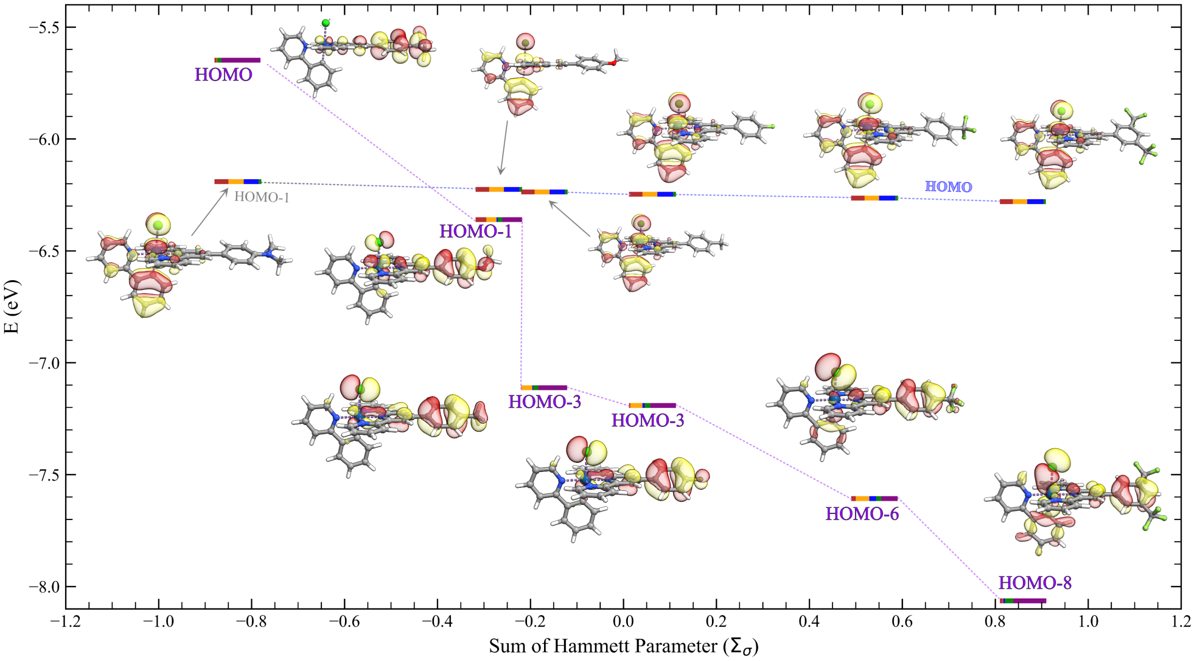

Figure S47: Energy diagram showing the selected Kohn–Sham MO surfaces obtained by DFT calculations for the [Ir(**RPhTerpy**)(ppy)Cl](PF_6) series plotted as a function of $_\sigma$. Only the occupied orbitals with compositions dominated by ppy and RPh- are plotted for clairty. The horizonal bars are orbital composition bars indicating contributions for the five defined fragments of the complexes: Ir = red, Cl = orange, ppy moiety= blue, terpy moiety = green, RPh- moiety = purple. The blue dashed lines connects the ppy dominated HOMOs, and the purple dashed lines connects RPh- dominated occipied orbitals.

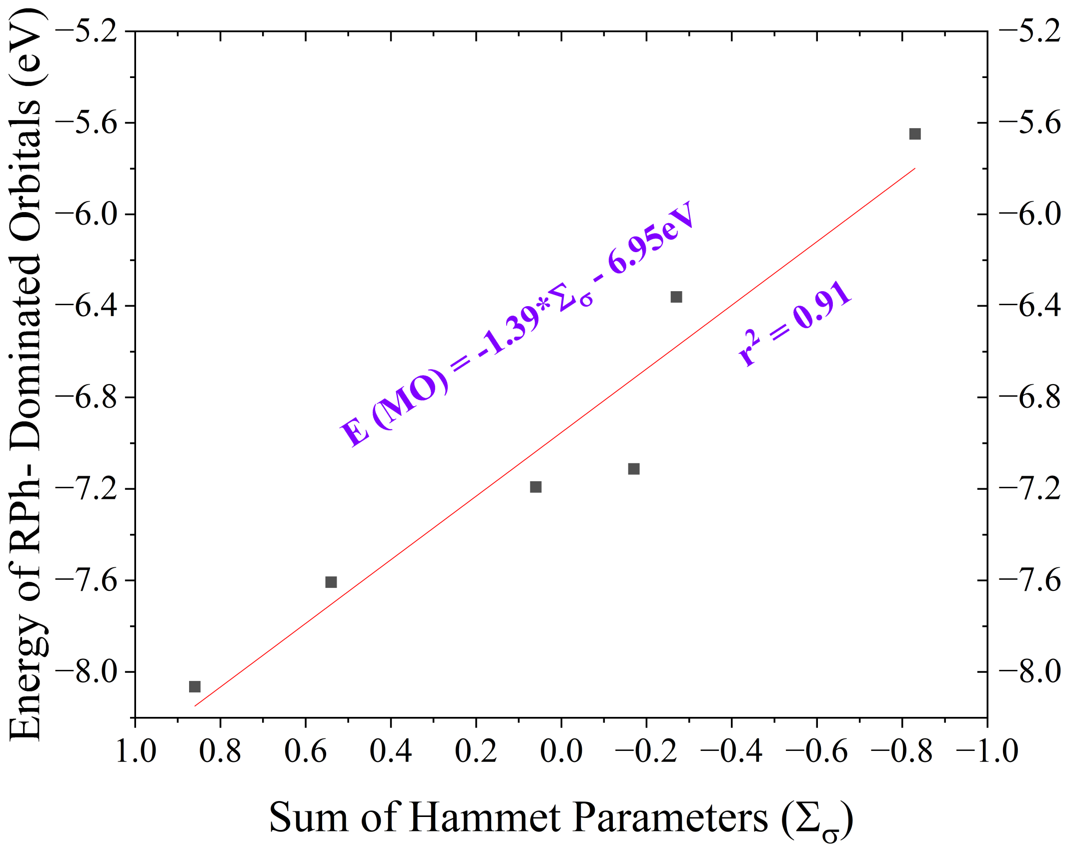

Figure S48: Plot Kohn–Sham RPh-dominated highest occupied MO energies obtained from DFT calculations for the [Ir(**RPhTerpy**)(ppy)Cl](PF_6) series, plotted as a function of $_\sigma$.

Table S19: Table of RPh-Dominated MO's and their energies used in Figure S48

Complex	Hammett Parameter Sum	MO	MO Energy (eV)
[Ir(**3,5-CF$_3$PhTerpy**)(ppy)Cl](PF_6)	0.86	HOMO-8	-8.06426
[Ir(**4-CF$_3$PhTerpy**)(ppy)Cl](PF_6)	0.54	HOMO-6	-7.60727
[Ir(**4-FPhTerpy**)(ppy)Cl](PF_6)	0.06	HOMO-3	-7.19132
[Ir(**4-MePhTerpy**)(ppy)Cl](PF_6)	-0.17	HOMO-3	-7.11192
[Ir(**4-OMePhTerpy**)(ppy)Cl](PF_6)	-0.27	HOMO-1	-6.36056
[Ir(**4-NMe$_2$PhTerpy**)(ppy)Cl](PF_6)	-0.83	HOMO	-5.64819

7.8.2 Percent Composition Tables for Select MOs

Table S20: Percent Composition Table for select orbitals of [Ir(**3,5-CF$_3$PhTerpy**)(ppy)Cl](PF$_6$), and their associated energies.

Moiety	HOMO-8	HOMO-7	HOMO-6	HOMO-5	HOMO-4	HOMO-3	HOMO-2	HOMO-1	HOMO	LUMO	LUMO+1
Ir	6	14	21	59	7	15	2	31	28	7	2
Cl	1	3	29	8	52	27	18	57	31	2	0
ppy	4	16	12	24	25	54	80	5	35	0	0
terpy	18	67	14	9	10	4	0	6	5	84	97
RPh	71	0	24	0	6	0	0	1	0	6	0
E (eV)	-8.0645	-7.8511	-7.6353	-7.5815	-7.5570	-7.2357	-6.9737	-6.7070	-6.2802	-3.2063	-2.9317

Table S21: Percent Composition Table for select orbitals of [Ir(**4-CF$_3$PhTerpy**)(ppy)Cl](PF$_6$), and their associated energies.

Moiety	HOMO-6	HOMO-5	HOMO-4	HOMO-3	HOMO-2	HOMO-1	HOMO	LUMO	LUMO+1
Ir	10	59	6	15	2	32	29	6	2
Cl	29	8	47	26	18	55	31	2	0
ppy	15	24	19	54	79	5	35	0	0
terpy	11	9	12	5	0	7	6	85	97
RPh	35	0	15	0	0	2	0	6	0
E (eV)	-7.6073	-7.5462	-7.5073	-7.2136	-6.9670	-6.6771	-6.2644	-3.1641	-2.8883

Table S22: Percent Composition Table for select orbitals of [Ir(**4-FPhTerpy**)(ppy)Cl](PF$_6$), and their associated energies.

Moiety	HOMO-4	HOMO-3	HOMO-2	HOMO-1	HOMO	LUMO	LUMO+1
Ir	16	3	2	32	29	6	2
Cl	25	26	19	51	32	2	0
ppy	54	5	79	5	34	0	0
terpy	5	12	0	7	6	87	97
RPh	0	55	0	5	0	5	0
E (eV)	-7.2296	-7.1913	-6.9573	-6.6071	-6.2472	-3.0975	-2.8529

Table S23: Percent Composition Table for select orbitals of [Ir(**4-MePhTerpy**)(ppy)Cl](PF_6), and their associated energies.

Moiety	HOMO-4	HOMO-3	HOMO-2	HOMO-1	HOMO	LUMO	LUMO+1
Ir	17	1	2	32	29	6	2
Cl	24	23	20	48	32	2	0
ppy	54	2	78	4	33	0	0
terpy	5	11	0	7	6	87	97
RPh	0	62	0	8	0	5	0
E (eV)	-7.1787	-7.1119	-6.9516	-6.5624	-6.2370	-3.0740	-2.8262

Table S24: Percent Composition Table for select orbitals of [Ir(**4-OMePhTerpy**)(ppy)Cl](PF_6), and their associated energies.

Moiety	HOMO-4	HOMO-3	HOMO-2	HOMO-1	HOMO	LUMO	LUMO+1
Ir	18	2	9	23	29	6	2
Cl	23	20	40	22	32	2	0
ppy	54	78	2	3	33	0	0
terpy	5	0	7	9	6	87	97
RPh	0	0	42	43	0	5	0
E (eV)	-7.1631	-6.9450	-6.8376	-6.3606	-6.2254	-3.0434	-2.8058

Table S25: Percent Composition Table for select orbitals of [Ir(**4-NMe$_2$PhTerpy**)(ppy)Cl](PF_6), and their associated energies.

Moiety	HOMO-4	HOMO-3	HOMO-2	HOMO-1	HOMO	LUMO	LUMO+1
Ir	20	2	28	30	5	5	2
Cl	20	22	57	33	1	2	0
ppy	54	76	4	32	0	0	0
terpy	6	0	6	6	9	86	97
RPh	0	0	6	0	84	6	0
E (eV)	-7.1174	-6.9251	-6.6522	-6.1909	-5.6482	-2.9673	-2.7360

7.9 TD DFT Predicted and Experimental UV-Vis Spectra Overlays

[Page intentionally left black due to formatting requirements.]

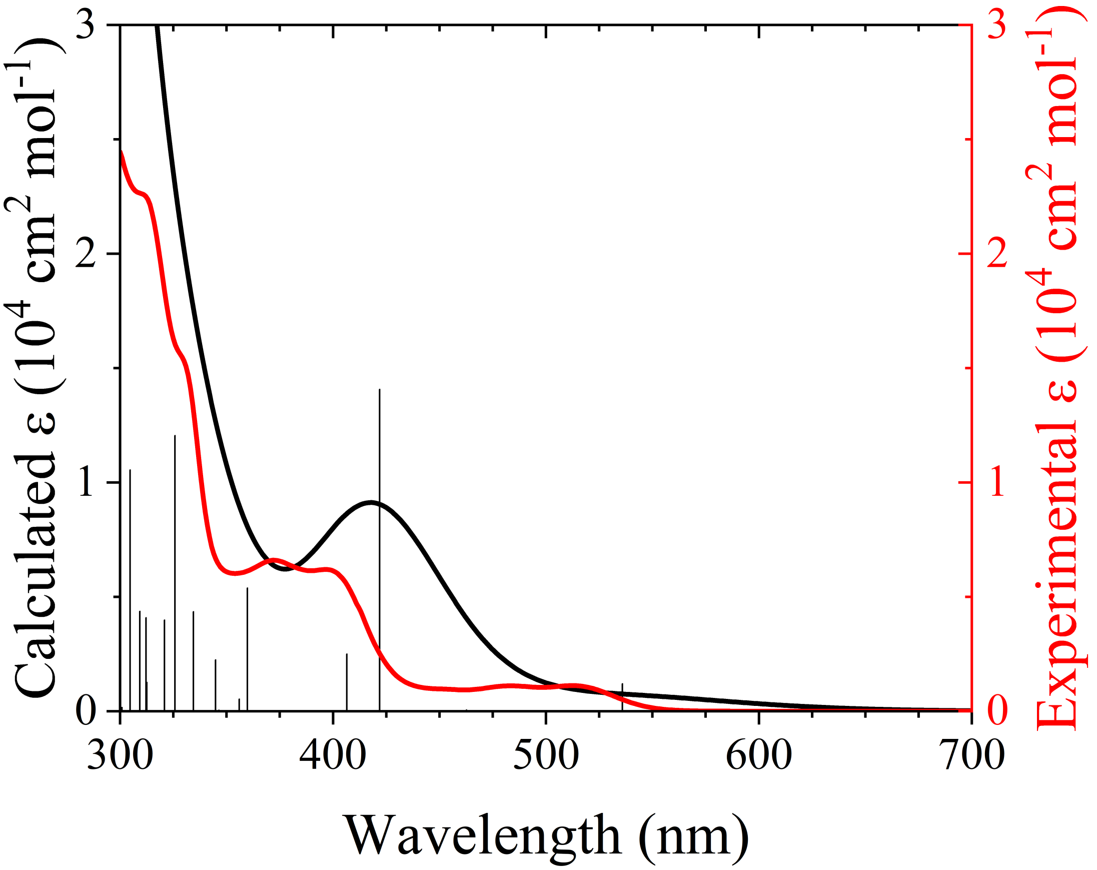

Figure S49: Overlay of the UV-Vis Spectrum (red) and TD DFT predicted UV-Vis spectra (black) of the S_0 ground state for **[Ir(3,5-CF$_3$PhTerpy)(ppy)Cl][PF$_6$]**. The black lines represent the transition wavelengths, with relative oscillator strength indicated by line height. The calculated UV-Vis spectrum was generated with GaussView 5.0.9 using a UV-Vis Peak Half-Width at Half Height setting of 2000 cm^{-1}.

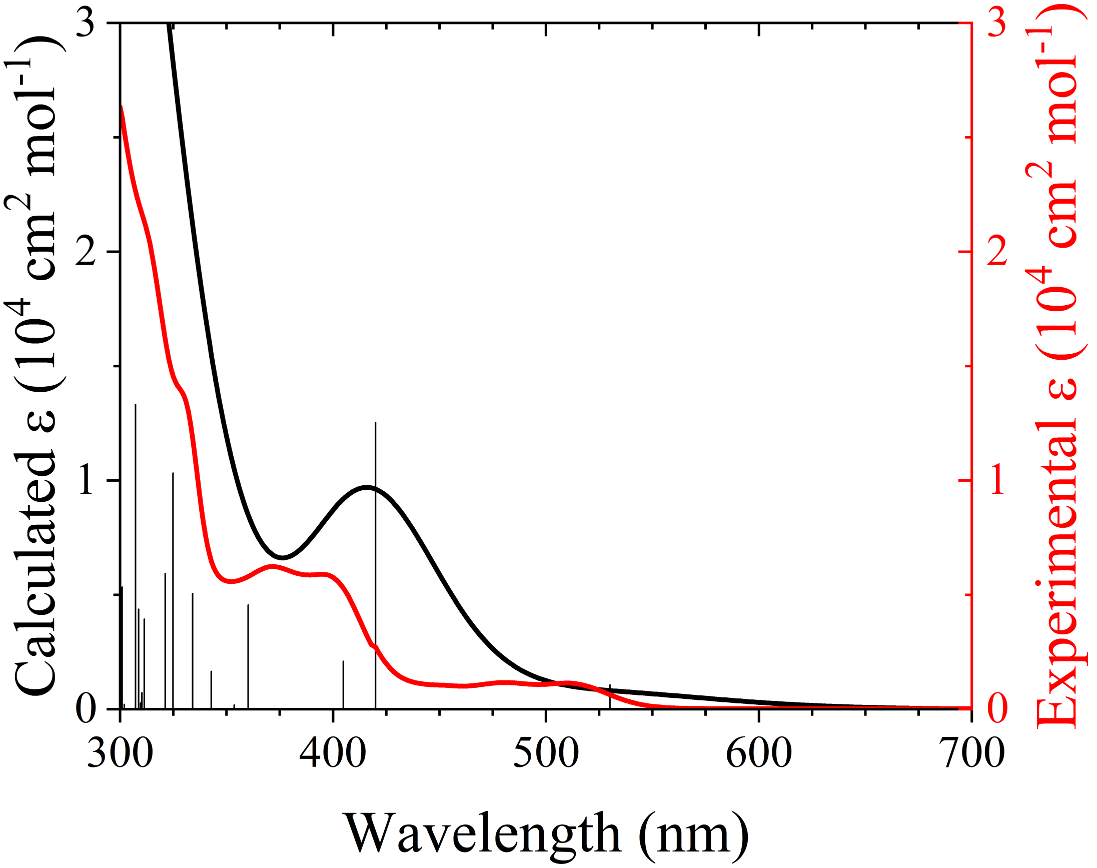

Figure S50: Overlay of the UV-Vis Spectrum (red) and TD DFT predicted UV-Vis spectra (black) of the S_0 ground state for **[Ir(4-CF$_3$PhTerpy)(ppy)Cl][PF$_6$]**. The black lines represent the transition wavelengths, with relative oscillator strength indicated by line height. The calculated UV-Vis spectrum was generated with GaussView 5.0.9 using a UV-Vis Peak Half-Width at Half Height setting of 2000 cm^{-1}.

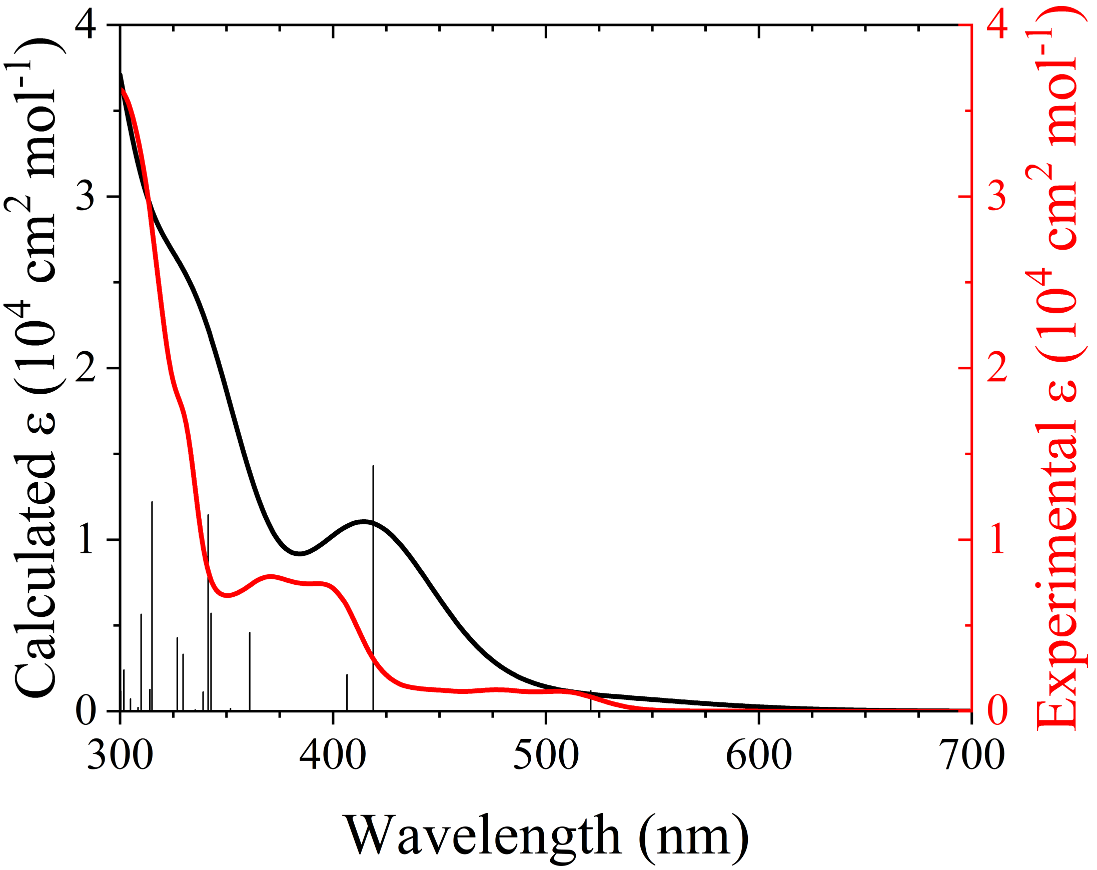

Figure S51: Overlay of the UV-Vis Spectrum (red) and TD DFT predicted UV-Vis spectra (black) of the S_0 ground state for **[Ir(4-FPhTerpy)(ppy)Cl][PF$_6$]**. The black lines represent the transition wavelengths, with relative oscillator strength indicated by line height. The calculated UV-Vis spectrum was generated with GaussView 5.0.9 using a UV-Vis Peak Half-Width at Half Height setting of 2000 cm^{-1}.

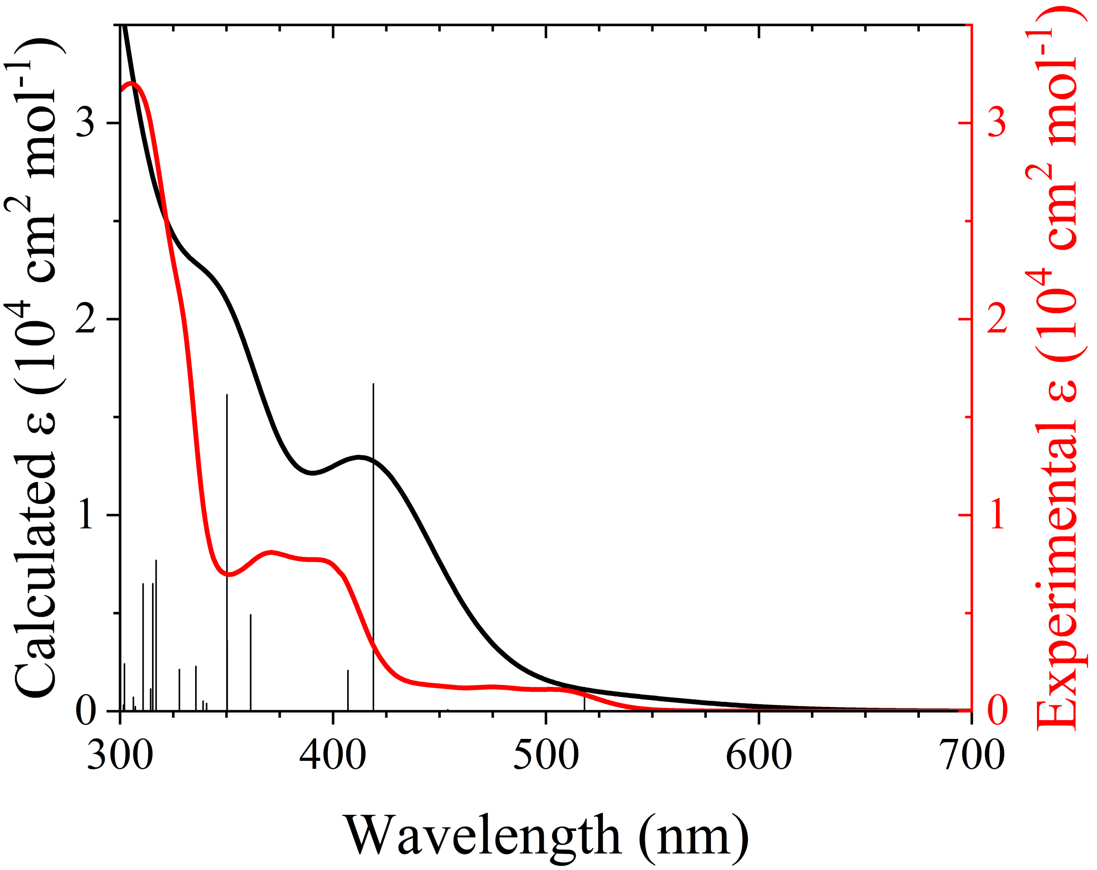

Figure S52: Overlay of the UV-Vis Spectrum (red) and TD DFT predicted UV-Vis spectra (black) of the S_0 ground state for **[Ir(4-MePhTerpy)(ppy)Cl][PF$_6$]**. The black lines represent the transition wavelengths, with relative oscillator strength indicated by line height. The calculated UV-Vis spectrum was generated with GaussView 5.0.9 using a UV-Vis Peak Half-Width at Half Height setting of 2000 cm^{-1}.

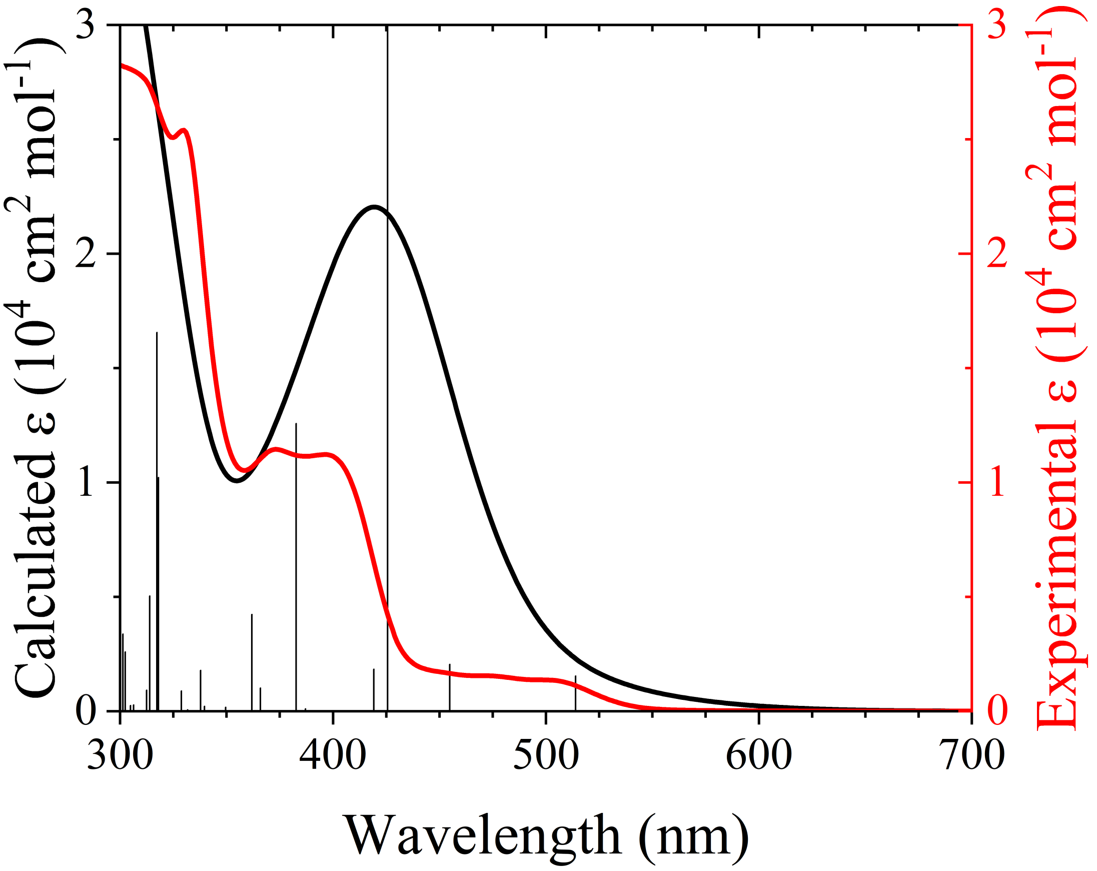

Figure S53: Overlay of the UV-Vis Spectrum (red) and TD DFT predicted UV-Vis spectra (black) of the S_0 ground state for **[Ir(4-OMePhTerpy)(ppy)Cl][PF$_6$]**. The black lines represent the transition wavelengths, with relative oscillator strength indicated by line height. The calculated UV-Vis spectrum was generated with GaussView 5.0.9 using a UV-Vis Peak Half-Width at Half Height setting of 2000 cm^{-1}.

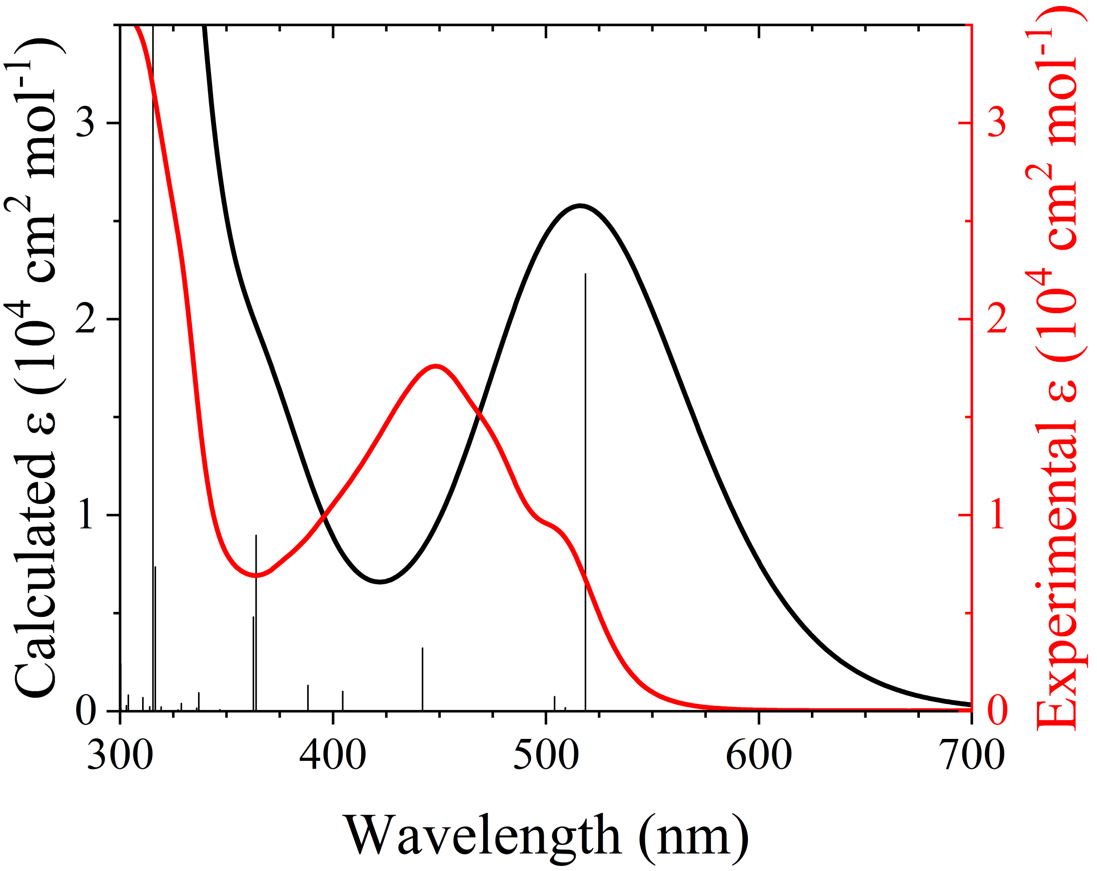

Figure S54: Overlay of the UV-Vis Spectrum (red) and TD DFT predicted UV-Vis spectra (black) of the S_0 ground state for **[Ir(4-NMe$_2$PhTerpy)(ppy)Cl][PF$_6$]**. The black lines represent the transition wavelengths, with relative oscillator strength indicated by line height. The calculated UV-Vis spectrum was generated with GaussView 5.0.9 using a UV-Vis Peak Half-Width at Half Height setting of 2000 cm^{-1}.

7.10 XYZ coordinates of Optimized Structures

Ir4CF3PhTerpyPpyCl1s

Ir -1.837663000 0.020472000 -0.400831000

C -2.193364000 -0.073901000 1.575424000

N -3.911921000 0.027482000 -0.419037000

Cl -1.592603000 0.138696000 -2.855023000

N -1.504862000 2.029276000 -0.269420000

N 0.106177000 0.008089000 -0.270273000

N -1.516664000 -1.993071000 -0.471482000

C -1.238984000 -0.125281000 2.600156000

C -1.632877000 -0.186243000 3.939931000

C -2.990691000 -0.196773000 4.281271000

C -3.955944000 -0.146263000 3.278234000

C -3.565891000 -0.085460000 1.930540000

C -4.504761000 -0.029922000 0.808769000

C -5.901461000 -0.032360000 0.909178000

C -6.679342000 0.023989000 -0.241576000

C -6.050022000 0.082244000 -1.487781000

C -4.662838000 0.081827000 -1.533855000

C -2.421676000 3.004111000 -0.251219000

C -2.068012000 4.346695000 -0.147015000

C -0.719221000 4.684607000 -0.056860000

C 0.236105000 3.668804000 -0.071229000

C -0.175663000 2.342622000 -0.175607000

C 0.745660000 1.190109000 -0.189656000

C 2.135556000 1.207013000 -0.118436000

C 2.846550000 -0.006299000 -0.136514000

C 2.128273000 -1.211874000 -0.230370000

C 0.738448000 -1.180286000 -0.299791000

C -0.189616000 -2.322676000 -0.406579000

C 0.213733000 -3.655226000 -0.438226000

C -0.747680000 -4.660874000 -0.533558000

C -2.094021000 -4.306945000 -0.593389000

C -2.439373000 -2.958630000 -0.558148000

C 4.325007000 -0.013611000 -0.060488000

C 4.998611000 -1.012355000 0.662845000

C 5.079075000 0.976349000 -0.713048000

C 6.388769000 -1.019080000 0.737536000

C 6.469641000 0.968576000 -0.645362000

C 7.125042000 -0.028204000 0.082448000

H -0.171833000 -0.117390000 2.360671000

H -6.620599000 0.127556000 -2.416857000

H 2.656629000 -2.164058000 -0.275291000

H -6.372694000 -0.079074000 1.891279000

H 2.667261000 2.153859000 -0.026156000

H -0.873579000 -0.225679000 4.726717000

H 4.433787000 -1.780297000 1.196316000

H 4.579905000 1.745929000 -1.306419000

H 7.046321000 1.733048000 -1.169915000

H 6.901366000 -1.796855000 1.307308000

H 1.273421000 -3.906599000 -0.387154000

H 1.297554000 3.907122000 0.001268000

H -3.479054000 -2.630631000 -0.601488000

H -0.444172000 -5.709565000 -0.558828000

H -2.880105000 -5.060326000 -0.665755000

H -3.463615000 2.688726000 -0.323743000

H -0.409166000 5.728407000 0.025967000

H -2.849699000 5.108027000 -0.136818000

H -3.295071000 -0.244152000 5.329907000

H -5.013895000 -0.154431000 3.552242000

H -4.104504000 0.125144000 -2.471980000

H -7.769568000 0.022299000 -0.168052000

C 8.626570000 -0.005288000 0.204612000

F 9.208862000 0.549118000 -0.869060000

F 9.021692000 0.708131000 1.275219000

F 9.136439000 -1.237693000 0.348758000

Ir4CF3PhTerpyPpyCl1t

Ir -1.850384000 0.018903000 -0.400502000

C -2.156527000 -0.066696000 1.544287000

N -3.936065000 0.022804000 -0.403842000

Cl -1.640087000 0.128218000 -2.820326000

N -1.513300000 2.032292000 -0.286498000

N 0.100225000 0.010074000 -0.297615000

N -1.522650000 -1.997167000 -0.471914000

C -1.162279000 -0.112159000 2.544694000

C -1.520213000 -0.168107000 3.885575000

C -2.876767000 -0.179541000 4.255873000

C -3.880091000 -0.135564000 3.286259000

C -3.534108000 -0.079516000 1.932962000

C -4.501024000 -0.030677000 0.836320000

C -5.892322000 -0.036119000 0.976782000

C -6.697739000 0.013140000 -0.156914000

C -6.098069000 0.067270000 -1.416100000

C -4.709999000 0.070392000 -1.498780000

C -2.439288000 3.011222000 -0.262264000

C -2.094956000 4.345537000 -0.163975000

C -0.731579000 4.689065000 -0.080333000

C 0.225249000 3.687841000 -0.097780000

C -0.166198000 2.344308000 -0.200354000

C 0.742771000 1.211976000 -0.219241000

C 2.128437000 1.217228000 -0.148458000

C 2.847364000 -0.002470000 -0.156820000

C 2.122286000 -1.215198000 -0.241449000

C 0.736687000 -1.197729000 -0.317656000

C -0.176845000 -2.322268000 -0.410946000

C 0.207788000 -3.671719000 -0.432247000

C -0.753605000 -4.665244000 -0.513185000

C -2.115271000 -4.308793000 -0.569554000

C -2.453253000 -2.969602000 -0.542888000

C 4.320547000 -0.008980000 -0.077508000

C 5.008301000 -1.063144000 0.556005000

C 5.082373000 1.036666000 -0.635288000

C 6.397353000 -1.072037000 0.631214000

C 6.471926000 1.032068000 -0.561118000

C 7.135076000 -0.022967000 0.072999000

H -0.106771000 -0.102993000 2.264525000

H -6.691214000 0.106928000 -2.331098000

H 2.651993000 -2.167455000 -0.277263000

H -6.339724000 -0.079519000 1.969875000

H 2.660089000 2.164598000 -0.056359000

H -0.747314000 -0.203081000 4.657719000

H 4.451170000 -1.878555000 1.022887000

H 4.587117000 1.853747000 -1.164826000

H 7.044505000 1.844922000 -1.012848000

H 6.910604000 -1.896958000 1.130364000

H 1.266655000 -3.928596000 -0.383539000

H 1.285588000 3.934049000 -0.030083000

H -3.492083000 -2.637867000 -0.583187000

H -0.455568000 -5.715843000 -0.530930000

H -2.900978000 -5.062805000 -0.633520000

H -3.479919000 2.689674000 -0.329157000

H -0.428461000 5.735291000 -0.000183000

H -2.877356000 5.105613000 -0.153336000

H -3.150952000 -0.223119000 5.312799000

H -4.925981000 -0.145334000 3.599553000

H -4.179684000 0.111287000 -2.452574000

H -7.785616000 0.009078000 -0.058008000

C 8.632726000 -0.006695000 0.204181000

F 9.219683000 0.682525000 -0.787009000

F 9.025807000 0.565890000 1.359253000

F 9.151733000 -1.245439000 0.194060000

Ir4FPhTerpyPpyCl1s

Ir -1.188463000 0.020189000 -0.404656000

C -1.575981000 -0.076347000 1.565047000

N -3.262057000 0.024161000 -0.457065000

Cl -0.902572000 0.140107000 -2.855158000

N -0.860267000 2.029598000 -0.267354000

N 0.753486000 0.011254000 -0.240800000

N -0.861432000 -1.992764000 -0.470303000

C -0.638711000 -0.128564000 2.605563000

C -1.054613000 -0.191703000 3.938549000

C -2.417883000 -0.203742000 4.257539000

C -3.366464000 -0.152727000 3.238748000

C -2.954249000 -0.089735000 1.897758000

C -3.874795000 -0.034221000 0.760861000

C -5.272980000 -0.037752000 0.838501000

C -6.032046000 0.018382000 -0.324761000

C -5.382545000 0.077474000 -1.560543000

C -3.994781000 0.078192000 -1.583905000

C -1.778623000 3.003260000 -0.266389000

C -1.428973000 4.346406000 -0.156887000

C -0.082440000 4.686225000 -0.042725000

C 0.874333000 3.671734000 -0.039068000

C 0.466694000 2.344757000 -0.149853000

C 1.390262000 1.193573000 -0.147252000

C 2.777981000 1.212815000 -0.047621000

C 3.495105000 0.001473000 -0.054532000

C 2.777160000 -1.204436000 -0.165199000

C 1.389200000 -1.175329000 -0.258785000

C 0.465058000 -2.319584000 -0.382530000

C 0.871227000 -3.651478000 -0.407440000

C -0.086364000 -4.659035000 -0.519240000

C -1.432255000 -4.307853000 -0.601880000

C -1.780615000 -2.960251000 -0.572558000

C 4.969618000 -0.004464000 0.050366000

C 5.634998000 -1.025132000 0.753424000

C 5.737123000 1.009841000 -0.550531000

C 7.023382000 -1.035829000 0.859761000

C 7.126559000 1.007914000 -0.458228000

C 7.749988000 -0.016904000 0.249079000

H 0.432252000 -0.119847000 2.383675000

H -5.937897000 0.122606000 -2.498818000

H 3.306303000 -2.156343000 -0.204429000

H -5.760117000 -0.085026000 1.812801000

H 3.303866000 2.161284000 0.059529000

H -0.308366000 -0.231766000 4.737716000

H 5.063687000 -1.811012000 1.253112000

H 5.249991000 1.800435000 -1.126293000

H 7.732006000 1.783103000 -0.932160000

H 7.548289000 -1.816234000 1.414329000

H 1.930390000 -3.900701000 -0.337979000

H 1.934041000 3.911453000 0.051958000

H -2.820034000 -2.634220000 -0.633565000

H 0.219632000 -5.707131000 -0.539376000

H -2.215526000 -5.062769000 -0.687548000

H -2.818647000 2.686247000 -0.357296000

H 0.224657000 5.730538000 0.044726000

H -2.211789000 5.106634000 -0.161411000

H -2.739453000 -0.252854000 5.300961000

H -4.428828000 -0.162392000 3.495142000

H -3.421037000 0.122308000 -2.512667000

H -7.123330000 0.015887000 -0.268987000

F 9.082327000 -0.022671000 0.344419000

Ir4FPhTerpyPpyCl1t

Ir -1.199185000 0.020980000 -0.405672000

C -1.537248000 -0.076684000 1.533927000

N -3.284415000 0.025341000 -0.442797000

Cl -0.949512000 0.120113000 -2.823807000

N -0.870626000 2.029850000 -0.282981000

N 0.749944000 0.011508000 -0.270543000

N -0.866230000 -1.996848000 -0.473715000

C -0.559786000 -0.131556000 2.549791000

C -0.939732000 -0.190195000 3.884748000

C -2.301935000 -0.197002000 4.233030000

C -3.288909000 -0.145834000 3.247209000

C -2.920548000 -0.087075000 1.899808000

C -3.869470000 -0.032418000 0.787745000

C -5.262911000 -0.036970000 0.905493000

C -6.049624000 0.018014000 -0.240980000

C -5.429494000 0.076404000 -1.490072000

C -4.040349000 0.078089000 -1.550038000

C -1.797993000 3.009674000 -0.271623000

C -1.458279000 4.342608000 -0.161161000

C -0.094105000 4.688698000 -0.049245000

C 0.863505000 3.690501000 -0.052126000

C 0.478523000 2.343885000 -0.168256000

C 1.386422000 1.216952000 -0.173180000

C 2.774035000 1.219698000 -0.071184000

C 3.493119000 0.005342000 -0.073183000

C 2.771571000 -1.210236000 -0.178813000

C 1.388642000 -1.192475000 -0.280083000

C 0.477391000 -2.320493000 -0.390774000

C 0.865717000 -3.667790000 -0.407298000

C -0.092567000 -4.663851000 -0.505525000

C -1.452800000 -4.309365000 -0.583889000

C -1.793838000 -2.970452000 -0.561280000

C 4.966389000 -0.000759000 0.035341000

C 5.638907000 -1.037645000 0.710702000

C 5.740096000 1.029560000 -0.533450000

C 7.027937000 -1.050464000 0.818098000

C 7.129667000 1.030829000 -0.433626000

C 7.755718000 -0.012560000 0.242804000

H 0.500216000 -0.125881000 2.286880000

H -6.007635000 0.120499000 -2.414436000

H 3.304657000 -2.160835000 -0.207771000

H -5.726417000 -0.084180000 1.891051000

H 3.304170000 2.166429000 0.037996000

H -0.179466000 -0.230808000 4.669090000

H 5.070275000 -1.839147000 1.188409000

H 5.255128000 1.834746000 -1.090721000

H 7.733496000 1.822407000 -0.882444000

H 7.552075000 -1.847271000 1.350103000

H 1.924208000 -3.922294000 -0.341297000

H 1.921928000 3.938471000 0.037627000

H -2.832444000 -2.640517000 -0.618522000

H 0.207645000 -5.713852000 -0.519680000

H -2.236190000 -5.064536000 -0.661826000

H -2.836818000 2.687109000 -0.359828000

H 0.205141000 5.735207000 0.041283000

H -2.241723000 5.101644000 -0.162708000

H -2.593520000 -0.243149000 5.285185000

H -4.339957000 -0.152323000 3.542912000

H -3.494230000 0.121832000 -2.494756000

H -7.138992000 0.014967000 -0.159858000

F 9.090933000 -0.018156000 0.341948000

Ir4MePhTerpyPpyCl1s

Ir -1.200339000 0.019671000 -0.404655000

C -1.580539000 -0.073497000 1.566406000

N -3.274203000 0.024055000 -0.449532000

Cl -0.923111000 0.135577000 -2.856692000

N -0.870713000 2.029240000 -0.271701000

N 0.742310000 0.010426000 -0.246746000

N -0.873674000 -1.993531000 -0.468289000

C -0.639469000 -0.124086000 2.603645000

C -1.050423000 -0.184793000 3.938258000

C -2.412520000 -0.195984000 4.262348000

C -3.364843000 -0.146557000 3.246976000

C -2.957597000 -0.086017000 1.904338000

C -3.882388000 -0.032249000 0.770780000

C -5.280293000 -0.035471000 0.853685000

C -6.043736000 0.018875000 -0.306799000

C -5.398862000 0.075897000 -1.545107000

C -4.011175000 0.076390000 -1.573653000

C -1.788725000 3.003287000 -0.270052000

C -1.438384000 4.346469000 -0.163861000

C -0.091371000 4.685916000 -0.053868000

C 0.864993000 3.671070000 -0.050941000

C 0.456719000 2.343961000 -0.158259000

C 1.380087000 1.192448000 -0.156026000

C 2.767779000 1.211319000 -0.058953000

C 3.486001000 -0.000089000 -0.064768000

C 2.765887000 -1.205650000 -0.172694000

C 1.377979000 -1.176184000 -0.264008000

C 0.453031000 -2.320376000 -0.383714000

C 0.858703000 -3.652474000 -0.407885000

C -0.099354000 -4.659992000 -0.515656000

C -1.445450000 -4.308705000 -0.595092000

C -1.793356000 -2.961013000 -0.566669000

C 4.959633000 -0.006139000 0.039298000

C 5.631211000 -1.036406000 0.720918000

C 5.730102000 1.015509000 -0.542709000

C 7.020106000 -1.038215000 0.819543000

C 7.119657000 1.002371000 -0.447015000

C 7.793090000 -0.020342000 0.238756000

H 0.430639000 -0.115944000 2.377650000

H -5.957759000 0.119588000 -2.481349000

H 3.294490000 -2.157858000 -0.210912000

H -5.763785000 -0.081126000 1.829881000

H 3.294169000 2.159785000 0.045512000

H -0.301230000 -0.223594000 4.734738000

H 5.062916000 -1.834120000 1.206250000

H 5.244159000 1.815291000 -1.107673000

H 7.695450000 1.801283000 -0.923267000

H 7.516127000 -1.846682000 1.364564000

H 1.918046000 -3.901650000 -0.341016000

H 1.925074000 3.910390000 0.036774000

H -2.832860000 -2.634770000 -0.625234000

H 0.206388000 -5.708183000 -0.535182000

H -2.229103000 -5.063586000 -0.677548000

H -2.829079000 2.686417000 -0.357722000

H 0.216356000 5.730276000 0.030889000

H -2.220919000 5.106994000 -0.167680000

H -2.730229000 -0.243177000 5.307045000

H -4.426251000 -0.155516000 3.507354000

H -3.440861000 0.118929000 -2.504618000

H -7.134807000 0.016594000 -0.246923000

C 9.289994000 -0.012024000 0.374343000

H 9.588732000 0.488916000 1.311440000

H 9.698874000 -1.033070000 0.407515000

H 9.769451000 0.530489000 -0.453812000

Ir4MePhTerpyPpyCl1t

Ir -1.209652000 0.015741000 -0.405186000

C -1.548306000 -0.051383000 1.535982000

N -3.294919000 0.015223000 -0.442810000

Cl -0.959990000 0.149083000 -2.821892000

N -0.870497000 2.032902000 -0.285621000

N 0.739653000 0.010226000 -0.269477000

N -0.887842000 -1.993574000 -0.468890000

C -0.571114000 -0.079261000 2.552991000

C -0.951376000 -0.130675000 3.888276000

C -2.313558000 -0.151015000 4.235725000

C -3.300224000 -0.120849000 3.248775000

C -2.931475000 -0.070163000 1.901065000

C -3.880154000 -0.033450000 0.788019000

C -5.273607000 -0.045088000 0.905222000

C -6.060127000 -0.006623000 -0.242031000

C -5.439806000 0.043349000 -1.491421000

C -4.050709000 0.052686000 -1.550826000

C -1.795389000 3.012284000 -0.276215000

C -1.450035000 4.346928000 -0.174207000

C -0.089266000 4.688836000 -0.071098000

C 0.866466000 3.684609000 -0.072956000

C 0.472830000 2.343919000 -0.179419000

C 1.382787000 1.206886000 -0.181138000

C 2.764947000 1.214275000 -0.082532000

C 3.484200000 -0.009448000 -0.076925000

C 2.761215000 -1.215530000 -0.173371000

C 1.371579000 -1.202366000 -0.273625000

C 0.462653000 -2.320546000 -0.378763000

C 0.842597000 -3.674753000 -0.385046000

C -0.116961000 -4.665451000 -0.478902000

C -1.481485000 -4.306519000 -0.563893000

C -1.817439000 -2.968923000 -0.551303000

C 4.957367000 -0.015359000 0.028439000

C 5.637440000 -1.054085000 0.691684000

C 5.732223000 1.015782000 -0.534138000

C 7.027119000 -1.058042000 0.786050000

C 7.122395000 1.005147000 -0.437191000

C 7.800355000 -0.028871000 0.226113000

H 0.488937000 -0.063187000 2.290650000

H -6.017791000 0.074880000 -2.416398000

H 3.292724000 -2.167525000 -0.196109000

H -5.737225000 -0.084785000 1.891059000

H 3.297084000 2.160288000 0.018914000

H -0.191210000 -0.155139000 4.673399000

H 5.072481000 -1.861144000 1.165766000

H 5.246949000 1.825492000 -1.085689000

H 7.695573000 1.815630000 -0.897645000

H 7.523917000 -1.877148000 1.314984000

H 1.899967000 -3.933265000 -0.313475000

H 1.926295000 3.928201000 0.009751000

H -2.855158000 -2.636731000 -0.613358000

H 0.179167000 -5.716750000 -0.484648000

H -2.266466000 -5.060205000 -0.638975000

H -2.835407000 2.692180000 -0.358120000

H 0.214417000 5.734637000 0.011915000

H -2.231618000 5.108013000 -0.176011000

H -2.605396000 -0.189935000 5.288106000

H -4.351394000 -0.136757000 3.543730000

H -3.504361000 0.090895000 -2.495653000

H -7.149495000 -0.015706000 -0.161304000

C 9.298171000 -0.020977000 0.361086000

H 9.600792000 0.453458000 1.310829000

H 9.709720000 -1.041776000 0.363696000

H 9.775915000 0.544529000 -0.452891000

Ir4NMe2PhTerpyPpyCl1s

Ir -1.649349000 0.025468000 -0.402911000

C -2.004208000 -0.094874000 1.570117000

N -3.723873000 0.026565000 -0.422418000

Cl -1.400569000 0.175359000 -2.858336000

N -1.317282000 2.033499000 -0.245506000

N 0.295846000 0.016746000 -0.267834000

N -1.317877000 -1.986262000 -0.497695000

C -1.050204000 -0.157299000 2.595174000

C -1.444110000 -0.236279000 3.933888000

C -2.802079000 -0.254228000 4.275072000

C -3.767182000 -0.193385000 3.272460000

C -3.377065000 -0.114578000 1.925601000

C -4.316444000 -0.047932000 0.804602000

C -5.713277000 -0.056327000 0.905209000

C -6.491647000 0.011547000 -0.244618000

C -5.862654000 0.087263000 -1.490126000

C -4.475368000 0.092064000 -1.536105000

C -2.236258000 3.006587000 -0.217744000

C -1.886672000 4.348344000 -0.095532000

C -0.538539000 4.687557000 0.002741000

C 0.418641000 3.673962000 -0.021456000

C 0.011462000 2.347546000 -0.143321000

C 0.936699000 1.196705000 -0.168236000

C 2.323108000 1.216817000 -0.085041000

C 3.053570000 0.007088000 -0.115973000

C 2.322660000 -1.197015000 -0.234276000

C 0.935920000 -1.167259000 -0.307801000

C 0.010528000 -2.311587000 -0.432561000

C 0.416794000 -3.643125000 -0.478475000

C -0.540826000 -4.650697000 -0.587763000

C -1.888489000 -4.300754000 -0.646759000

C -2.237356000 -2.954171000 -0.597191000

C 4.517667000 0.000804000 -0.029757000

C 5.224359000 -1.110101000 0.474974000

C 5.288840000 1.104996000 -0.447166000

C 6.606447000 -1.123977000 0.566602000

C 6.672159000 1.106266000 -0.375684000

C 7.383486000 -0.012248000 0.139336000

H 0.016736000 -0.144021000 2.354940000

H -6.433636000 0.142032000 -2.418482000

H 2.844580000 -2.151047000 -0.297452000

H -6.184281000 -0.116864000 1.886706000

H 2.841374000 2.167978000 0.030654000

H -0.684932000 -0.284062000 4.720422000

H 4.680661000 -1.983745000 0.843739000

H 4.800373000 1.983217000 -0.877076000

H 7.206819000 1.985038000 -0.735454000

H 7.087068000 -2.007357000 0.986327000

H 1.477231000 -3.891394000 -0.426801000

H 1.479498000 3.913258000 0.056786000

H -3.277883000 -2.628445000 -0.639002000

H -0.233753000 -5.698091000 -0.624113000

H -2.672032000 -5.055751000 -0.729301000

H -3.277186000 2.689373000 -0.297403000

H -0.230763000 5.730913000 0.099353000

H -2.670039000 5.107830000 -0.078102000

H -3.106489000 -0.315593000 5.323013000

H -4.825191000 -0.207646000 3.546235000

H -3.916803000 0.148761000 -2.473506000

H -7.581870000 0.005201000 -0.170963000

N 8.745206000 -0.018416000 0.219571000

C 9.440033000 -1.183126000 0.729390000

H 9.163737000 -1.402625000 1.776024000

H 9.230707000 -2.083416000 0.125152000

H 10.521269000 -1.003407000 0.699030000

C 9.505758000 1.138612000 -0.207202000

H 9.233574000 2.042688000 0.365437000

H 10.574079000 0.950291000 -0.047497000

H 9.358091000 1.356615000 -1.279943000

Ir4NMe2PhTerpyPpyCl1t

Ir -1.641091000 0.012276000 -0.411948000

C -1.970465000 -0.049098000 1.562662000

N -3.729027000 0.011635000 -0.398207000

Cl -1.468485000 0.089598000 -2.895662000

N -1.316979000 2.026201000 -0.324939000

N 0.284556000 0.009659000 -0.291837000

N -1.314425000 -2.002480000 -0.452860000

C -0.996946000 -0.081587000 2.571925000

C -1.365606000 -0.121421000 3.919042000

C -2.716991000 -0.129698000 4.286873000

C -3.700362000 -0.098387000 3.300972000

C -3.336492000 -0.058778000 1.944854000

C -4.297788000 -0.025283000 0.840931000

C -5.692529000 -0.029142000 0.970652000

C -6.494186000 0.004803000 -0.164605000

C -5.889819000 0.042320000 -1.423973000

C -4.503307000 0.044422000 -1.497049000

C -2.243386000 2.999513000 -0.324106000

C -1.909659000 4.341810000 -0.235707000

C -0.554467000 4.695612000 -0.138670000

C 0.406716000 3.696051000 -0.131398000

C 0.019072000 2.351138000 -0.222688000

C 0.935738000 1.218160000 -0.214923000

C 2.311013000 1.229336000 -0.131702000

C 3.049510000 0.006073000 -0.113179000

C 2.311336000 -1.215286000 -0.186775000

C 0.936787000 -1.200778000 -0.281668000

C 0.021841000 -2.331928000 -0.367683000

C 0.410689000 -3.679641000 -0.361504000

C -0.549285000 -4.677541000 -0.436577000

C -1.904638000 -4.319622000 -0.514628000

C -2.239755000 -2.974769000 -0.517082000

C 4.496624000 0.003710000 -0.024856000

C 5.234557000 -1.187475000 0.245816000

C 5.266640000 1.191698000 -0.205232000

C 6.606175000 -1.200425000 0.325877000

C 6.638638000 1.196966000 -0.130202000

C 7.365856000 -0.004287000 0.137400000

H 0.063839000 -0.075382000 2.306651000

H -6.479244000 0.069709000 -2.342050000

H 2.828051000 -2.173611000 -0.196142000

H -6.143738000 -0.059122000 1.962824000

H 2.824478000 2.186410000 -0.055418000

H -0.591492000 -0.146160000 4.692158000

H 4.711364000 -2.127610000 0.422329000

H 4.772048000 2.135339000 -0.436969000

H 7.162628000 2.138019000 -0.293798000

H 7.103266000 -2.143651000 0.549505000

H 1.469282000 -3.934730000 -0.295155000

H 1.465288000 3.947462000 -0.052125000

H -3.277982000 -2.642625000 -0.574396000

H -0.250888000 -5.728319000 -0.432307000

H -2.692435000 -5.072393000 -0.572264000

H -3.281441000 2.670673000 -0.400172000

H -0.257115000 5.744260000 -0.067177000

H -2.698422000 5.095748000 -0.242432000

H -3.001581000 -0.160548000 5.341755000

H -4.753083000 -0.105166000 3.594983000

H -3.962720000 0.072674000 -2.446423000

H -7.582763000 0.001917000 -0.068899000

N 8.719668000 -0.009357000 0.208237000

C 9.447453000 -1.248248000 0.440505000

H 9.196037000 -1.671833000 1.425839000

H 9.211976000 -1.991433000 -0.336703000

H 10.522784000 -1.045852000 0.413118000

C 9.477141000 1.223318000 0.049800000

H 9.169835000 1.969770000 0.798260000

H 10.542472000 1.012583000 0.186600000

H 9.330669000 1.646652000 -0.956713000

Ir4OMePhTerpyPpyCl1s

Ir 1.420900000 -0.027669000 -0.405989000

C 1.810532000 0.058520000 1.563254000

N 3.494067000 -0.076133000 -0.459143000

Cl 1.131074000 -0.140478000 -2.857293000

N 1.047459000 -2.029569000 -0.270364000

N -0.520702000 0.023526000 -0.239728000

N 1.136397000 1.992046000 -0.469773000

C 0.874934000 0.129657000 2.604347000

C 1.292355000 0.182209000 3.937278000

C 2.655669000 0.164573000 4.256034000

C 3.602681000 0.094255000 3.236925000

C 3.188940000 0.041707000 1.895917000

C 4.108030000 -0.032428000 0.758801000

C 5.506021000 -0.059104000 0.836349000

C 6.263716000 -0.129981000 -0.327004000

C 5.613042000 -0.173218000 -1.562836000

C 4.225546000 -0.144089000 -1.586020000

C 1.944173000 -3.023291000 -0.272799000

C 1.565469000 -4.358565000 -0.165025000

C 0.211863000 -4.668706000 -0.049086000

C -0.722262000 -3.633407000 -0.041910000

C -0.286018000 -2.315304000 -0.150871000

C -1.184342000 -1.143861000 -0.144671000

C -2.570808000 -1.132887000 -0.038621000

C -3.266184000 0.093470000 -0.042931000

C -2.517617000 1.282712000 -0.156996000

C -1.131638000 1.223071000 -0.254268000

C -0.182687000 2.347186000 -0.379060000

C -0.559499000 3.687773000 -0.401705000

C 0.419328000 4.674483000 -0.514702000

C 1.757209000 4.294492000 -0.600503000

C 2.076107000 2.939679000 -0.572910000

C -4.735543000 0.132332000 0.067977000

C -5.389172000 1.206380000 0.707359000

C -5.532060000 -0.896758000 -0.460409000

C -6.770131000 1.243292000 0.815826000

C -6.922145000 -0.868586000 -0.367321000

C -7.555501000 0.206697000 0.277437000

H -0.195986000 0.144272000 2.382538000

H 6.167281000 -0.228818000 -2.501219000

H -3.023301000 2.247112000 -0.196632000

H 5.994156000 -0.023536000 1.810649000

H -3.113887000 -2.070845000 0.073361000

H 0.547327000 0.237363000 4.736709000

H -4.806859000 2.014000000 1.157891000

H -5.067529000 -1.732350000 -0.990075000

H -7.499241000 -1.682517000 -0.807048000

H -7.273922000 2.067689000 1.325414000

O -8.884931000 0.331542000 0.427228000

C -9.739149000 -0.677197000 -0.077806000

H -10.763303000 -0.365765000 0.164505000

H -9.538263000 -1.653548000 0.396120000

H -9.644034000 -0.778975000 -1.172741000

H -1.612807000 3.959789000 -0.329151000

H -1.786944000 -3.849819000 0.049999000

H 3.108017000 2.590970000 -0.636020000

H 0.135921000 5.728961000 -0.533110000

H 2.556391000 5.032426000 -0.687139000

H 2.990641000 -2.728674000 -0.365108000

H -0.118004000 -5.706189000 0.036936000

H 2.331383000 -5.135810000 -0.172365000

H 2.978442000 0.205527000 5.299446000

H 4.665061000 0.080625000 3.493161000

H 3.650896000 -0.174270000 -2.514815000

H 7.354807000 -0.151067000 -0.271255000

Ir4OMePhTerpyPpyCl1t

Ir 1.430446000 -0.035948000 -0.407868000

C 1.772563000 0.094912000 1.530471000

N 3.515062000 -0.093991000 -0.443962000

Cl 1.181651000 -0.103364000 -2.827394000

N 1.066694000 -2.029231000 -0.273776000

N -0.518706000 0.020747000 -0.272812000

N 1.135320000 1.994250000 -0.485373000

C 0.797845000 0.208084000 2.543480000

C 1.179216000 0.271915000 3.878172000

C 2.540225000 0.232879000 4.227660000

C 3.524658000 0.130346000 3.243444000

C 3.154694000 0.064643000 1.896581000

C 4.101538000 -0.036029000 0.785850000

C 5.494533000 -0.072037000 0.904087000

C 6.279254000 -0.167873000 -0.240994000

C 5.657750000 -0.225050000 -1.489505000

C 4.269312000 -0.185428000 -1.550013000

C 1.972932000 -3.031140000 -0.255410000

C 1.607022000 -4.353651000 -0.131265000

C 0.231638000 -4.671071000 -0.010548000

C -0.704125000 -3.655669000 -0.019116000

C -0.295235000 -2.314089000 -0.149567000

C -1.176072000 -1.174429000 -0.160707000

C -2.568304000 -1.142370000 -0.050532000

C -3.262513000 0.080399000 -0.064095000

C -2.513668000 1.284159000 -0.185549000

C -1.134565000 1.231566000 -0.289278000

C -0.196977000 2.345134000 -0.407666000

C -0.561121000 3.696367000 -0.433605000

C 0.417864000 4.674463000 -0.536540000

C 1.767731000 4.292962000 -0.609397000

C 2.082565000 2.946060000 -0.577368000

C -4.733333000 0.120020000 0.049602000

C -5.388945000 1.193069000 0.689449000

C -5.536092000 -0.906962000 -0.474499000

C -6.771109000 1.231290000 0.801885000

C -6.927000000 -0.881584000 -0.371311000

C -7.559146000 0.193856000 0.272212000

H -0.261642000 0.237998000 2.280024000

H 6.234333000 -0.299692000 -2.412884000

H -3.025760000 2.245517000 -0.223531000

H 5.959303000 -0.024052000 1.889015000

H -3.116767000 -2.076815000 0.073552000

H 0.420422000 0.352230000 4.660905000

H -4.805902000 2.002726000 1.135924000

H -5.073738000 -1.742404000 -1.006816000

H -7.504939000 -1.697954000 -0.806013000

H -7.272148000 2.058641000 1.310005000

O -8.892216000 0.318926000 0.427759000

C -9.742901000 -0.693185000 -0.071469000

H -10.768391000 -0.386368000 0.172301000

H -9.538025000 -1.668839000 0.403258000

H -9.651649000 -0.799404000 -1.166758000

H -1.614555000 3.971306000 -0.371093000

H -1.767042000 -3.881990000 0.077386000

H 3.114253000 2.594475000 -0.629963000

H 0.138117000 5.729951000 -0.558066000

H 2.566409000 5.031723000 -0.690270000

H 3.017995000 -2.731218000 -0.350377000

H -0.088015000 -5.710543000 0.091139000

H 2.374183000 -5.129064000 -0.128683000

H 2.833150000 0.285020000 5.279167000

H 4.575229000 0.103261000 3.539719000

H 3.722342000 -0.225459000 -2.494395000

H 7.368205000 -0.197069000 -0.159335000

Ir35CF3PhTerpyPpyCl1s

Ir -2.293106000 0.153372000 -0.382355000

C -2.645625000 -0.543796000 1.470307000

N -4.367355000 0.161370000 -0.394880000

Cl -2.052510000 1.019335000 -2.681671000

N -1.961937000 2.025855000 0.356546000

N -0.349706000 0.102694000 -0.262923000

N -1.972356000 -1.741483000 -1.065705000

C -1.689175000 -0.903115000 2.429181000

C -2.080394000 -1.375051000 3.685430000

C -3.437524000 -1.496622000 4.007203000

C -4.404792000 -1.144680000 3.068646000

C -4.017449000 -0.670337000 1.804854000

C -4.958246000 -0.275567000 0.755044000

C -6.354745000 -0.314956000 0.849862000

C -7.134488000 0.092488000 -0.226436000

C -6.507306000 0.537761000 -1.392938000

C -5.120211000 0.556842000 -1.437356000

C -2.879661000 2.948566000 0.668471000

C -2.527112000 4.196839000 1.174487000

C -1.178672000 4.492311000 1.363632000

C -0.222234000 3.529112000 1.043619000

C -0.633045000 2.296610000 0.542113000

C 0.288516000 1.202854000 0.179264000

C 1.678202000 1.194697000 0.260423000

C 2.386658000 0.043470000 -0.124626000

C 1.671721000 -1.073927000 -0.589648000

C 0.282137000 -1.020651000 -0.653373000

C -0.645383000 -2.075020000 -1.106832000

C -0.242743000 -3.333630000 -1.546572000

C -1.205220000 -4.261915000 -1.942604000

C -2.551543000 -3.907452000 -1.887096000

C -2.895855000 -2.634421000 -1.440674000

C 3.864983000 0.006960000 -0.042369000

C 4.526974000 -1.161524000 0.360805000

C 4.627020000 1.138771000 -0.366485000

C 5.918836000 -1.193208000 0.434862000

C 6.018256000 1.097003000 -0.288340000

C 6.675064000 -0.066962000 0.111294000

H -0.622457000 -0.817110000 2.203528000

H -7.079405000 0.867184000 -2.261820000

H 2.201512000 -1.966871000 -0.921017000

H -6.824758000 -0.665807000 1.768897000

H 2.209261000 2.066044000 0.643104000

H -1.319541000 -1.650752000 4.421756000

H 3.956591000 -2.047407000 0.646876000

H 4.138590000 2.052825000 -0.709718000

H 0.816613000 -3.589585000 -1.578083000

H 0.838823000 3.736242000 1.184936000

H -3.935196000 -2.308544000 -1.378569000

H -0.902109000 -5.252367000 -2.288524000

H -3.338316000 -4.602954000 -2.183692000

H -3.921141000 2.668840000 0.502805000

H -0.869363000 5.462393000 1.758327000

H -3.309331000 4.919064000 1.413701000

H -3.739939000 -1.865594000 4.990525000

H -5.462042000 -1.241616000 3.327762000

H -4.563754000 0.891402000 -2.316001000

H -8.224568000 0.063295000 -0.156763000

H 7.764226000 -0.095986000 0.169906000

C 6.821133000 2.338428000 -0.592794000

F 6.204817000 3.122901000 -1.487454000

F 7.012594000 3.076690000 0.513604000

F 8.032690000 2.039048000 -1.080386000

C 6.614197000 -2.475295000 0.823687000

F 6.853290000 -3.244519000 -0.251835000

F 7.795081000 -2.242180000 1.411732000

F 5.874190000 -3.203893000 1.671037000

Ir35CF3PhTerpyPpyCl1t

Ir -2.304377000 0.114226000 -0.390780000

C -2.629342000 -0.415223000 1.479136000

N -4.389315000 0.129539000 -0.411665000

Cl -2.068248000 0.769457000 -2.718804000

N -1.967759000 2.047771000 0.182177000

N -0.354887000 0.077388000 -0.276616000

N -1.975835000 -1.835521000 -0.913961000

C -1.645091000 -0.695770000 2.450877000

C -2.016549000 -1.059939000 3.738622000

C -3.376904000 -1.152507000 4.083281000

C -4.370541000 -0.880415000 3.141269000

C -4.010948000 -0.512029000 1.841653000

C -4.966677000 -0.203989000 0.778033000

C -6.359250000 -0.228516000 0.902940000

C -7.153246000 0.089093000 -0.194791000

C -6.541128000 0.426949000 -1.402766000

C -5.152372000 0.436123000 -1.471899000

C -2.894756000 2.995647000 0.422223000

C -2.551837000 4.272634000 0.823623000

C -1.189761000 4.586325000 0.992015000

C -0.231799000 3.614280000 0.754179000

C -0.622001000 2.329942000 0.346164000

C 0.287492000 1.229604000 0.076022000

C 1.672107000 1.217264000 0.152964000

C 2.390982000 0.030713000 -0.131456000

C 1.666545000 -1.130404000 -0.495398000

C 0.282256000 -1.093895000 -0.570702000

C -0.631450000 -2.167348000 -0.921970000

C -0.246293000 -3.477204000 -1.244715000

C -1.207907000 -4.426601000 -1.549563000

C -2.568549000 -4.065891000 -1.528241000

C -2.906383000 -2.765941000 -1.203350000

C 3.862593000 0.004664000 -0.049853000

C 4.550191000 -1.174339000 0.292121000

C 4.625179000 1.157397000 -0.311228000

C 5.941168000 -1.195012000 0.364465000

C 6.015769000 1.128948000 -0.227455000

C 6.690416000 -0.045356000 0.108769000

H -0.586787000 -0.625907000 2.190451000

H -7.125041000 0.682519000 -2.288406000

H 2.194687000 -2.050159000 -0.747351000

H -6.816448000 -0.494672000 1.856041000

H 2.201293000 2.118732000 0.462171000

H -1.251508000 -1.275851000 4.488859000

H 3.997140000 -2.083541000 0.534005000

H 4.135340000 2.084821000 -0.613031000

H 0.812192000 -3.739986000 -1.250911000

H 0.827729000 3.838471000 0.883353000

H -3.945021000 -2.432893000 -1.170358000

H -0.909661000 -5.446751000 -1.801122000

H -3.354269000 -4.785917000 -1.760897000

H -3.934550000 2.697762000 0.277473000

H -0.887470000 5.586634000 1.309724000

H -3.334609000 5.011210000 1.001665000

H -3.661679000 -1.439986000 5.098385000

H -5.419513000 -0.959563000 3.433621000

H -4.612880000 0.691548000 -2.386379000

H -8.242011000 0.072884000 -0.107653000

H 7.779359000 -0.064288000 0.170338000

C 6.799555000 2.396859000 -0.457742000

F 6.185768000 3.214813000 -1.325339000

F 6.958912000 3.087183000 0.685463000

F 8.026282000 2.146085000 -0.937125000

C 6.653385000 -2.487045000 0.677818000

F 6.967642000 -3.156581000 -0.445445000

F 7.800256000 -2.275864000 1.339995000

F 5.900738000 -3.307809000 1.424666000

8 Photophysical Data

8.1 Extinction Coefficients

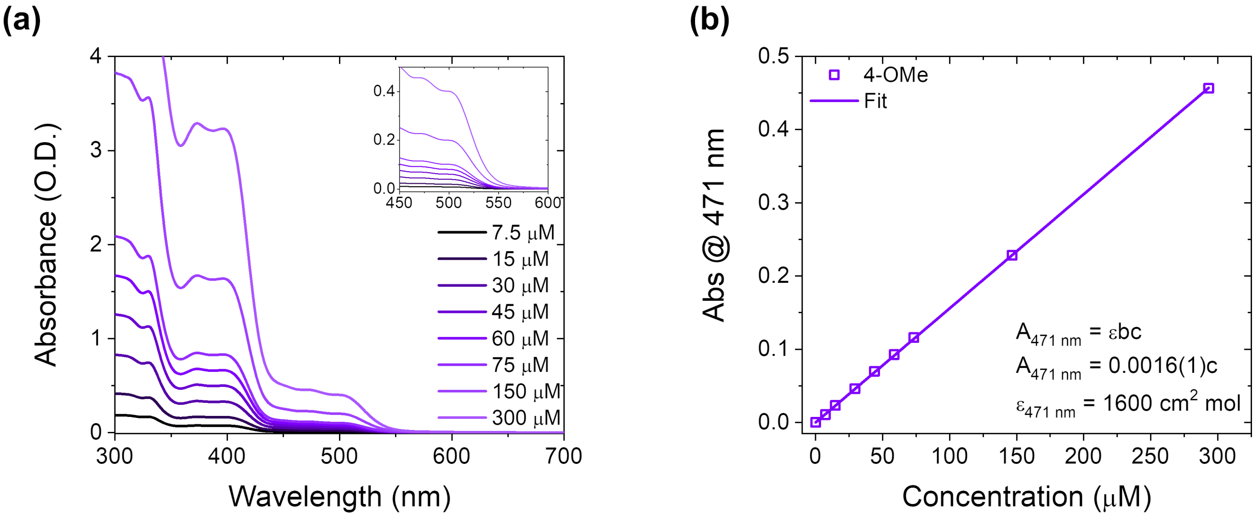

Figure S55: (a) Absorption spectra of [Ir(**4-OMePhTerpy**)(ppy)Cl](PF_6) in Acetonitrile at various concentrations; (b) Extinction coefficient Beer-Lambert plot at the highest energy MLCT band (λ = 471 nm) for [Ir(**4-OMePhTerpy**)(ppy)Cl](PF_6) in Acetonitrile.

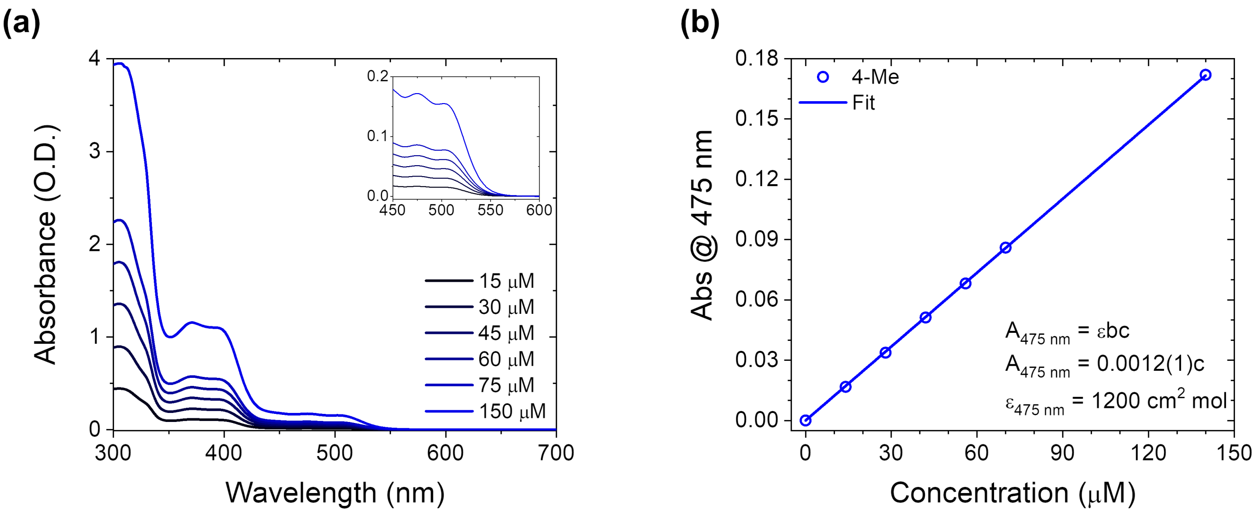

Figure S56: (a) Absorption spectra of [Ir(**4-MePhTerpy**)(ppy)Cl](PF_6) in Acetonitrile at various concentrations; (b) Extinction coefficient Beer-Lambert plot at the highest energy MLCT band (λ = 475 nm) for [Ir(**4-MePhTerpy**)(ppy)Cl](PF_6) in Acetonitrile.

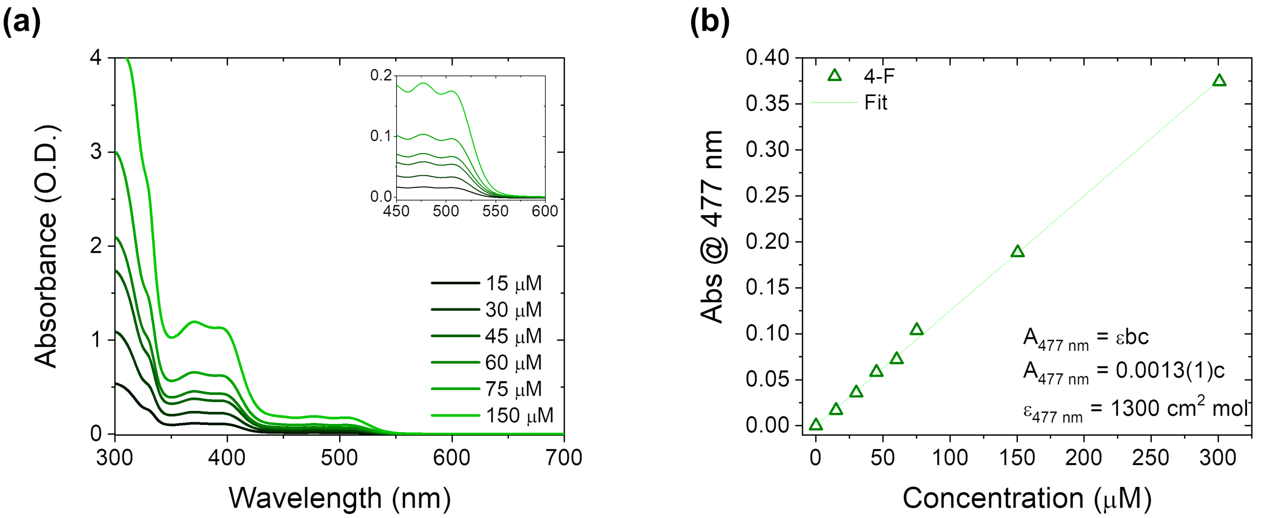

Figure S57: (a) Absorption spectra of [Ir(**4-FPhTerpy**)(ppy)Cl](PF_6) in Acetonitrile at various concentrations; (b) Extinction coefficient Beer-Lambert plot at the highest energy MLCT band (λ = 477 nm) for [Ir(**4-FPhTerpy**)(ppy)Cl](PF_6) in Acetonitrile.

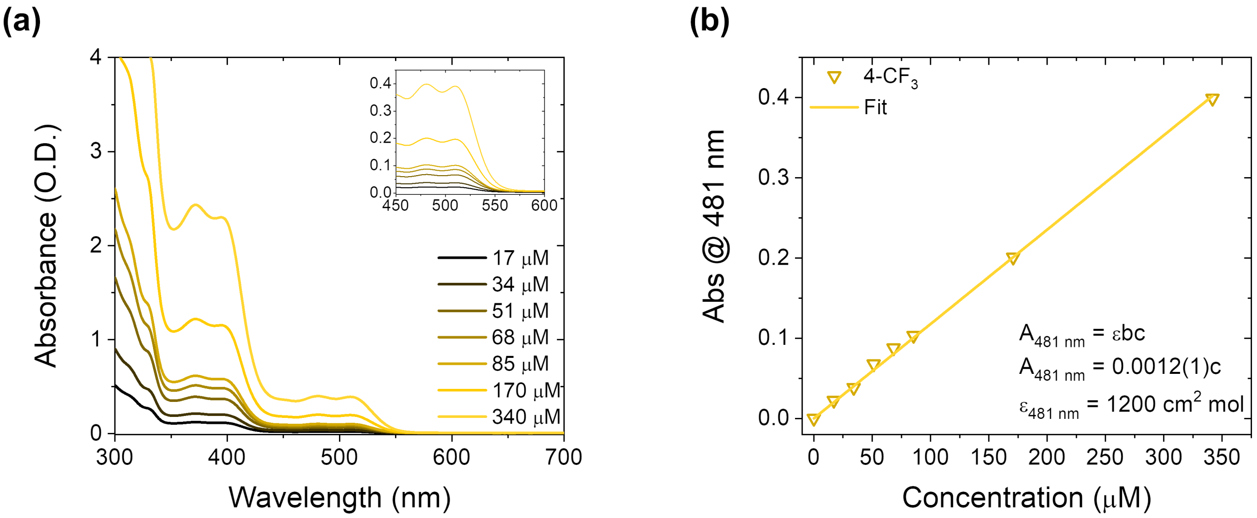

Figure S58: (a) Absorption spectra of [Ir(**4-CF$_3$PhTerpy**)(ppy)Cl](PF$_6$) in Acetonitrile at various concentrations; (b) Extinction coefficient Beer-Lambert plot at the highest energy MLCT band (λ = 481 nm) for [Ir(**4-CF$_3$PhTerpy**)(ppy)Cl](PF$_6$) in Acetonitrile.

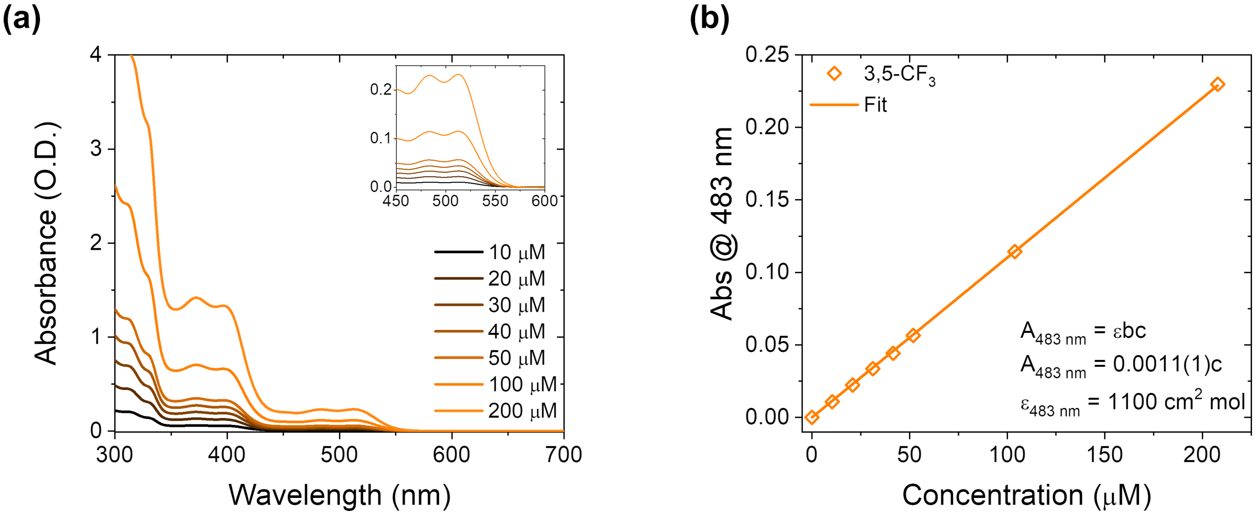

Figure S59: (a) Absorption spectra of [Ir(**3,5-CF$_3$OMePhTerpy**)(ppy)Cl](PF$_6$) in Acetonitrile at various concentrations; (b) Extinction coefficient Beer-Lambert plot at the highest energy MLCT band (λ = 483 nm) for [Ir(**3,5-CF$_3$OMePhTerpy**)(ppy)Cl](PF$_6$) in Acetonitrile.

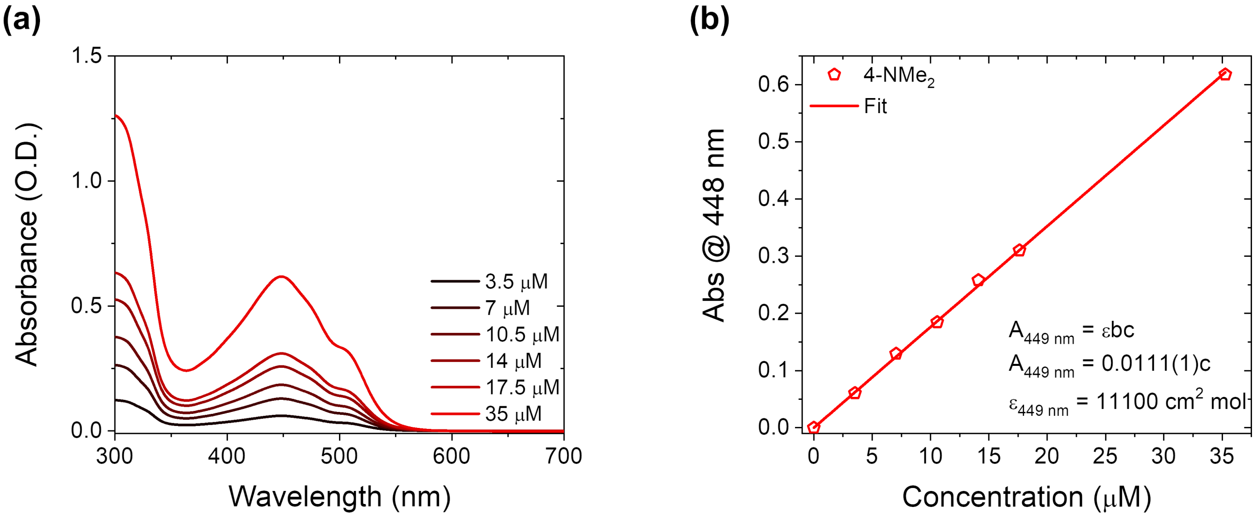

Figure S60: (a) Absorption spectra of [Ir(**4-NMe$_2$PhTerpy**)(ppy)Cl](PF$_6$) in Acetonitrile at various concentrations; (b) Extinction coefficient Beer-Lambert plot at the highest energy MLCT band ($\lambda = 448$ nm) for [Ir(**4-NMe$_2$PhTerpy**)(ppy)Cl](PF$_6$) in Acetonitrile.

8.2 Franck-Condon Line Shape Analysis

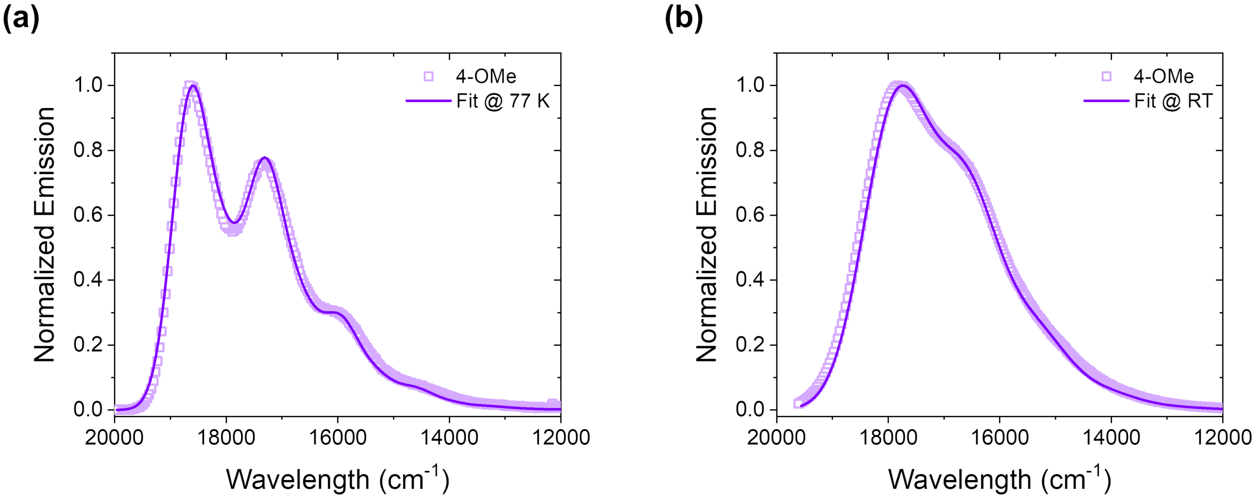

Figure S61: Emission spectra (purple squares) and calculated FCLSA fit (solid line) for [Ir(**4-OMePhTerpy**)(ppy)Cl](PF_6) (a) in a 4:5 prop:but glass at 77 K (λ_{ex} = 450 nm) and (b) freeze-pump-thaw degassed Acetonitrile at room temperature (λ_{ex} = 500 nm).

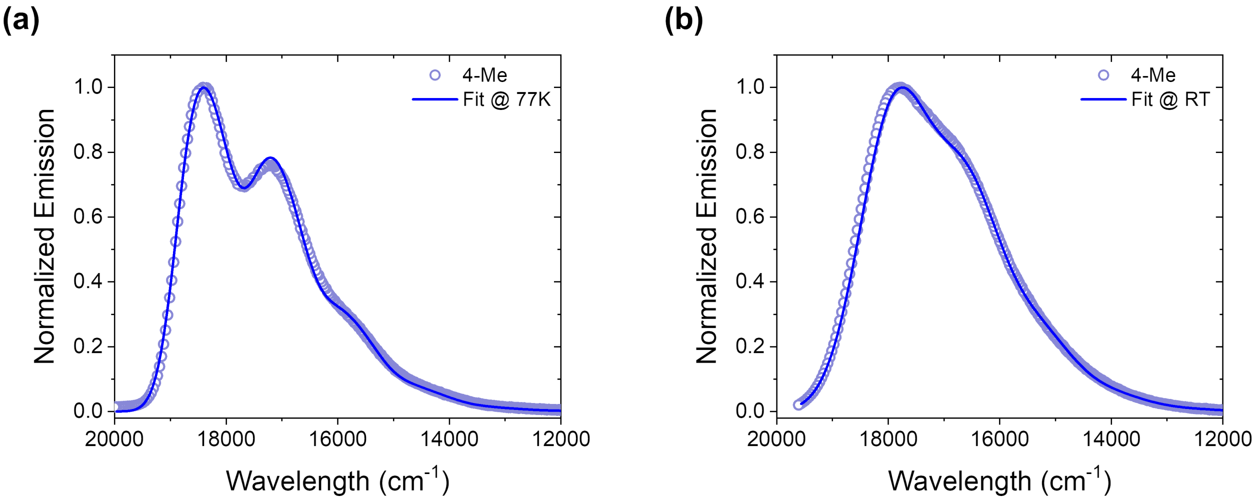

Figure S62: Emission spectra (blue circles) and calculated FCLSA fit (solid line) for [Ir(**4-MePhTerpy**)(ppy)Cl](PF_6) (a) in a 4:5 prop:but glass at 77 K (λ_{ex} = 450 nm) and (b) freeze-pump-thaw degassed Acetonitrile at room temperature (λ_{ex} = 500 nm).

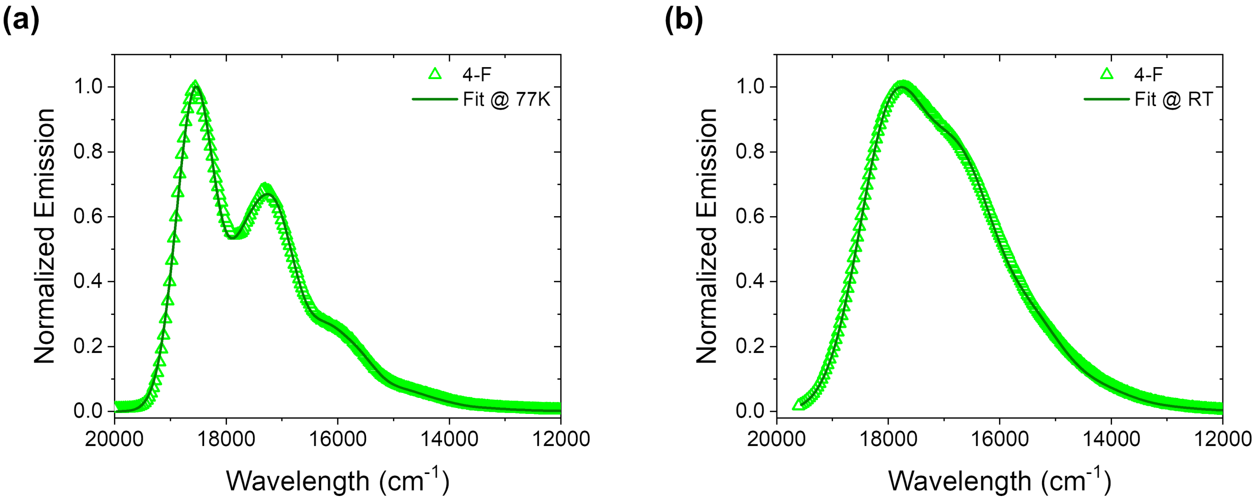

Figure S63: Emission spectra (green up-triangles) and calculated FCLSA fit (solid line) for [Ir(**4-FPhTerpy**)(ppy)Cl](PF_6) (a) in a 4:5 prop:but glass at 77 K (λ_{ex} = 450 nm) and (b) freeze-pump-thaw degassed Acetonitrile at room temperature (λ_{ex} = 500 nm).

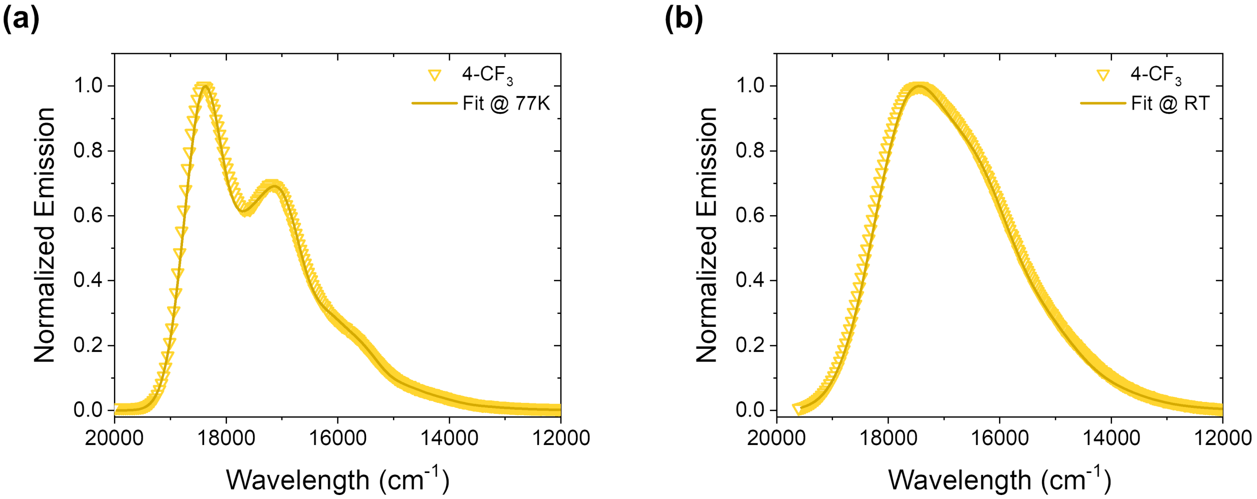

Figure S64: Emission spectra (yellow down-triangles) and calculated FCLSA fit (solid line) for [Ir(**4-CF$_3$PhTerpy**)(ppy)Cl](PF_6) (a) in a 4:5 prop:but glass at 77 K (λ_{ex} = 450 nm) and (b) freeze-pump-thaw degassed Acetonitrile at room temperature (λ_{ex} = 500 nm).

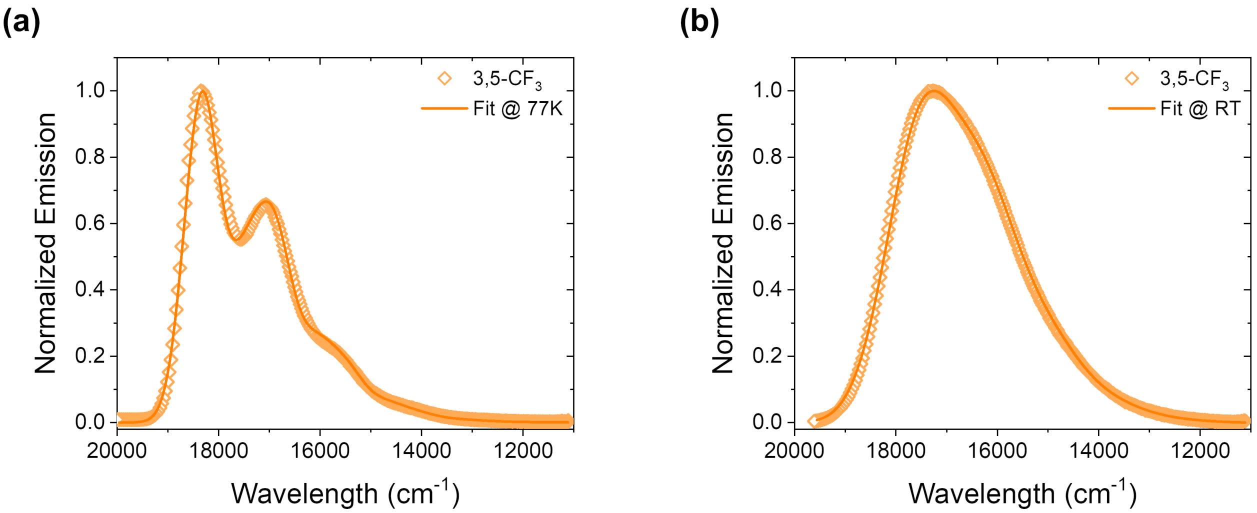

Figure S65: Emission spectra (orange diamonds) and calculated FCLSA fit (solid line) for $[Ir(\mathbf{3,5\text{-}CF_3PhTerpy})(ppy)Cl](PF_6)$ (a) in a 4:5 prop:but glass at 77 K (λ_{ex} = 450 nm) and (b) freeze-pump-thaw degassed Acetonitrile at room temperature (λ_{ex} = 500 nm).

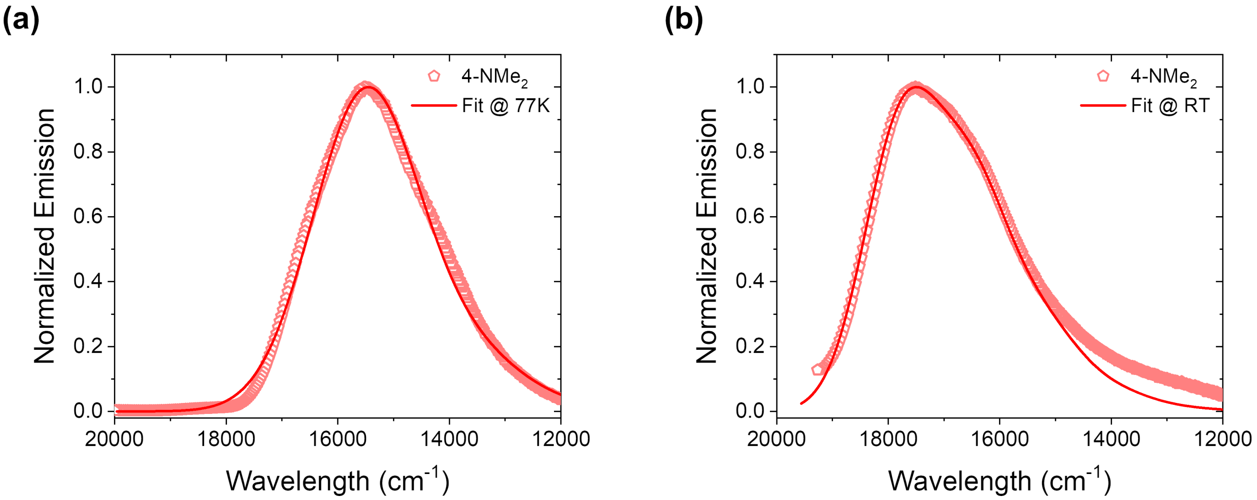

Figure S66: Emission spectra (red pentagons) and calculated FCLSA fit (solid line) for $[Ir(\mathbf{4\text{-}NMe_2PhTerpy})(ppy)Cl](PF_6)$ (a) in a 4:5 prop:but glass at 77 K (λ_{ex} = 450 nm) and (b) freeze-pump-thaw degassed Acetonitrile at room temperature (λ_{ex} = 500 nm).

Table S 26: Franck-Condon line shape analysis (FCLSA) parameters calculated for [Ir(**RPhTerpy**)(ppy)Cl](PF_6)complexes in a 4:5 prop:but glass at 77 K (λ_{ex} = 550 nm).

textbfComplex	E_{00} / **cm^{-1}**	$\Delta\nu_{1/2}$/ **cm^{-1}**	S_{M1}	$\hbar\omega_1$ / **cm^{-1}**	S_{M2}	$\hbar\omega_2$ / **cm^{-1}**
[Ir(**4-OMePhTerpy**)(ppy)Cl](PF_6)	18649	774	0.90	1352	0.49	628
[Ir(**4-MePhTerpy**)(ppy)Cl](PF_6)	18638	761	0.89	1361	0.95	469
[Ir(**4-FPhTerpy**)(ppy)Cl](PF_6)	18552	808	0.67	1441	0.51	873
[Ir(**4-CF$_3$PhTerpy**)(ppy)Cl](PF_6)	18403	818	0.68	1398	0.54	798
[Ir(**3,5-CF$_3$PhTerpy**)(ppy)Cl](PF_6)	18333	812	0.67	1406	0.49	839
[Ir(**4-NMe$_2$PhTerpy**)(ppy)Cl](PF_6)	15493	2258	0.23	1930	–	–

Table S 27: Franck-Condon line shape analysis (FCLSA) parameters calculated for [Ir(**RPhTerpy**)(ppy)Cl](PF_6)complexes in freeze-pump-thaw degassed Acetonitrile at room temperature (λ_{ex} = 500 nm).

**Complex**	E_{00} / **cm^{-1}**	$\Delta\nu_{1/2}$ / **cm^{-1}**	S_M	$\hbar\omega$ / **cm^{-1}**	**Chromaticity**
[Ir(**4-OMePhTerpy**)(ppy)Cl](PF_6)	17859	1372	0.87	1256	(0.482, 0.514)
[Ir(**4-MePhTerpy**)(ppy)Cl](PF_6)	17894	1460	0.88	1300	(0.479, 0.517)
[Ir(**4-FPhTerpy**)(ppy)Cl](PF_6)	17930	1400	0.94	1240	(0.482, 0.514)
[Ir(**4-CF$_3$PhTerpy**)(ppy)Cl](PF_6)	17652	1500	0.88	1240	(0.507, 0.490)
[Ir(**3,5-CF$_3$PhTerpy**)(ppy)Cl](PF_6)	17521	1600	0.90	1260	(0.523, 0.475)
[Ir(**4-NMe$_2$PhTerpy**)(ppy)Cl](PF_6)	17763	1600	0.92	1280	(0.496, 0.499)

8.3 Miscellaneous Photophysical Data

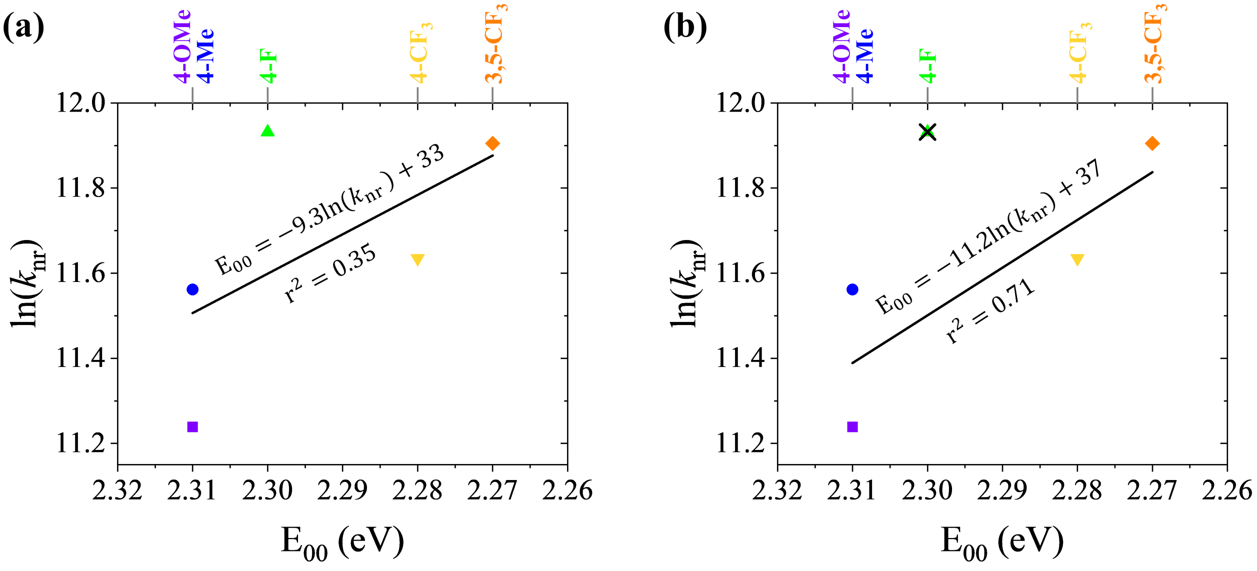

Figure S67: Energy Gap law plots (a) with [Ir(**4-FPhTerpy**)(ppy)Cl](PF_6) and (b) without [Ir(**4-FPhTerpy**)(ppy)Cl](PF_6).

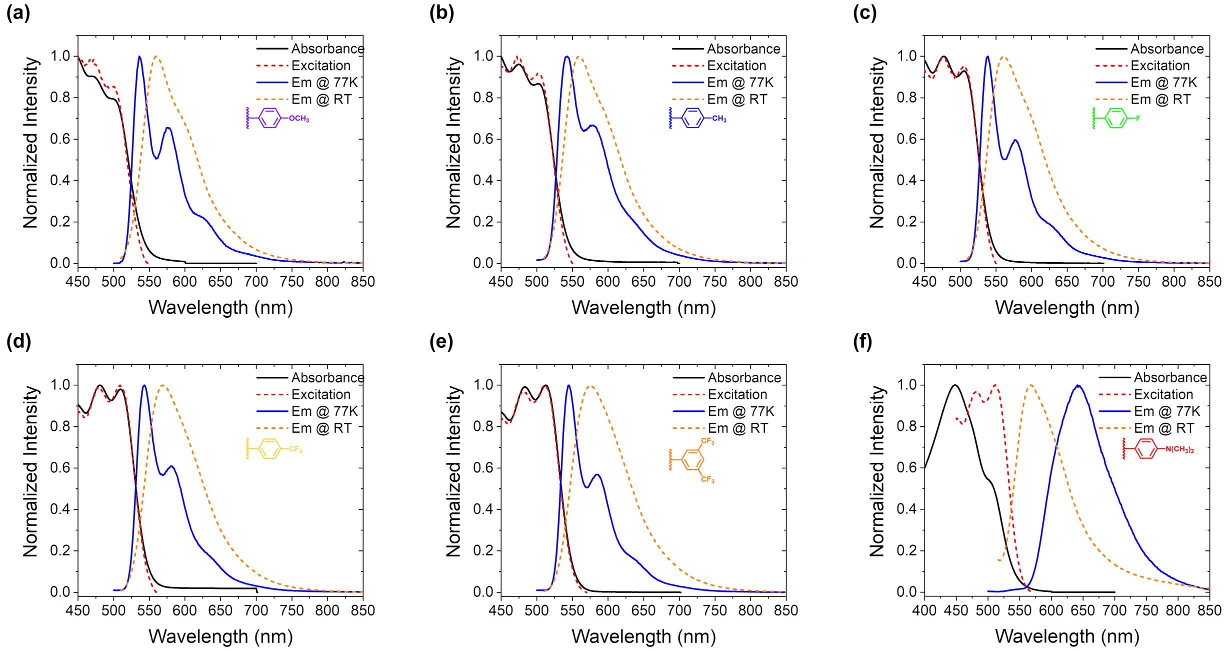

Figure S68: Absorbance (black solid line), emission at 77 K (blue solid line, prop:but glass, λ_{ex} = 450 nm), and emission at room temperature (orange dashed line, acetonitrile, λ_{ex} = 500 nm) for (a) [Ir(**4-OMePhTerpy**)(ppy)Cl](PF_6); (b) [Ir(**4-MePhTerpy**)(ppy)Cl](PF_6); (c) [Ir(**4-FPhTerpy**)(ppy)Cl](PF_6); (d) [Ir(**3-CF$_3$PhTerpy**)(ppy)Cl](PF_6); (e) [Ir(**3,5-CF$_3$PhTerpy**)(ppy)Cl](PF_6); (f) [Ir(**4-NMe$_2$PhTerpy**)(ppy)Cl](PF_6).

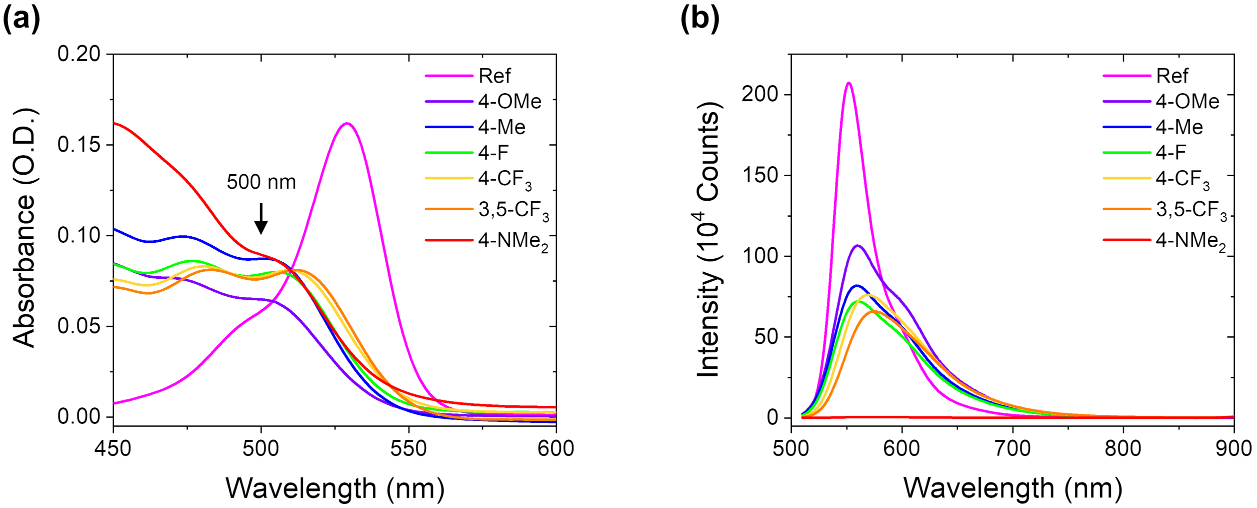

Figure S69: Absorbance (a) and emission (b) from quantum yield measurements. Traces: Rhodamine 6G (Ref, pink); [Ir(**4-OMePhTerpy**)(ppy)Cl](PF_6) (purple); [Ir(**4-MePhTerpy**)(ppy)Cl](PF_6) (blue); [Ir(**4-FPhTerpy**)(ppy)Cl](PF_6) (green); [Ir(**3-CF$_3$PhTerpy**)(ppy)Cl](PF_6) (yellow); [Ir(**3,5-CF$_3$PhTerpy**)(ppy)Cl](PF_6) (orange); [Ir(**4-NMe$_2$PhTerpy**)(ppy)Cl](PF_6) (red).

9 Mass Spectra

[Page intentionally left black due to formatting requirements.]

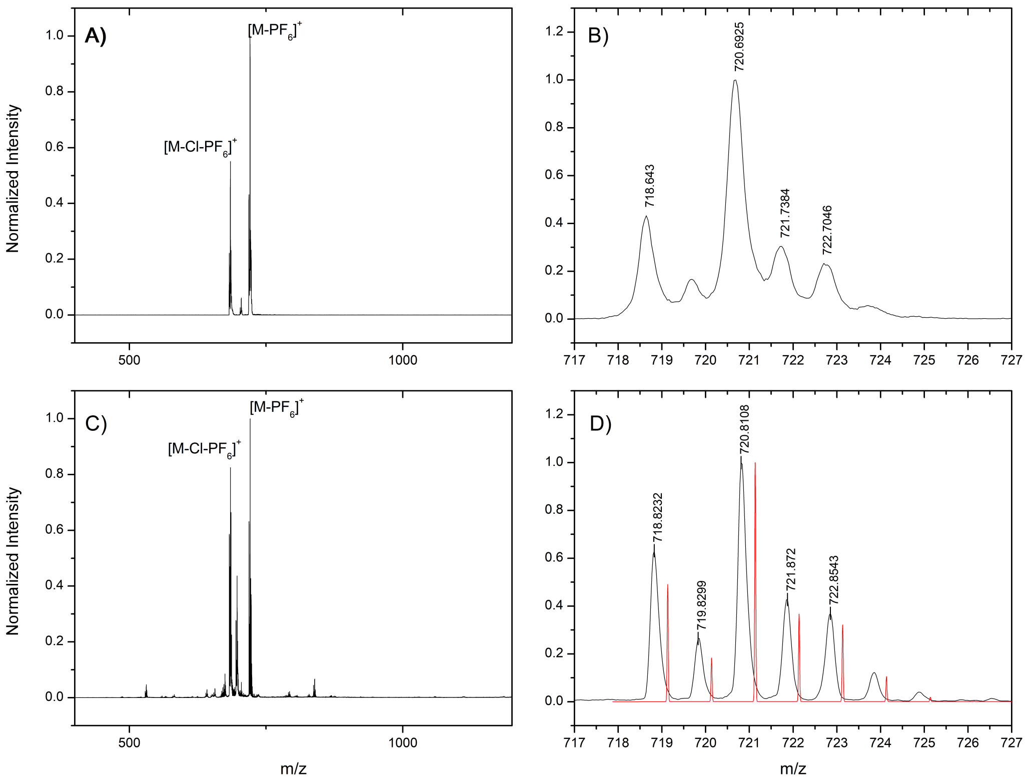

Figure S70: Mass spectra of [Ir(**4-OMePhTerpy**)(ppy)Cl](PF_6). A) LDI-TOF. B) LDI-TOF of [Ir(**4-OMePhTerpy**)(ppy)Cl]$^+$ isotopic window. C) MALDI-TOF using DHB matrix. D) MALDI-TOF of [Ir(**4-OMePhTerpy**)(ppy)Cl]$^+$ isotopic window with simulated mass spectrum (red).

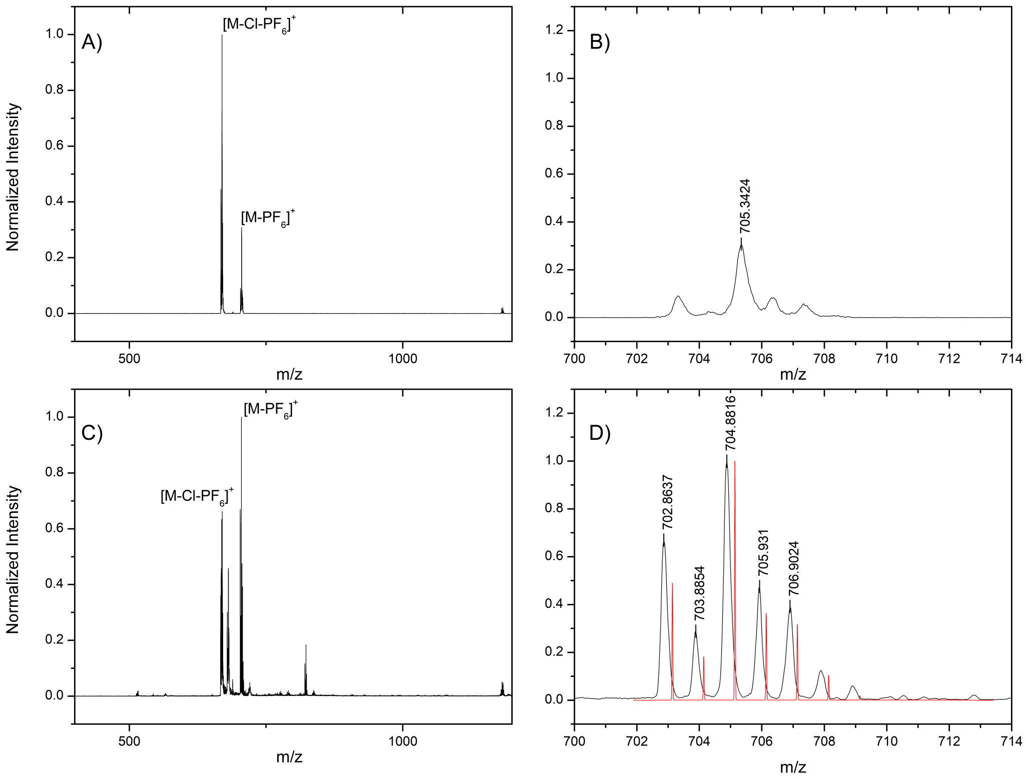

Figure S71: Mass spectra of [Ir(**4-MePhTerpy**)(ppy)Cl](PF_6). A) LDI-TOF. B) LDI-TOF of [Ir(**4-MePhTerpy**)(ppy)Cl]$^+$ isotopic window. C) MALDI-TOF using DHB matrix. D) MALDI-TOF of [Ir(**4-MePhTerpy**)(ppy)Cl]$^+$ isotopic window with simulated mass spectrum (red).

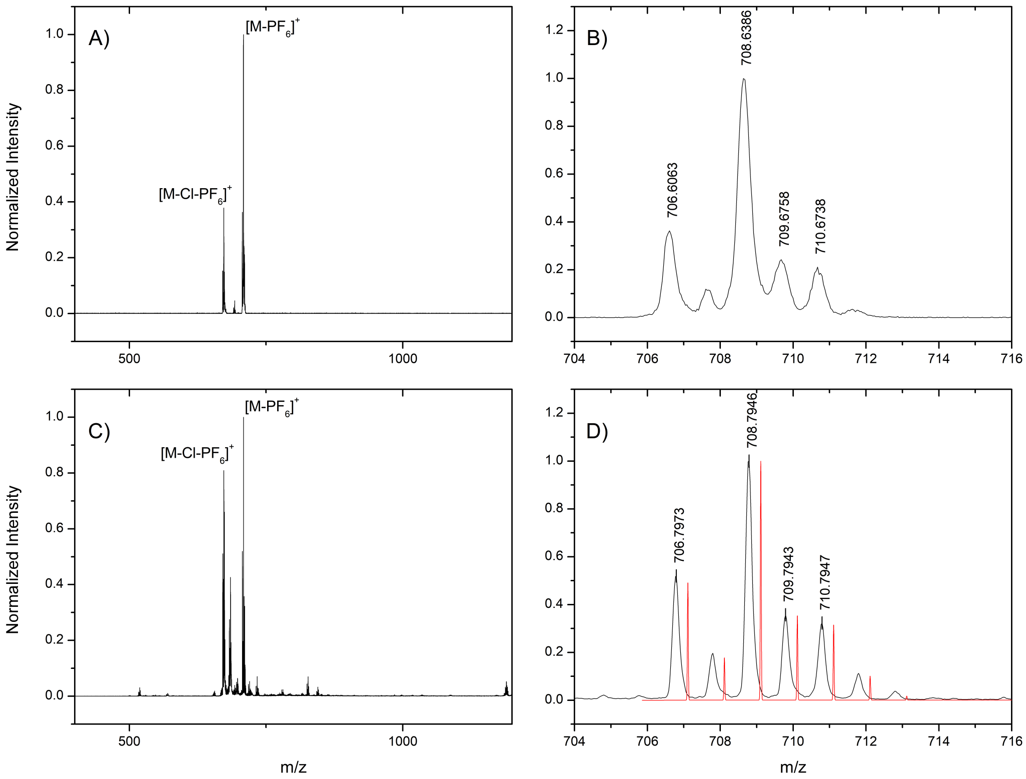

Figure S72: Mass spectra of [Ir(**4-FPhTerpy**)(ppy)Cl](PF_6). A) LDI-TOF. B) LDI-TOF of [Ir(**4-FPhTerpy**)(ppy)Cl]$^+$ isotopic window. C) MALDI-TOF using DHB matrix. D) MALDI-TOF of [Ir(**4-FPhTerpy**)(ppy)Cl]$^+$ isotopic window with simulated mass spectrum (red).

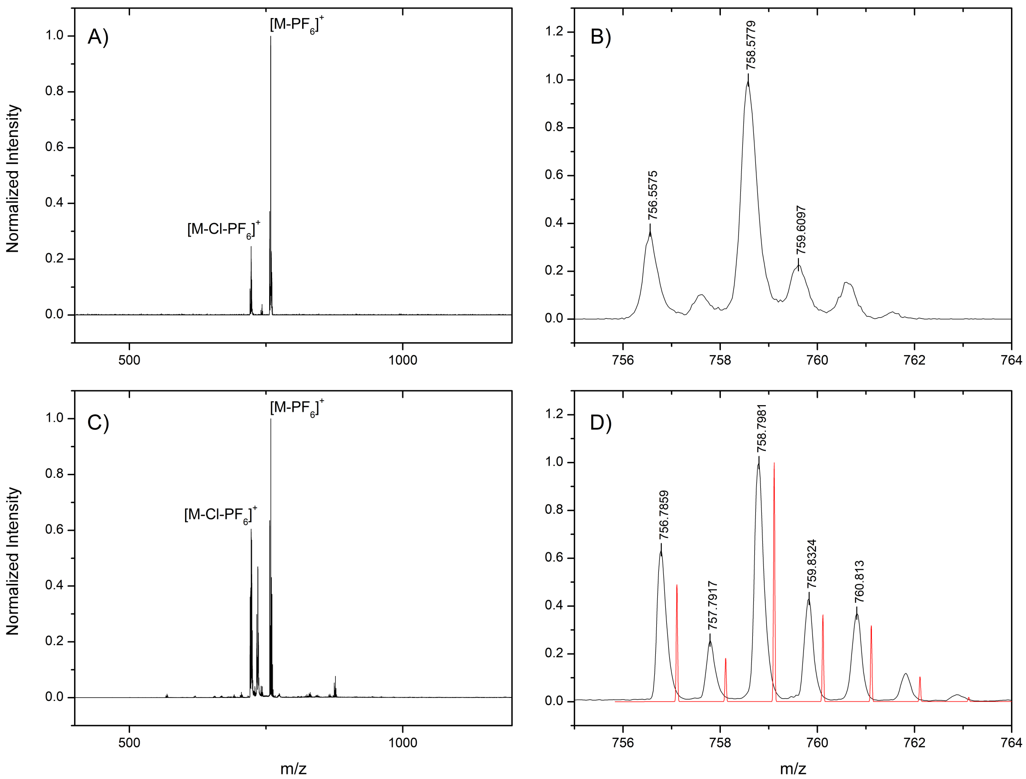

Figure S73: Mass spectra of [Ir(**4-CF$_3$PhTerpy**)(ppy)Cl](PF$_6$). A) LDI-TOF. B) LDI-TOF of [Ir(**4-CF$_3$PhTerpy**)(ppy)Cl]$^+$ isotopic window. C) MALDI-TOF using DHB matrix. D) MALDI-TOF of [Ir(**4-CF$_3$PhTerpy**)(ppy)Cl]$^+$ isotopic window with simulated mass spectrum (red).

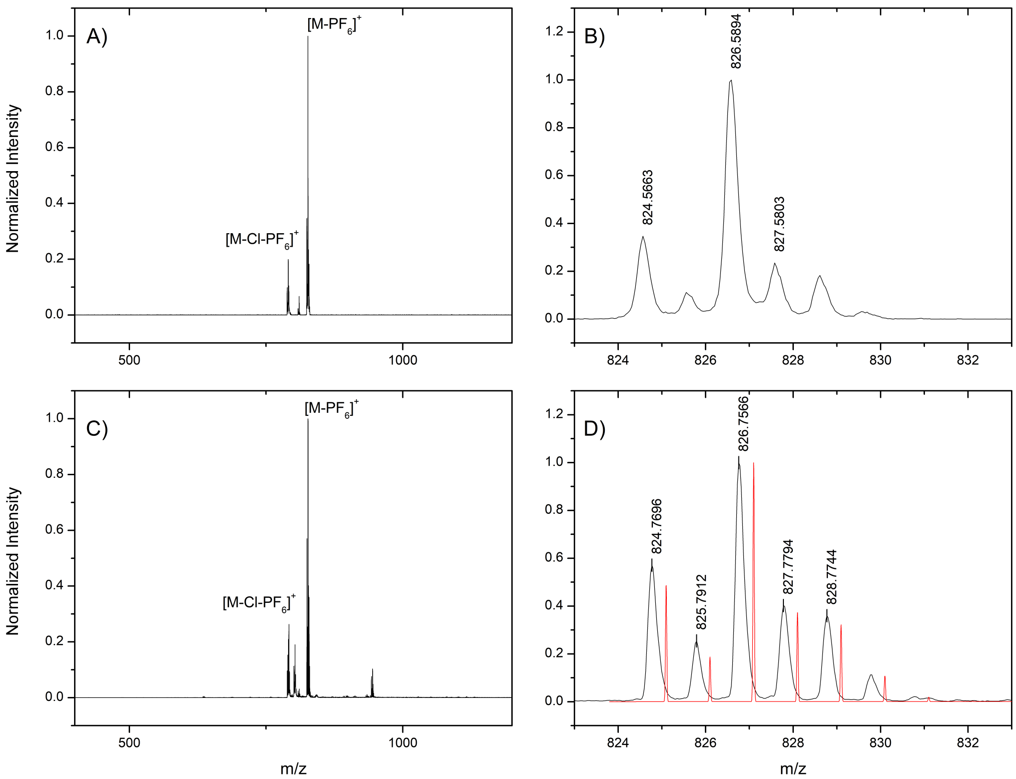

Figure S74: Mass spectra of [Ir(**3,5-CF$_3$PhTerpy**)(ppy)Cl](PF$_6$). A) LDI-TOF. B) LDI-TOF of [Ir(**3,5-CF$_3$PhTerpy**)(ppy)Cl]$^+$ isotopic window. C) MALDI-TOF using DHB matrix. D) MALDI-TOF of [Ir(**3,5-CF$_3$PhTerpy**)(ppy)Cl]$^+$ isotopic window with simulated mass spectrum (red).

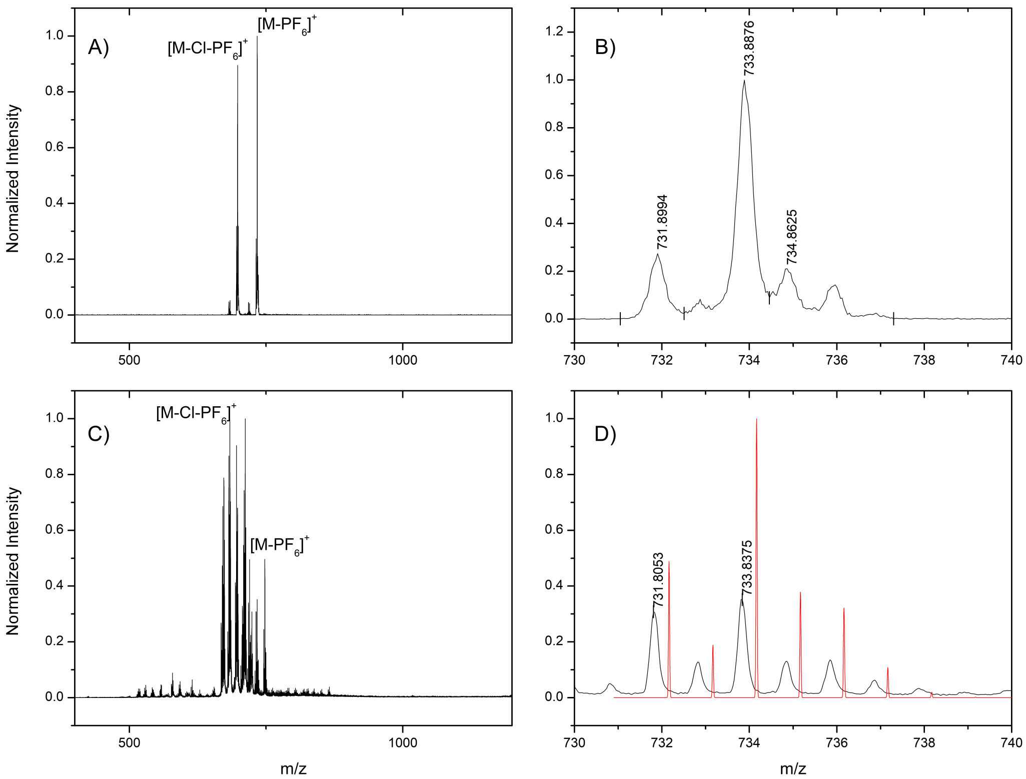

Figure S75: Mass spectra of [Ir(**4-NMe$_2$PhTerpy**)(ppy)Cl](PF_6). A) LDI-TOF. B) LDI-TOF of [Ir(**4-NMe$_2$PhTerpy**)(ppy)Cl]$^+$ isotopic window. C) MALDI-TOF using DHB matrix. D) MALDI-TOF of [Ir(**4-NMe$_2$PhTerpy**)(ppy)Cl]$^+$ isotopic window with simulated mass spectrum (red).

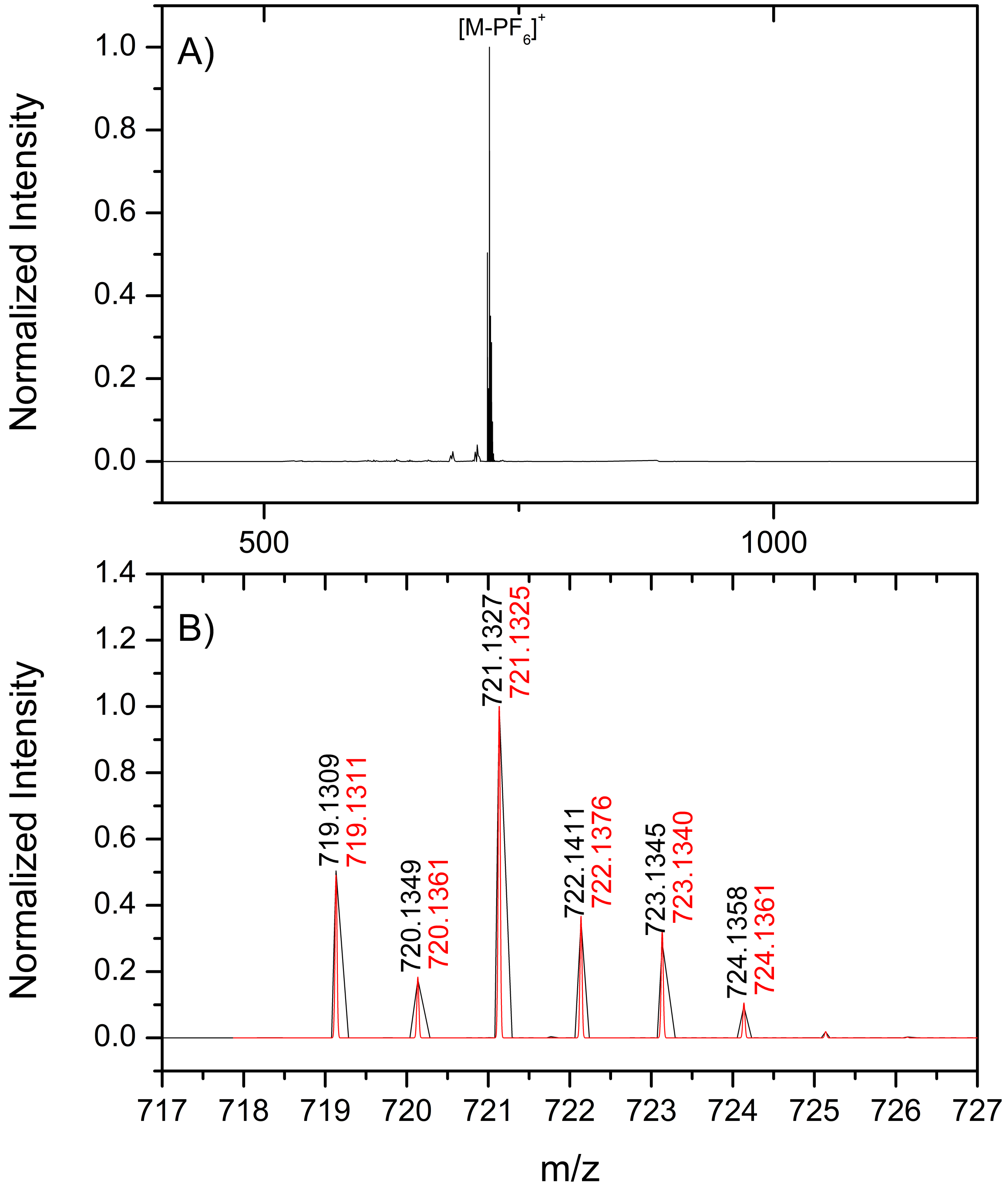

Figure S76: ESI-MS of [Ir(**4-OMePhTerpy**)(ppy)Cl](PF_6). A) Displays the full MS window analyzed and B) is an expanded region a the [M-PF_6]$^+$ peak. The black traces are the experimental data while red trace is the simulated mass spectrum isotopic window.

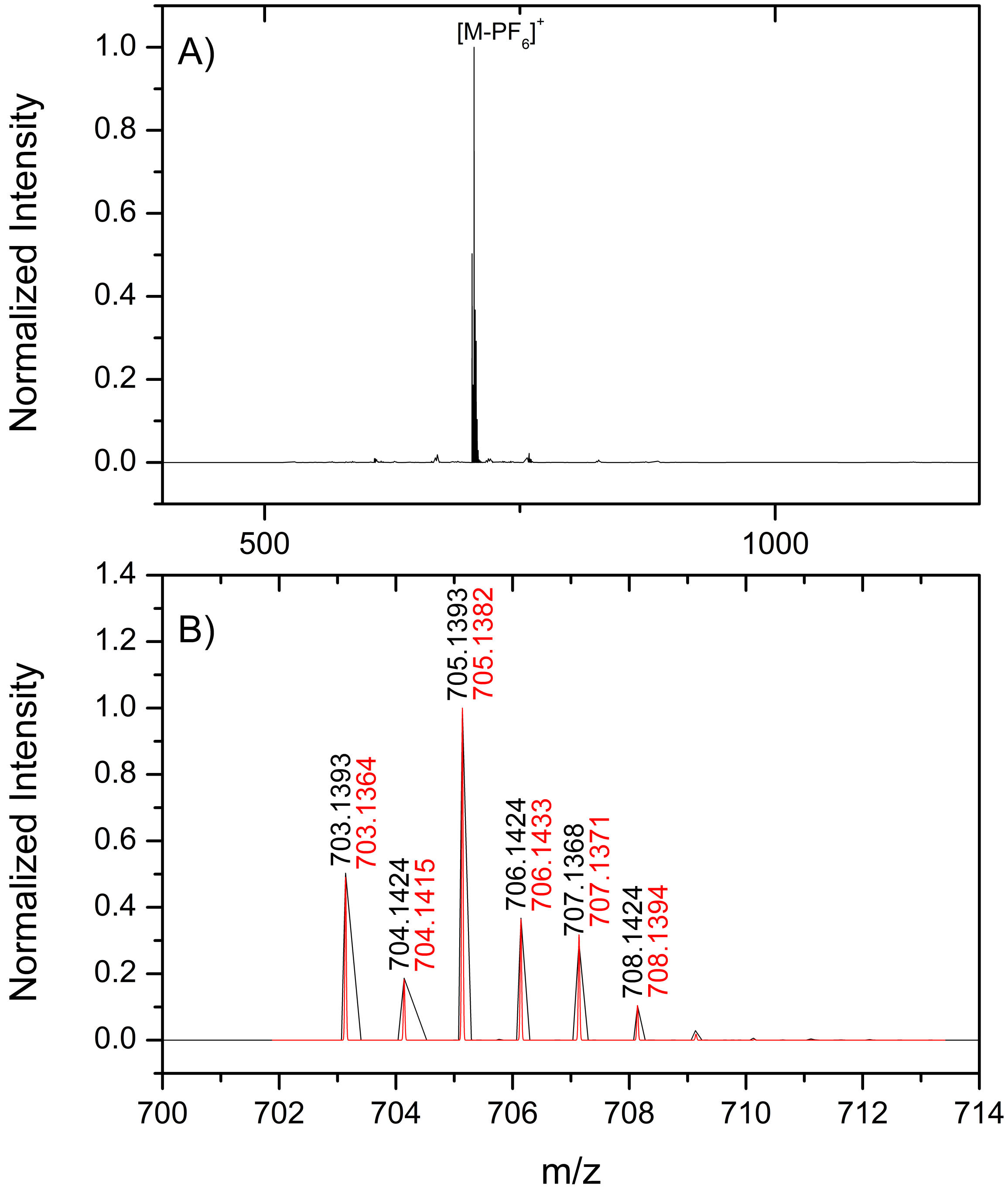

Figure S77: ESI-MS of [Ir(**4-MePhTerpy**)(ppy)Cl](PF_6). A) Displays the full MS window analyzed and B) is an expanded region a the [M-PF_6]$^+$ peak. The black traces are the experimental data while red trace is the simulated mass spectrum isotopic window.

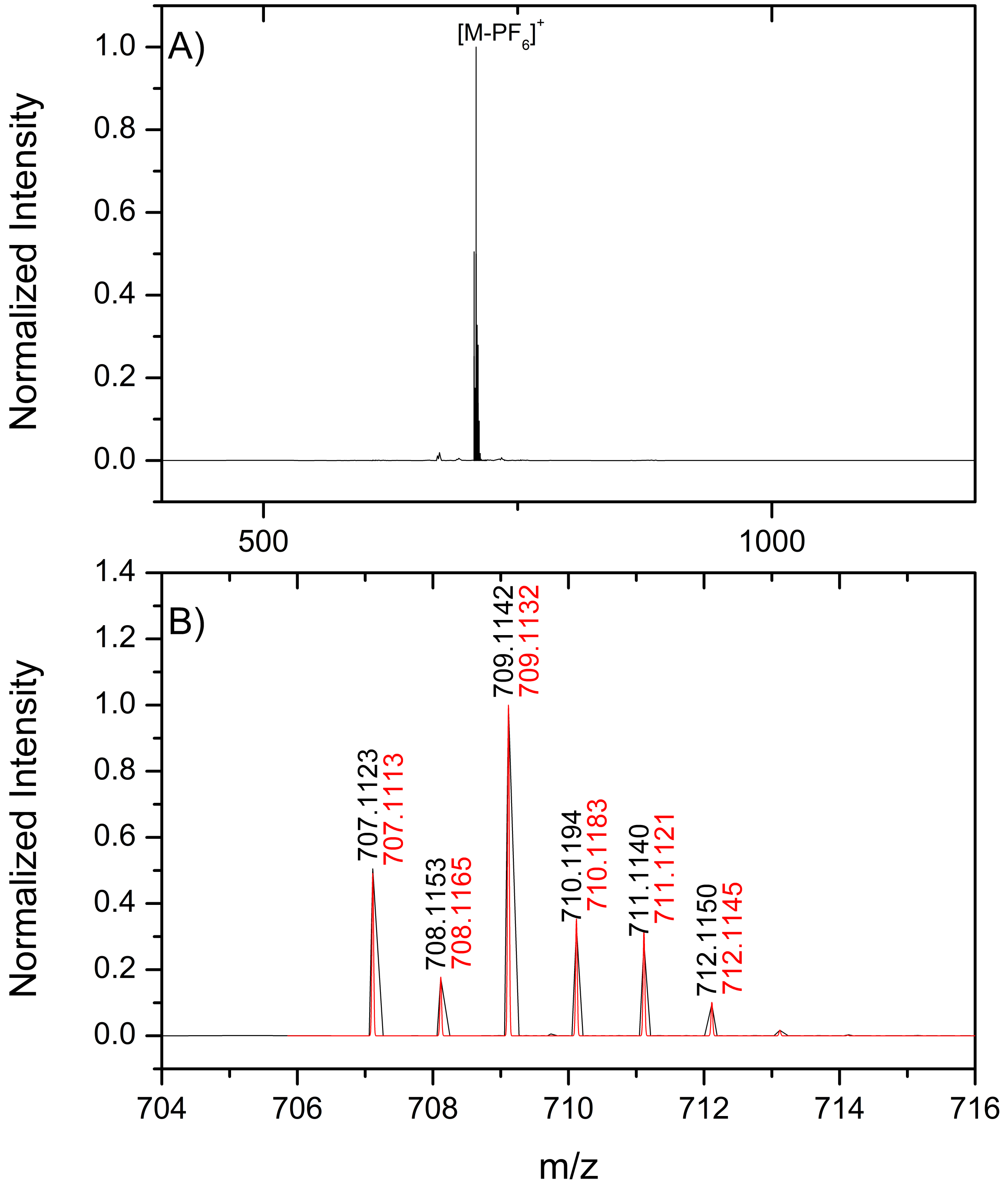

Figure S78: ESI-MS of [Ir(**4-FPhTerpy**)(ppy)Cl](PF_6). A) Displays the full MS window analyzed and B) is an expanded region a the $[M\text{-}PF_6]^+$ peak. The black traces are the experimental data while red trace is the simulated mass spectrum isotopic window.

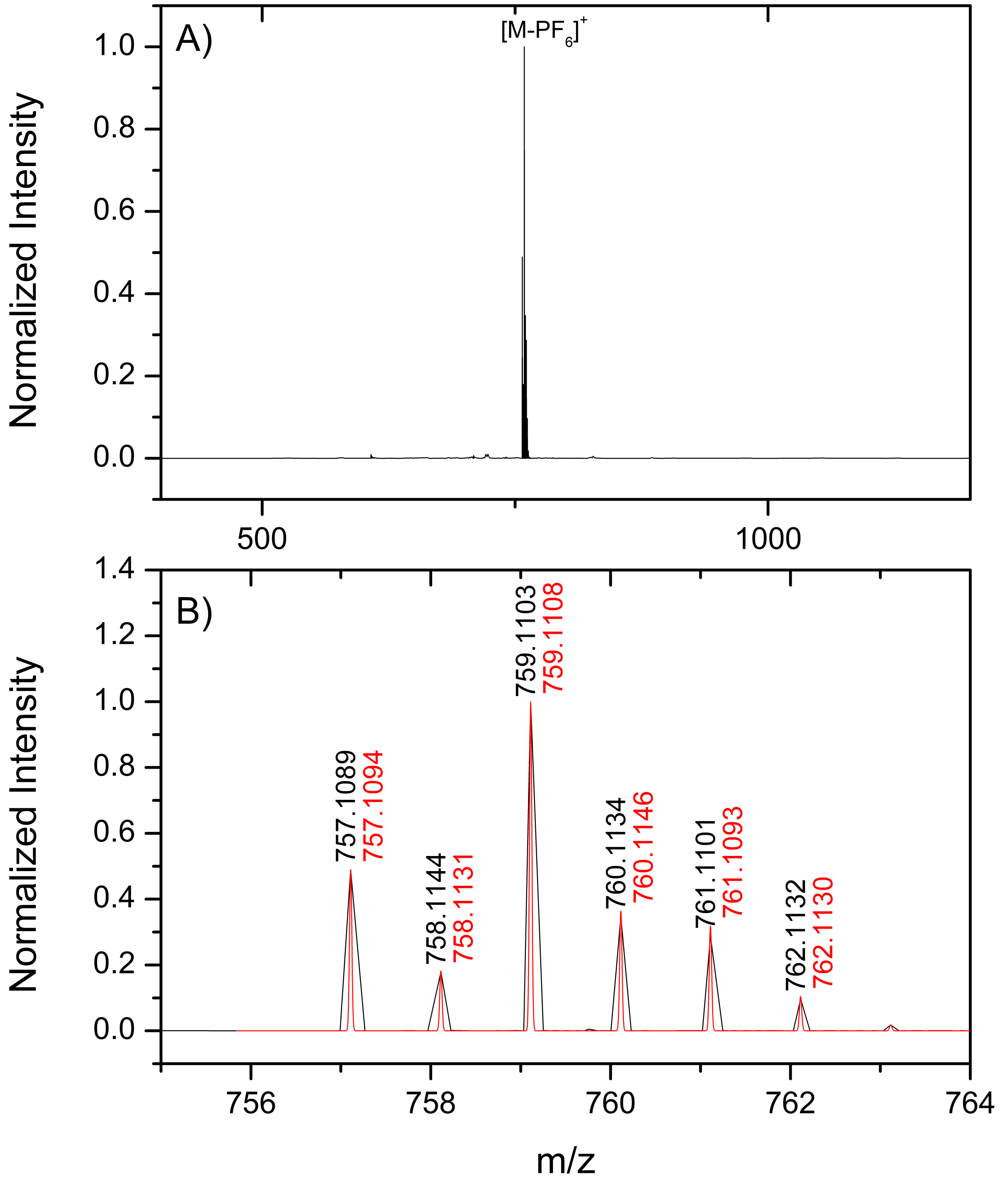

Figure S79: ESI-MS of [Ir(**4-CF$_3$PhTerpy**)(ppy)Cl](PF$_6$). A) Displays the full MS window analyzed and B) is an expanded region a the [M-PF$_6$]$^+$ peak. The black traces are the experimental data while red trace is the simulated mass spectrum isotopic window.

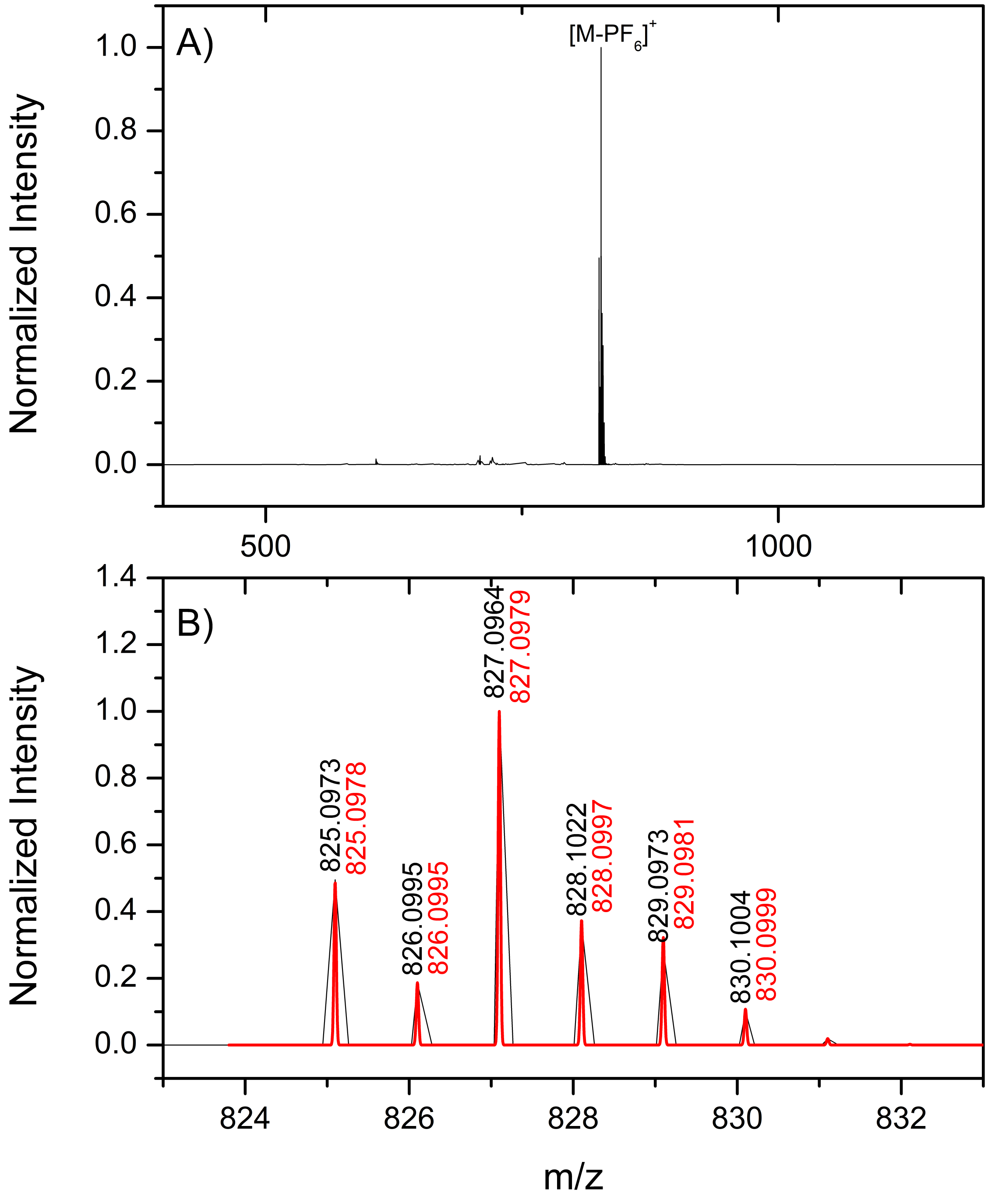

Figure S80: ESI-MS of [Ir(**3,5-CF$_3$PhTerpy**)(ppy)Cl](PF_6). A) Displays the full MS window analyzed and B) is an expanded region a the [M-PF_6]$^+$ peak. The black traces are the experimental data while red trace is the simulated mass spectrum isotopic window.

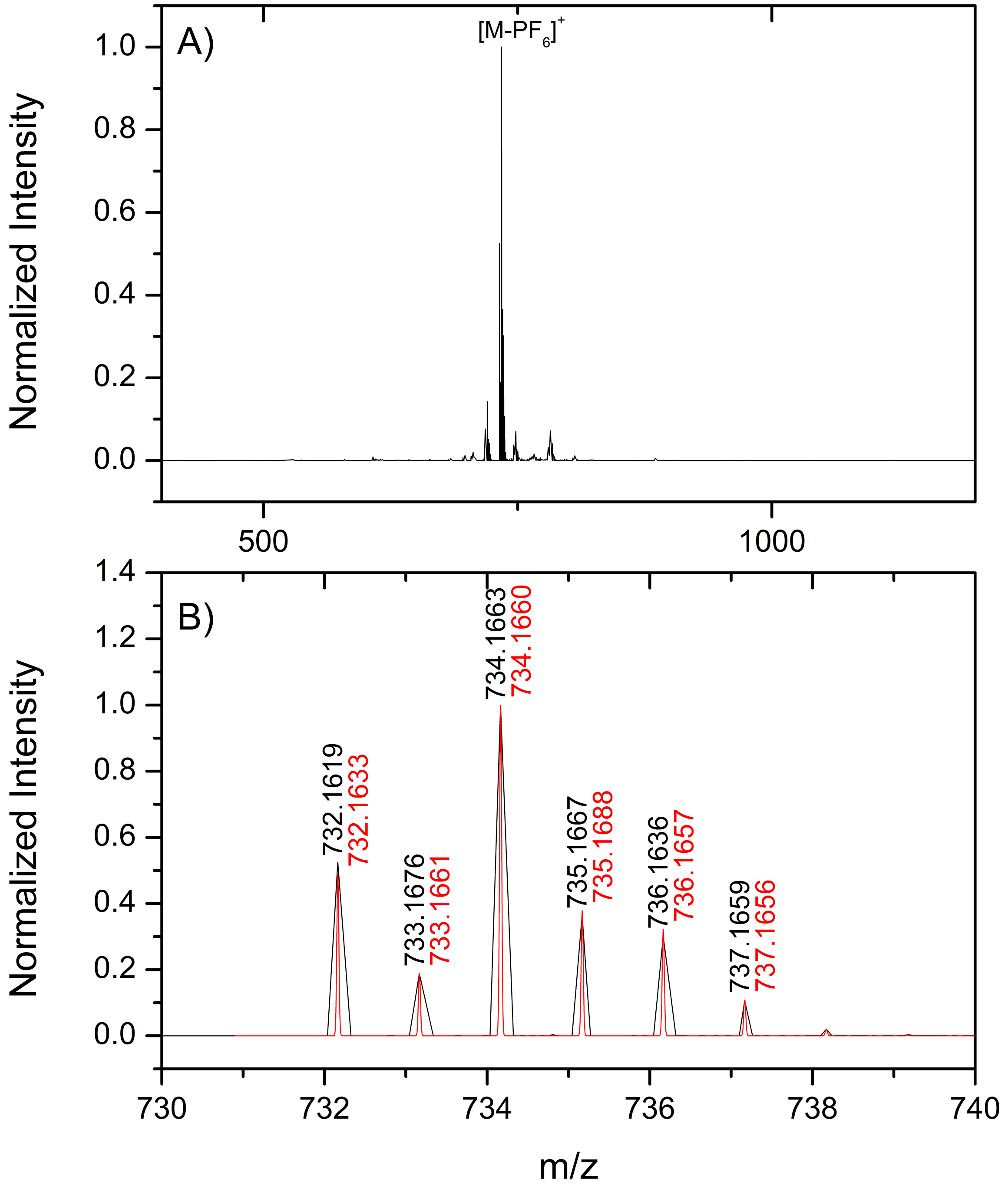

Figure S81: ESI-MS of [Ir(**4-NMe$_2$PhTerpy**)(ppy)Cl](PF_6). A) Displays the full MS window analyzed and B) is an expanded region a the [M-PF_6]$^+$ peak. The black traces are the experimental data while red trace is the simulated mass spectrum isotopic window.

References

(1) Conradie, J. A review of electrochemistry of osmium(II)-polypyridines and supporting DFT studies. *Electrochimica Acta* **2025**, *514*, 145633.

(2) Cummings, S. D.; Eisenberg, R. Tuning the Excited-State Properties of Platinum(II) Diimine Dithiolate Complexes. *Journal of the American Chemical Society* **1996**, *118*, 1949–1960.

(3) Bhaumik, C.; Das, S.; Saha, D.; Dutta, S.; Baitalik, S. Synthesis, Characterization, Photophysical, and Anion-Binding Studies of Luminescent Heteroleptic Bis-Tridentate Ruthenium(II) Complexes Based on 2,6-Bis(Benzimidazole-2-yl)Pyridine and 4-Substituted 2,2:6,2 Terpyridine Derivatives. *Inorganic Chemistry* **2010**, *49*, 5049–5062, PMID: 20469925.

(4) Skórka, Ł.; Filapek, M.; Zur, L.; Małecki, J. G.; Pisarski, W.; Olejnik, M.; Danikiewicz, W.; Krompiec, S. Highly Phosphorescent Cyclometalated Iridium(III) Complexes for Optoelectronic Applications: Fine Tuning of the Emission Wavelength through Ancillary Ligands. *The Journal of Physical Chemistry C* **2016**, *120*, 7284–7294.

(5) Quan, L. M.; Stringer, B. D.; Haghighatbin, M. A.; Agugiaro, J.; Barbante, G. J.; Wilson, D. J. D.; Hogan, C. F.; Barnard, P. J. Tuning the electrochemiluminescent properties of iridium complexes of N-heterocyclic carbene ligands. *Dalton Trans.* **2019**, *48*, 653–663.

(6) Palion-Gazda, J.; Kwiecień, A.; Choroba, K.; Penkala, M.; Erfurt, K.; Machura, B. Effect of the Appended Morpholinyl Group on Photophysical Behavior of Mono- and Bis-cyclometalated Terpyridine Iridium(III) Chromophores. *Inorganic Chemistry* **2025**, *64*, 646–661, PMID: 39725640.

(7) Chirdon, D. N.; Transue, W. J.; Kagalwala, H. N.; Kaur, A.; Maurer, A. B.; Pintauer, T.; Bernhard, S. [Ir(N^N^N)(C^N)L]+: A New Family of Luminophores Combining Tunability and Enhanced Photostability. *Inorganic Chemistry* **2014**, *53*, 1487–1499, PMID: 24437359.

(8) Garg, K.; Matsubara, Y.; Ertem, M. Z.; Lewandowska-Andralojc, A.; Sato, S.; Szalda, D. J.; Muckerman, J. T.; Fujita, E. Striking Differences in Properties of Geometric Isomers of $[Ir(tpy)(ppy)H]^{+}$: Experimental and Computational Studies of their Hydricities, Interaction with CO2, and Photochemistry. *Angewandte Chemie International Edition* **2015**, *54*, 14128–14132.

(9) Takizawa, S.-y.; Katoh, S.; Okazawa, A.; Ikuta, N.; Matsushima, S.; Zeng, F.; Murata, S. Triplet Excited States Modulated by Push–Pull Substituents in Monocyclometalated Iridium(III) Photosensitizers. *Inorganic Chemistry* **2021**, *60*, 4891–4903, PMID: 33715380.

(10) Pettersen, E. F.; Goddard, T. D.; Huang, C. C.; Couch, G. S.; Greenblatt, D. M.; Meng, E. C.; Ferrin, T. E. UCSF Chimera—A visualization system for exploratory research and analysis. *Journal of Computational Chemistry* **2004**, *25*, 1605–1612.
